# Supplementary material for: Upcycling poly(succinates) with amines to N-substituted succinimides over succinimide anion-based ionic liquids
Source: Nat Commun. 2024 Jan 24;15:712. doi: 10.1038/s41467-024-44892-1 (PMC10808099; doi:10.1038/s41467-024-44892-1)
Supplement: Supplementary file 1 — Supplementary Information [file 41467_2024_44892_MOESM1_ESM.pdf]

Supplementary Information

Upcycling poly(succinates) with amines to N-substituted succinimides over succinimide anion-based ionic liquids

*Fengtian Wu, Yuepeng Wang, Yanfei Zhao, Shaojuan Zeng, Zhenpeng Wang, Minhao Tang, Wei Zeng, Ying Wang, Xiaoqian Chang, Junfeng Xiang, Zongbo Xie, Buxing Han and Zhimin Liu**

1. Supplementary Figures and Supplementary Tables

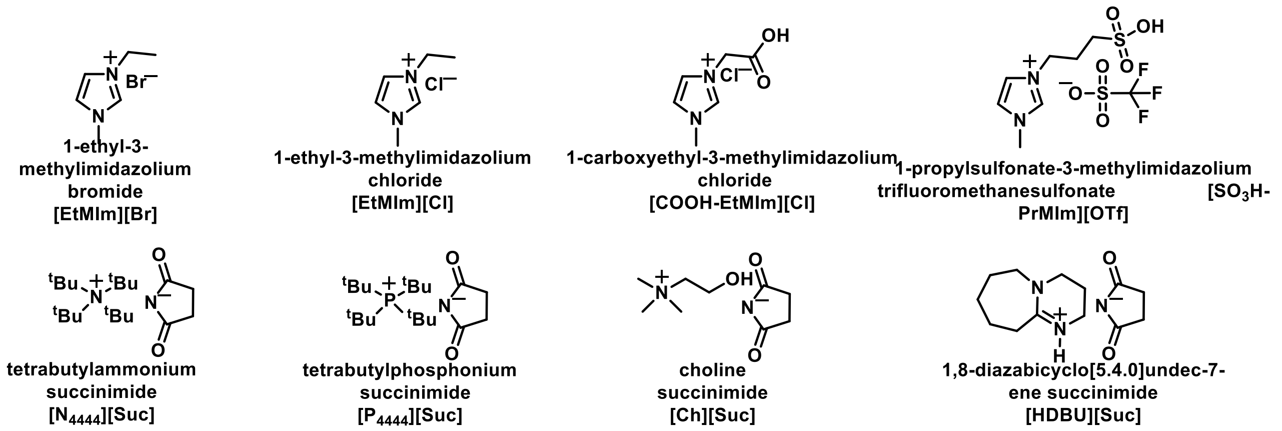

Supplementary Fig. 1. The ILs used in this study.

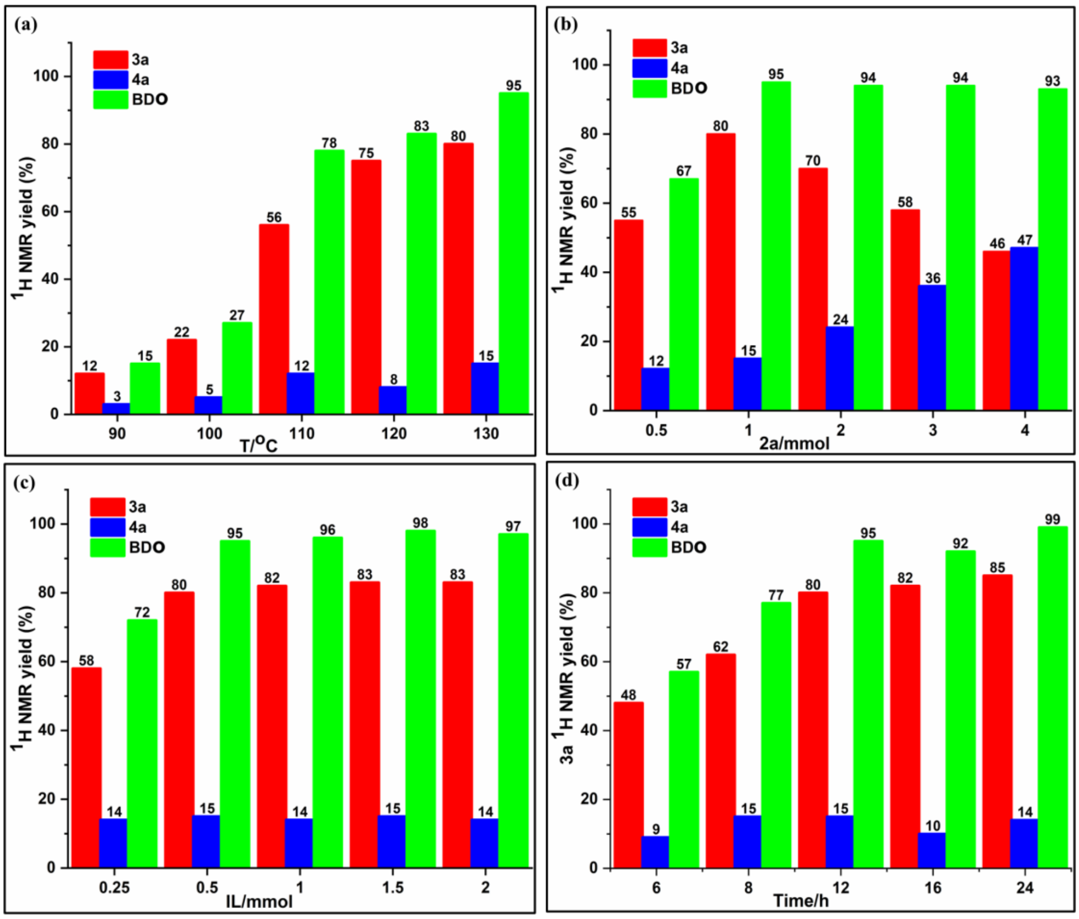

Supplementary Fig. 2. Screening reaction conditions. (a) effect of temperature on the reaction, (b) effect of amount of **2a** on the reaction, (c) effect of amount of IL on the reaction, (d) effect of time on the reaction.

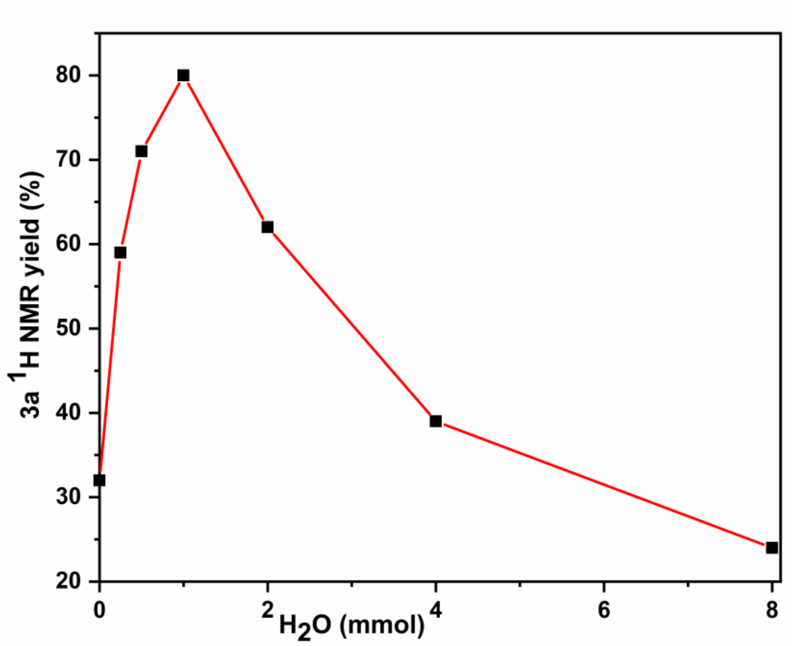

Supplementary Fig. 3. Dependence of **3a** yield on the amount of H_2O.

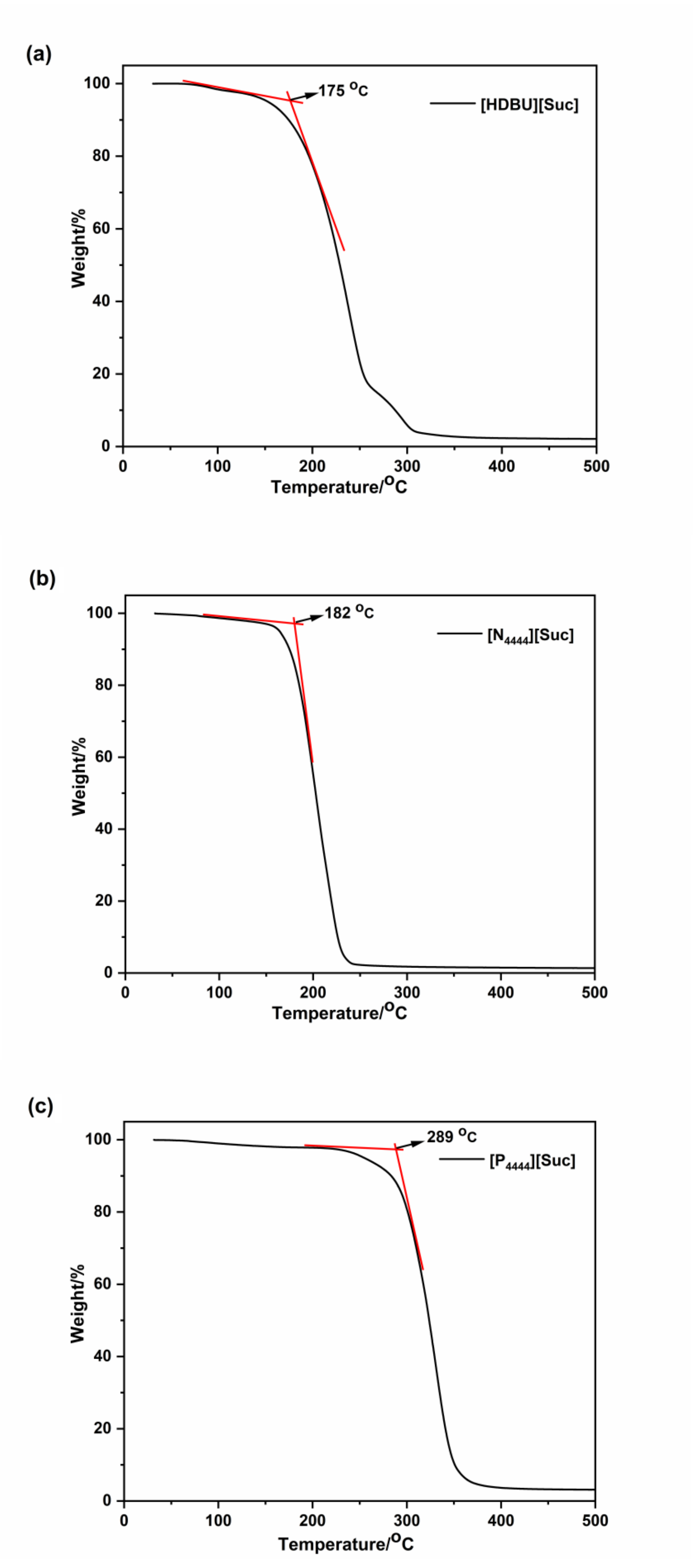

Supplementary Fig. 4. TGA curves of Ils: (a) [HDBU][Suc], (b) [N_{4444}][Suc] and (c) [P_{4444}][Suc]. Note: it was performed at 5 °C/min. with an N_2 sweep of 20 mL/min..

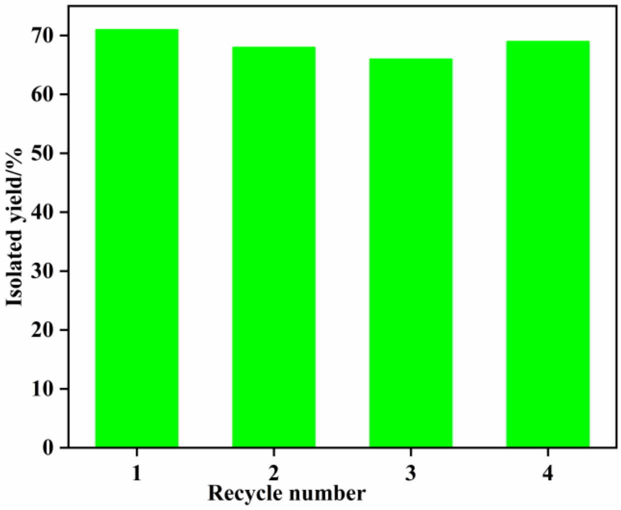

Supplementary Fig. 5. The recycling experiments of [HDBU][Suc]. Note: reaction conditions: PBS (0.5 mmol structural unit), **2h** (1.0 mmol), [HDUB][Suc] (0.5 mmol), H_2O (1.0 mmol), 130 ^oC, 24 h; first time: 71%, second time: 68%, third time: 66%, fourth time: 69%; To separate the IL from the reaction solution, water was first added to dilute the reaction solution, and then ethyl acetate was added to extract organic products including generated alcohol and succinimides for three times. The recovered ethyl acetate solutions were combined and evaporated to remove ethyl acetate, and the mixture of alcohol and succinimide was obtained. Followed by separation via gel chromatography, alcohol and succinimide were separated. The collected aqueous phase was evaporated to remove water and dried at 65 ^oC for 24 h in vacuum, obtaining IL, which was used for the next run.

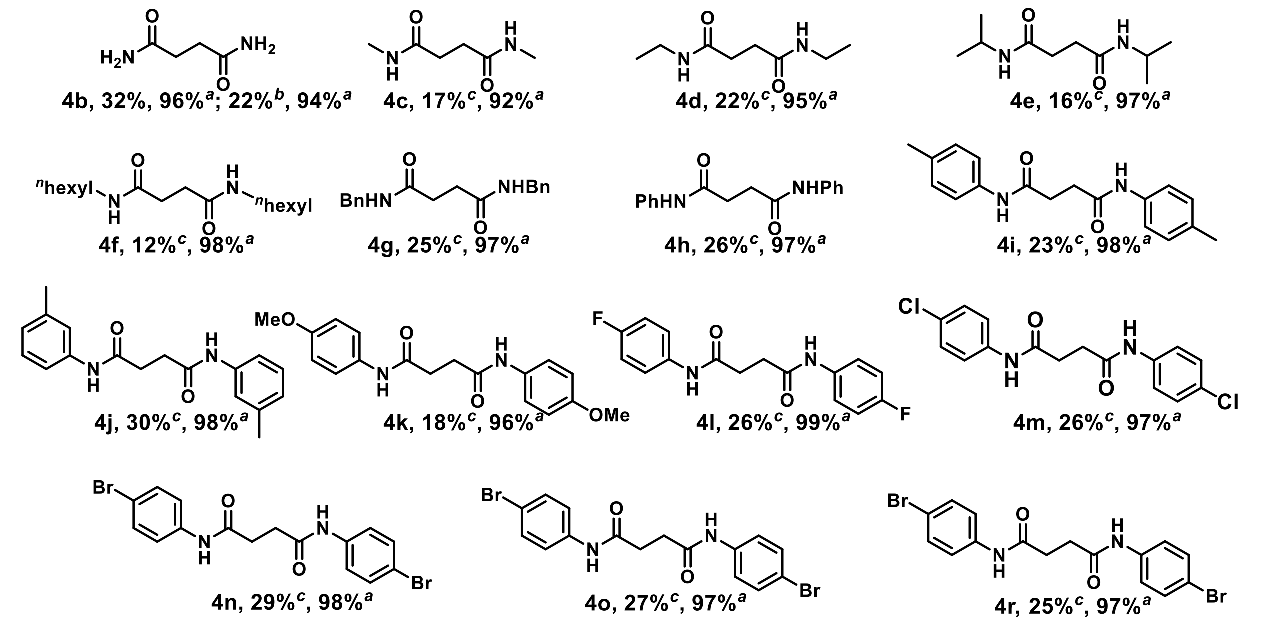

4b, 32%, 96%[a]; 22%[b], 94%[a] **4c, 17%[c], 92%[a]** **4d, 22%[c], 95%[a]** **4e, 16%[c], 97%[a]**

4f, 12%[c], 98%[a] **4g, 25%[c], 97%[a]** **4h, 26%[c], 97%[a]** **4i, 23%[c], 98%[a]**

4j, 30%[c], 98%[a] **4k, 18%[c], 96%[a]** **4l, 26%[c], 99%[a]** **4m, 26%[c], 97%[a]**

4n, 29%[c], 98%[a] **4o, 27%[c], 97%[a]** **4r, 25%[c], 97%[a]**

Supplementary Fig. 6. The yields of BDO and byproducts **4** derived from aminolysis of PBS. [a]The yield of BDO. [b]$[P_{4444}]$[Suc] as the IL. [c]24 h.

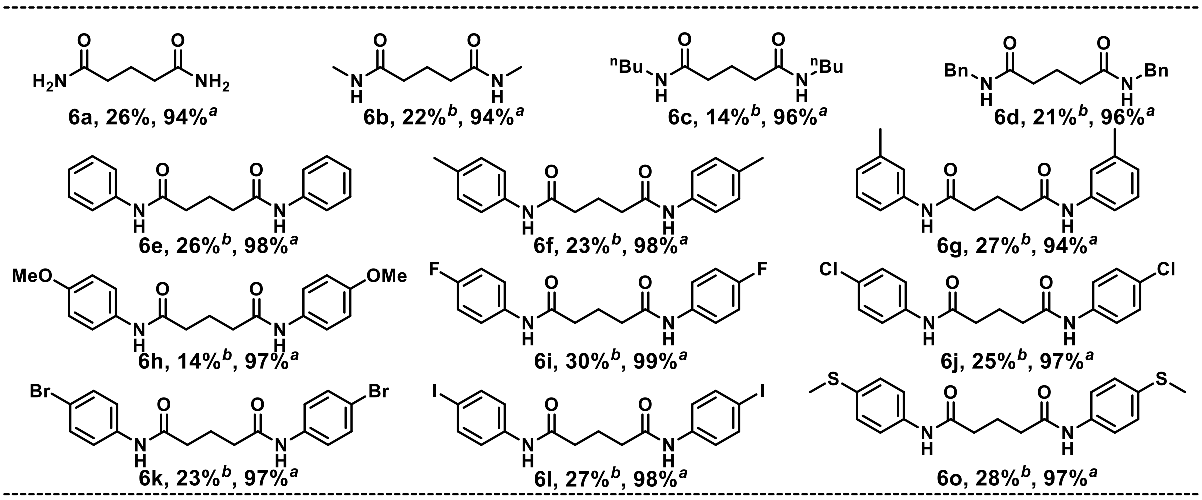

Supplementary Fig. 7. The byproduct **6** derived from aminolysis of PTG. [a]The yield of BDO. [b]24 h.

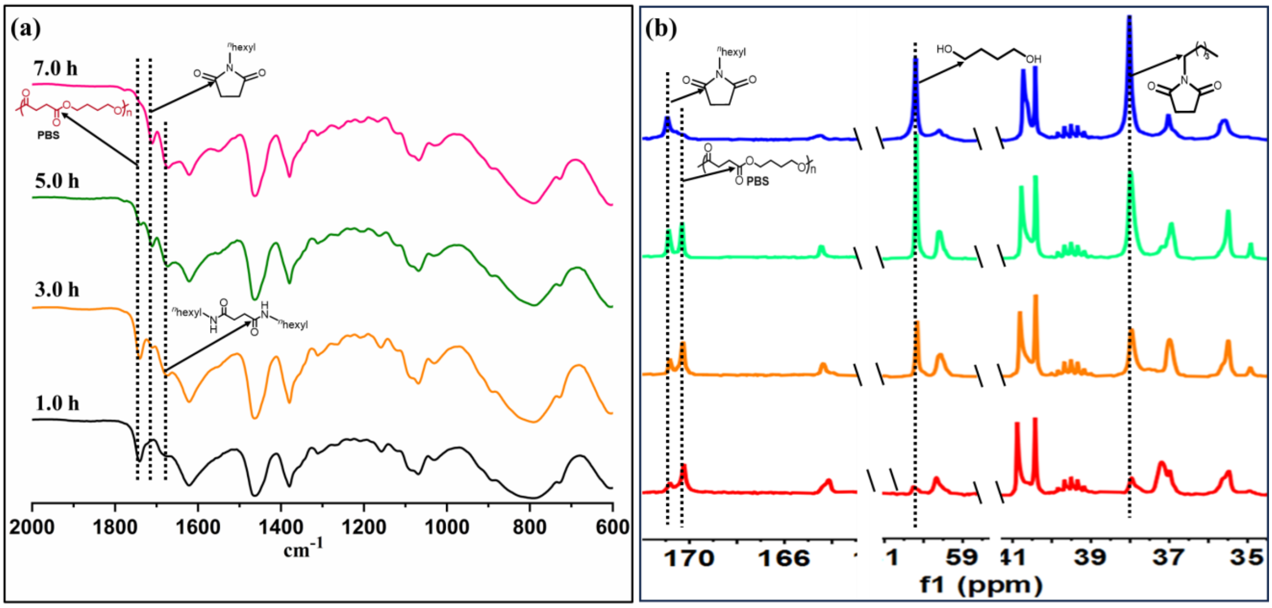

Supplementary Fig. 8. In situ IR spectra (a) and in situ ^{13}C NMR spectra (b) recorded in the reaction of PBS with hexylamine performed at 120 ºC.

Note: The IR stretching vibration absorbance about the carbonyl group of PBS at 1740 cm^{-1} gradually decreased with the increasing reaction time, indicating the decomposition of PBS occurred, meanwhile the IR stretching vibration absorbance of the carbonyl group at 1714 cm^{-1} became more and more intense, showing that the formation of product **3f**. A new IR stretching vibration absorbance at 1673 cm^{-1} was observed, belonging to the carbonyl group in byproduct N′, N′-dihexylsuccinamide.

For NMR analysis, the chemical shift at 39.5 ppm for C in DMSO was taken as the internal standard. From the in-situ NMR analysis, it is observed that the altitude of ^{13}C NMR resonances (δ = 170.94 ppm) assigning to the carbonyl C gradually enhanced with the time, and the altitude of ^{13}C NMR resonances (δ = 170.23 ppm) assigning to the carbonyl C in PBS decreased. Moreover, both the altitude of ^{13}C NMR resonances (δ = 60.13 ppm) assigning to the C atom linking with hydroxyl group in butanediol and the C atom (δ = 37.96 ppm) linking with N atom in **3f** gradually increased with reaction time. All these findings indicate the formation of product **3f**.

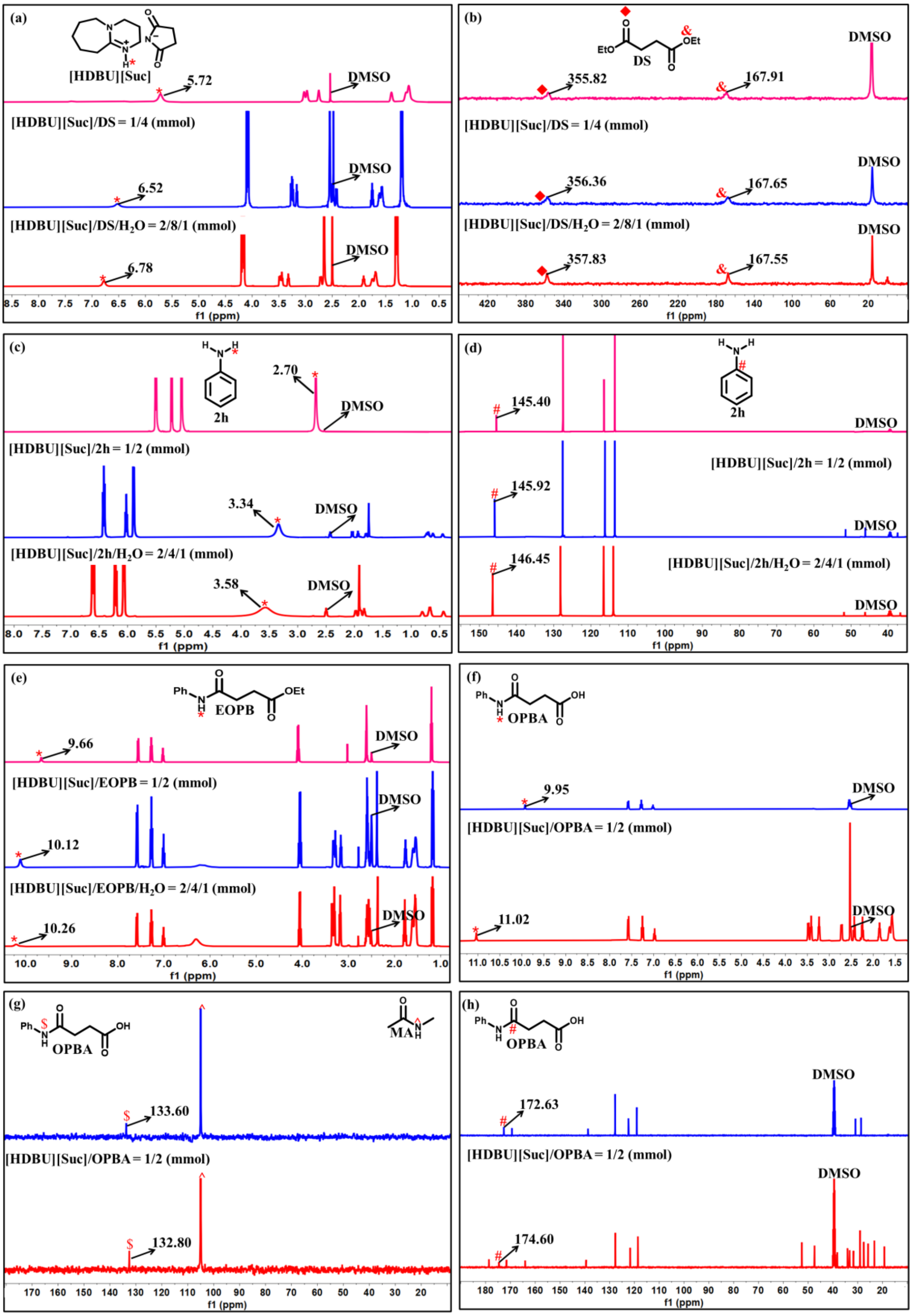

Supplementary Fig. 9. NMR analysis for mechanism investigation. (a) ^{1}H NMR spectra of [HDBU][Suc], [HDBU][Suc]/DS, [HDBU][Suc]/DS/H_2O recorded at 60 °C. (b) ^{17}O NMR spectra of DS, [HDBU][Suc]/DS, [HDBU][Suc]/DS/H_2O recorded at 60 °C. (c) ^{1}H NMR spectra of **2h**, [HDBU][Suc]/**2h**, [HDBU][Suc]/**2h**/H_2O recorded at 60 °C. (d) ^{13}C NMR spectra of **2h**, [HDBU][Suc]/**2h**, [HDBU][Suc]/**2h**/H_2O recorded at 60 °C. (e) ^{1}H NMR spectra of EOPB, [HDBU][Suc]/EOPB, [HDBU][Suc]/EOPB/H_2O recorded at 60 °C. (f) ^{1}H NMR spectra of OPBA, [HDBU][Suc]/OPBA, [HDBU][Suc]/OPBA/H_2O recorded at 60 °C. (g) ^{15}N NMR spectra of OPBA, [HDBU][Suc]/OPBA, [HDBU][Suc]/OPBA/H_2O recorded at 60 °C. (h) ^{13}C NMR spectra of OPBA, [HDBU][Suc]/OPBA, [HDBU][Suc] /OPBA/H_2O recorded at 60 °C. Note: 2.50 ppm of H in DMSO was taken as the internal standard in a, c, e and f, 16.00 ppm of O in DMSO as the internal standard in b, 105.00 ppm for N in N-methylacetamide as the internal standard in c and g, and 39.50 ppm of C in DMSO as the internal standard in d and h.

The detailed description for the chemical shift changes are shown as follows:

(a) From the ^{1}H NMR spectra of [HDBU][Suc], [HDBU][Suc]/DS, [HDBU][Suc]/DS/H_2O recorded, it is clear that the chemical shifts assigning to the H atom linking with N ion of [HDBU] in the mixtures shifted from 5.72 ppm for pure IL to 6.52 ppm for the IL/DS mixture, and further to 6.78 ppm in IL/DS/H_2O mixture, indicating that the electron cloud density of the H atom linking with N ion in IL decreased.
(b) From the ^{17}O NMR spectra of DS, [HDBU][Suc]/DS, [HDBU][Suc]/DS/H_2O, it is clear that the chemical shifts assigning to the carbonyl O atom of DS in the mixtures shifted from 355.82 ppm for pure DS to 356.36 ppm for the IL/DS mixture, and further to 357.83 ppm in IL/DS/H_2O mixture, indicating that the electron cloud density of the carbony O atom was enhanced. The chemical shift assigning to the O atom linking with Et group in DS is not obvious, indicating that this O atom was little affected.
(c) From the ^{1}H NMR spectra of **2h**, [HDBU][Suc]/**2h**, [HDBU][Suc]/**2h**/H_2O, it is clear that the chemical shifts assigning to the H atom in -NH_2 of **2h** in the mixtures shifted from 2.70 ppm for pure **2h** to 3.34 ppm for the IL/**2h** mixture, and further to 3.58 ppm for the IL/**2h**/H_2O mixture, indicating that the electron cloud density of the N atom in -NH_2 of **2h** decreased.
(d) From the ^{13}C NMR spectra of **2h**, [HDBU][Suc]/**2h**, [HDBU][Suc]/**2h**/H_2O, it is clear that the chemical shifts assigning to the N atom of **2h** in the mixtures shifted from 145.40 ppm for pure **2h** to 145.92 ppm for the IL/**2h** mixture, and further to 146.45 ppm for the IL/**2h**/H_2O mixture, indicating that the electron cloud density of the N atom in **2h** was enhanced.
(e) From the ^{1}H NMR spectra of EOPB, [HDBU][Suc]/EOPB, [HDBU][Suc]/EOPB/H_2O, it is clear that the chemical shifts assigning to the H atom in -NH- of EOPB in the mixtures shifted from 9.66 ppm for pure EOPB to 10.12 ppm for the IL/EOPB mixture, and further to 10.26 ppm for the IL/EOPB/H_2O mixture, indicating that the electron cloud density of the N atom in -NH- of EOPB was decreased.
(f) From the ^{1}H NMR spectra of OPBA, [HDBU][Suc]/OPBA, it is clear that the chemical shifts assigning to the H atom linking with N ion of [HDBU] in the mixtures shifted from 9.95 ppm for pure IL to 11.02 ppm for the IL/OPBA mixture, indicating that the electron cloud density of the H atom linking with N ion in IL was decreased.
(g) From the ^{15}N NMR spectra of OPBA, [HDBU][Suc]/OPBA, it is clear that the chemical shifts assigning to the N atom of OPBA in the mixtures shifted from 133.60 ppm for pure OPBA to 132.80 ppm for the IL/OPBA mixture, indicating that the electron cloud density of the N atom in OPBA was enhanced.
(h) From the ^{13}C NMR spectra of OPBA, [HDBU][Suc]/OPBA, it is clear that the chemical shifts assigning to the carbonyl C atom of OPBA in the mixtures shifted from 172.63 ppm for pure OPBA to 174.60 ppm for the IL/OPBA mixture, indicating that the electron cloud density of the C atom in OPBA was decreased.

Supplementary Table 1. Comparison of the Kamlet-Taft parameters of succinimide anion-based ILs.

ILs	*α*	*β*	*π*
[N_{4444}][Suc]	1.42	0.60	1.55
[P_{4444}][Suc]	1.47	0.79	1.42
[Ch][Suc]	1.59	0.45	1.47
[HDBU][Suc]	1.61	0.57	1.52

2. Supplementary Data

Ethyl 4-oxo-4-(phenylamino)butanoate[1] (EOPB)

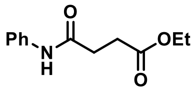

^{1}H NMR (400 MHz, DMSO-d_6, 25 °C): δ = 9.98 (s, 1H), 7.57-7.59 (m, 2H), 7.27-7.31 (m, 2H), 7.00-7.04 (m, 1H), 4.06 (q, J = 7.2 Hz, 3H), 2.60 (s, 4H), 1.18 ppm (t, J = 7.2 Hz, 3H); ^{13}C NMR (101 MHz, DMSO-d_6, 25 °C): δ = 171.6, 169.2, 138.7, 128.0, 122.5, 119.0, 59.3, 30.7, 28.7, 13.5 ppm.

Tetrabutylphosphonium succinimide[2] [P_{4444}][Suc]

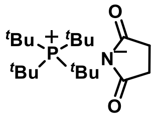

^{1}H NMR (400 MHz, DMSO-d_6, 25 °C): δ = 3.18-3.22 (m, 8H), 2.06 (s, 4H), 1.56 (m, 8H), 1.29-1.34 (m, 8H), 0.93 ppm (t, J = 7.2 Hz, 12H); ^{13}C NMR (101 MHz, DMSO-d_6, 25 °C): δ = 194.3, 32.9, 23.9, 23.7, 23.2， 23.1, 18.1, 17.5, 13.7 ppm.

Tetrabutylammonium succinimide[2] [N_{4444}][Suc]

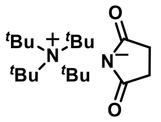

^{1}H NMR (400 MHz, DMSO-d_6, 25 °C): δ = 2.21-2.29 (m, 8H), 2.09 (s, 4H), 1.36-1.51 (m, 16H), 0.91 ppm (t, J = 7.2 Hz, 12H); ^{13}C NMR (101 MHz, DMSO-d_6, 25 °C): δ = 195.0, 58.0, 33.04, 23.6, 19.7, 14.0 ppm.

Choline succinimide[2] [Ch][Suc]

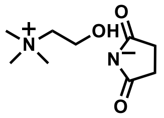

^{1}H NMR (400 MHz, DMSO-d_6, 25 °C): δ = 3.87 (s, 2H), 3.45-3.53 (m, 2H), 3.16 (s, 9H), 2.18 ppm (s, 4H); ^{13}C NMR (101 MHz, DMSO-d_6, 25 °C): δ = 194.0, 72.6, 55.1, 53.5, 32.6 ppm.

8-Diazabicyclo[5.4.0]undec-7-ene succinimide[2] [HDBU][Suc]

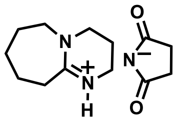

^{1}H NMR (400 MHz, DMSO-d_6, 25 °C): δ = 3.31-3.38 (m, 2H), 3.17-3.21 (m, 2H), 2.57-2.60 (m, 2H), 2.34 (s, 4H), 1.75-1.83 (m, 2H), 1.56-1.63 ppm (m, 6H); ^{13}C NMR (101 MHz, DMSO-d_6, 25 °C): δ = 187.7, 163.5, 53.0, 49.0, 48.1, 33.6, 31.5, 29.2, 27.5, 25.1, 20.9 ppm.

1-Butylpyrrolidine-2,5-dione[3] (3a)

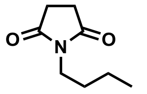

^{1}H NMR (400 MHz, DMSO-d_6, 25 °C): δ = 3.46 (t, J = 10.0 Hz, 2H), 2.68 (s, 4H), 1.47-1.57 (m, 2H), 1.25-1.37 (m, 2H), 0.93 ppm (t, J = 9.6 Hz, 3H); ^{13}C NMR (101 MHz, DMSO-d_6, 25 °C): δ = 178.6, 37.9, 29.4, 27.7, 19.7, 12.6 ppm.

Pyrrolidine-2,5-dione[4] (3b)

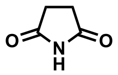

^{1}H NMR (400 MHz, DMSO-d_6, 25 °C): δ = 4.70 (s, 1H), 2.74 ppm (s, 4H); ^{13}C NMR (101 MHz, DMSO-d_6, 25 °C): δ = 183.2, 29.5 ppm.

1-Methylpyrrolidine-2,5-dione[5] (3c)

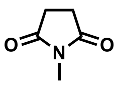

^{1}H NMR (400 MHz, DMSO-d_6, 25 °C): δ = 2.81 (s, 3H), 2.62 ppm (s, 4H); ^{13}C NMR (101 MHz, DMSO-d_6, 25 °C): δ = 183.2, 29.5 ppm.

1-Ethylpyrrolidine-2,5-dione[6] (3d)

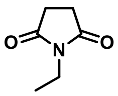

^{1}H NMR (400 MHz, DMSO-d_6, 25 °C): δ = 3.38 (q, J = 9.6 Hz, 2H), 2.61 (s, 4H), 1.04 ppm (t, J = 9.6 Hz, 3H); ^{13}C NMR (101 MHz, DMSO-d_6, 25 °C): δ = 178.0, 33.2, 28.5, 13.2 ppm.

1-Isopropylpyrrolidine-2,5-dione[7] (3e)

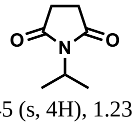

^{1}H NMR (400 MHz, DMSO-d_6, 25 °C): δ = 4.11-4.18 (m, 1H), 2.45 (s, 4H), 1.23 (s, 3H), 1.21 ppm (s, 3H); ^{13}C NMR (101 MHz, DMSO-d_6, 25 °C): δ = 179.9, 42.9, 30.0, 19.42 ppm.

1-Hexylpyrrolidine-2,5-dione[3] (3f)

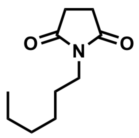

^{1}H NMR (400 MHz, DMSO-d_6, 25 °C): δ = 3.24-3.33 (m, 2H), 2.54 (s, 4H), 1.36-1.41 (m, 2H), 1.17-1.19 (m, 6H), 0.77-0.80 ppm (m, 3H); ^{13}C NMR (101 MHz, DMSO-d_6, 25 °C): δ = 178.1, 38.2, 31.3, 28.4, 27.6, 26.4, 22.4, 14.3 ppm.

1-Benzylpyrrolidine-2,5-dione[3] (3g)

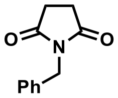

^{1}H NMR (400 MHz, DMSO-d_6, 25 °C): δ = 7.24-7.34 (m, 5H), 4.54 (s, 2H), 2.69 ppm (s, 4H); ^{13}C NMR (101 MHz, DMSO-d_6, 25 °C): δ = 178.1, 136.8, 128.9, 128.0, 127.8, 41.8, 28.6 ppm.

1-Phenylpyrrolidine-2,5-dione[4] (3h)

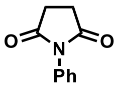

^{1}H NMR (400 MHz, DMSO-d_6, 25 °C): δ = 7.47-7.52 (m, 2H), 7.39-7.42 (m, 2H), 7.25-7.27 (m, 1H), 2.78 ppm (s, 4H); ^{13}C NMR (101 MHz, DMSO-d_6, 25 °C): δ = 177.4, 134.6, 133.2, 129.3, 128.6, 127.8, 127.6, 28.9 ppm.

1-(*p*-Tolyl)pyrrolidine-2,5-dione[8] (3i)

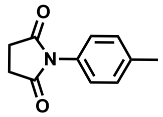

^{1}H NMR (400 MHz, DMSO-d_6, 25 °C): δ = 7.29-7.31 (m, 2H), 7.14-7.16 (m, 2H), 2.77 (s, 4H), 2.35 ppm (s, 3H); ^{13}C NMR (101 MHz, DMSO-d_6, 25 °C): δ = 177.5, 138.1, 130.6, 129.8, 127.3, 28.9, 21.2 ppm.

1-(*m*-Tolyl)pyrrolidine-2,5-dione[10] (3j)

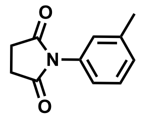

^{1}H NMR (400 MHz, DMSO-d_6, 25 °C): δ = 7.34-7.39 (m, 1H), 7.21-7.23 (m, 1H), 7.03-7.06 (m, 2H), 2.77 (s, 4H), 2.34 ppm (s, 3H); ^{13}C NMR (101 MHz, DMSO-d_6, 25 °C): δ = 177.4, 138.7, 133.2, 129.3, 129.1, 128.0, 124.7, 28.9, 21.3 ppm.

1-(4-Methoxyphenyl)pyrrolidine-2,5-dione[4] (3k)

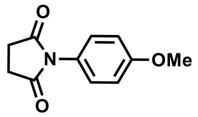

^{1}H NMR (400 MHz, DMSO-d_6, 25 °C): δ = 7.15-7.17 (m, 2H), 7.01-7.03 (m, 2H), 3.78 (s, 3H), 2.75 ppm (s, 4H); ^{13}C NMR (101 MHz, DMSO-d_6, 25 °C): δ = 177.6, 159.3, 128.8, 125.8, 114.5, 55.8, 28.9 ppm.

1-(4-Fluorophenyl)pyrrolidine-2,5-dione[7] (3l)

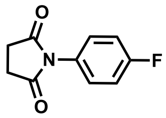

^{1}H NMR (400 MHz, DMSO-d_6, 25 °C): δ = 7.31-7.33 (m, 4H), 2.77 ppm (s, 4H); ^{13}C NMR (101 MHz, DMSO-d_6, 25 °C): δ = 177.4, 163.4, 160.2, 129.7 ($J_{C\text{-}F}$ = 12 Hz), 129.4 ($J_{C\text{-}F}$ = 4 Hz), 116.2 ($J_{C\text{-}F}$ = 30 Hz), 28.9 ppm; ^{19}F NMR (400 MHz, DMSO-d6, 25 °C): δ = -113.6 ppm.

1-(4-Chlorophenyl)pyrrolidine-2,5-dione[4] (3m)

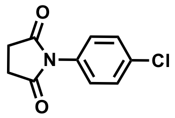

^{1}H NMR (400 MHz, DMSO-d_6, 25 °C): δ = 7.55-7.58 (m, 2H), 7.30-7.33 (m, 2H), 2.78 ppm (s, 4H); ^{13}C NMR (101 MHz, DMSO-d_6, 25 °C): δ = 177.2, 133.1, 132.1, 129.4, 129.3, 29.0 ppm.

1-(4-Bromophenyl)pyrrolidine-2,5-dione[8] (3n)

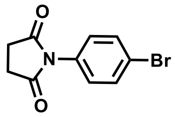

^{1}H NMR (400 MHz, DMSO-d_6, 25 °C): δ = 7.68-7.71 (m, 2H), 7.23-7.26 (m, 2H), 2.77 ppm (s, 4H); ^{13}C NMR (101 MHz, DMSO-d_6, 25 °C): δ = 177.2, 132.5, 132.3, 129.6, 121.6, 29.0 ppm.

1-(4-Iodophenyl)pyrrolidine-2,5-dione[10] (3o)

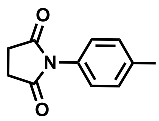

^{1}H NMR (400 MHz, DMSO-d_6, 25 °C): δ = 7.84-7.86 (m, 2H), 7.07-7.09 (m, 2H), 2.77 ppm (s, 4H); ^{13}C NMR (101 MHz, DMSO-d_6, 25 °C): δ = 177.1, 138.2, 133.0, 129.7, 29.0 ppm.

1-(4-(Methylthio)phenyl)pyrrolidine-2,5-dione[11] (3r)

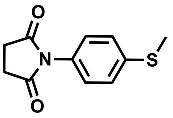

^{1}H NMR (400 MHz, DMSO-d_6, 25 °C): δ = 7.34-7.37 (m, 2H), 7.18-7.21 (m, 2H), 2.76 (s, 4H), 2.50 ppm (s, 3H); ^{13}C NMR (101 MHz, DMSO-d_6, 25 °C): δ = 177.4, 138.9, 129.9, 128.0, 126.5, 28.9, 15.2 ppm.

***N, N'*-Dibutylsuccinamide[12] (4a)**

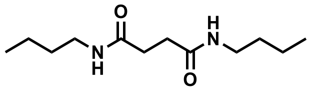

^{1}H NMR (400 MHz, DMSO-d_6, 25 °C): δ = 7.76 (s, 2H), 3.01 (q, *J* = 8.8 Hz, 4H), 2.27 (s, 4H), 1.19-1.39 (m, 8H), 0.85 ppm (t, *J* = 7.6 Hz, 3H); ^{13}C NMR (101 MHz, DMSO-d_6, 25 °C): δ = 171.6, 38.6, 31.7, 31.5, 20.0, 14.1 ppm.

Succinamide[12] (4b)

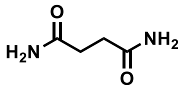

^{1}H NMR (400 MHz, DMSO-d_6, 25 °C): δ = 7.26 (s, 2H), 6.72 (s, 2H), 2.26 ppm (s, 4H); ^{13}C NMR (101 MHz, DMSO-d_6, 25 °C): δ = 174.5, 35.0, 21.6 ppm.

***N, N'*-Dimethylsuccinamide[12] (4c)**

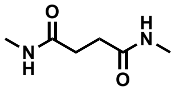

^{1}H NMR (400 MHz, DMSO-d_6, 25 °C): δ = 3.49 (s, 6H), 2.45 ppm (s, 4H); ^{13}C NMR (101 MHz, DMSO-d_6, 25 °C): δ = 172.9, 28.8, 28.5 ppm.

***N, N'*-diethylsuccinamide[12] (4d)**

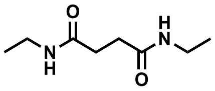

^{1}H NMR (400 MHz, DMSO-d_6, 25 °C): δ = 7.83 (s, 2H), 2.99-3.08 (m, 4H), 2.26 (s, 4H), 0.99 ppm (t, *J* = 9.6 Hz, 6H); ^{13}C NMR (101 MHz, DMSO-d_6, 25 °C): δ = 171.5, 33.7, 33.3, 15.2 ppm.

***N, N'*-Diisopropylsuccinamide[12] (4e)**

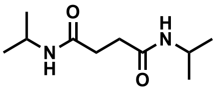

^{1}H NMR (400 MHz, DMSO-d_6, 25 °C): δ = 7.65 (d, *J* = 9.2 Hz, 2H), 3.73-3.85 (m, 2H), 2.23 (s, 4H), 1.01 ppm (d, *J* = 8.8 Hz, 12H); ^{13}C NMR (101 MHz, DMSO-d_6, 25 °C): δ = 170.8, 31.5, 29.2, 22.9 ppm.

***N, N'*-Dihexylsuccinamide[12] (4f)**

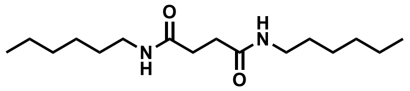

^{1}H NMR (400 MHz, DMSO-d_6, 25 °C): δ = 7.76 (s, 2H), 2.97-3.02 (m, 4H), 2.26 (s, 4H), 1.23-1.35 (m, 16H), 0.86 ppm (t, *J* = 10.0 Hz, 6H); ^{13}C NMR (101 MHz, DMSO-d_6, 25 °C): δ = 171.6, 31.4, 31.3, 29.6, 26.5, 22.5, 14.4 ppm.

***N, N'*-Dibenzylsuccinamide[13] (4g)**

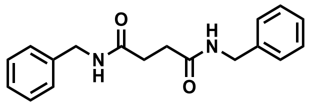

^{1}H NMR (400 MHz, DMSO-d_6, 25 °C): δ = 8.36 (t, *J* = 8.0 Hz, 2H), 7.20-7.33 (m, 10H), 4.25 (d, *J* = 10.0 Hz, 4H), 2.42 ppm (s, 4H); ^{13}C NMR (101 MHz, DMSO-d_6, 25 °C): δ = 171.8, 140.1, 128.7, 127.6, 127.1, 42.5, 31.2 ppm.

***N, N'*-Diphenylsuccinamide[14] (4h)**

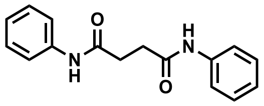

^{1}H NMR (400 MHz, DMSO-d_6, 25 °C): δ = 10.00 (s, 2H), 7.58-7.60 (m, 4H), 7.26-7.30 (m, 4H), 6.99-7.03 (m, 2H), 2.65 ppm (s, 4H); ^{13}C NMR (101 MHz, DMSO-d_6, 25 °C): δ = 170.8, 139.8, 130.2, 129.1, 123.4, 122.6, 119.4, 31.7 ppm.

***N, N'*-Di-*p*-tolylsuccinamide[15] (4i)**

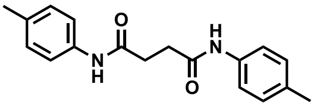

^{1}H NMR (400 MHz, DMSO-d_6, 25 °C): δ = 9.89 (s, 2H), 7.46-7.48 (m, 4H), 7.07-7.09 (m, 4H), 2.62 (s, 4H), 2.23 ppm (s, 6H); ^{13}C NMR (101 MHz, DMSO-d_6, 25 °C): δ = 170.6, 137.3, 132.2, 129.5, 119.4, 31.7, 20.9 ppm.

***N, N'*-Di-*m*-tolylsuccinamide[15] (4j)**

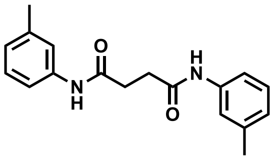

^{1}H NMR (400 MHz, DMSO-d_6, 25 °C): δ = 9.98 (s, 2H), 7.13-7.22 (m, 8H), 2.64 (s, 4H), 2.34 ppm (s, 6H); ^{13}C NMR (101 MHz, DMSO-d_6, 25 °C): δ = 170.8, 139.9, 138.2, 132.7, 130.0, 128.8, 120.5, 116.6, 31.8, 21.7 ppm.

***N, N'*-Bis(4-methoxyphenyl)succinamide[15] (4k)**

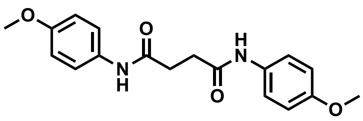

^{1}H NMR (400 MHz, DMSO-d_6, 25 °C): δ = 9.94 (s, 2H), 7.50-7.52 (m, 4H), 6.84-6.86 (m, 4H), 3.70 (s, 6H), 2.61 ppm (s, 4H); ^{13}C NMR (101 MHz, DMSO-d_6, 25 °C): δ = 170.3, 159.2, 125.0, 120.9, 115.3, 55.6, 31.7 ppm.

***N, N'*-Bis(4-fluorophenyl)succinamide[15] (4l)**

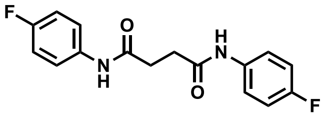

^{1}H NMR (400 MHz, DMSO-d_6, 25 °C): δ = 10.14 (s, 2H), 7.29-7.35 (m, 4H), 7.09-7.14 (m, 4H), 2.64 ppm (s, 4H); ^{13}C NMR (101 MHz, DMSO-d_6, 25 °C): δ = 172.9, 127.6 (J_{C-F} = 12 Hz), 123.2 (J_{C-F} = 10 Hz), 119.4, 119.2, 118.0, 117.7, 33.8 ppm; ^{19}F NMR (400 MHz, DMSO-d6, 25 °C): δ = -116.5 ppm.

***N, N'*-Bis(4-chlorophenyl)succinamide[15] (4m)**

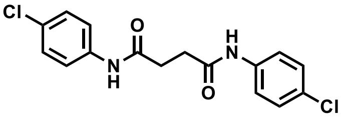

^{1}H NMR (400 MHz, DMSO-d_6, 25 °C): δ = 10.27 (s, 2H), 7.63-7.66 (m, 4H), 7.51-7.53 (m, 4H), 2.67 ppm (s, 4H); ^{13}C NMR (101 MHz, DMSO-d_6, 25 °C): δ = 171.0, 130.0, 129.0, 124.8, 120.9, 31.6 ppm.

***N, N'*-Bis(4-bromophenyl)succinamide[15] (4n)**

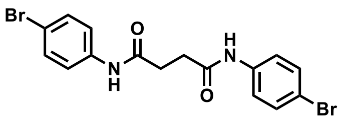

H NMR (400 MHz, DMSO-d_6, 25 °C): δ = 10.23 (s, 2H), 7.57-7.62 (m, 8H), 2.66 ppm (s, 4H); ^{13}C NMR (101 MHz, DMSO-d_6, 25 °C): δ = 171.0, 132.7, 131.9, 124.8, 121.3, 31.6 ppm.

***N, N'*-Bis(4-iodophenyl)succinamide (4o)**

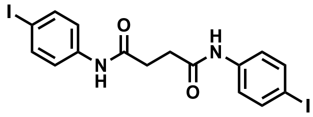

H NMR (400 MHz, DMSO-d_6, 25 °C): δ = 10.12 (s, 2H), 7.32-7.39 (m, 8H), 2.45 ppm (s, 4H); ^{13}C NMR (101 MHz, DMSO-d_6, 25 °C): δ = 171.0, 138.7, 134.7, 124.8, 121.6, 31.6 ppm.

***N, N'*-Bis(4-(methylthio)phenyl)succinamide (4r)**

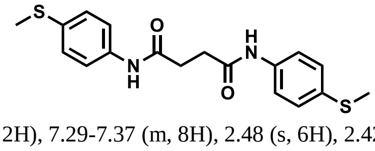

H NMR (400 MHz, DMSO-d_6, 25 °C): δ = 10.10 (s, 2H), 7.29-7.37 (m, 8H), 2.48 (s, 6H), 2.42 ppm (s, 4H); ^{13}C NMR (101 MHz, DMSO-d_6, 25 °C): δ = 171.8, 127.6, 127.5, 123.9, 120.1, 31.7, 15.3 ppm.

Piperidine-2,6-dione[5] (5a)

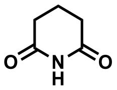

^{1}H NMR (400 MHz, DMSO-d_6, 25 °C): δ = 10.61 (s, 1H), 2.42-2.46 (m, 4H), 1.81-1.85 ppm (m, 2H); ^{13}C NMR (101 MHz, DMSO-d_6, 25 °C): δ = 174.2, 31.8, 18.0 ppm.

1-Methylpiperidine-2,6-dione[16] (5b)

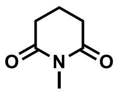

^{1}H NMR (400 MHz, DMSO-d_6, 25 °C): δ = 2.97 (s, 1H), 2.57-2.62 (m, 4H), 1.79-1.86 ppm (m, 2H); ^{13}C NMR (101 MHz, DMSO-d_6, 25 °C): δ = 173.3, 32.5, 26.1, 17.0 ppm.

1-Butylpiperidine-2,6-dione[17] (5c)

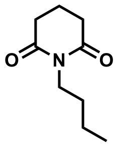

^{1}H NMR (400 MHz, DMSO-d_6, 25 °C): δ = 3.61 (t, J = 9.6 Hz, 2H), 2.59 (t, J = 8.8 Hz, 4H), 1.77-1.88 (m, 2H), 1.33-1.43 (m, 2H), 1.26-1.29 (m, 2H), 0.87 ppm (t, J = 8.8 Hz, 3H); ^{13}C NMR (101 MHz, DMSO-d_6, 25 °C): δ = 173.0, 38.7, 32.7, 30.2, 20.1, 17.1, 14.1 ppm.

1-Benzylpiperidine-2,6-dione[17] (5d)

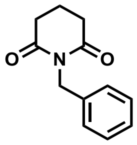

^{1}H NMR (400 MHz, DMSO-d_6, 25 °C): δ = 7.21-7.33 (m, 5H), 4.87 (s, 2H), 2.65-2.69 (m, 4H), 1.79-1.90 ppm (m, 2H); ^{13}C NMR (101 MHz, DMSO-d_6, 25 °C): δ = 173.2, 138.1, 128.7, 127.7, 127.3, 42.3, 32.6, 17.1 ppm.

1-Phenylpiperidine-2,6-dione[18] (5e)

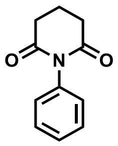

^{1}H NMR (400 MHz, DMSO-d_6, 25 °C): δ = 7.34-7.45 (m, 3H), 7.11-7.13 (m, 2H), 2.73 (t, J = 6.4 Hz, 4H), 1.96-2.03 ppm (m, 2H); ^{13}C NMR (101 MHz, DMSO-d_6, 25 °C): δ = 173.3, 136.6, 129.3, 129.1, 128.2, 32.9, 17.1 ppm.

1-(*p*-Tolyl)piperidine-2,6-dione[18] (5f)

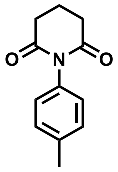

^{1}H NMR (400 MHz, DMSO-d_6, 25 °C): δ = 7.26-7.28 (m, 2H), 7.02-7.04 (m, 2H), 2.75-2.78 (m, 4H), 2.38 (s, 3H), 1.99-2.06 ppm (m, 2H); ^{13}C NMR (101 MHz, DMSO-d_6, 25 °C): δ = 173.3, 137.5, 133.9, 129.6, 129.0, 32.9, 21.1, 17.1 ppm.

1-(*m*-Tolyl)piperidine-2,6-dione[19] (5g)

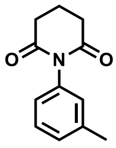

^{1}H NMR (400 MHz, DMSO-d_6, 25 °C): δ = 7.28-7.32 (m, 1H), 7.17-7.18 (m, 1H), 6.89-6.93 (m, 2H), 2.72 (t, J = 6.4 Hz, 4H), 2.32 (s, 3H), 1.95-2.01 ppm (m, 2H); ^{13}C NMR (101 MHz, DMSO-d_6, 25 °C): δ = 173.3, 138.5, 136.5, 129.7, 128.9, 128.8, 126.3, 32.9, 21.2, 17.1 ppm.

1-(4-Methoxyphenyl)piperidine-2,6-dione[18] (5h)

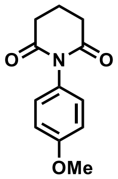

^{1}H NMR (400 MHz, DMSO-d_6, 25 °C): δ = 7.07-7.09 (m, 2H), 7.01-7.03 (m, 2H), 3.84 (s, 3H), 2.76 (t, J = 6.4 Hz, 4H), 2.00-2.05 ppm (m, 2H); ^{13}C NMR (101 MHz, DMSO-d_6, 25 °C): δ = 174.2, 159.0, 130.2, 129.0, 114.3, 55.7, 31.8, 18.0 ppm.

1-(4-Fluorophenyl)piperidine-2,6-dione[18] (5i)

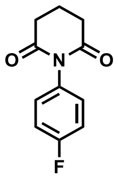

^{1}H NMR (400 MHz, DMSO-d_6, 25 °C): δ = 7.29-7.34 (m, 2H), 7.21-7.25 (m, 2H), 2.78 (t, J = 6.4 Hz, 4H), 2.02-2.08 ppm (m, 2H); ^{13}C NMR (101 MHz, DMSO-d_6, 25 °C): δ = 173.3, 132.6 ($J_{C\text{-}F}$ = 4 Hz), 131.4 ($J_{C\text{-}F}$ = 12 Hz), 116.1, 115.8, 32.2, 17.0 ppm; ^{19}F NMR (400 MHz, DMSO-d6, 25 °C): δ = -114.7 ppm.

1-(4-Chlorophenyl)piperidine-2,6-dione[18] (5j)

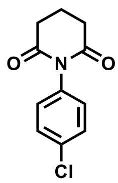

^{1}H NMR (400 MHz, DMSO-d_6, 25 °C): δ = 7.54-7.56 (m, 2H), 7.22-7.24 (m, 2H), 2.77 (t, J = 6.4 Hz, 4H), 2.01-2.08 ppm (m, 2H); ^{13}C NMR (101 MHz, DMSO-d_6, 25 °C): δ = 173.2, 135.5, 132.9, 131.3, 129.2, 32.8, 17.0 ppm.

1-(4-Bromophenyl)piperidine-2,6-dione[18] (5k)

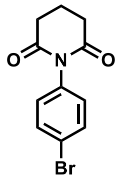

^{1}H NMR (400 MHz, DMSO-d_6, 25 °C): δ = 7.68-7.70 (m, 2H), 7.15-7.18 (m, 2H), 2.78 (t, J = 6.4 Hz, 4H), 2.00-2.09 ppm (m, 2H); ^{13}C NMR (101 MHz, DMSO-d_6, 25 °C): δ = 173.2, 138.0, 136.4, 131.8, 94.4, 32.8, 17.0 ppm.

1-(4-Iodophenyl)piperidine-2,6-dione (5l)

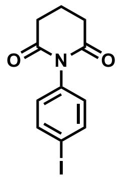

^{1}H NMR (400 MHz, DMSO-d_6, 25 °C): δ = 7.78-7.80 (m, 2H), 6.94-6.96 (m, 2H), 2.71 (t, J = 6.4 Hz, 4H), 1.94-2.03 ppm (m, 2H); ^{13}C NMR (101 MHz, DMSO-d_6, 25 °C): δ = 173.2, 138.0, 136.4, 131.8, 94.4, 32.8, 17.0 ppm.

1-(4-(Methylthio)phenyl)piperidine-2,6-dione (5o)

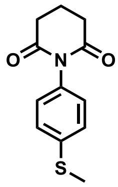

^{1}H NMR (400 MHz, DMSO-d_6, 25 °C): δ = 7.34-7.36 (m, 2H), 7.10-7.12 (m, 2H), 2.77 (t, J = 6.4 Hz, 4H), 2.00-2.06 ppm (m, 2H); ^{13}C NMR (101 MHz, DMSO-d_6, 25 °C): δ = 173.3, 138.3, 133.3, 129.8, 126.4, 94.4, 31.8, 17.9 ppm.

Glutaramide[20] (6a)

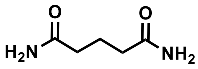

^{1}H NMR (400 MHz, DMSO-d_6, 25 °C): δ = 7.25 (s, 2H), 6.71 (s, 2H), 2.04 (t, J = 10.0 Hz, 4H), 1.63-1.73 ppm (m, 2H); ^{13}C NMR (101 MHz, DMSO-d_6, 25 °C): δ = 173.3, 34.7, 25.8, 21.1 ppm.

***N, N'*-Dimethylglutaramide[21] (6b)**

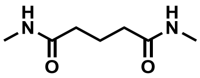

^{1}H NMR (400 MHz, DMSO-d_6, 25 °C): δ = 3.55 (s, 6H), 2.29 (q, J = 10.0 Hz, 4H), 1.13 ppm (t, J = 10.0 Hz, 2H); ^{13}C NMR (101 MHz, DMSO-d_6, 25 °C): δ = 173.3, 34.7, 25.8, 21.1 ppm.

***N, N'*-Dibutylglutaramide[21] (6c)**

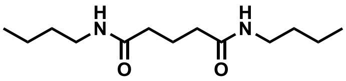

^{1}H NMR (400 MHz, DMSO-d_6, 25 °C): δ = 7.82 (s, 2H), 2.98-3.03 (m, 4H), 2.03 (q, J = 8.1 Hz, 4H), 1.68-1.71 (m, 2H), 1.50-1.58 (m, 8H), 0.88 ppm (t, J = 7.6 Hz, 6H); ^{13}C NMR (101 MHz, DMSO-d_6, 25 °C): δ = 172.0, 38.9, 35.3, 31.7, 20.0, 19.6, 14.0 ppm.

***N, N'*-Dibenzylglutaramide[21] (6d)**

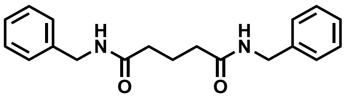

^{1}H NMR (400 MHz, DMSO-d_6, 25 °C): δ = 8.42 (s, 2H), 7.37-7.43 (m, 10H), 4.26 (d, J = 6.0 Hz, 4H), 2.17 (q, J = 7.6 Hz, 4H), 1.75-1.82 ppm (m, 2H); ^{13}C NMR (101 MHz, DMSO-d_6, 25 °C): δ = 172.2, 129.4, 129.0, 128.8, 128.7, 127.6, 127.1, 42.6, 35.3, 22.1 ppm.

***N, N'*-Diphenylglutaramide[15] (6e)**

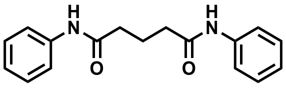

^{1}H NMR (400 MHz, DMSO-d_6, 25 °C): δ = 10.02 (s, 2H), 7.61-7.63 (m, 2H), 7.47-7.52 (m, 3H), 7.38-7.40 (m, 3H), 7.26-7.30 (m, 3H), 2.39 (q, J = 7.6 Hz, 4H), 1.87-1.94 ppm (m, 2H); ^{13}C NMR (101 MHz, DMSO-d_6, 25 °C): δ = 171.3, 132.7, 130.2, 129.1, 128.3, 123.6, 119.5, 36.1, 21.5 ppm.

***N*, *N'*-Di-*p*-tolylglutaramide[15] (6f)**

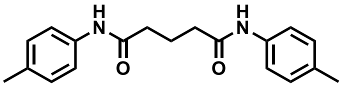

^{1}H NMR (400 MHz, DMSO-d_6, 25 °C): δ = 9.81 (s, 2H), 7.47-7.49 (m, 2H), 7.07-7.10 (m, 2H), 2.35 (t, J = 9.6 Hz, 4H), 2.24 (s, 6H), 1.42-1.51 ppm (m, 2H); ^{13}C NMR (101 MHz, DMSO-d_6, 25 °C): δ = 173.5, 137.2, 129.5, 119.5, 105.7, 35.6, 20.7, 19.0 ppm.

***N*, *N'*-Di-*m*-tolylglutaramide[22] (6g)**

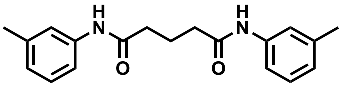

^{1}H NMR (400 MHz, DMSO-d_6, 25 °C): δ = 9.83 (s, 2H), 7.46 (s, 2H), 7.37-7.39 (m, 2H), 7.14-7.18 (m, 2H), 6.84-6.86 (m, 2H), 2.37 (t, J = 7.2 Hz, 4H), 2.27 (s, 6H), 1.89-1.94 ppm (m, 2H); ^{13}C NMR (101 MHz, DMSO-d_6, 25 °C): δ = 171.2, 139.7, 138.2, 128.9, 124.1, 120.1, 116.7, 36.8, 21.7, 21.5 ppm.

***N*, *N'*-Bis(4-methoxyphenyl)glutaramide[15] (6h)**

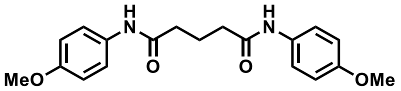

^{1}H NMR (400 MHz, DMSO-d_6, 25 °C): δ = 9.85 (s, 2H), 7.51-7.53 (m, 4H), 6.85-6.87 (m, 4H), 3.71 (s, 6H), 2.34 (t, J = 7.2 Hz, 4H), 1.85-1.92 ppm (m, 2H); ^{13}C NMR (101 MHz, DMSO-d_6, 25 °C): δ = 170.8, 124.9, 121.0, 115.3, 114.2, 55.9, 36.0, 21.7 ppm.

***N*, *N'*-Bis(4-fluorophenyl)glutaramide (6i)**

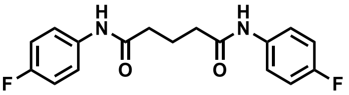

^{1}H NMR (400 MHz, DMSO-d_6, 25 °C): δ = 10.09 (s, 2H), 7.62-7.65 (m, 4H), 7.43-7.47 (m, 4H), 2.38 (t, J = 7.6 Hz, 4H), 1.87-1.94 ppm (m, 2H); ^{13}C NMR (101 MHz, DMSO-d_6, 25 °C): δ = 171.2, 136.2 ($J_{C\text{-}F}$ = 4.0 Hz), 129.0 ($J_{C\text{-}F}$ = 4.0 Hz), 125.6 ($J_{C\text{-}F}$ = 12.0 Hz), 121.2 ($J_{C\text{-}F}$ = 10.0 Hz), 121.2 ($J_{C\text{-}F}$ = 10.0 Hz), 117.2, 116.8, 115.8, 115.5, 35.9, 21.4 ppm; ^{19}F NMR (400 MHz, DMSO-d6, 25 °C): δ = -114.5 ppm..

***N*, *N'*-Bis(4-chlorophenyl)glutaramide[15] (6j)**

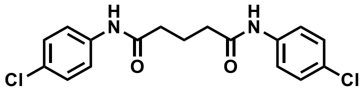

^{1}H NMR (400 MHz, DMSO-d_6, 25 °C): δ = 10.18 (s, 2H), 7.32-7.37 (m, 8H), 2.39 (t, J = 7.2 Hz, 4H), 1.86-1.94 ppm (m, 2H); ^{13}C NMR (101 MHz, DMSO-d_6, 25 °C): δ = 171.5, 130.0, 129.0, 124.7, 121.0, 36.0, 21.3 ppm.

***N*, *N'*-Bis(4-bromophenyl)glutaramide[15] (6k)**

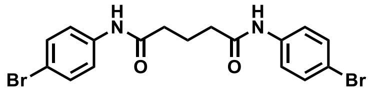

^{1}H NMR (400 MHz, DMSO-d_6, 25 °C): δ = 10.16 (s, 2H), 7.45-7.47 (m, 4H), 7.27-7.29 (m, 4H), 2.39 (t, J = 7.2 Hz, 4H), 1.86-1.93 ppm (m, 2H); ^{13}C NMR (101 MHz, DMSO-d_6, 25 °C): δ = 171.5, 132.9, 131.9, 124.6, 121.4, 36.0, 21.2 ppm.

***N*, *N'*-Bis(4-iodophenyl)glutaramide (6l)**

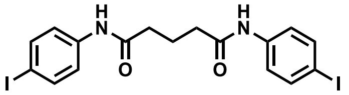

^{1}H NMR (400 MHz, DMSO-d_6, 25 °C): δ = 10.10 (s, 2H), 7.61-7.63 (m, 4H), 7.45-7.47 (m, 4H), 2.37 (t, J = 7.2 Hz, 4H), 1.87-1.92 ppm (m, 2H); ^{13}C NMR (101 MHz, DMSO-d_6, 25 °C): δ = 171.5, 138.7, 137.7, 124.3, 121.7, 36.0, 21.2 ppm.

***N*, *N'*-Bis(4-(methylthio)phenyl)glutaramide (6o)**

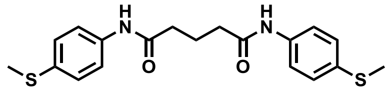

^{1}H NMR (400 MHz, DMSO-d_6, 25 °C): δ = 10.02 (s, 2H), 7.57-7.60 (m, 4H), 7.20-7.23 (m, 4H), 2.48 (s, 6H), 2.37 (t, J = 9.6 Hz, 4H), 1.84-1.94 ppm (m, 2H); ^{13}C NMR (101 MHz, DMSO-d_6, 25 °C): δ = 171.2, 127.6, 127.5, 123.9, 120.2, 36.0, 21.4, 15.3 ppm.

Azepane-2,7-dione[23] (7a)

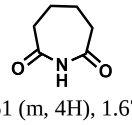

^{1}H NMR (400 MHz, DMSO-d_6, 25 °C): δ = 10.22 (s, 1H), 2.56-2.61 (m, 4H), 1.67-1.73 ppm (m, 4H); ^{13}C NMR (101 MHz, DMSO-d_6, 25 °C): δ = 176.0, 35.0, 26.7 ppm.

Adipamide[20] (8a)

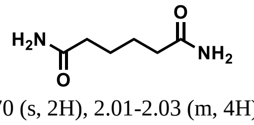

^{1}H NMR (400 MHz, DMSO-d_6, 25 °C): δ = 7.24 (s, 2H), 6.70 (s, 2H), 2.01-2.03 (m, 4H), 1.44-1.46 ppm (m, 4H); ^{13}C NMR (101 MHz, DMSO-d_6, 25 °C): δ = 174.7, 35.4, 25.3 ppm.

3. ^{1}H and ^{13}C NMR Spectra of products

Ethyl 4-oxo-4-(phenylamino)butanoate (EOPB)

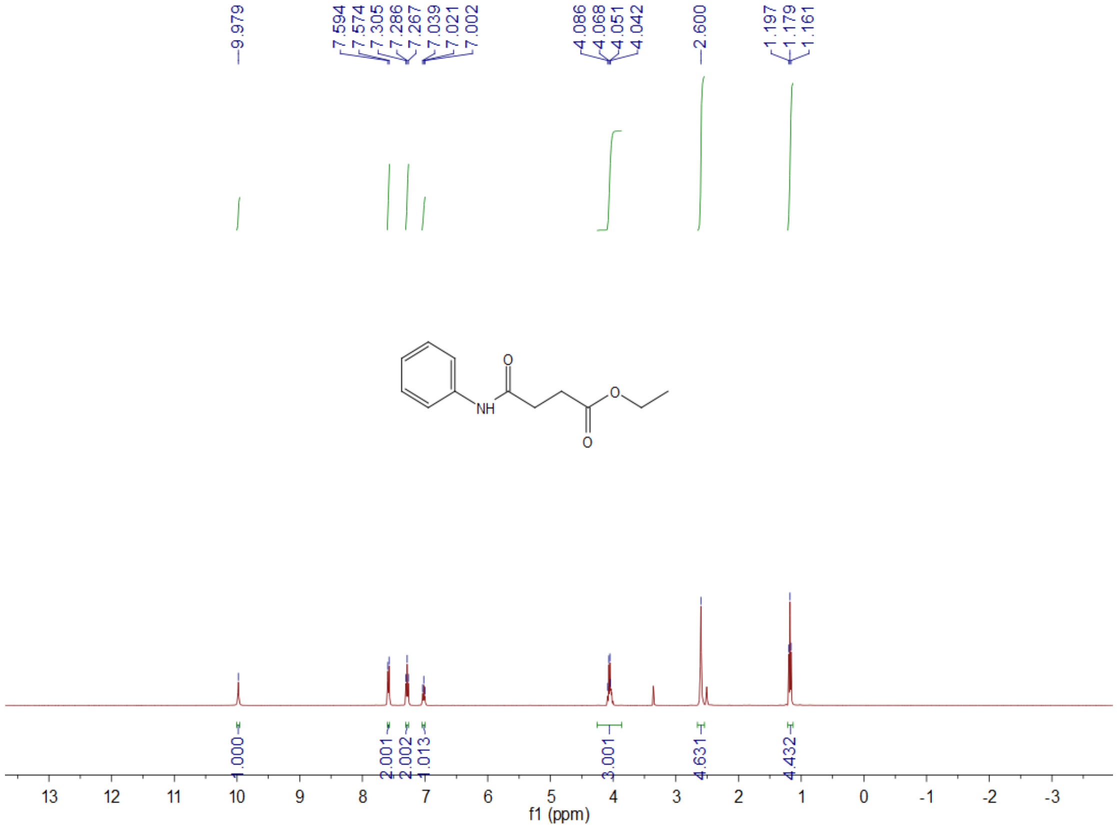

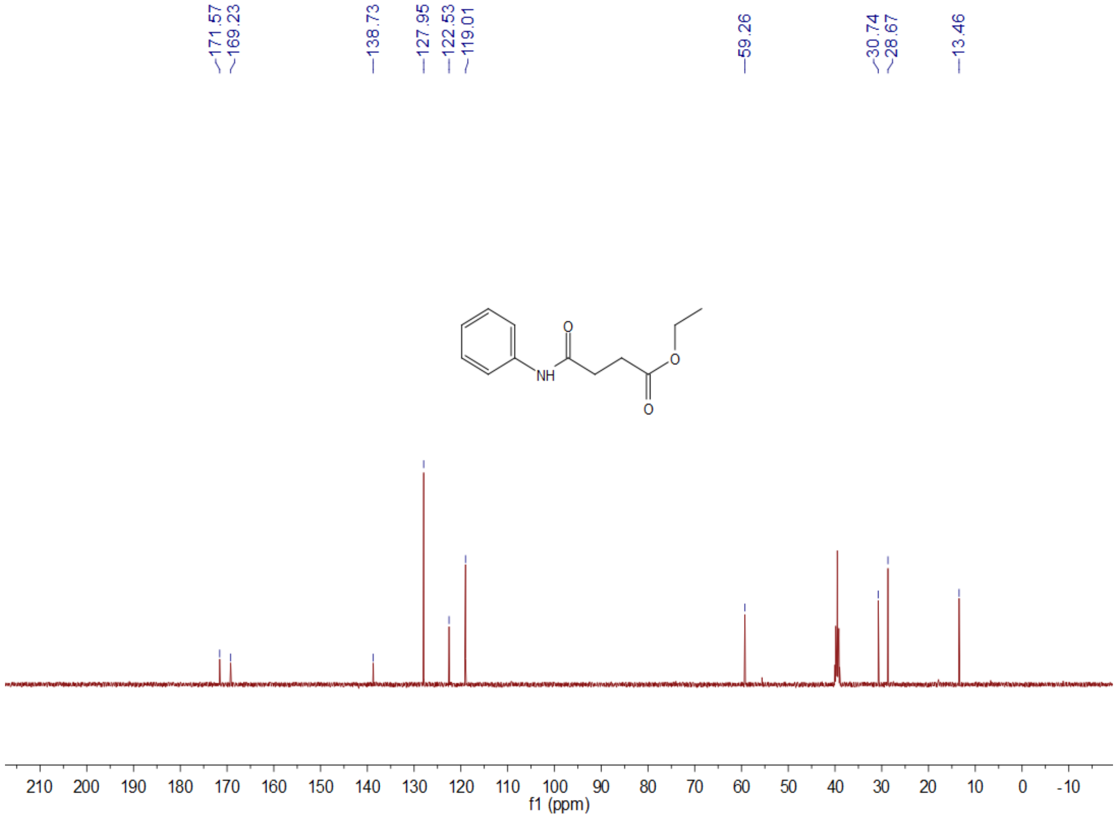

Tetrabutylphosphonium succinimide [P_{4444}][Suc]

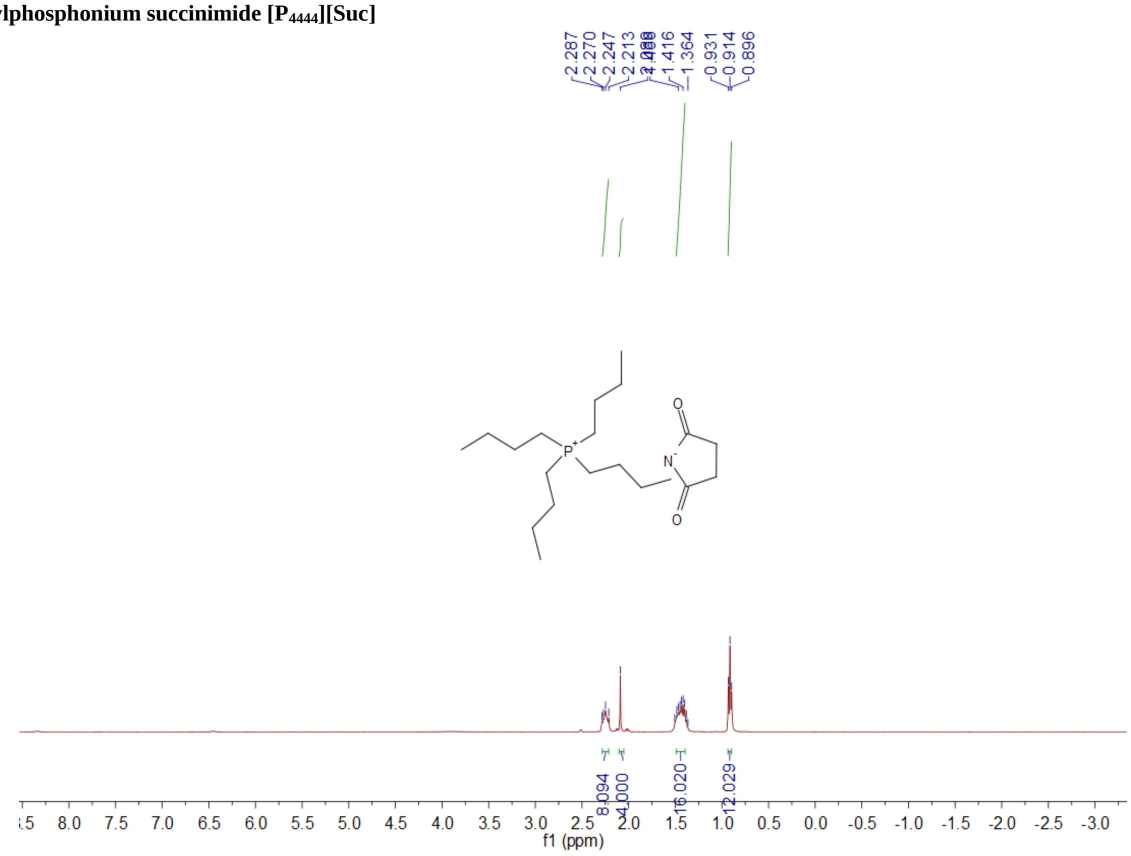

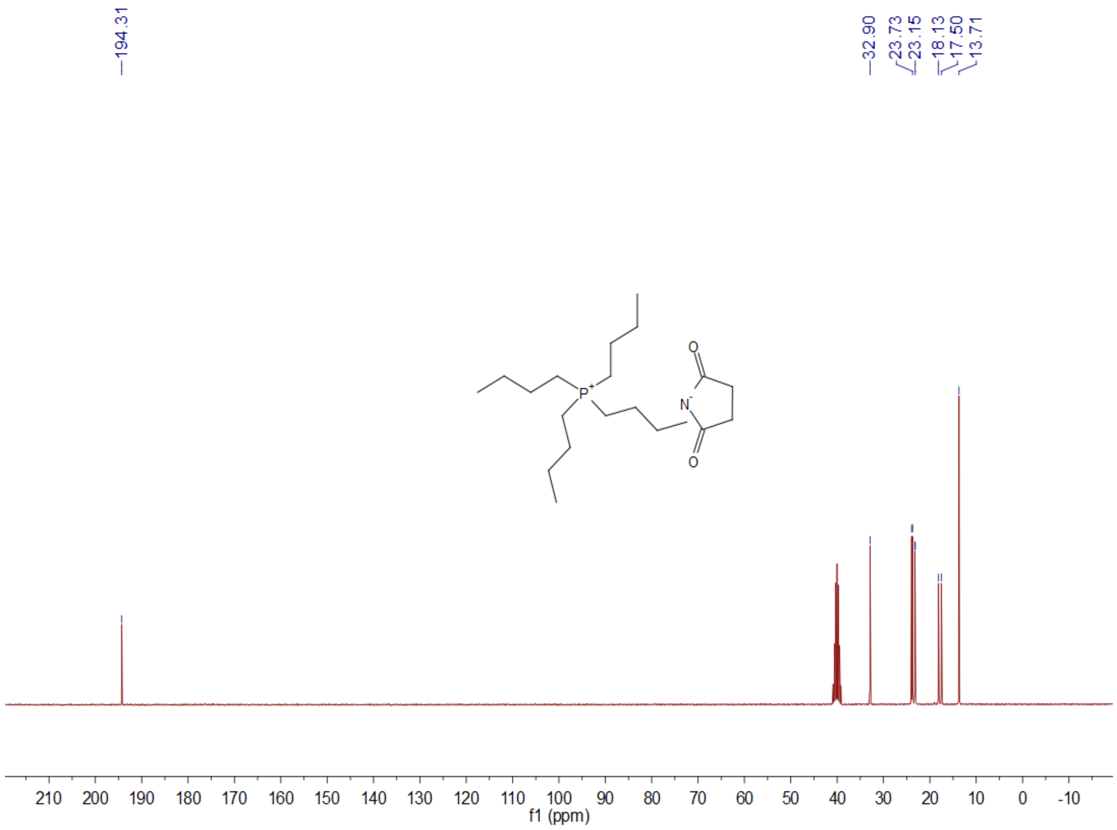

Tetrabutylammonium succinimide [N_{4444}][Suc]

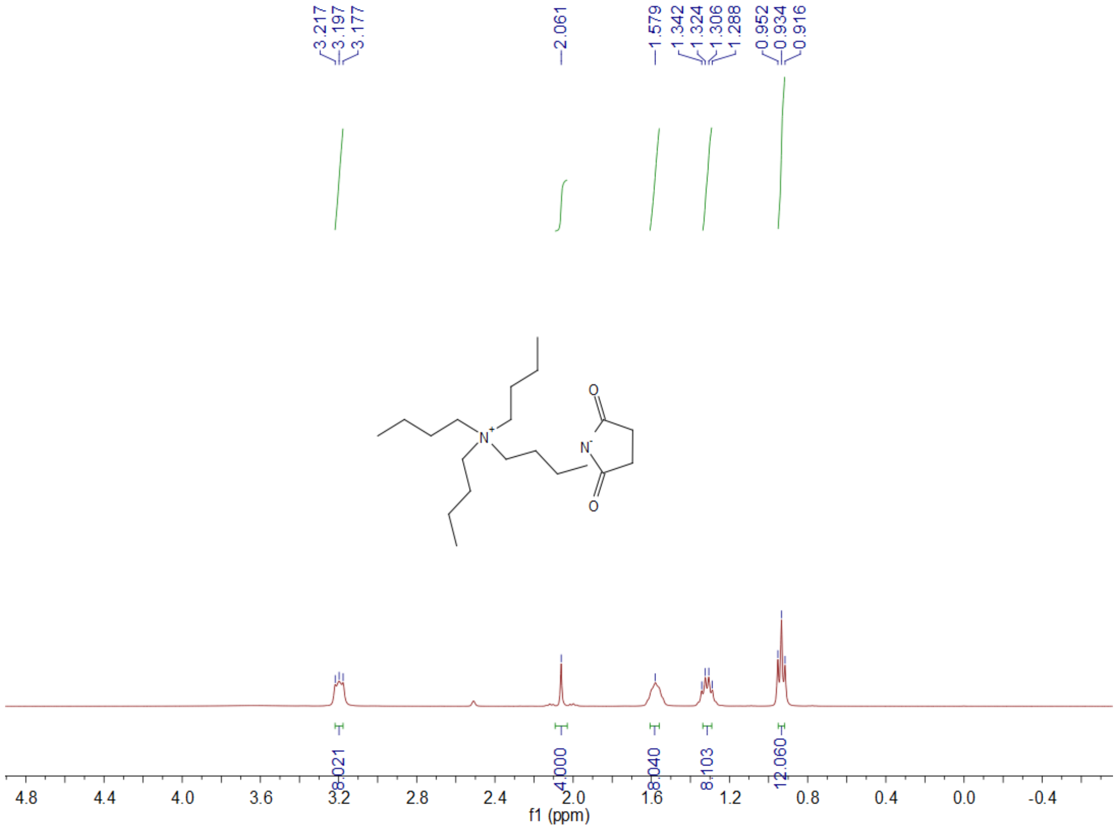

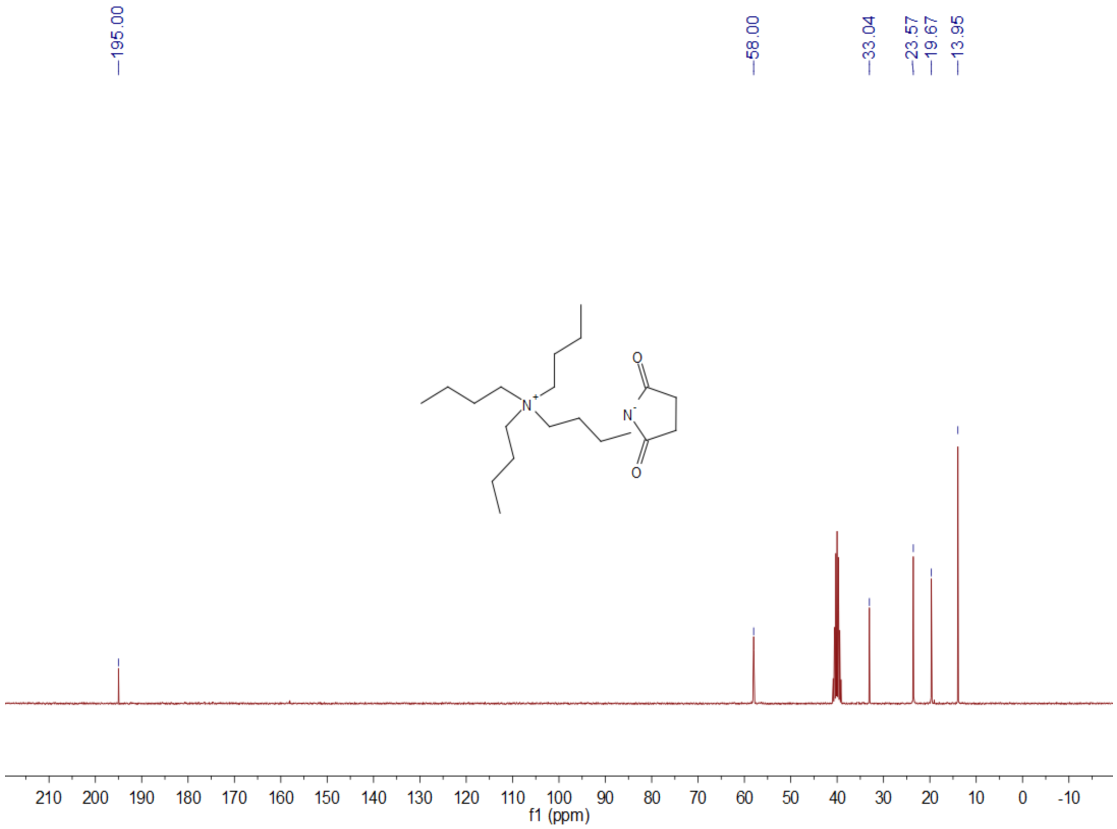

Choline succinimide [Ch][Suc]

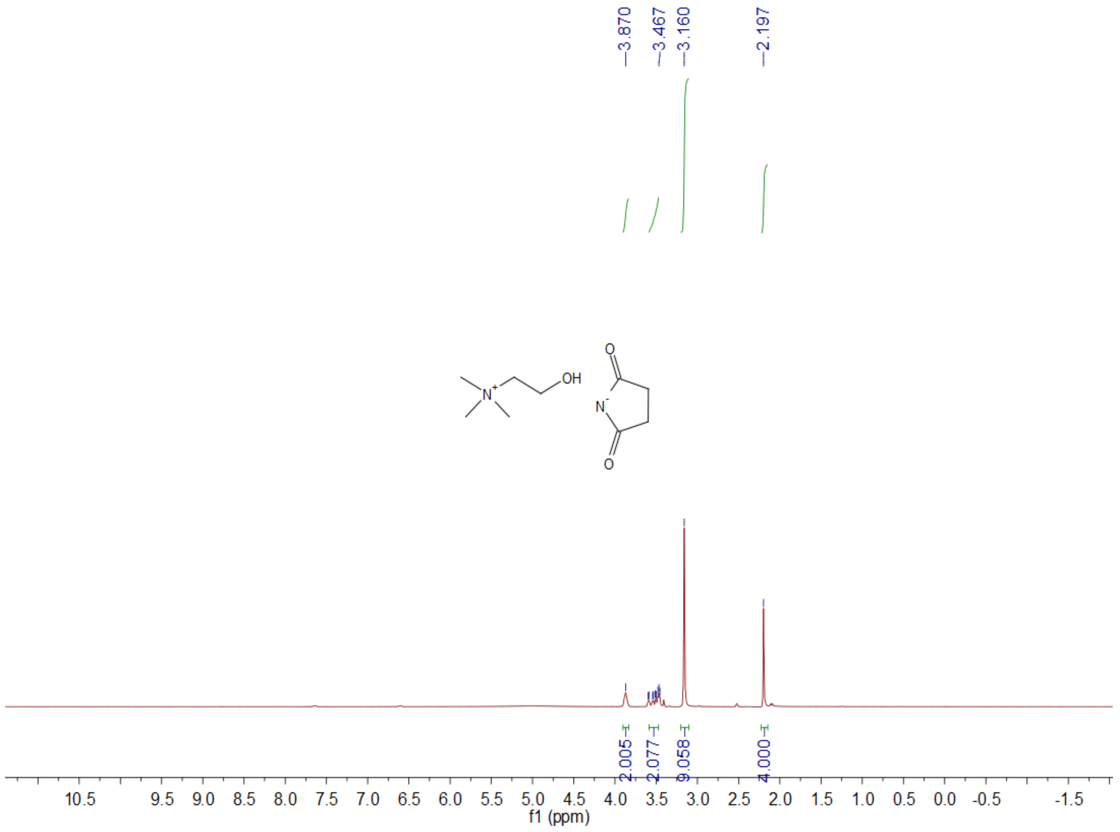

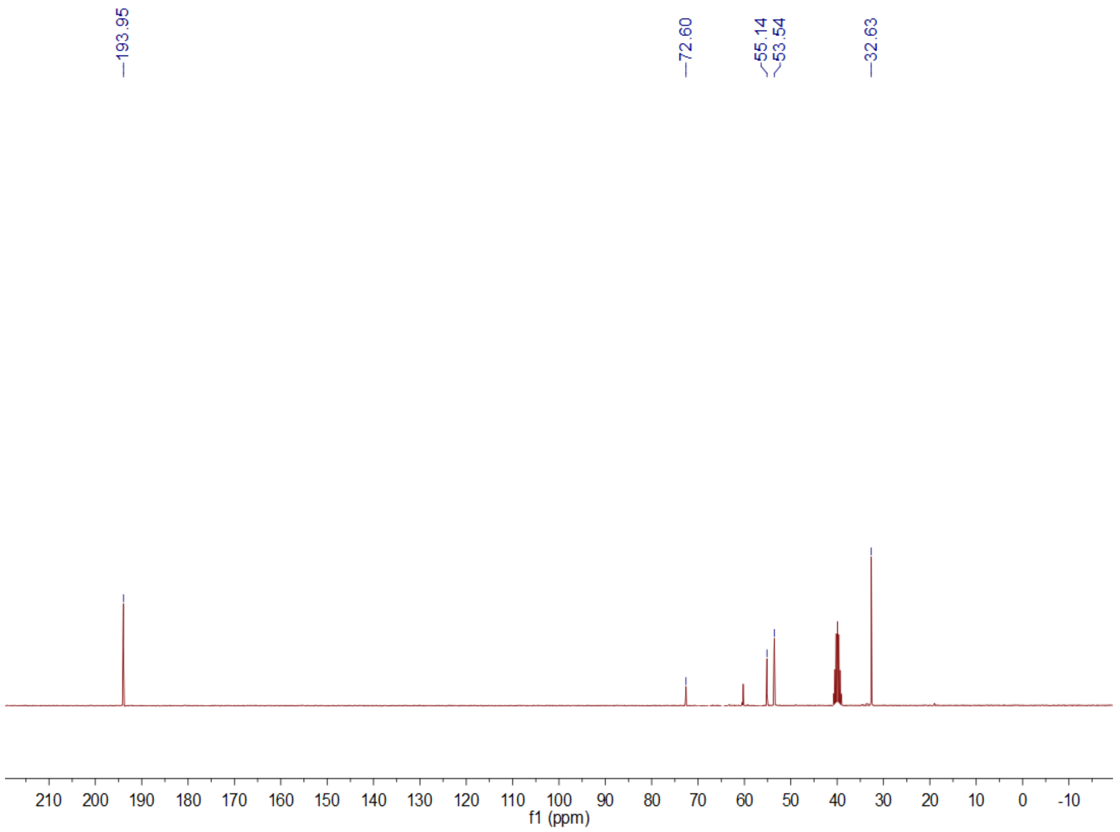

8-Diazabicyclo[5.4.0]undec-7-ene succinimide [HDBU][Suc]

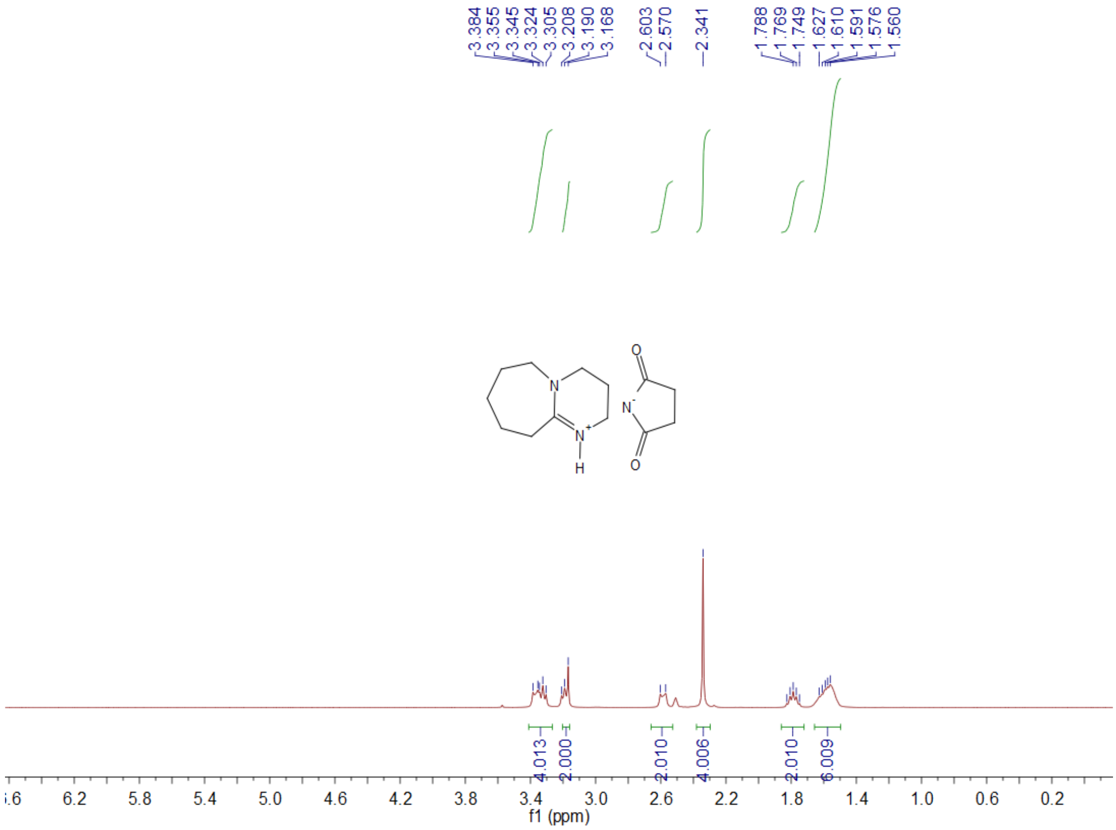

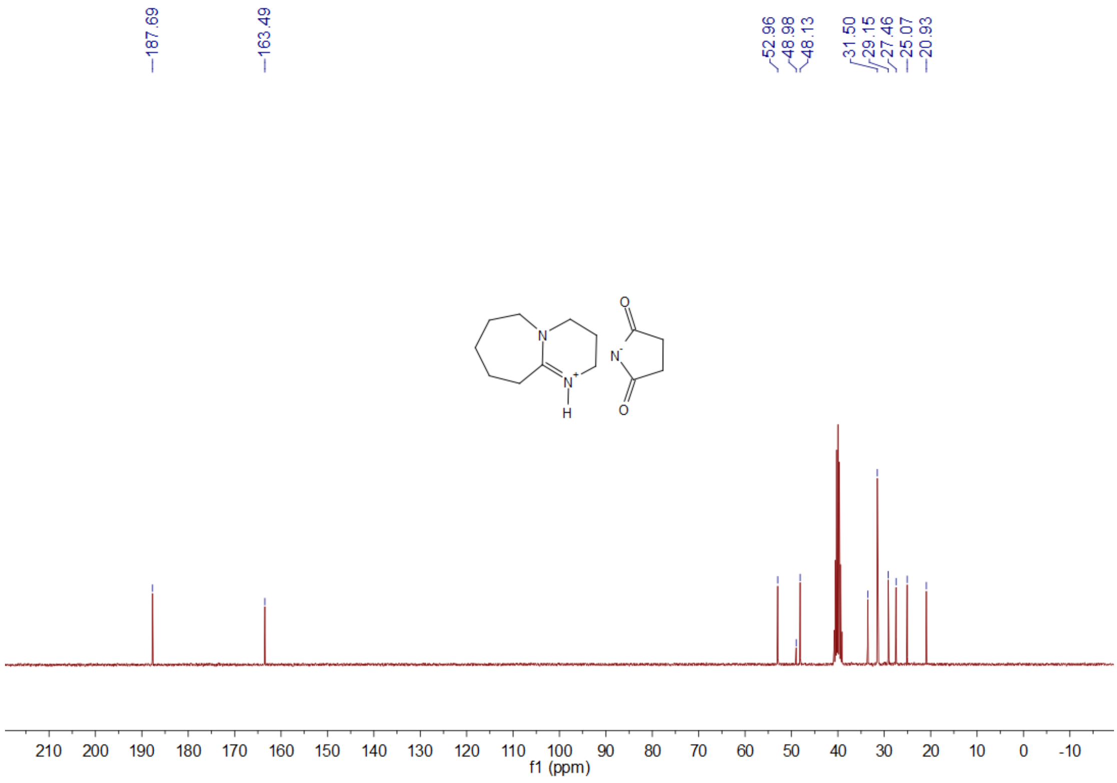

1-Butylpyrrolidine-2,5-dione (3a)

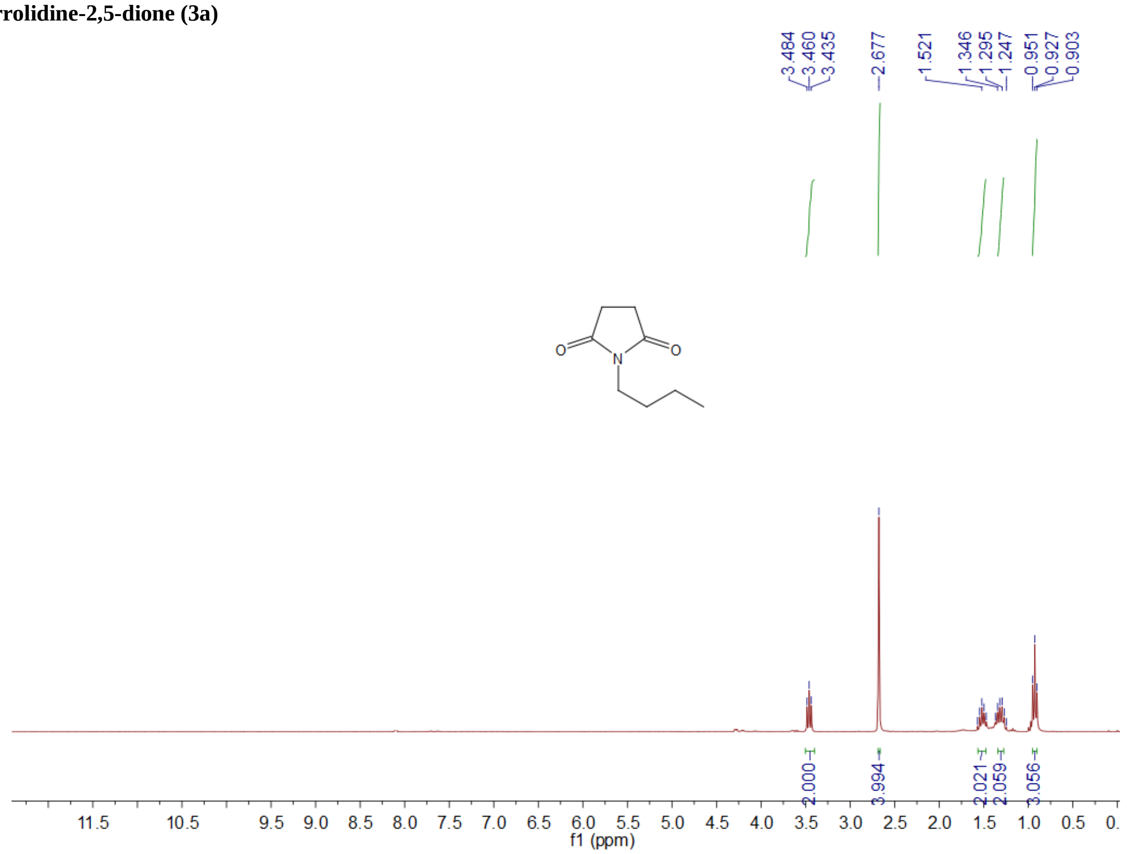

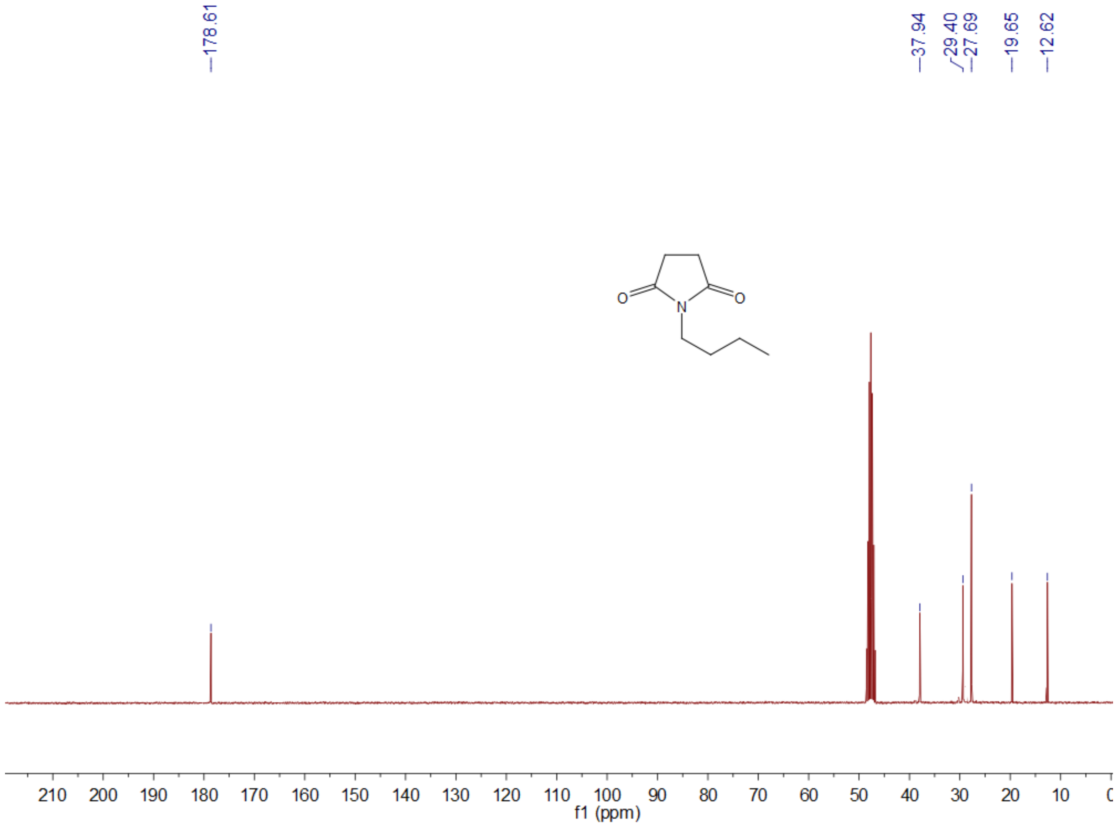

Pyrrolidine-2,5-dione (3b)

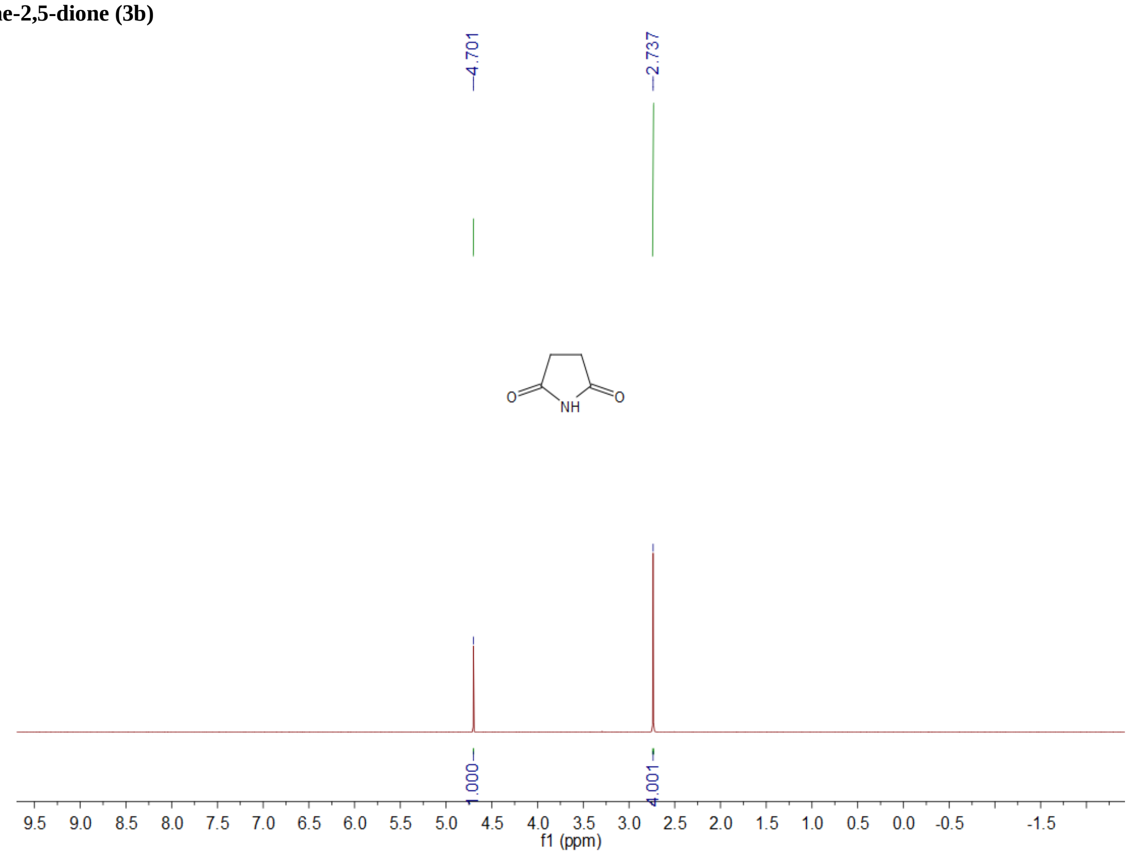

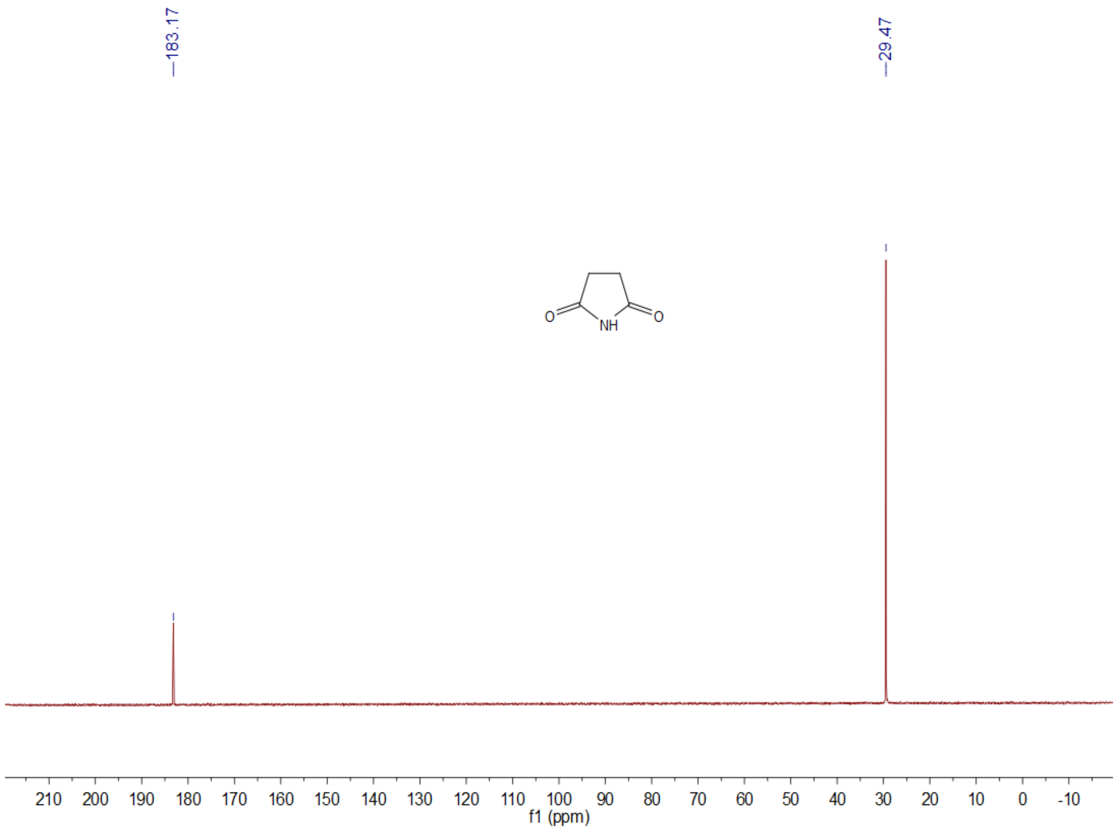

1-Methylpyrrolidine-2,5-dione (3c)

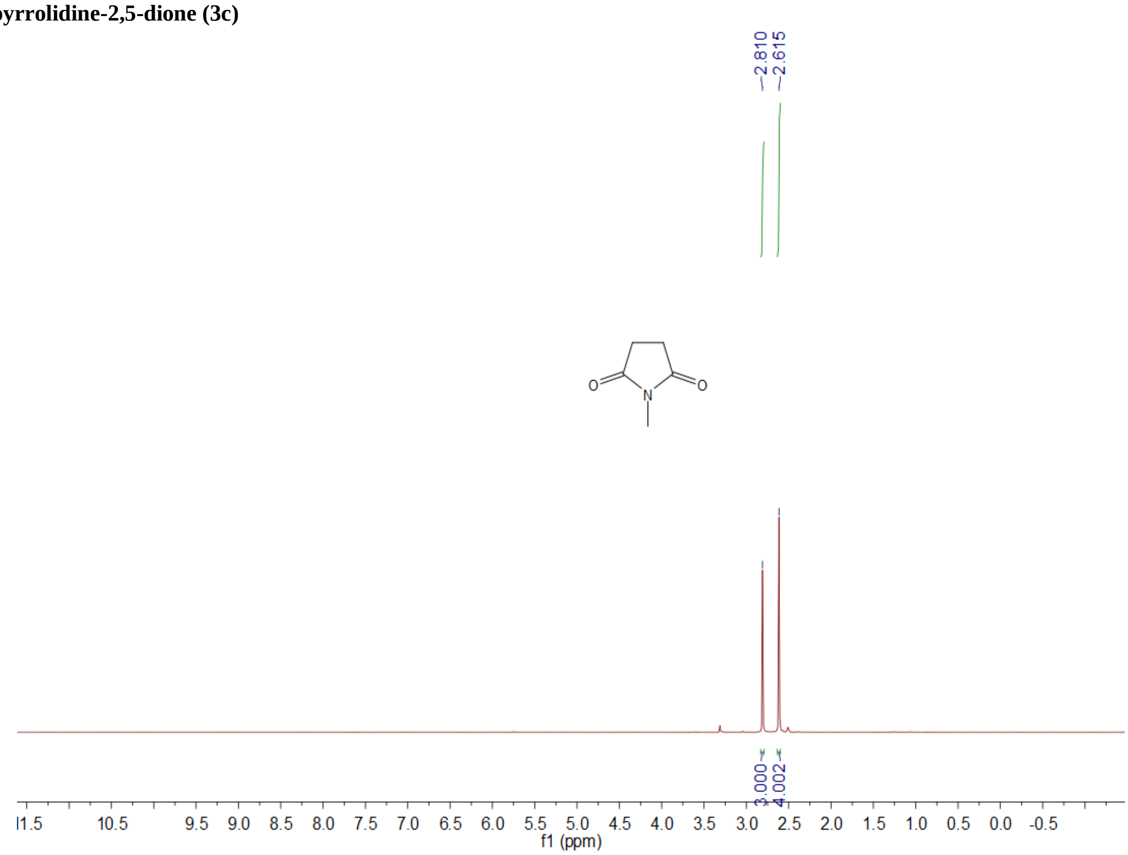

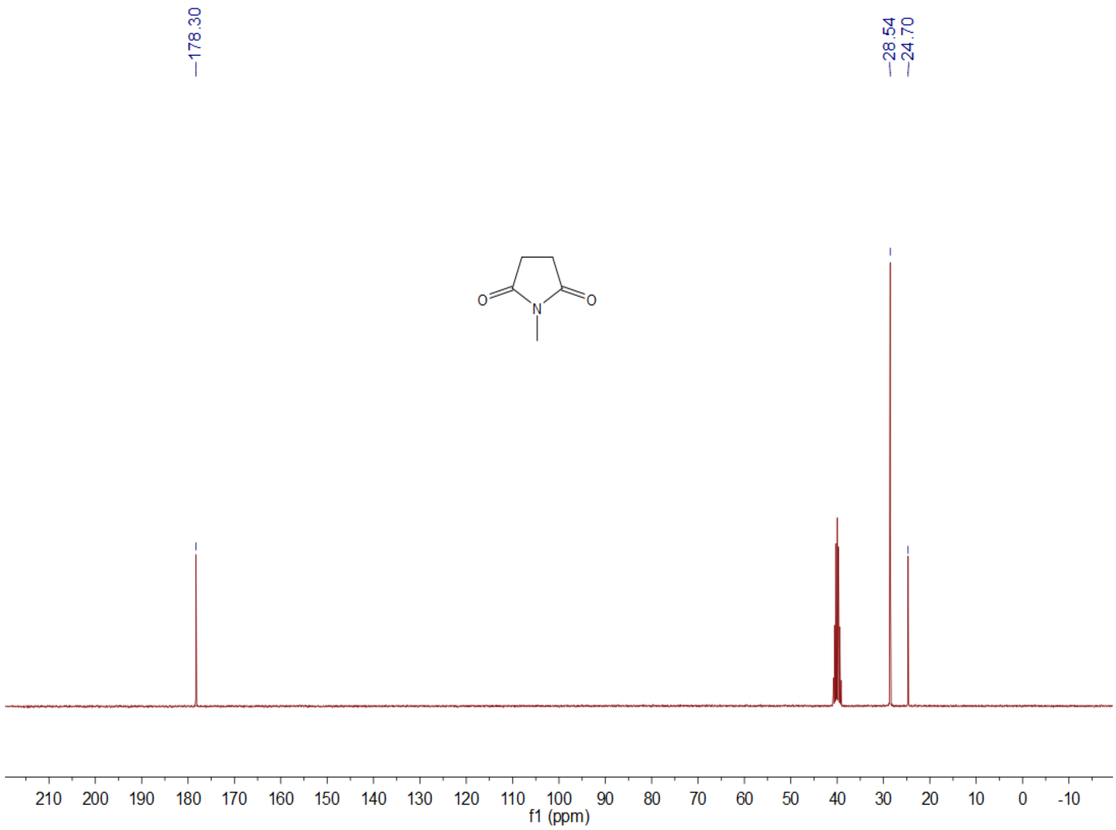

1-Ethylpyrrolidine-2,5-dione (3d)

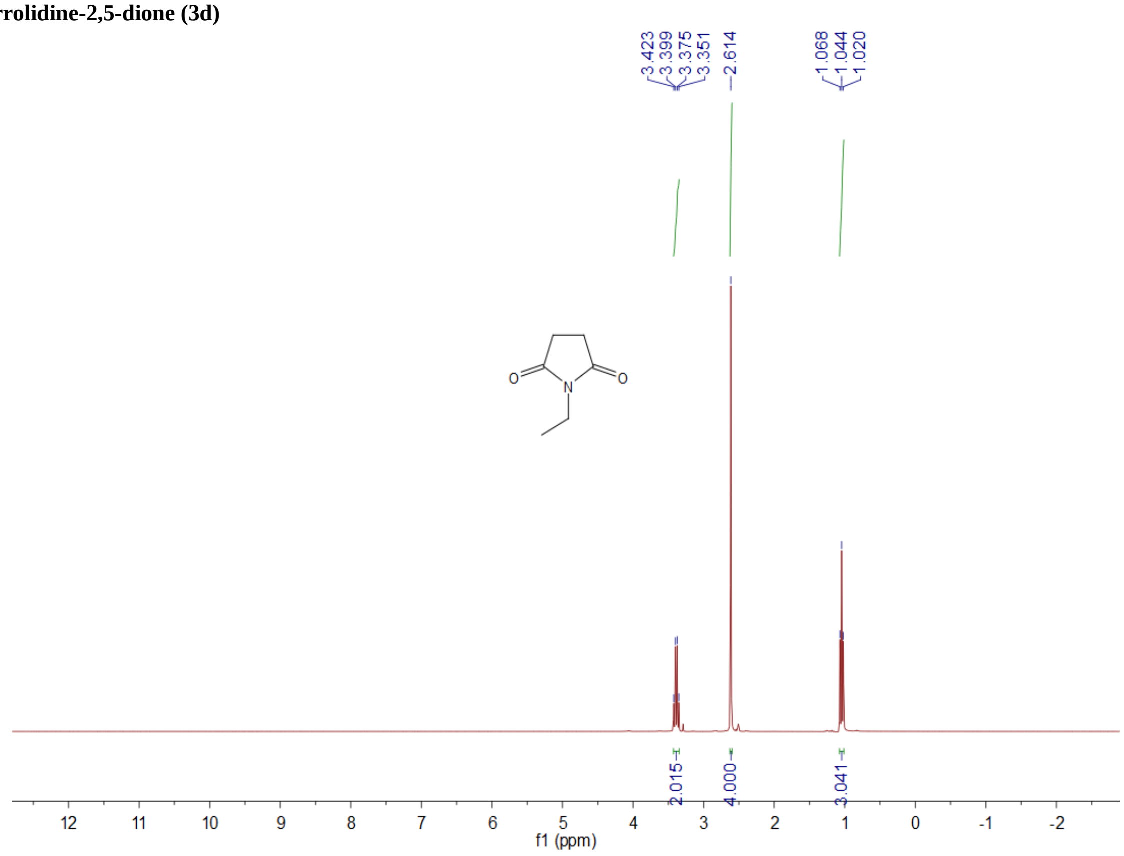

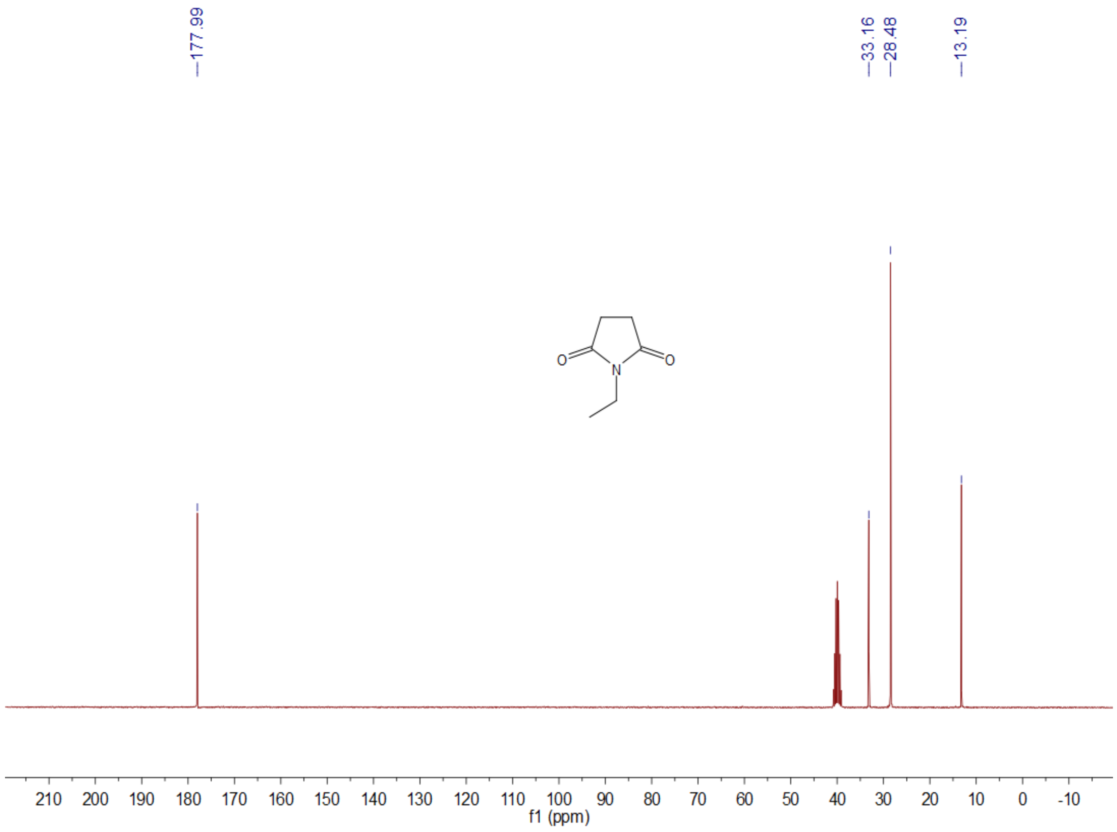

1-Isopropylpyrrolidine-2,5-dione (3e)

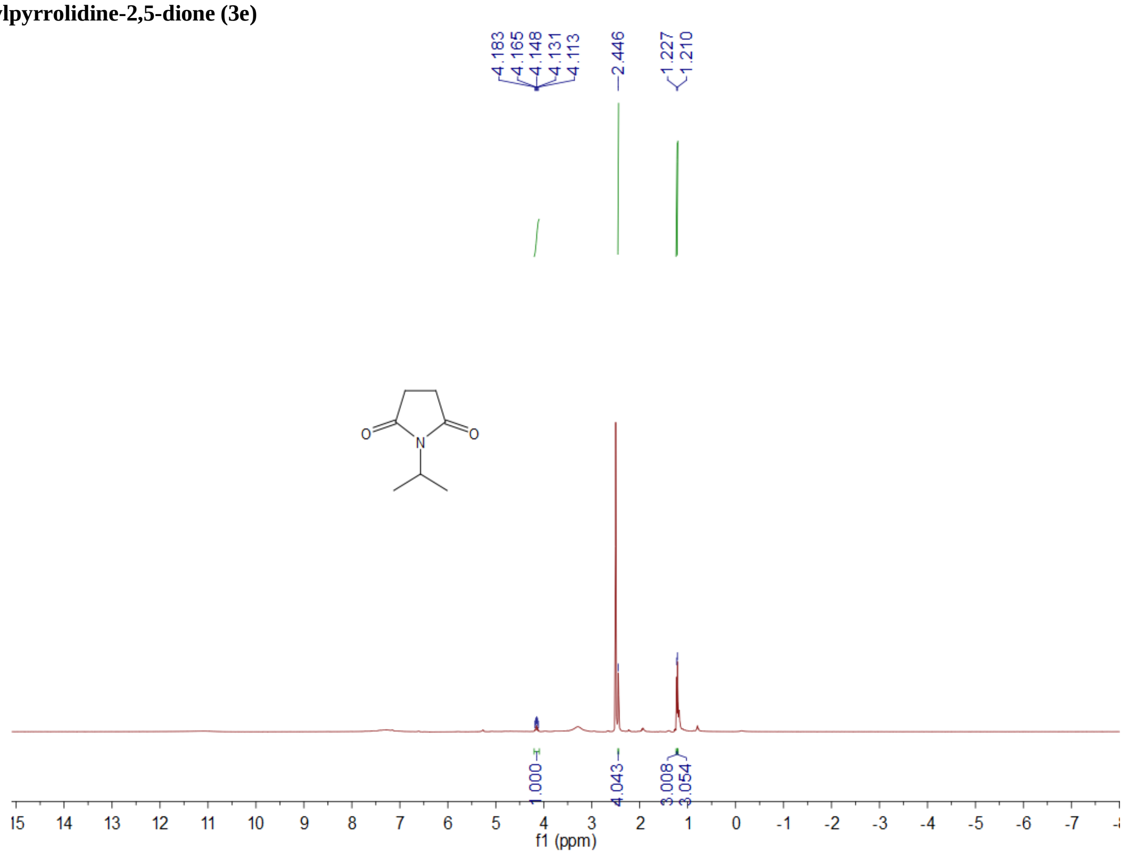

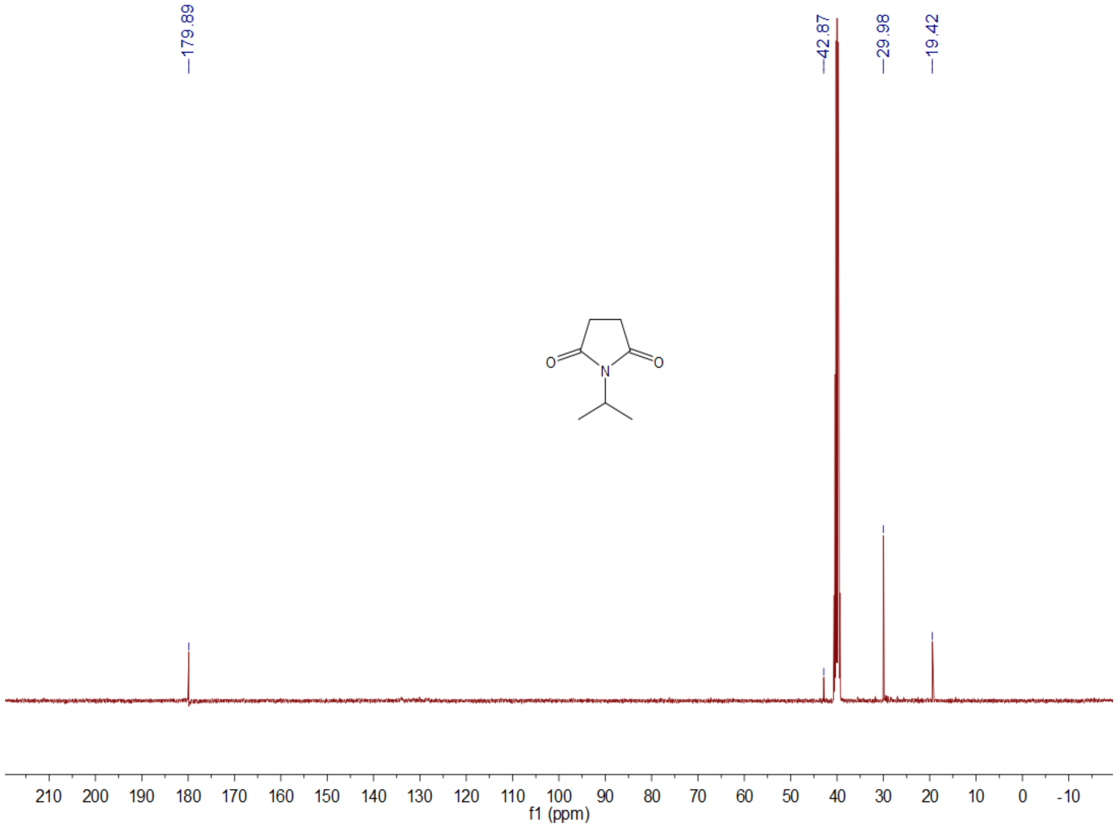

1-Hexylpyrrolidine-2,5-dione (3f)

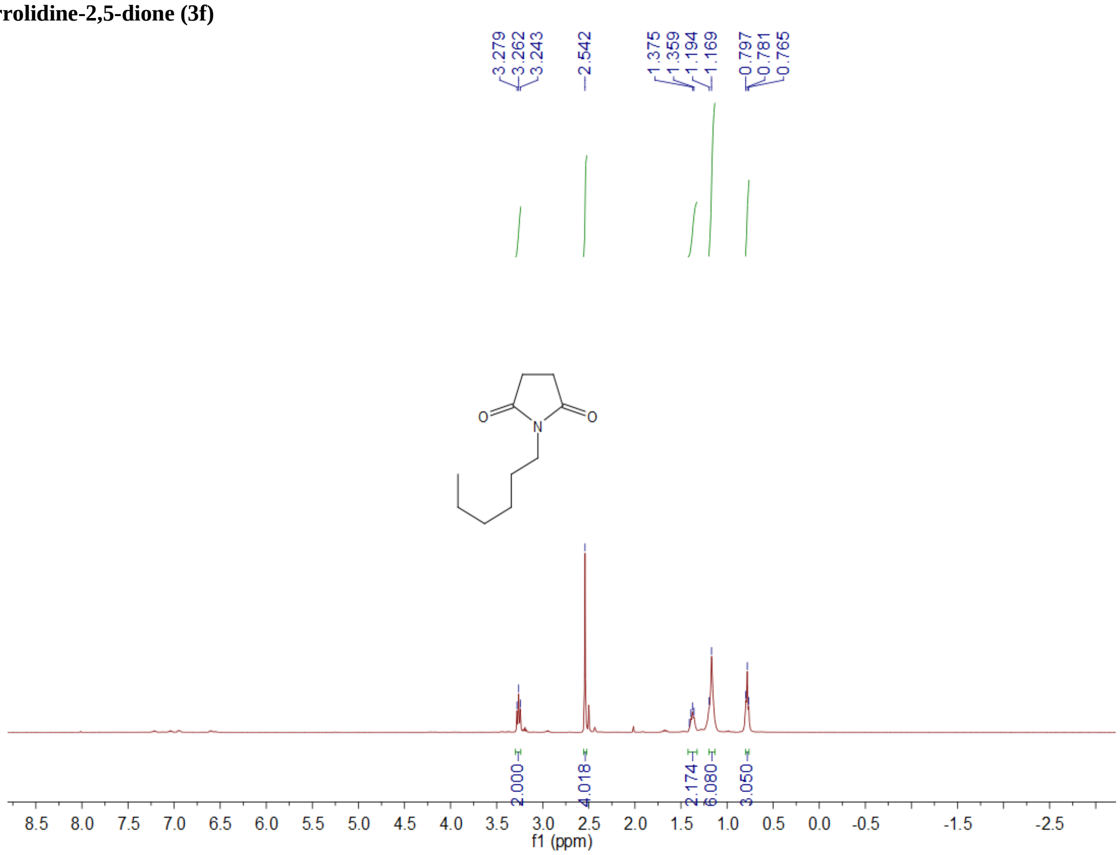

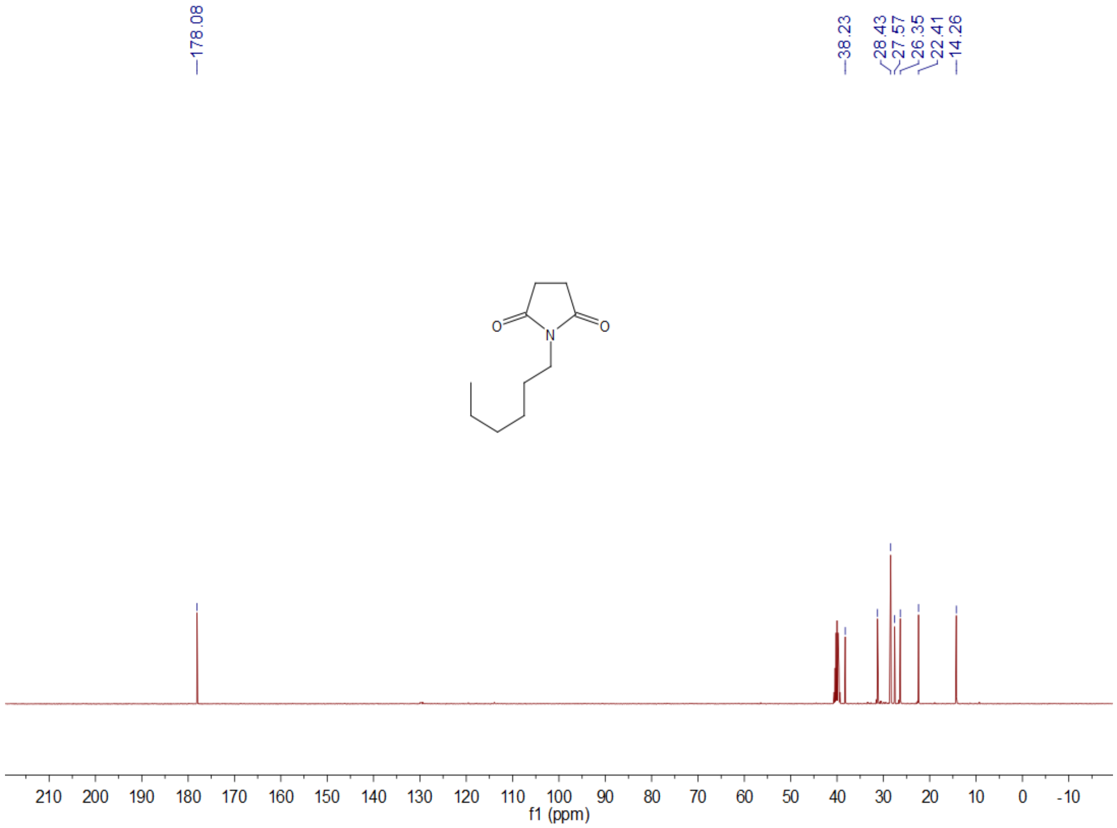

1-Benzylpyrrolidine-2,5-dione (3g)

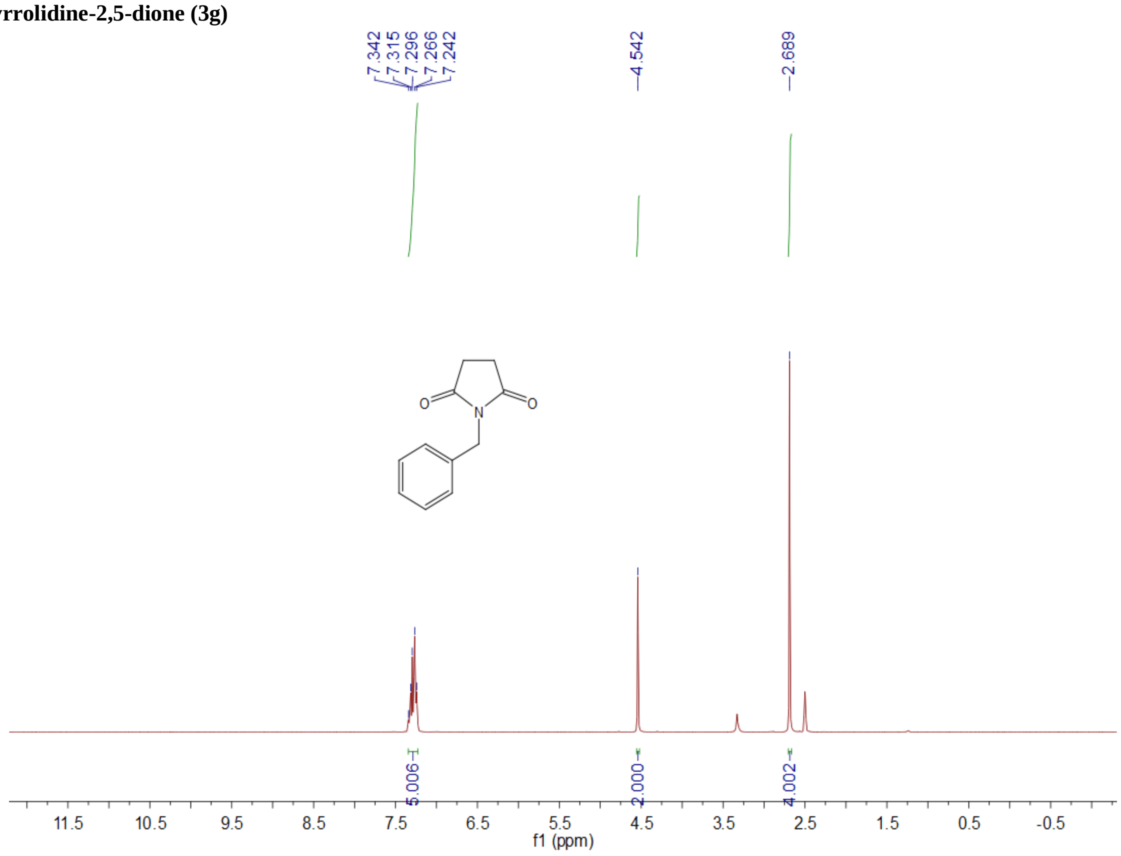

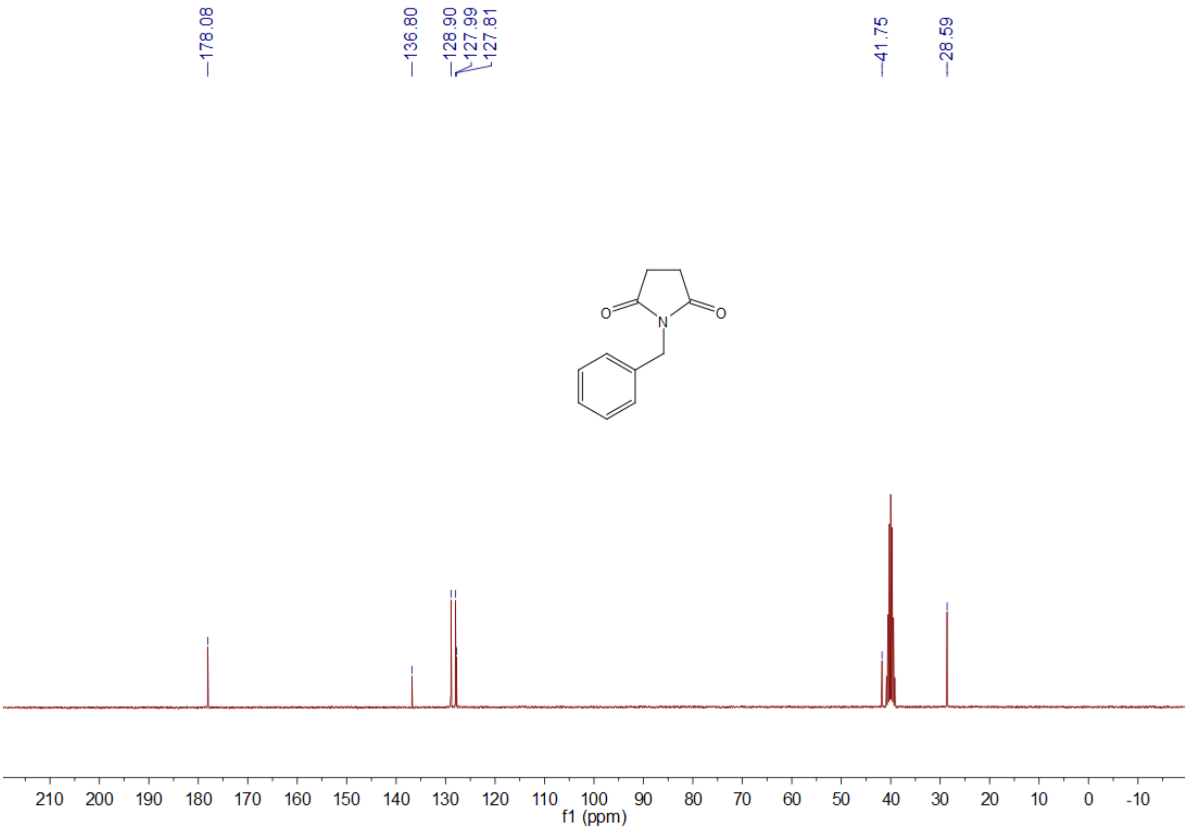

1-Phenylpyrrolidine-2,5-dione (3h)

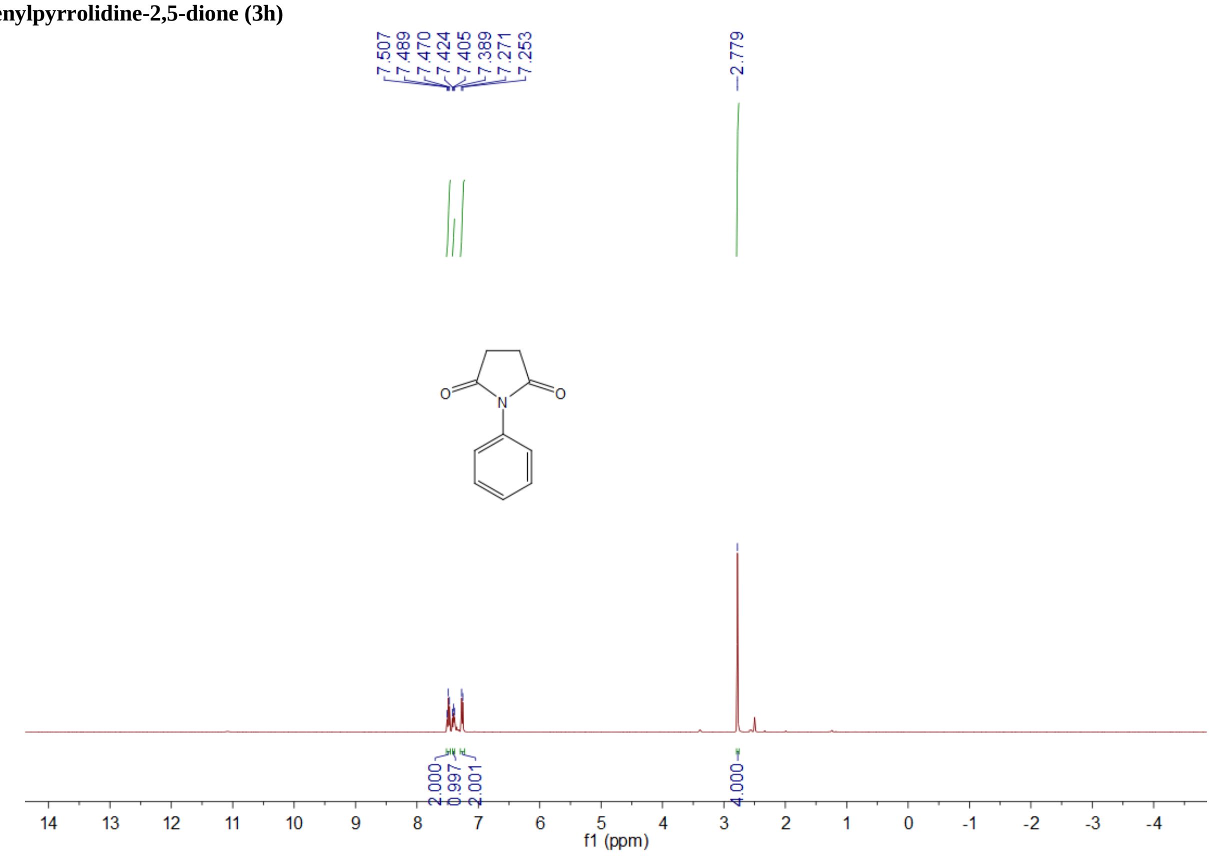

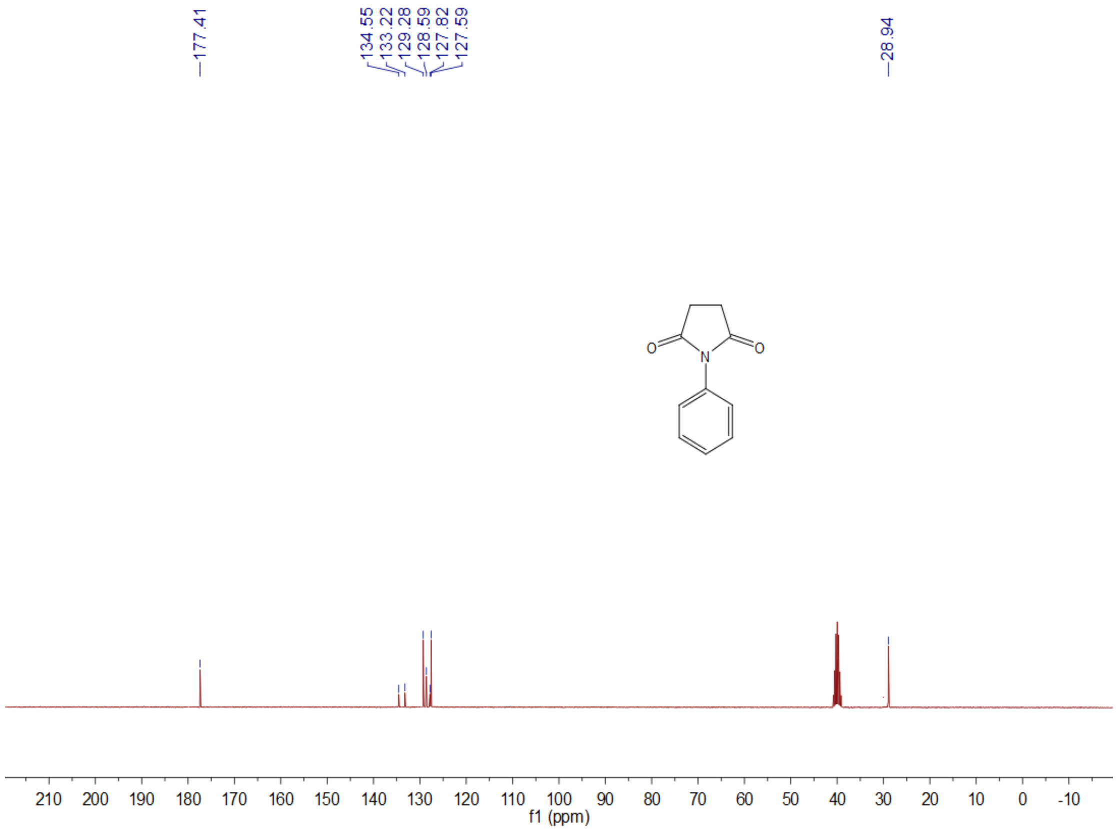

1-(*p*-Tolyl)pyrrolidine-2,5-dione (3i)

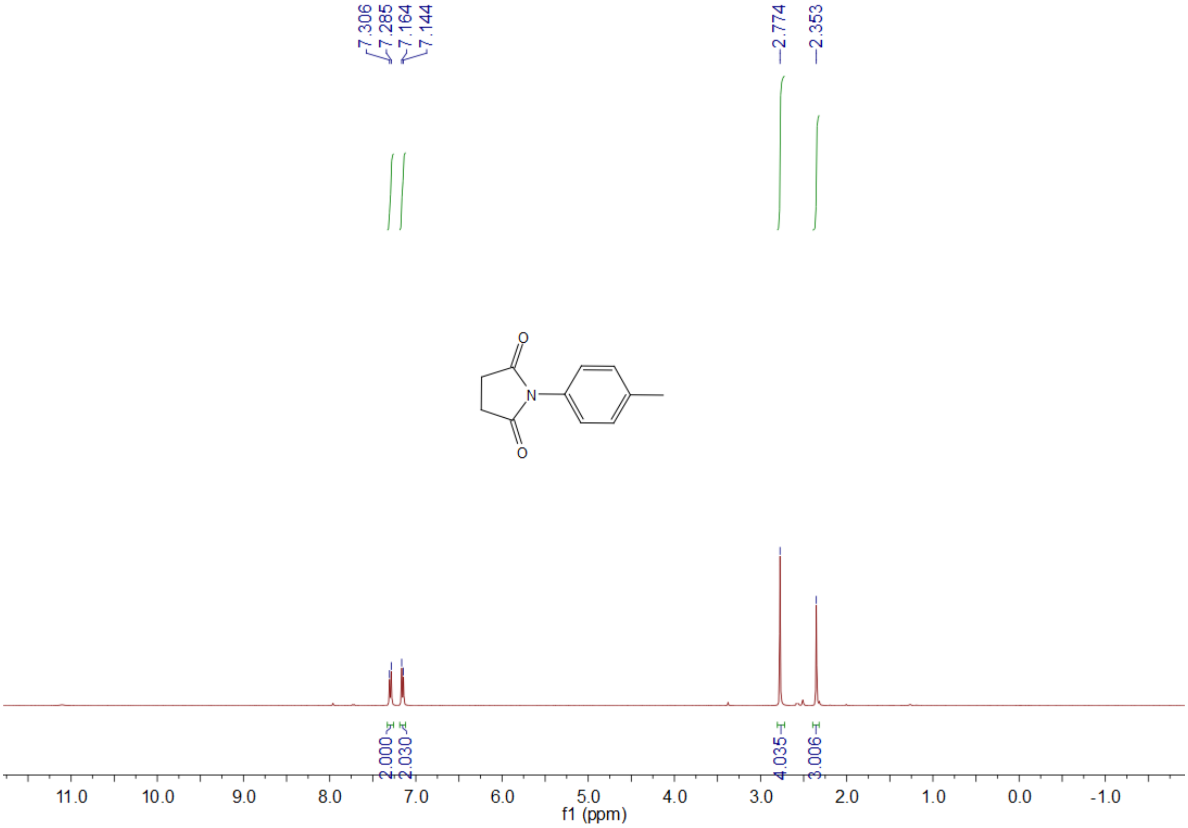

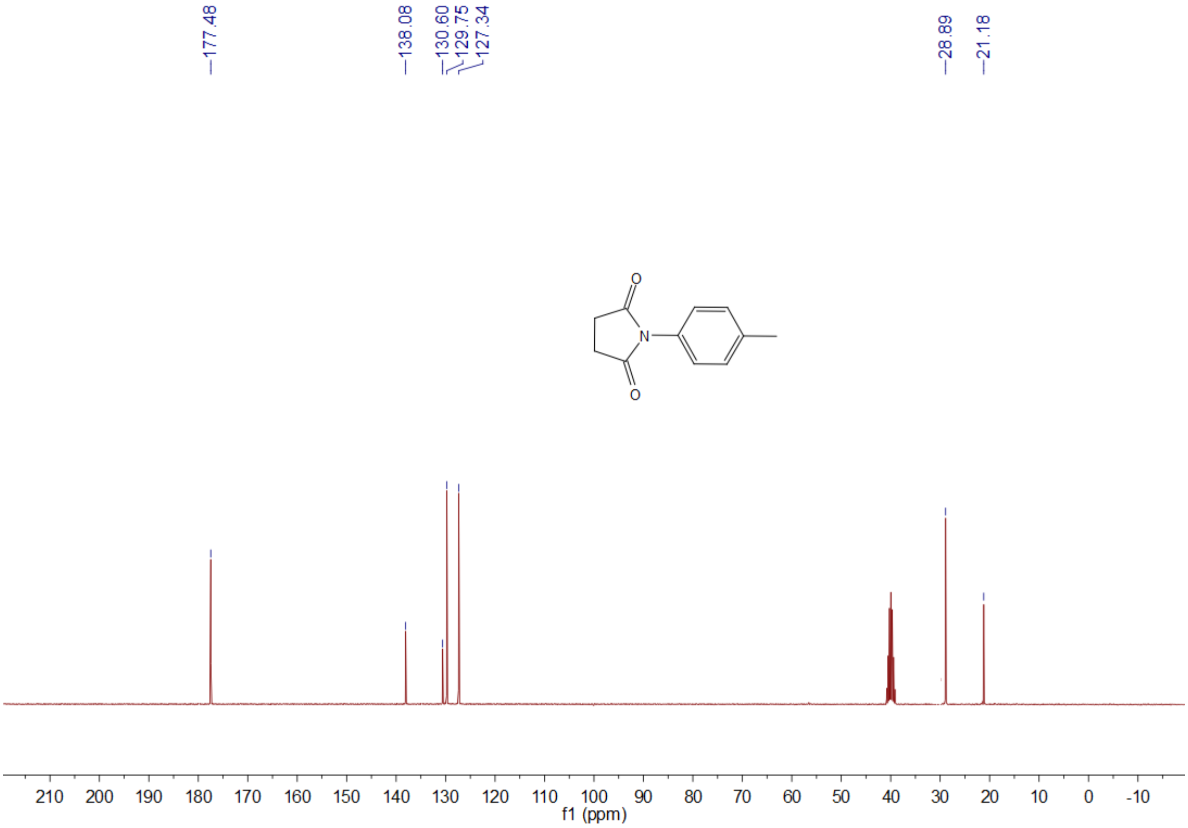

1-(*m*-Tolyl)pyrrolidine-2,5-dione (3j)

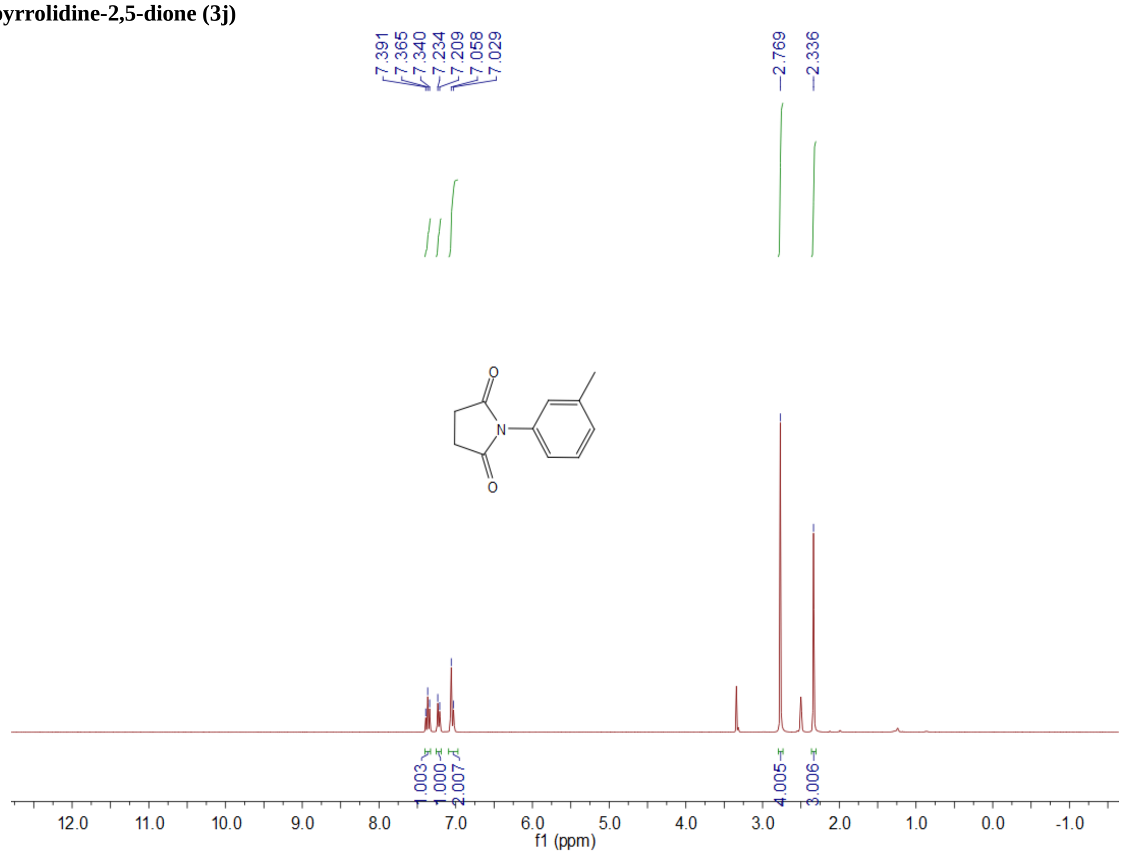

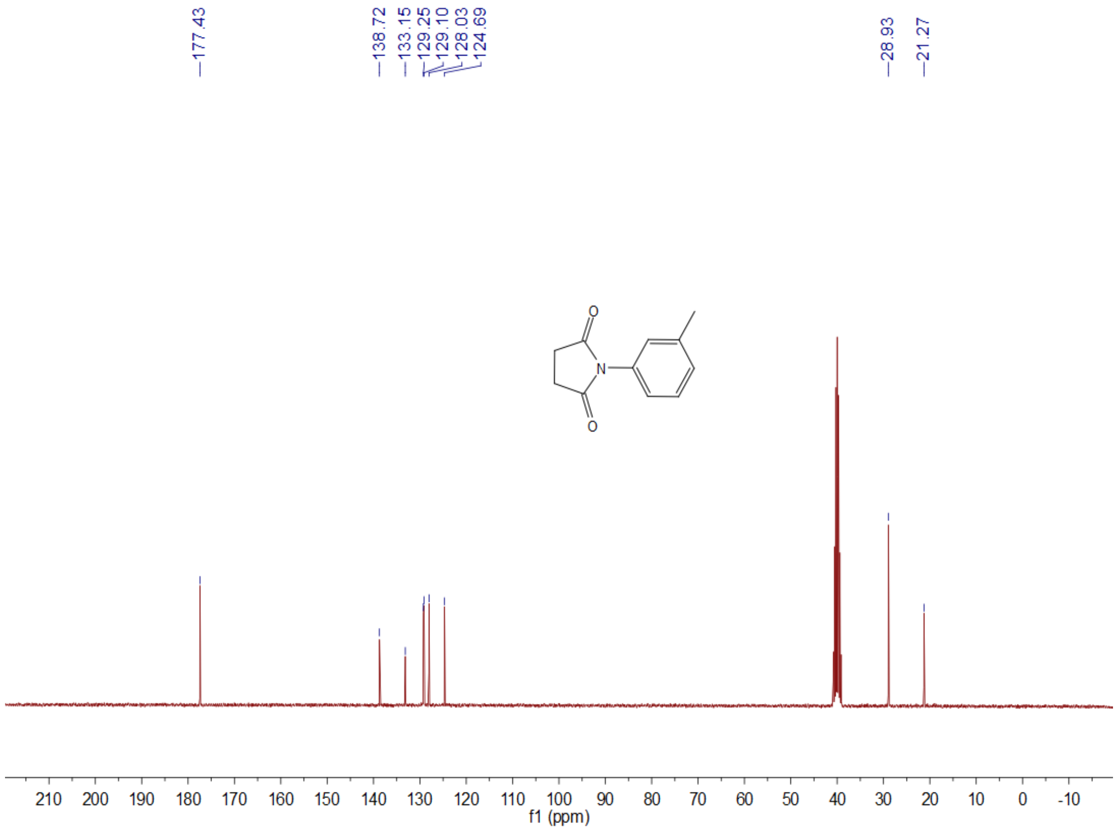

1-(4-Methoxyphenyl)pyrrolidine-2,5-dione (3k)

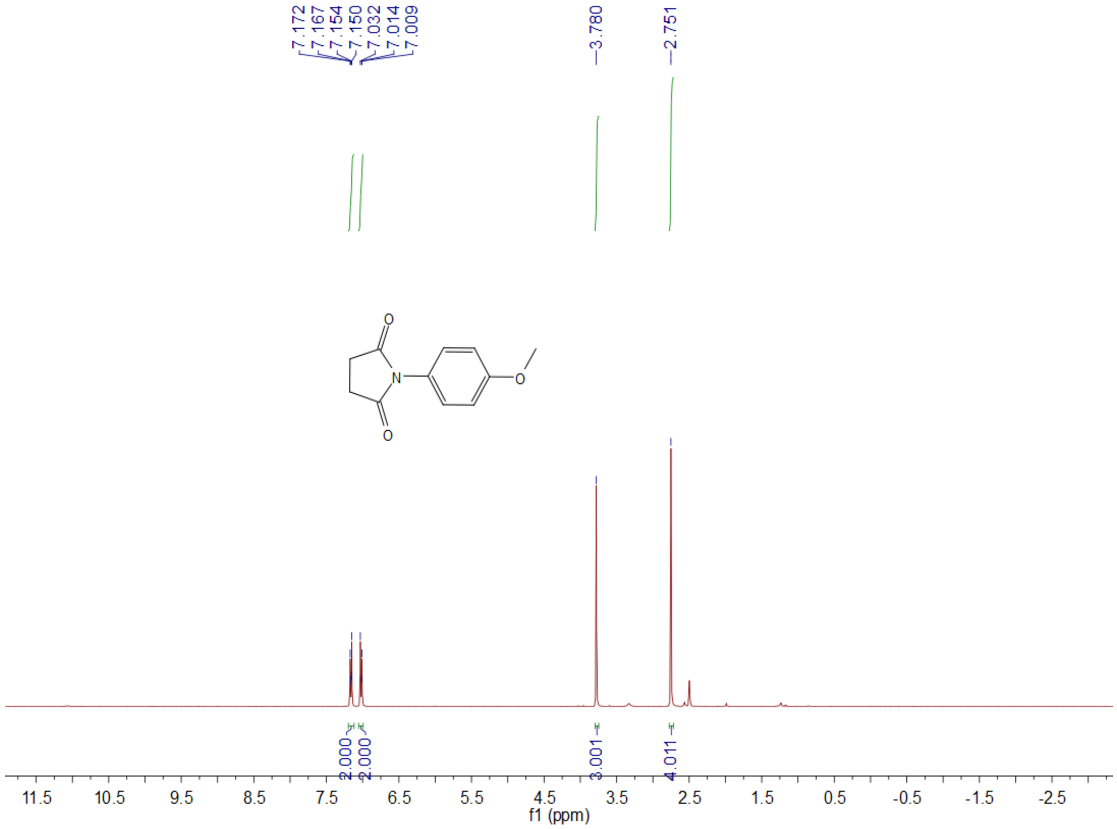

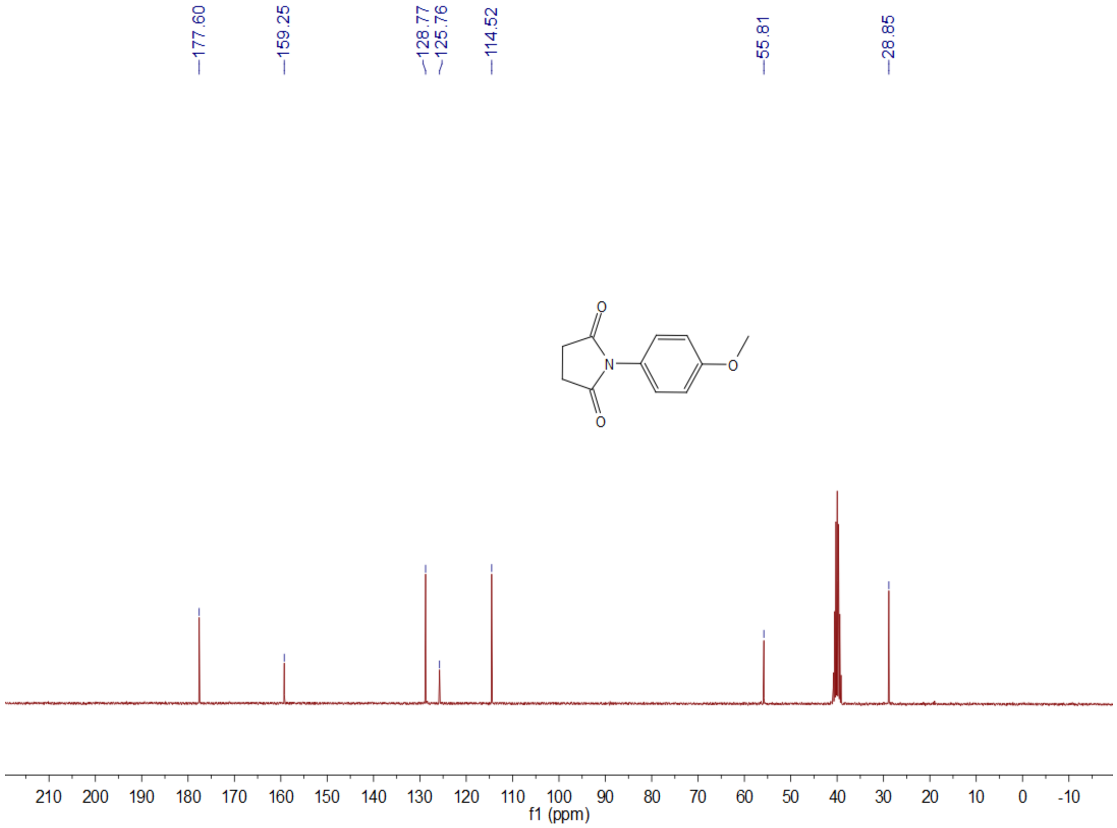

1-(4-Fluorophenyl)pyrrolidine-2,5-dione (3l)

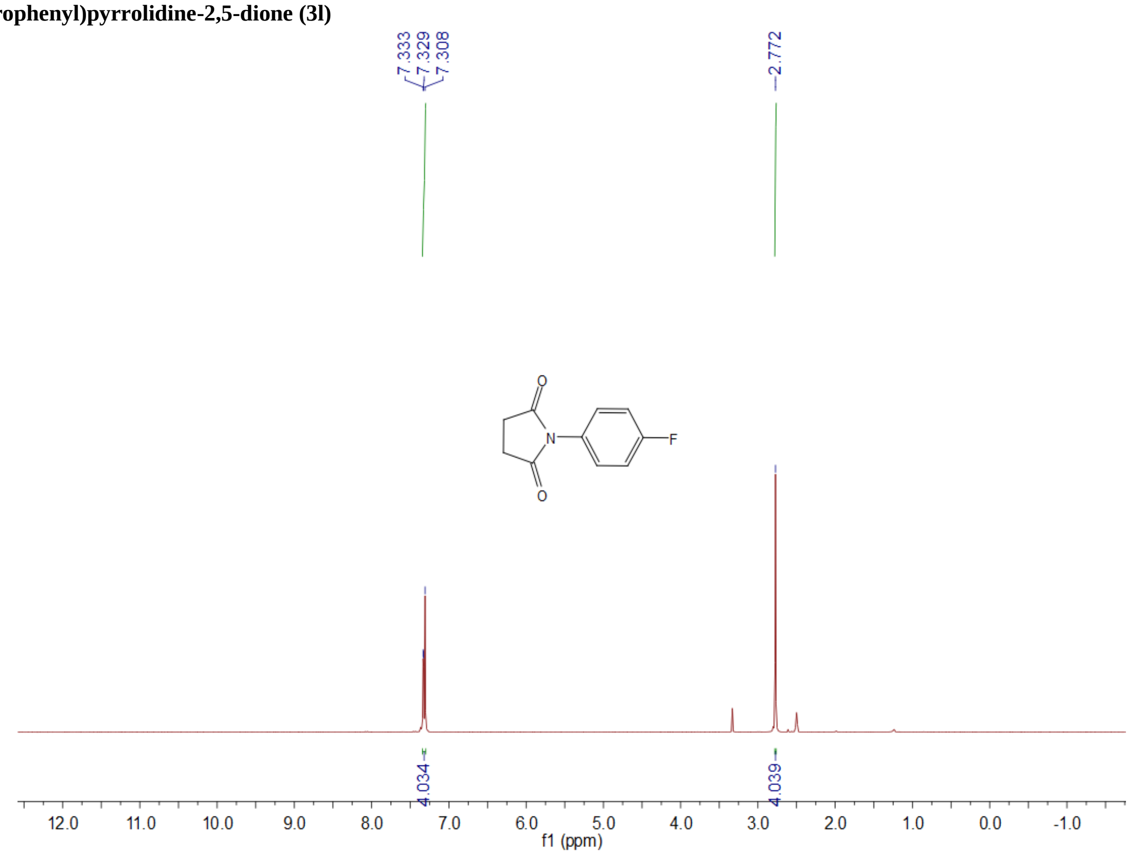

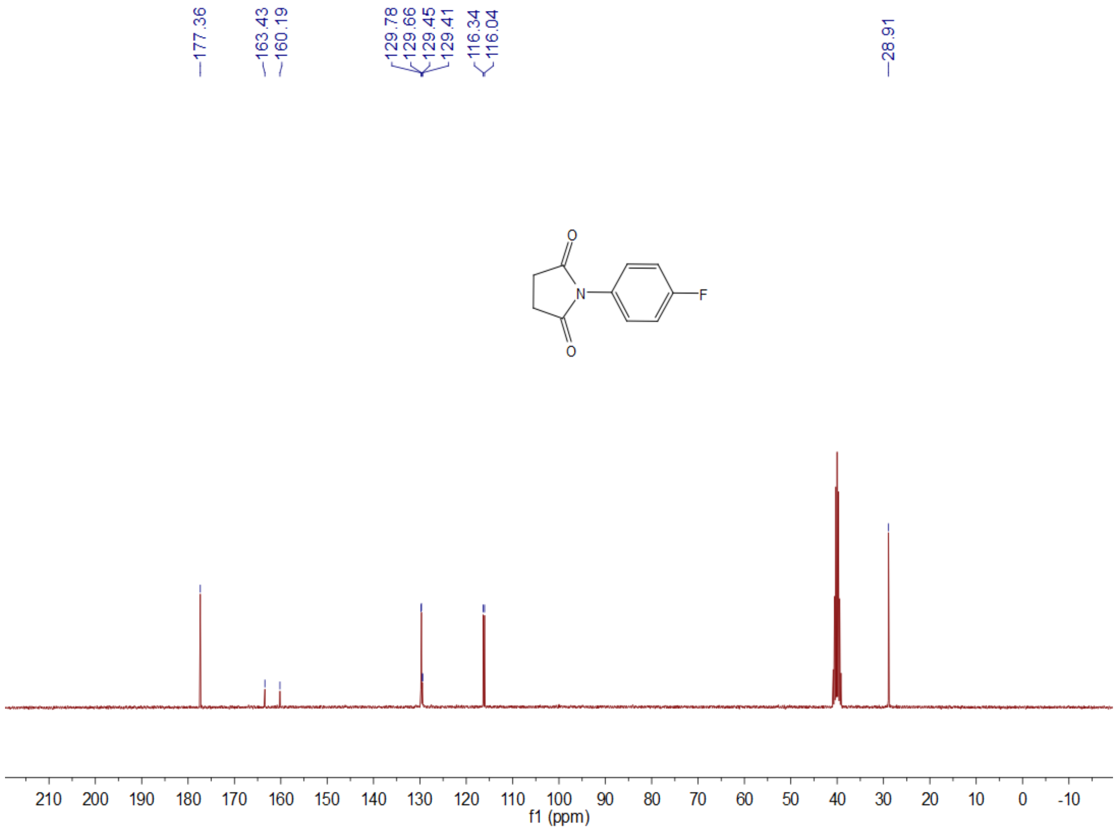

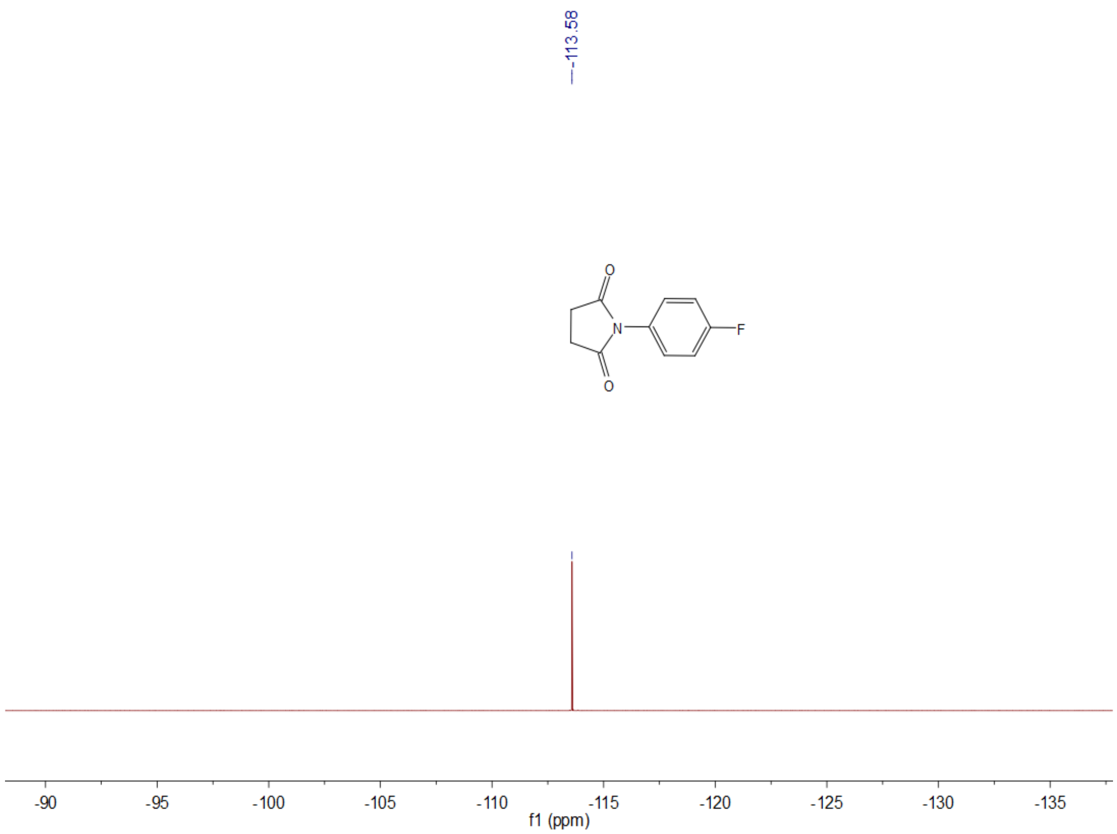
-113.58
O
N
F
O
-90
-95
-100
-105
-110
-115
-120
-125
-130
-135
f1 (ppm)

1-(4-Chlorophenyl)pyrrolidine-2,5-dione (3m)

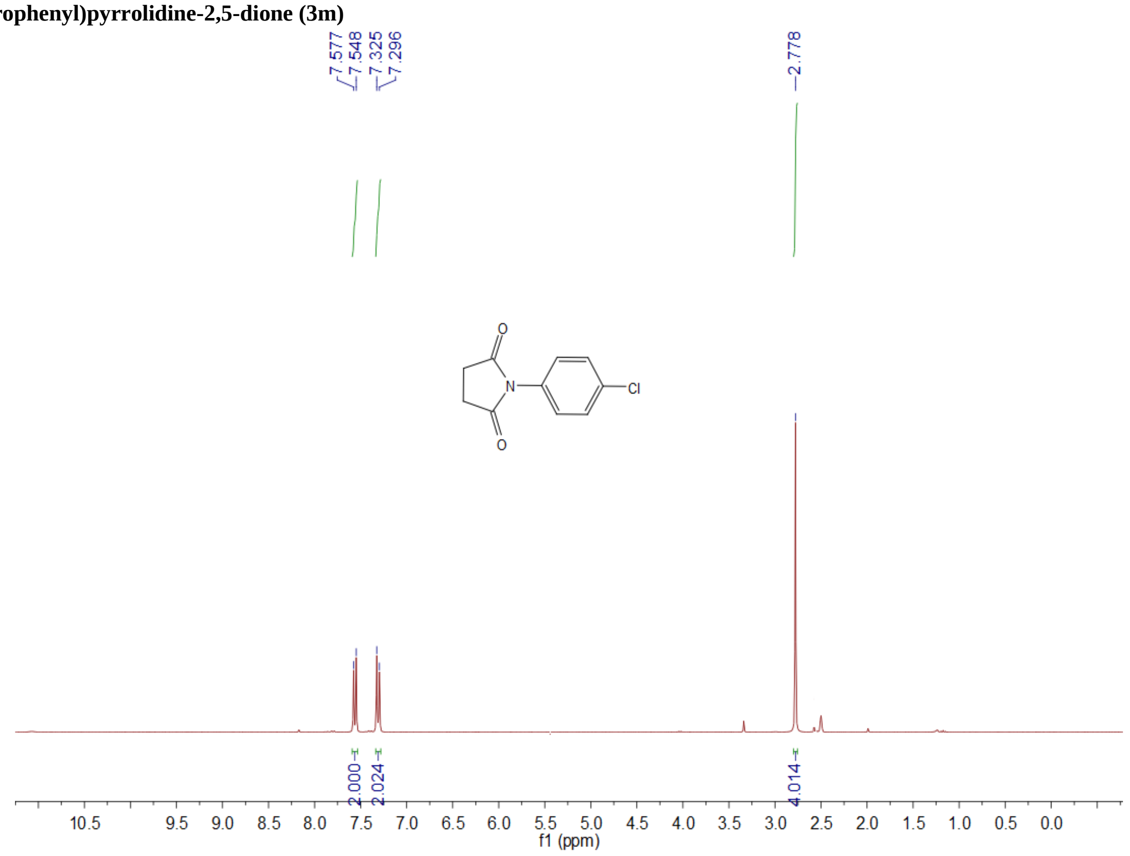

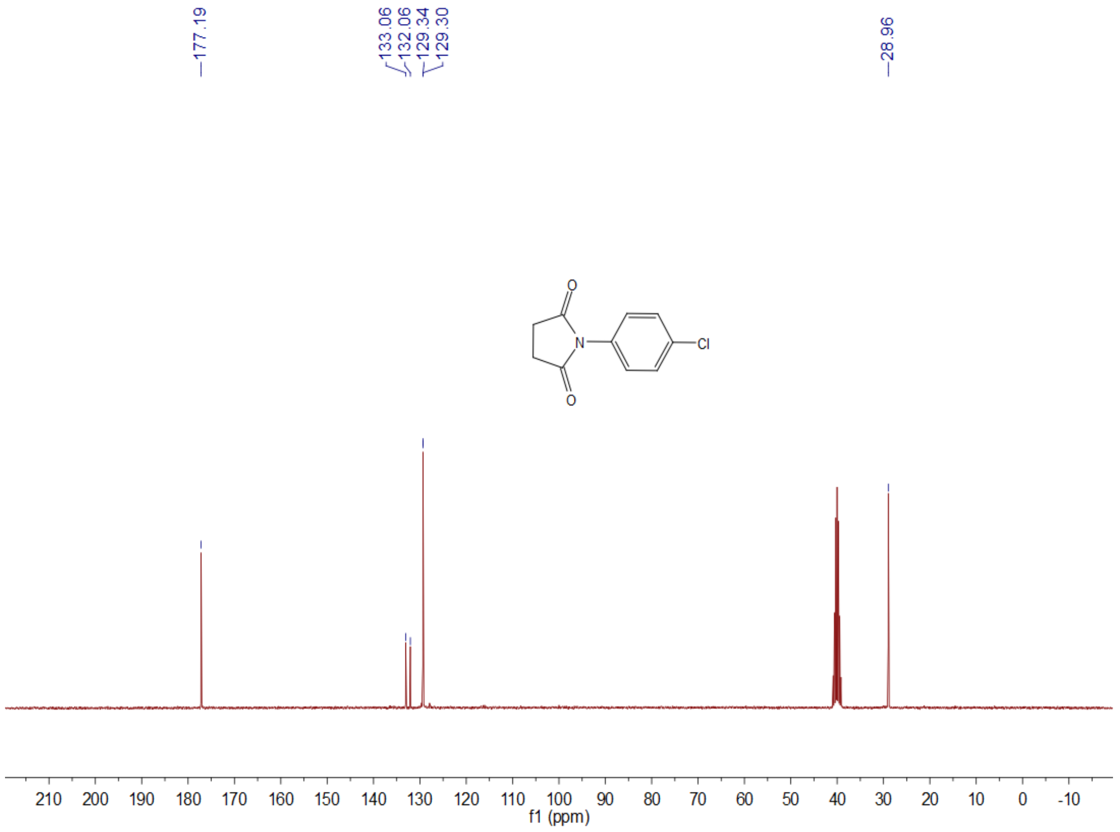

1-(4-Bromophenyl)pyrrolidine-2,5-dione (3n)

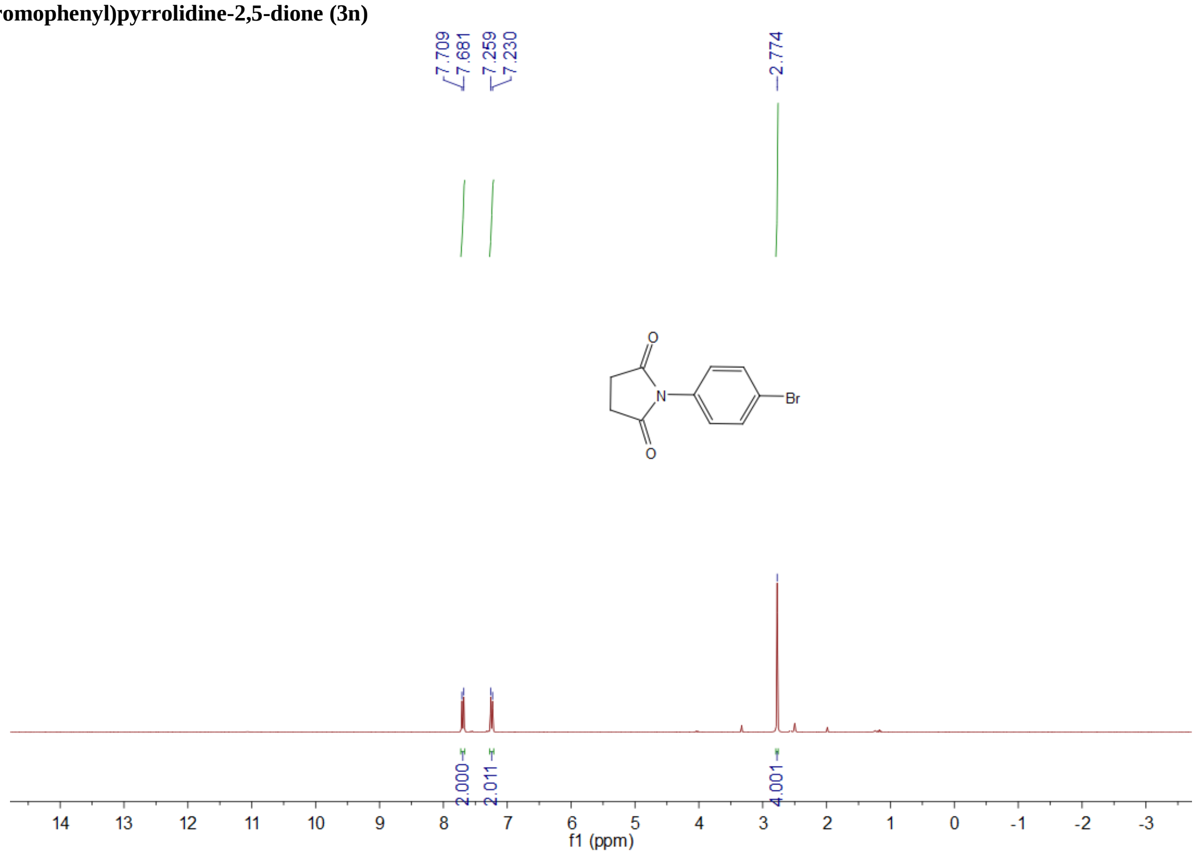

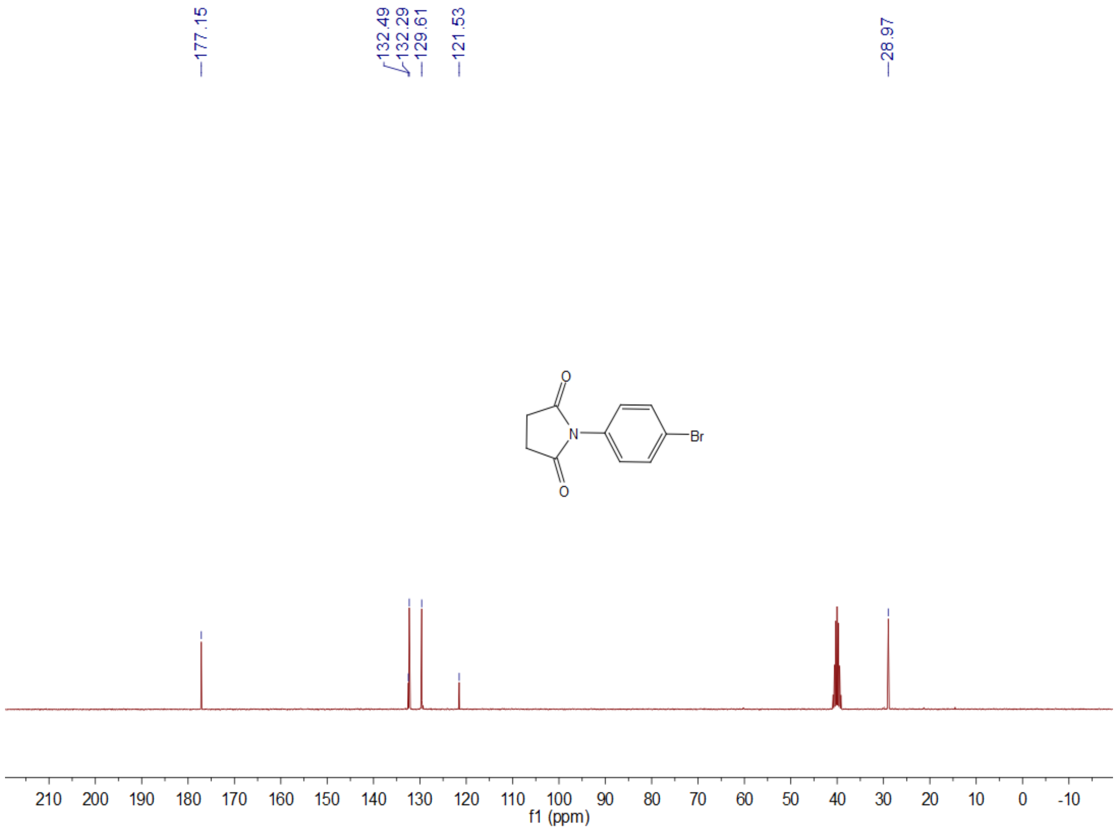

1-(4-Iodophenyl)pyrrolidine-2,5-dione (3o)

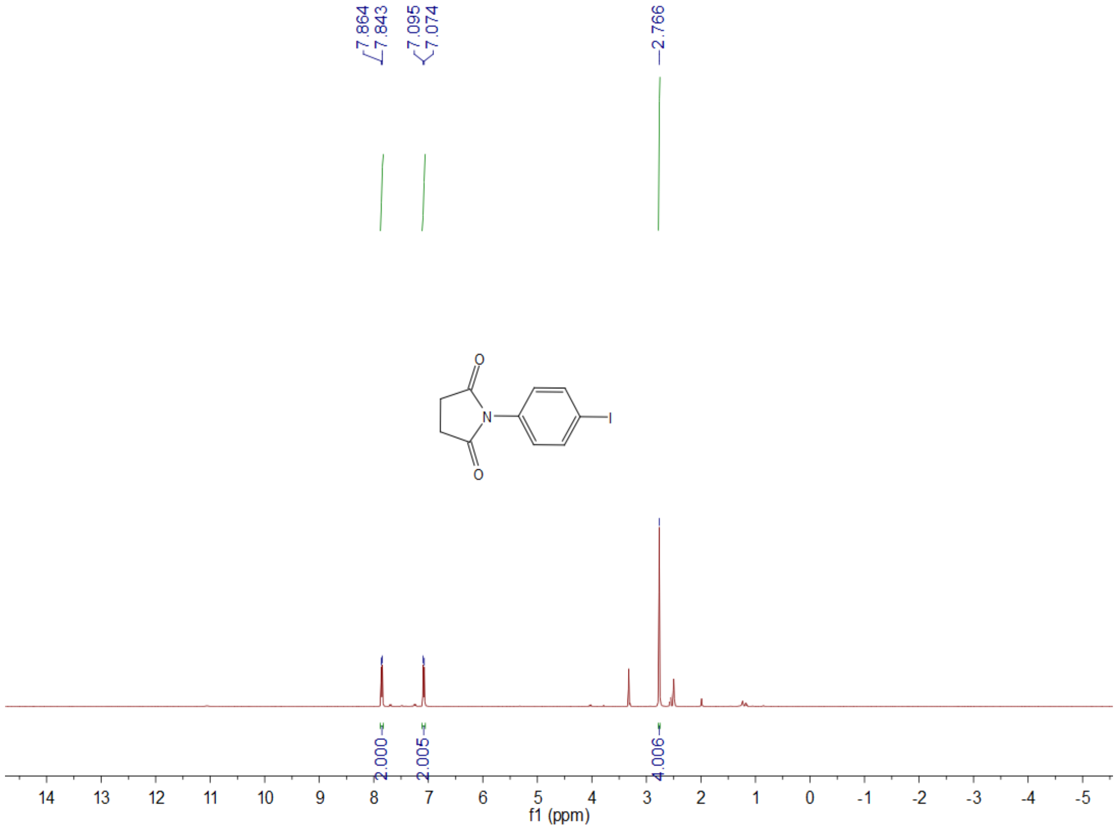

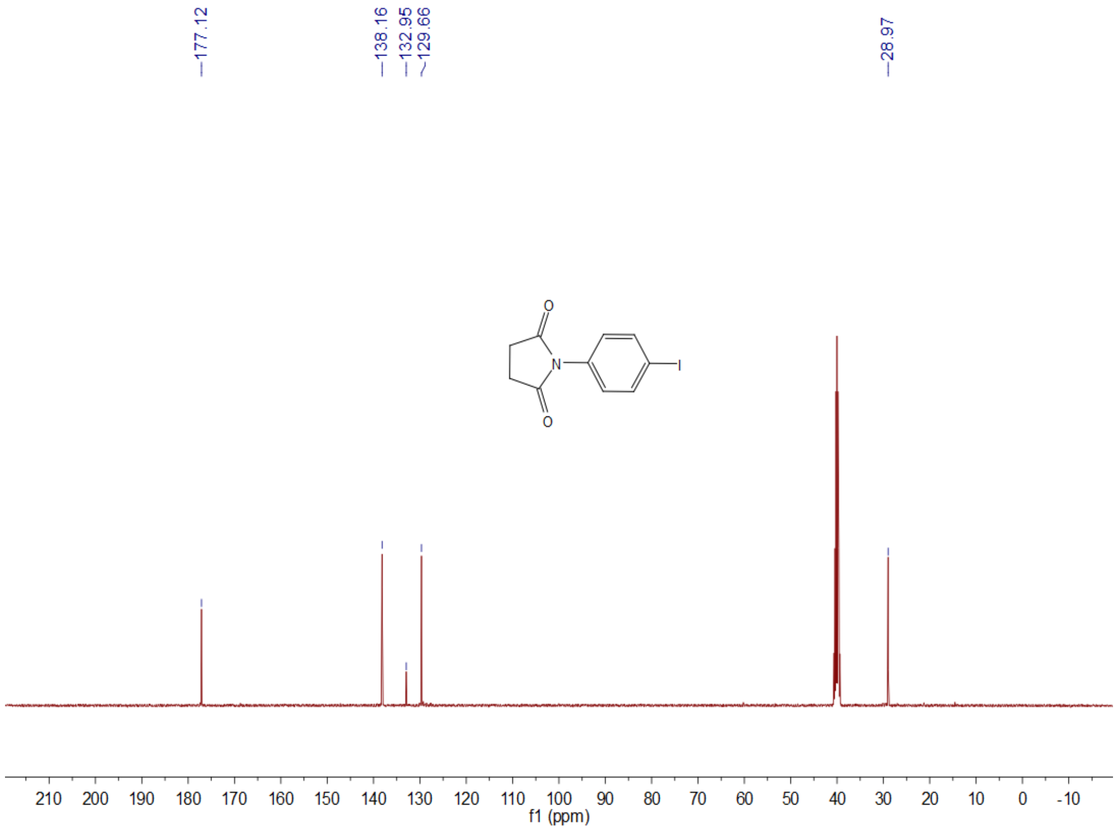

1-(4-(Methylthio)phenyl)pyrrolidine-2,5-dione (3r)

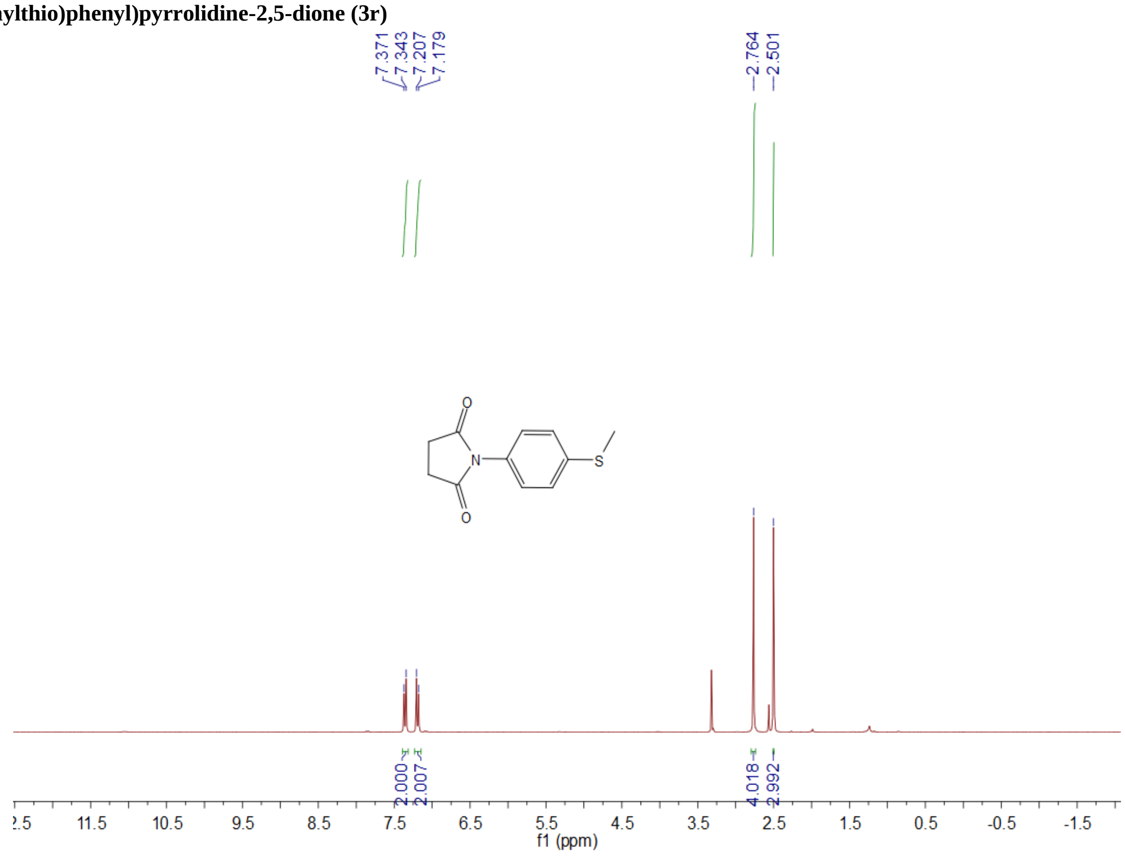

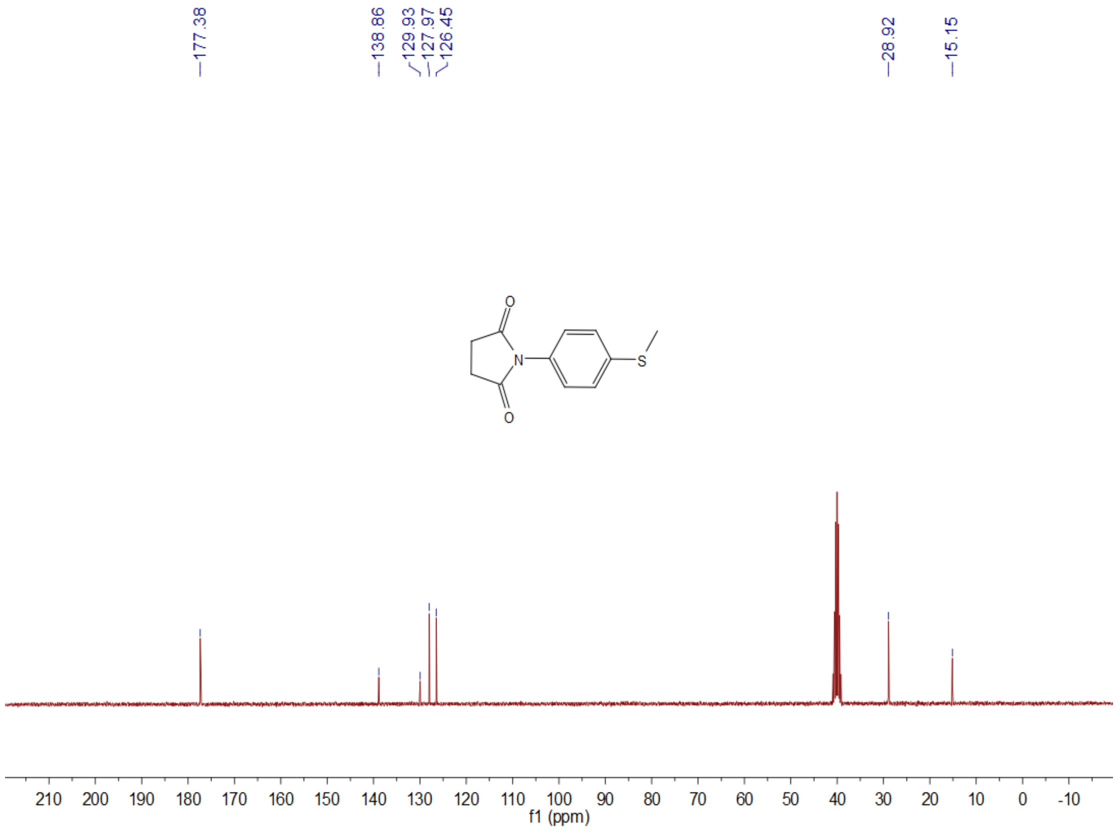

***N, N'*-Dibutylsuccinamide (4a)**

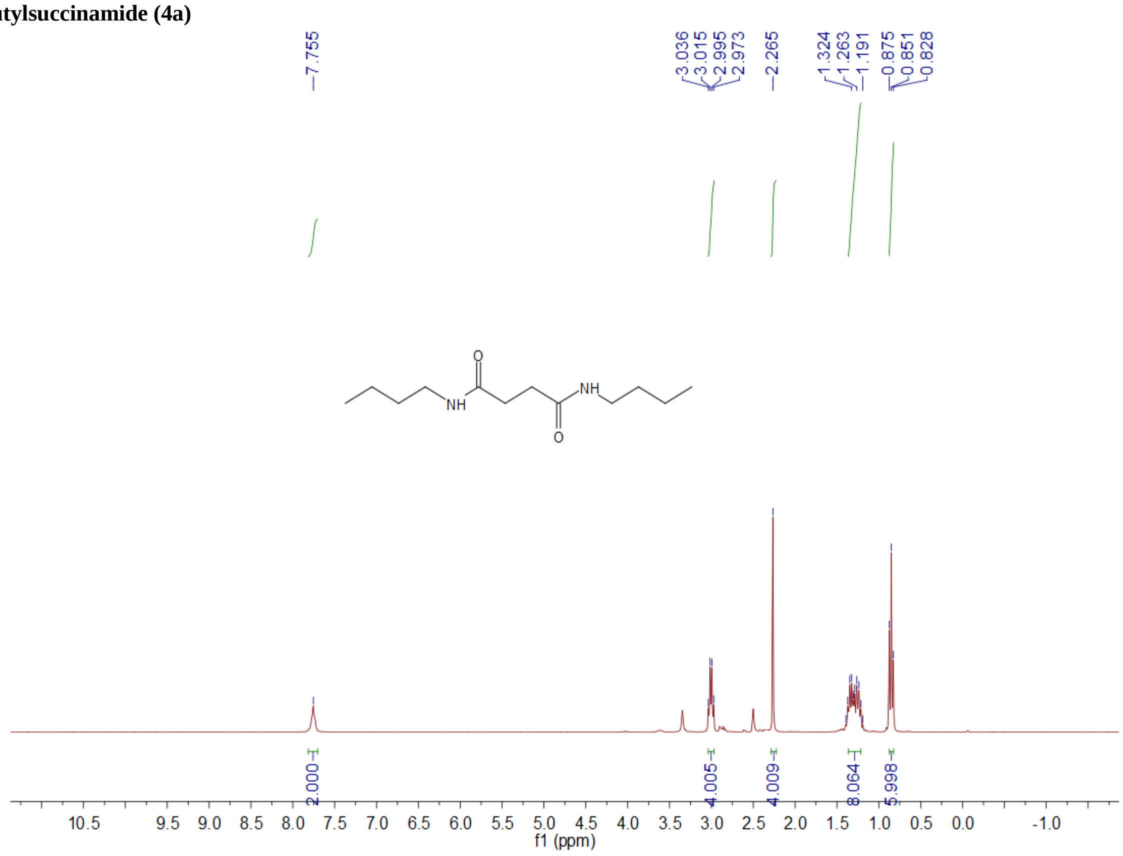

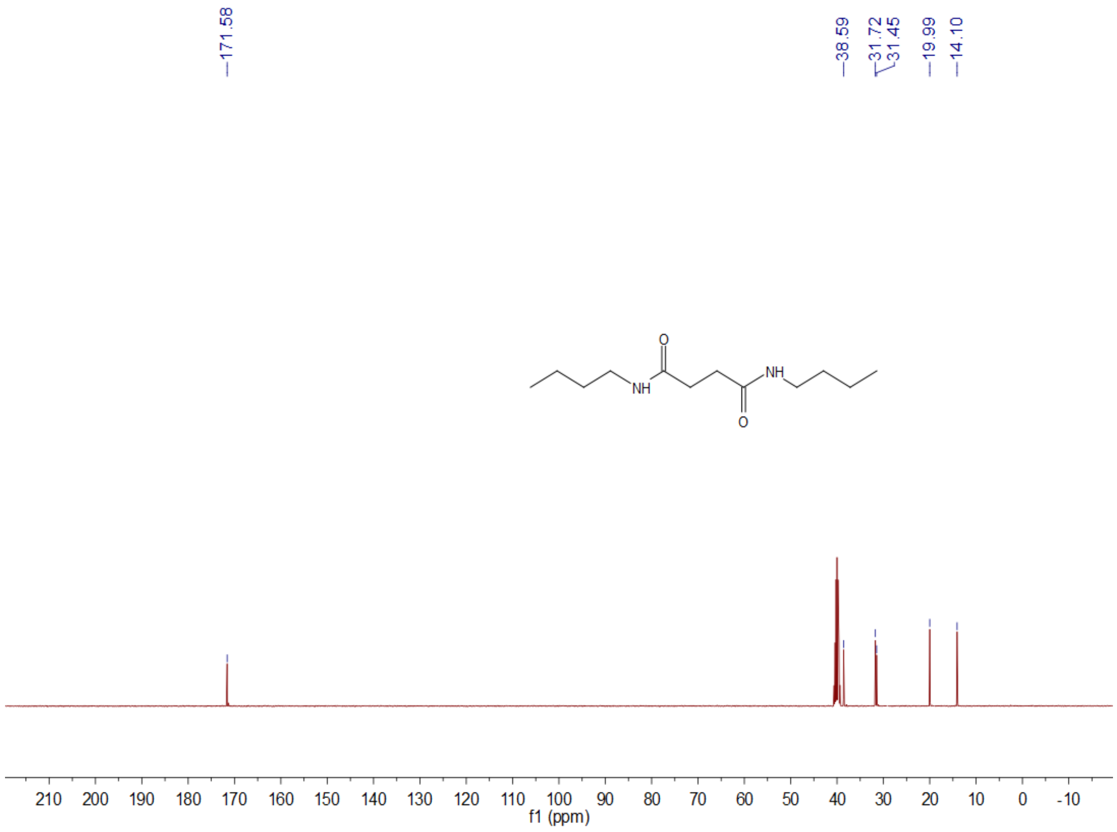

Succinamide (4b)

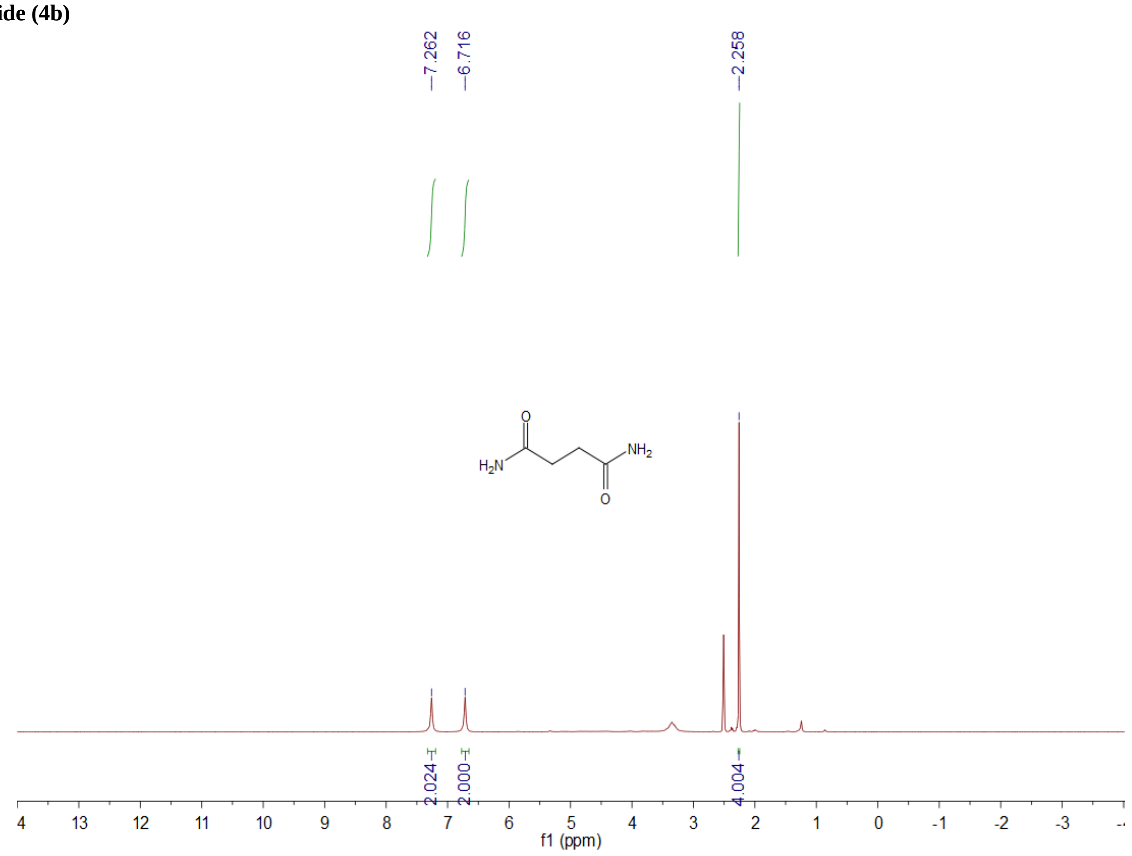

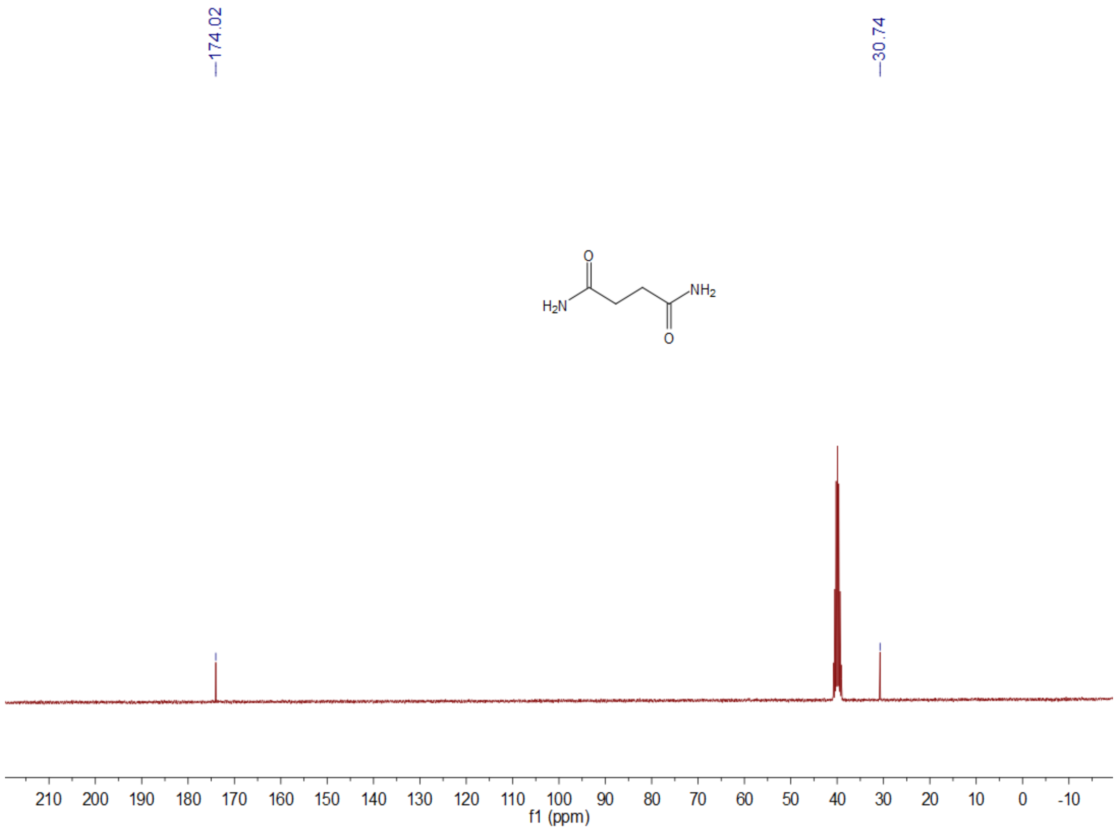

***N*, *N'*-Dimethylsuccinamide (4c)**

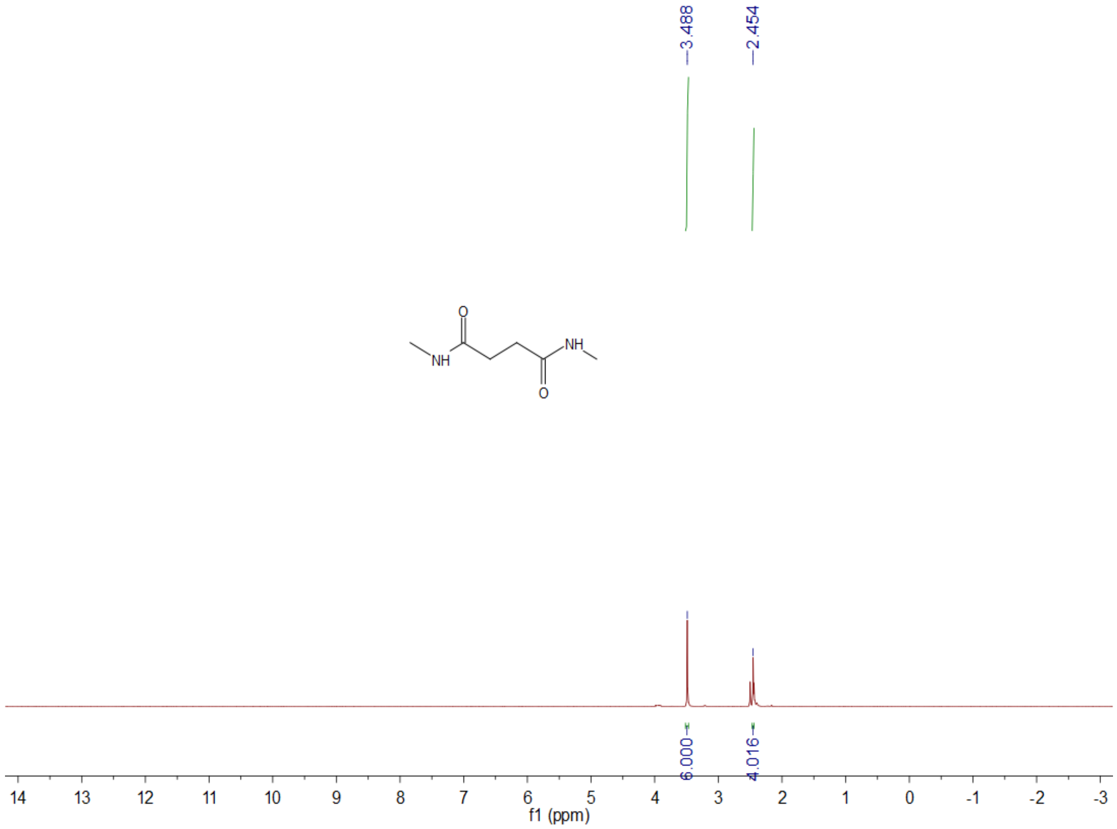

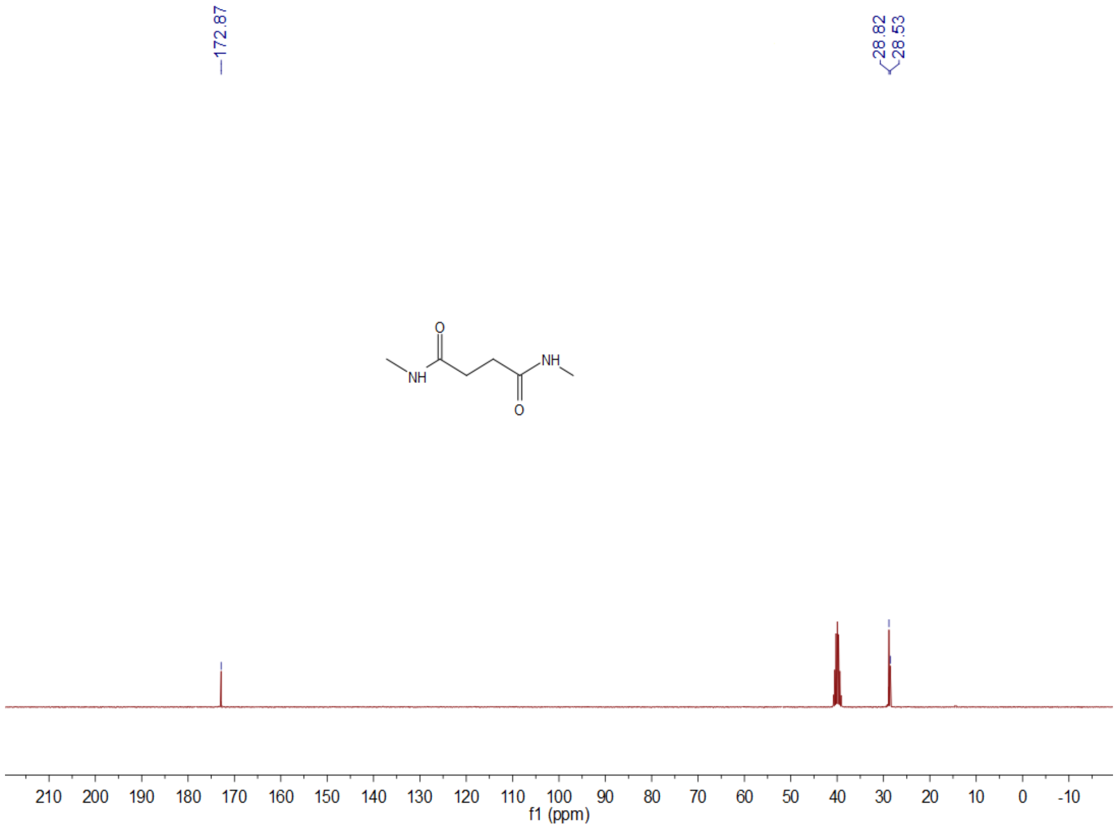

***N, N'*-diethylsuccinamide (4d)**

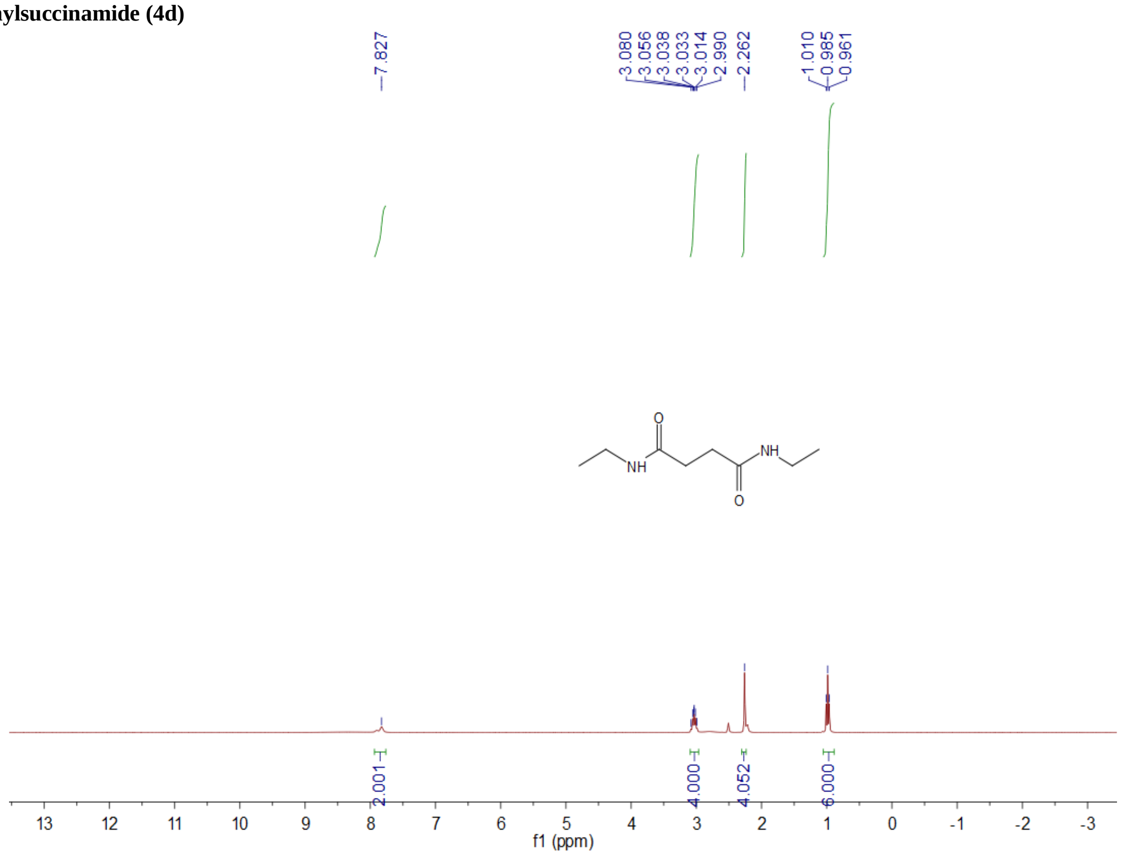

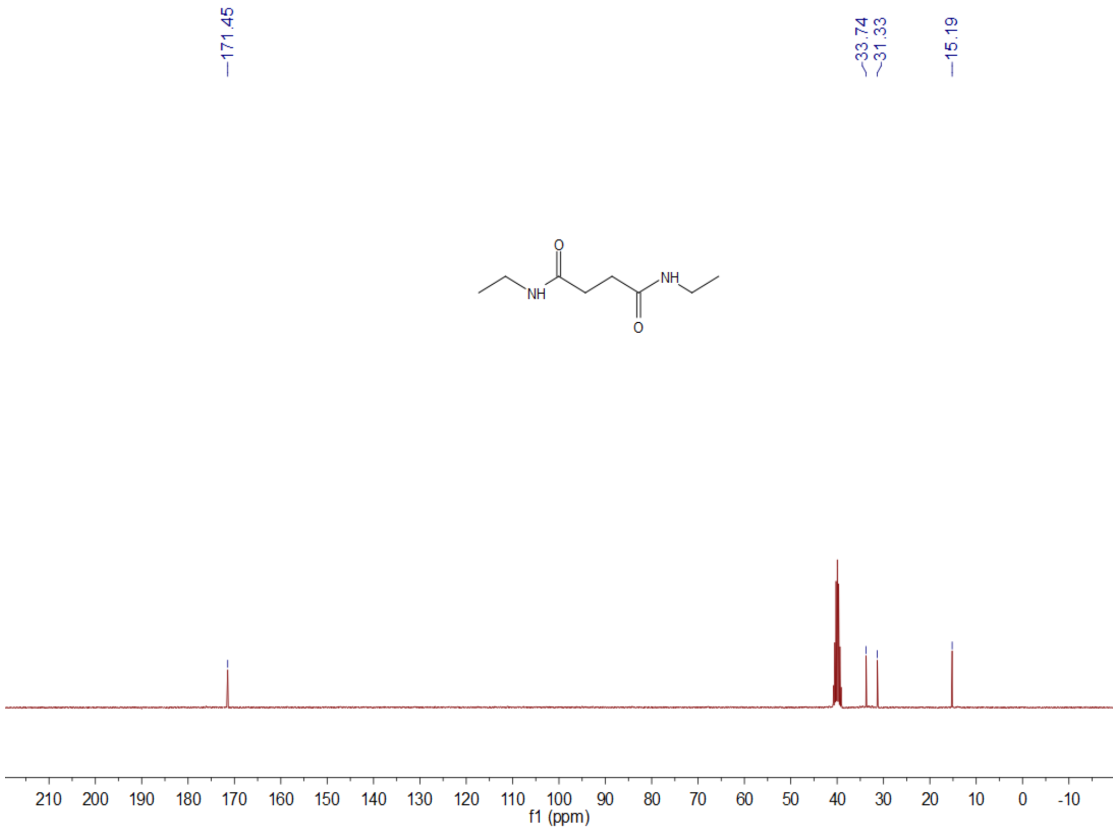

***N, N'*-Diisopropylsuccinamide (4e)**

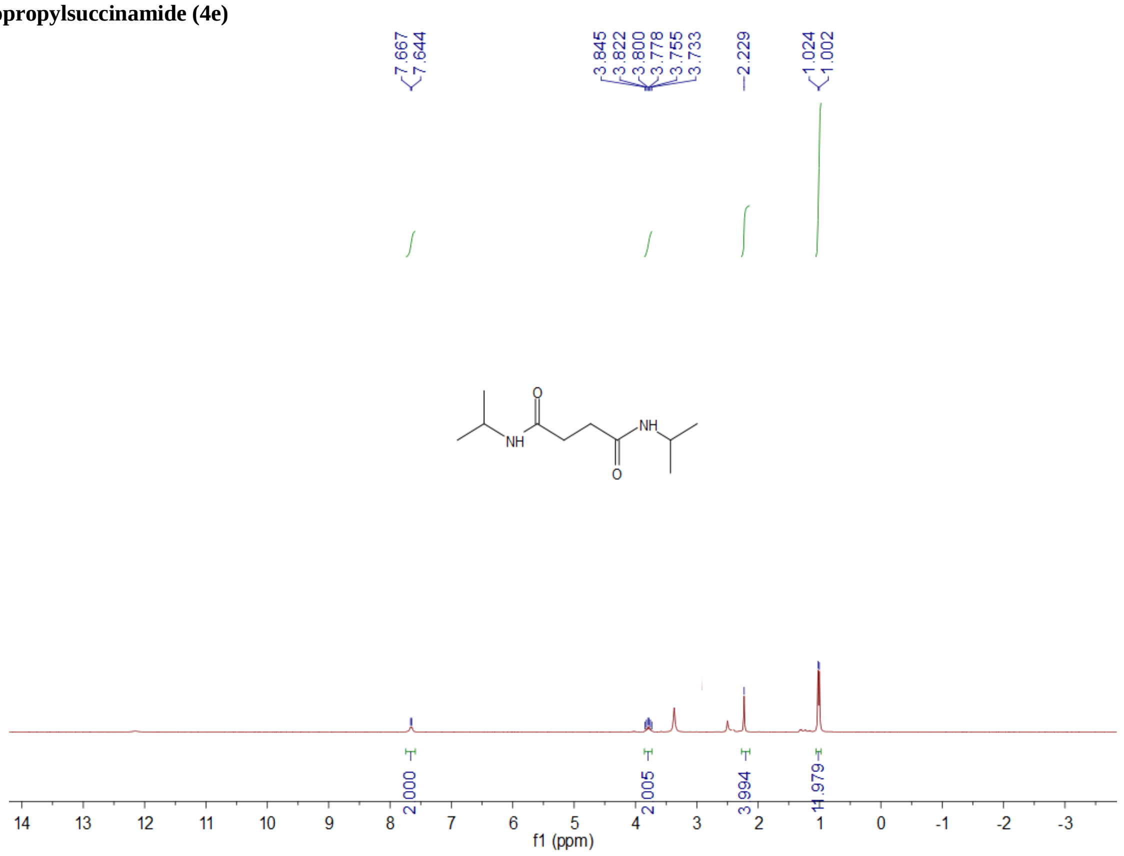

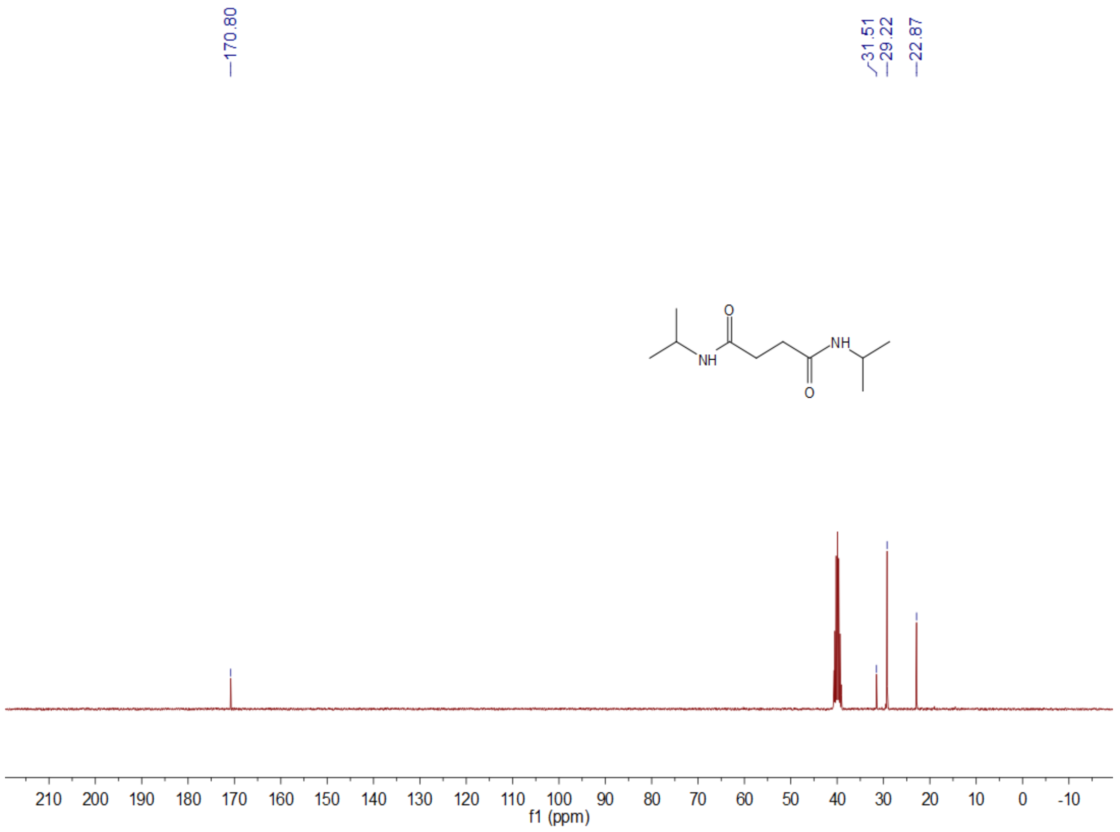

***N*, *N'*-Dihexylsuccinamide (4f)**

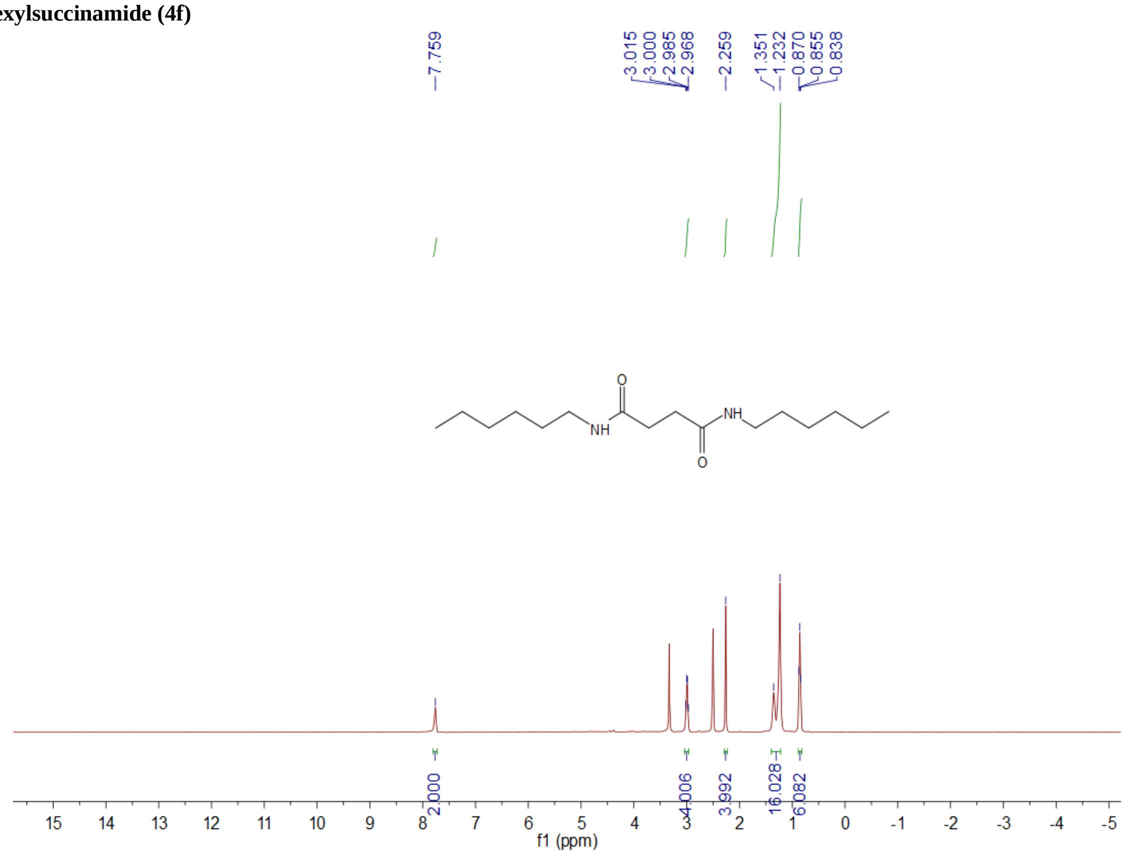

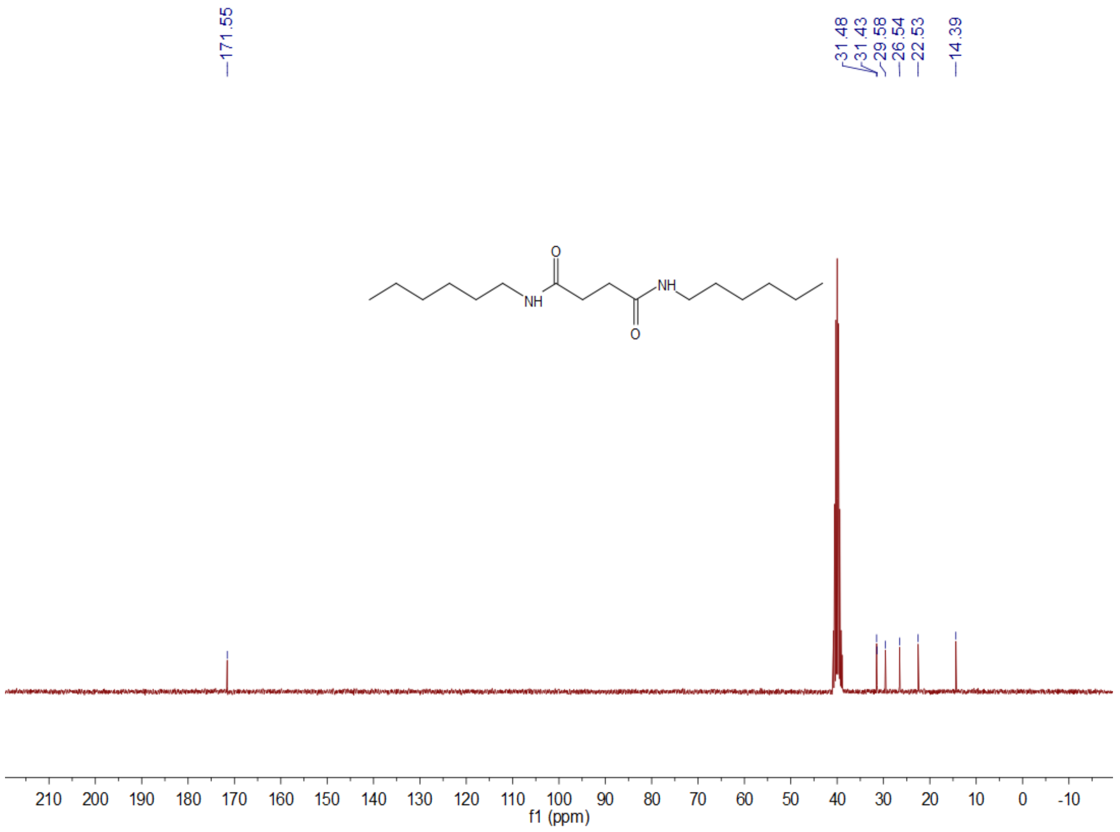

***N, N'*-Dibenzylsuccinamide (4g)**

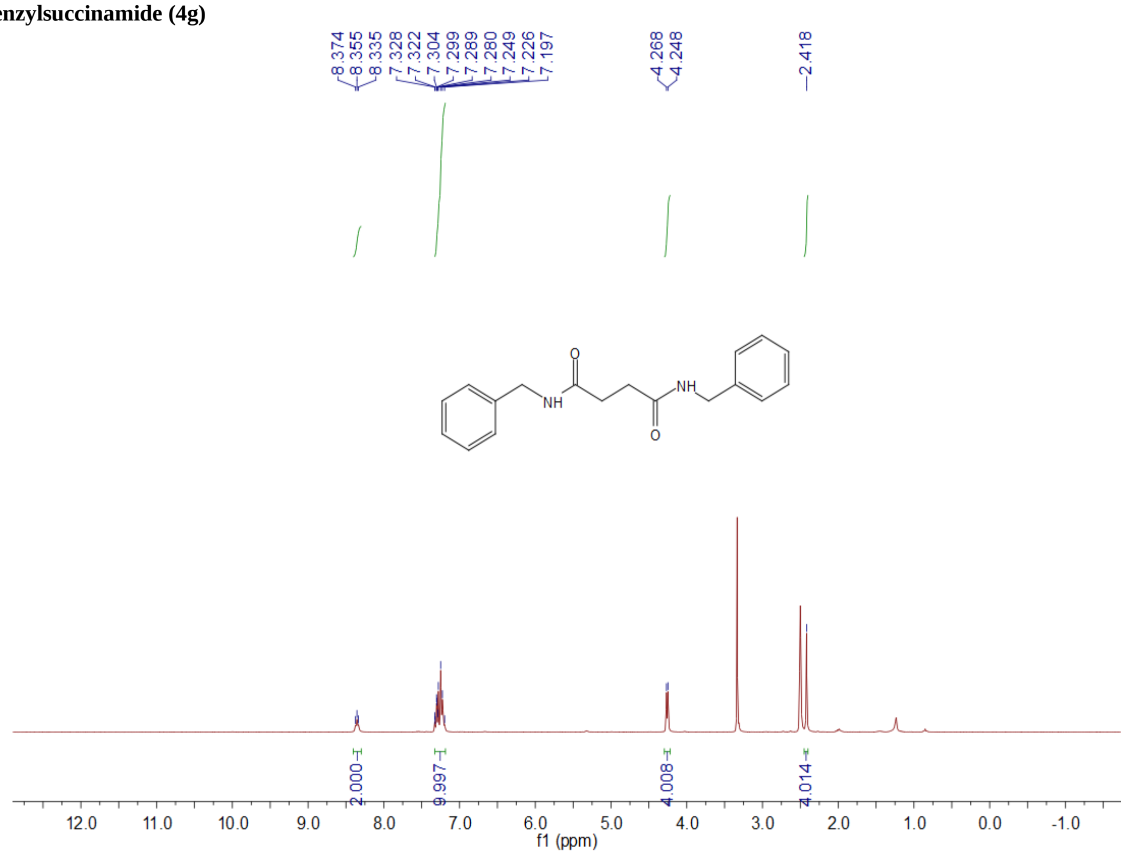

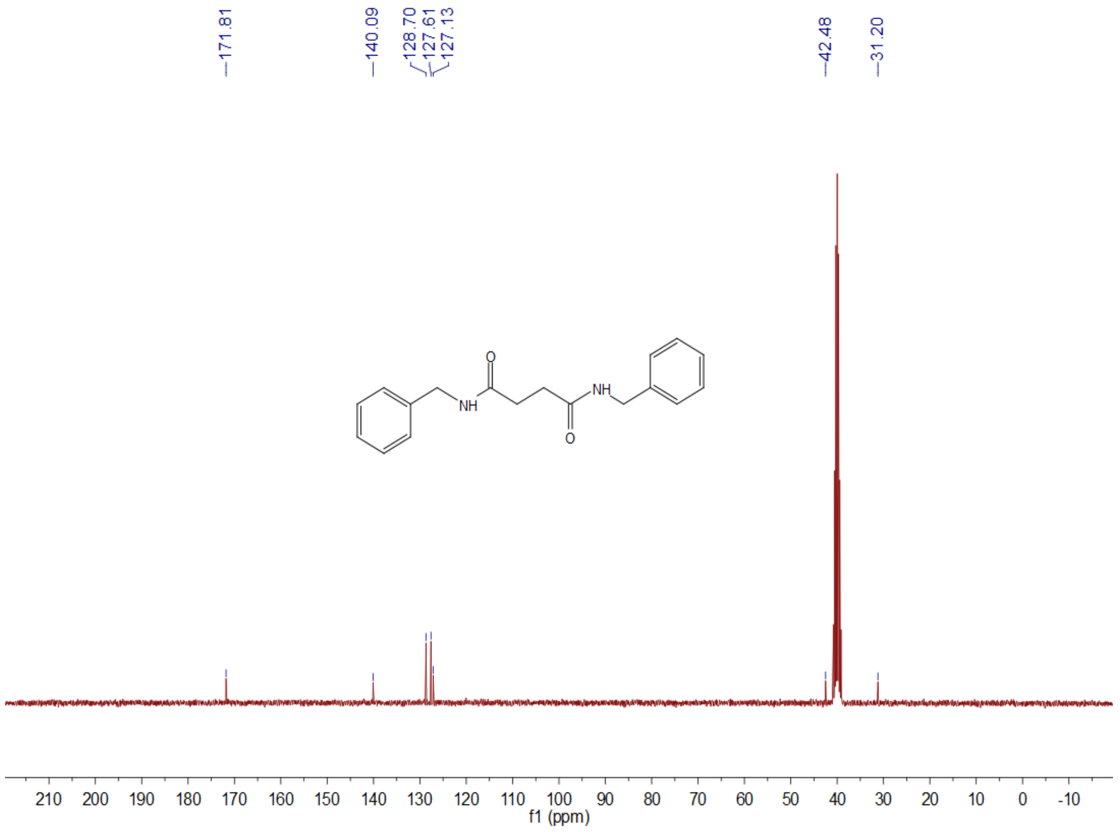

***N*, *N'*-Diphenylsuccinamide (4h)**

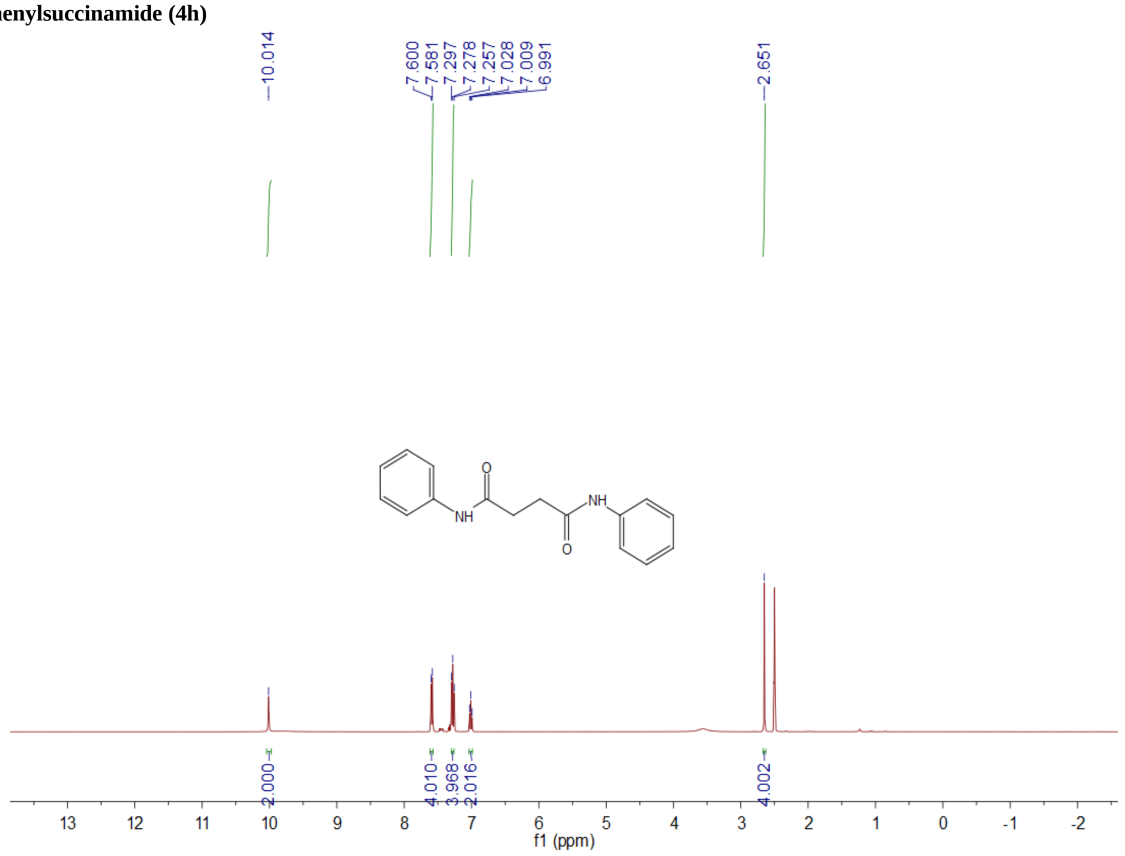

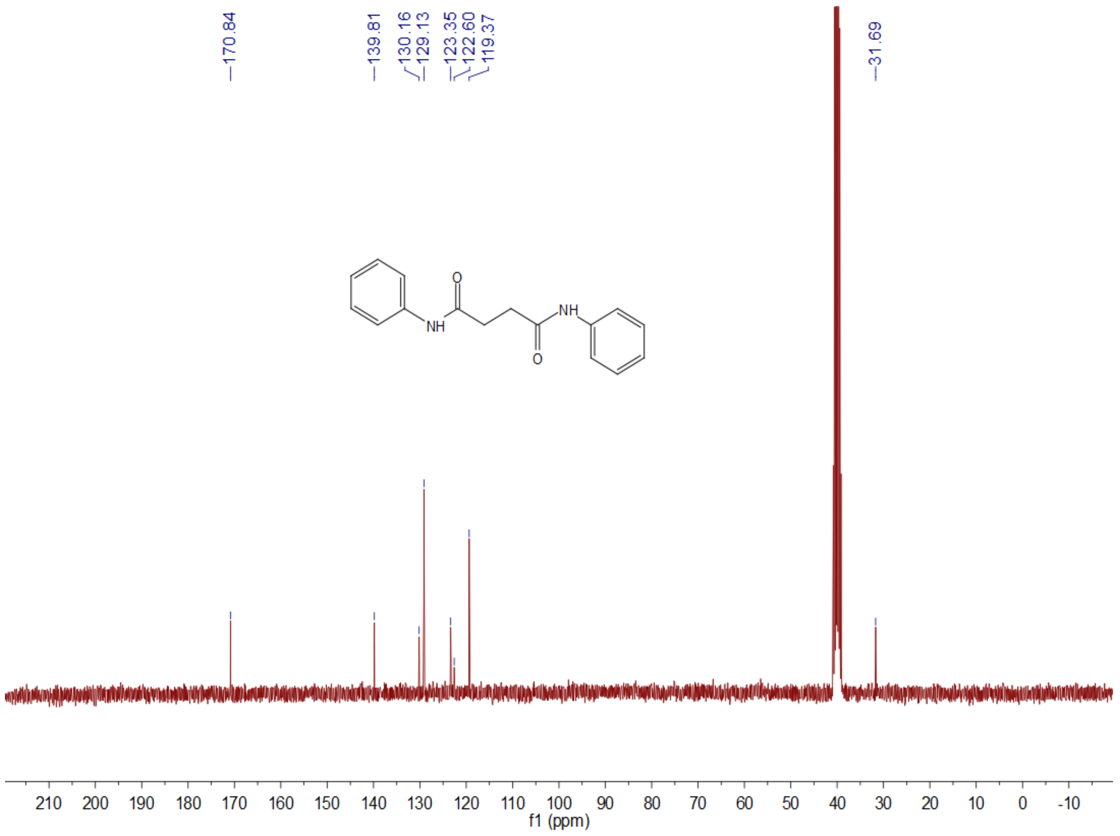

***N*, *N'*--Di-*p*-tolylsuccinamide (4i)**

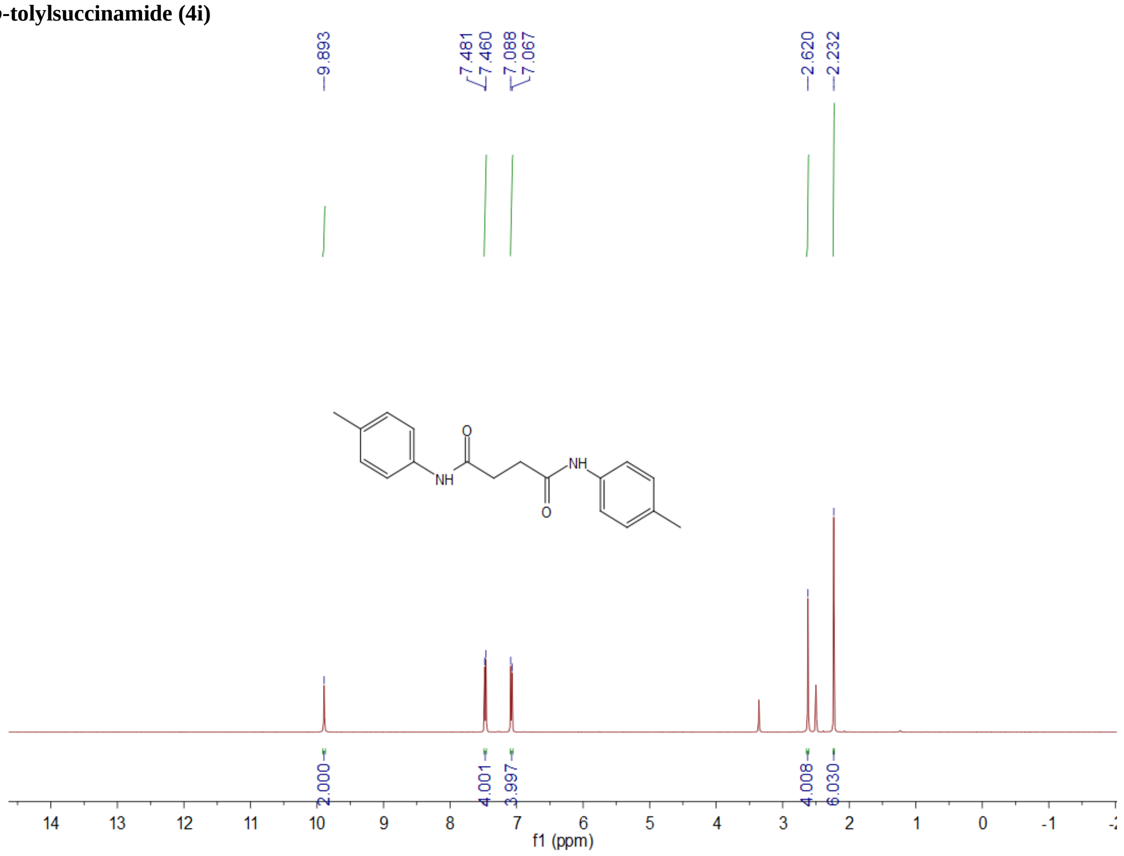

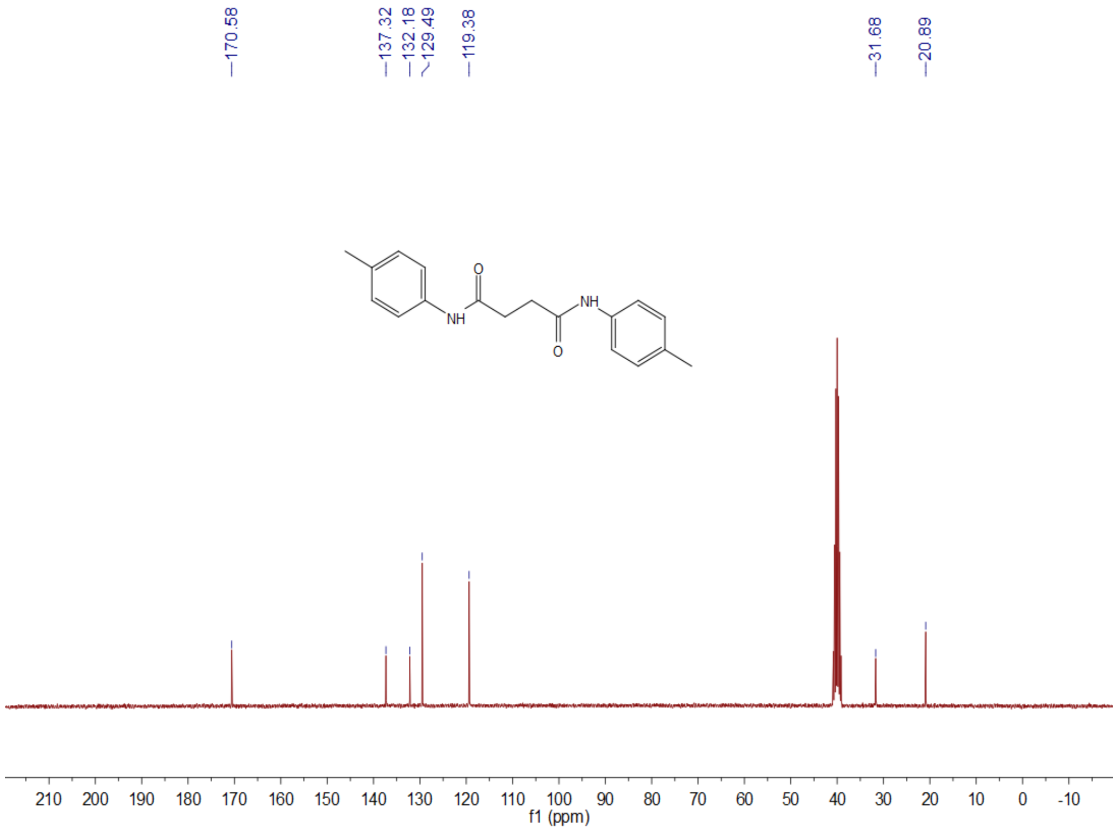

***N, N'*-Di-*m*-tolylsuccinamide (4j)**

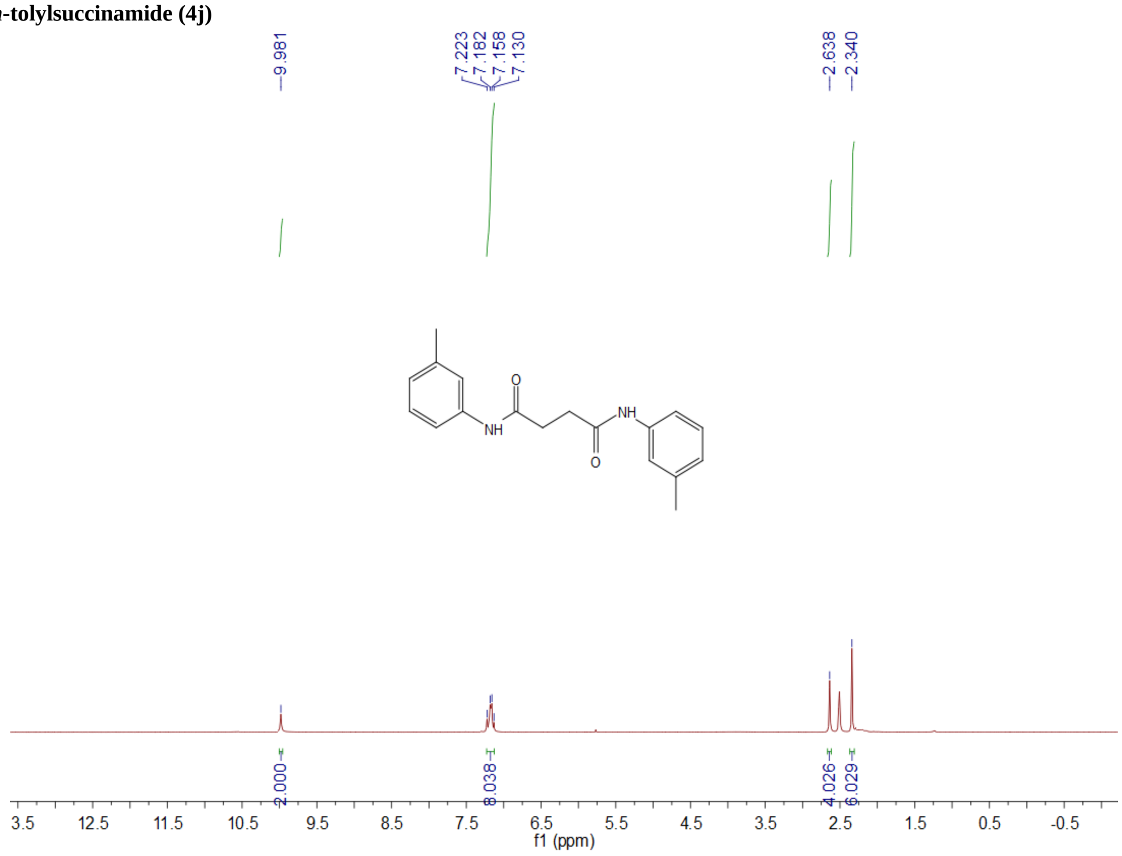

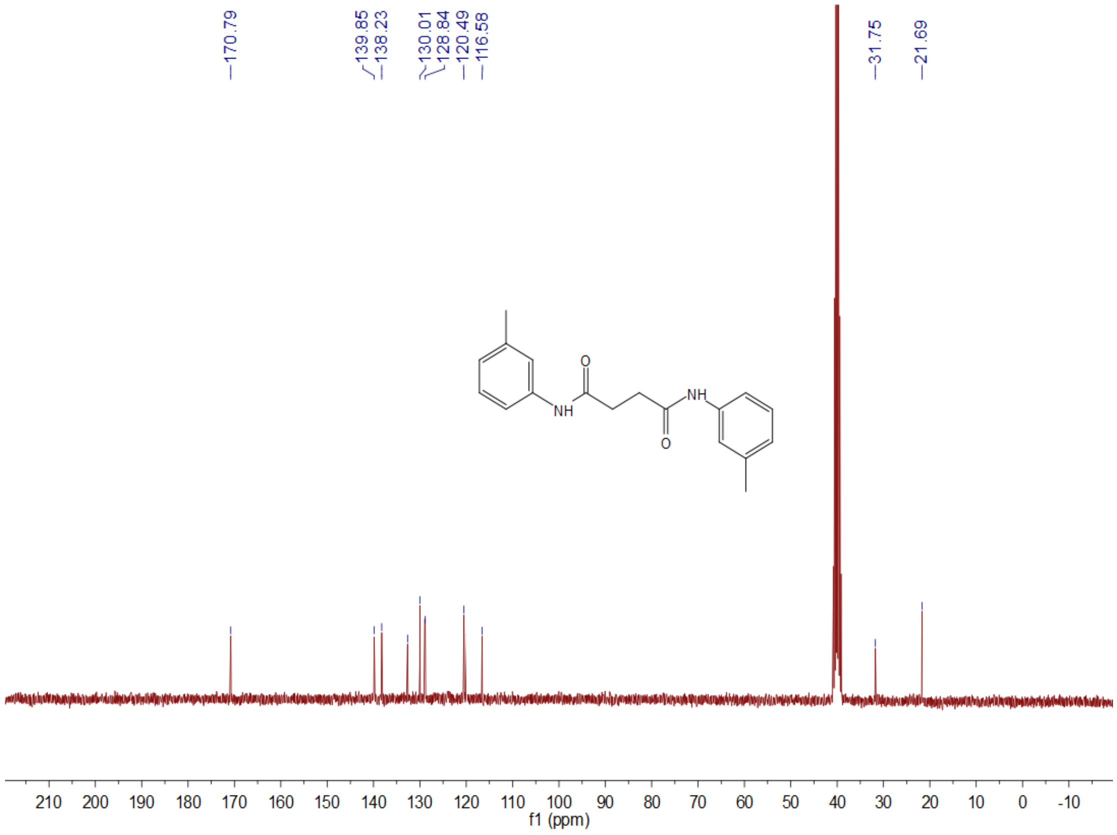

***N, N'*-Bis(4-methoxyphenyl)succinamide (4k)**

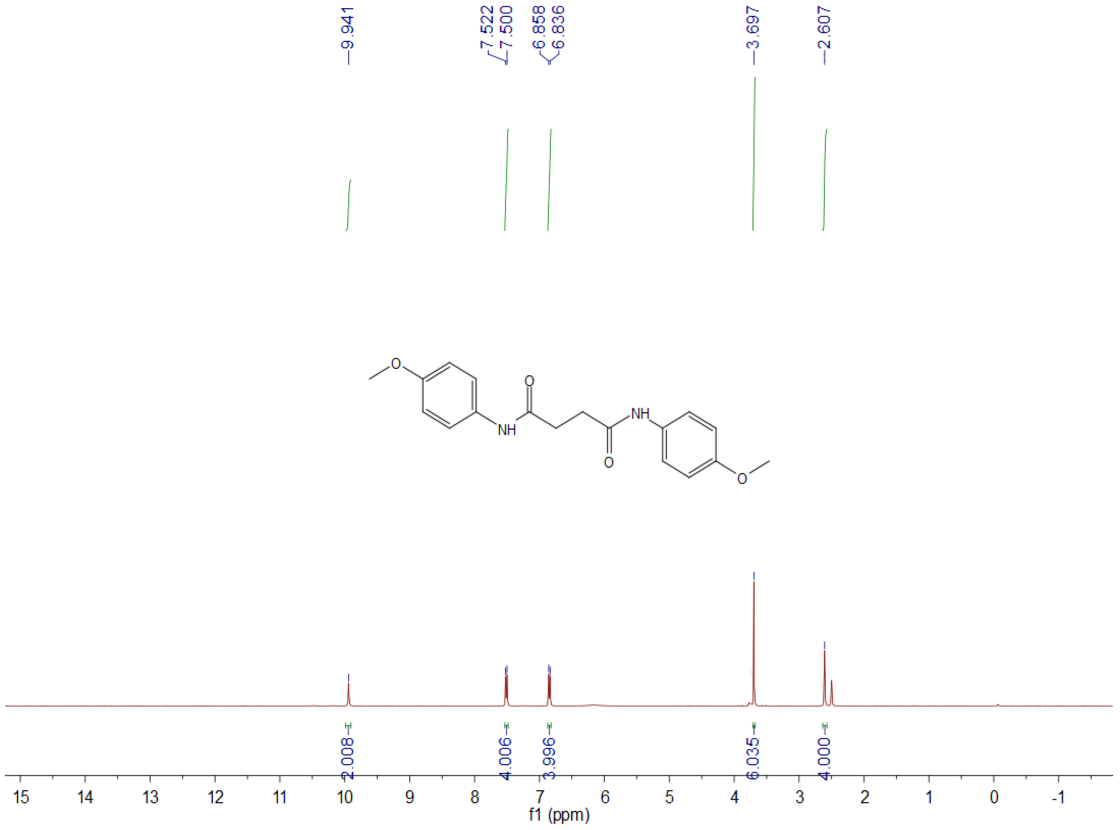

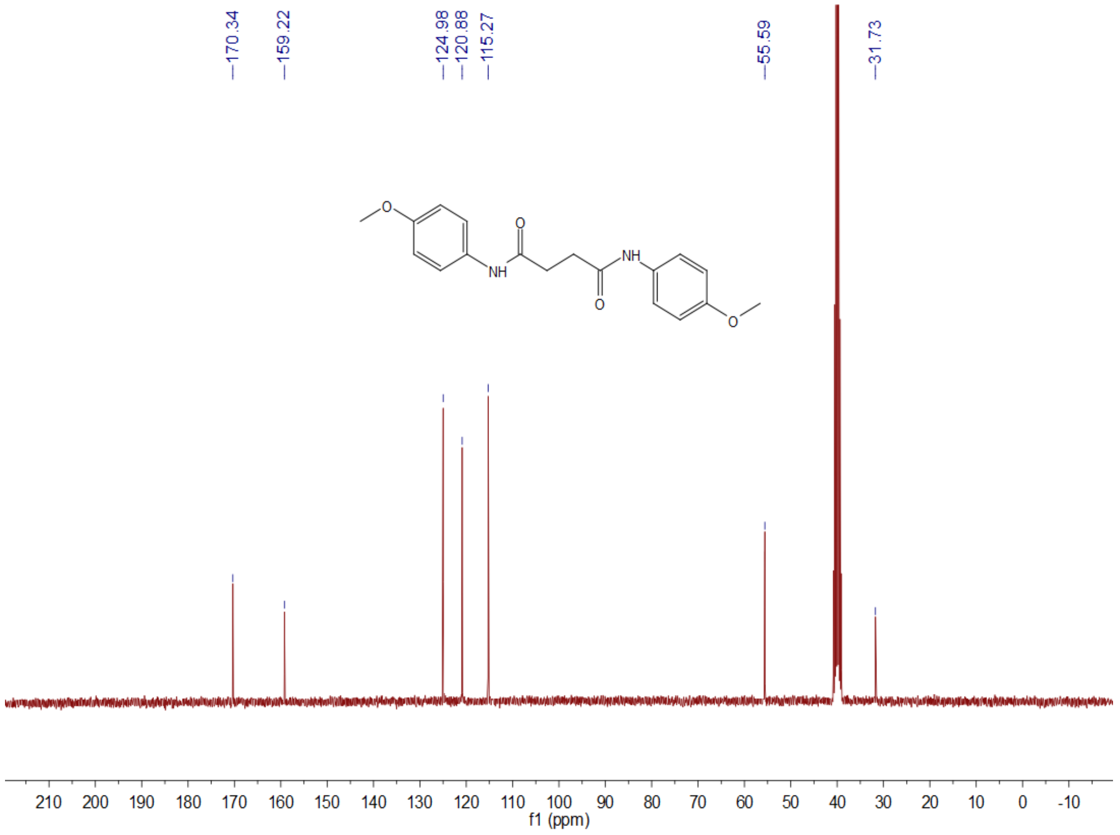

***N, N'*-Bis(4-fluorophenyl)succinamide (4l)**

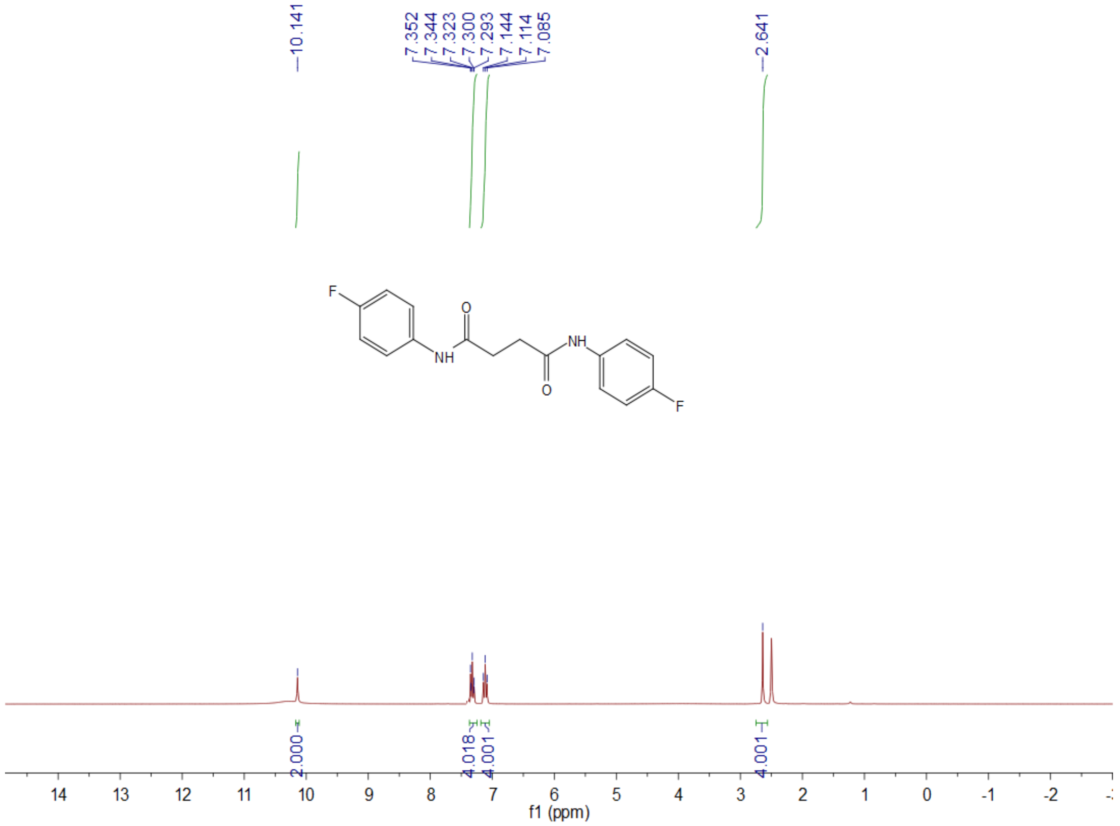

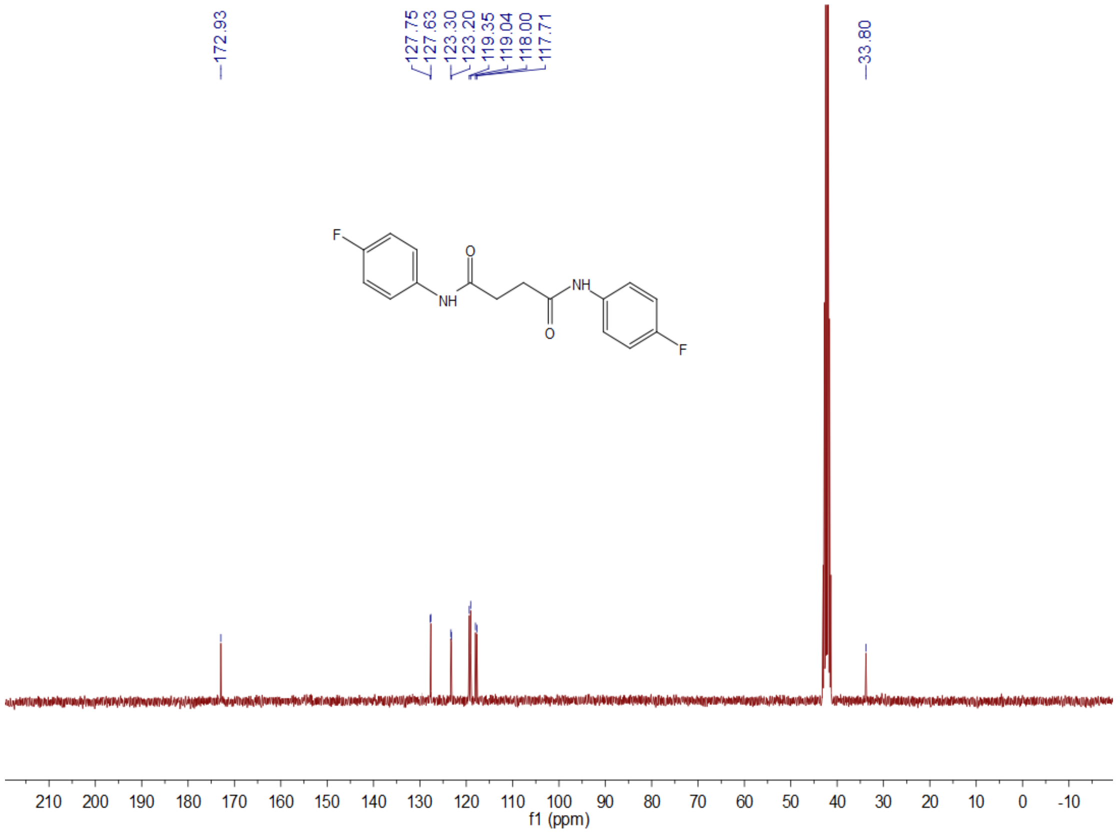

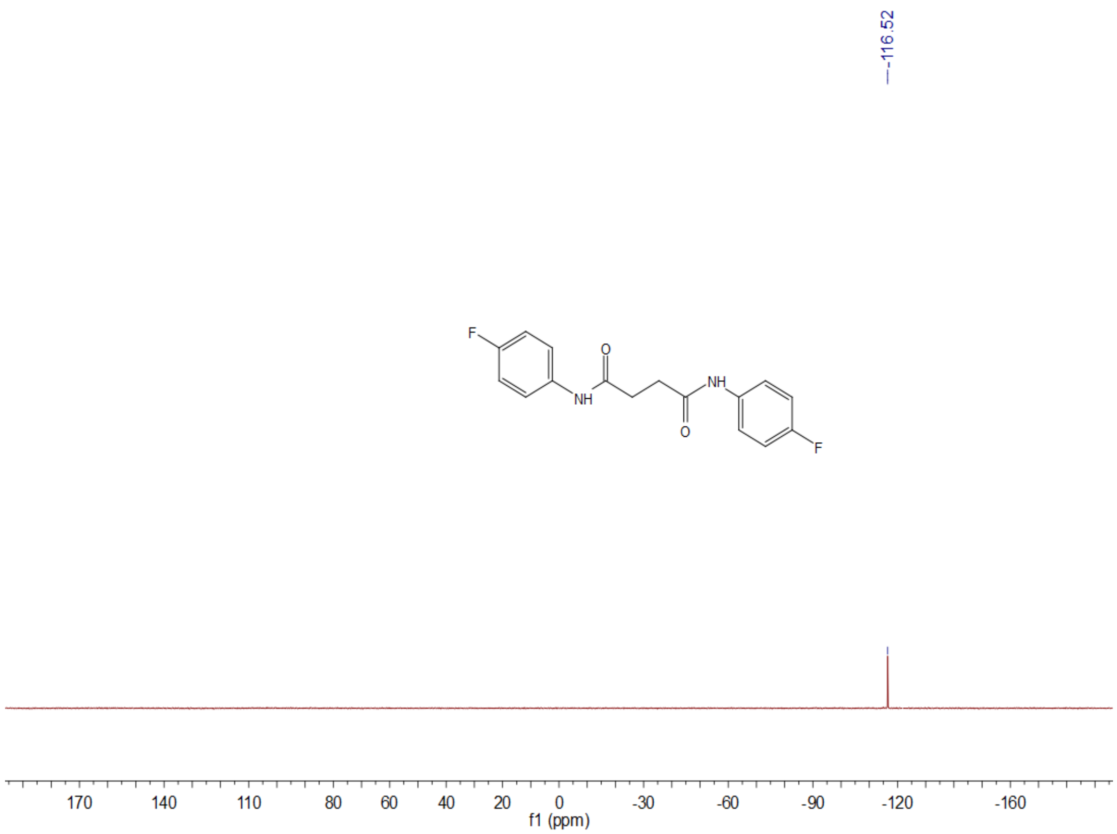
-116.52
F
O
NH
NH
O
F
170
140
110
80
60
40
20
0
-30
-60
-90
-120
-160
f1 (ppm)

***N, N'*-Bis(4-chlorophenyl)succinamide (4m)**

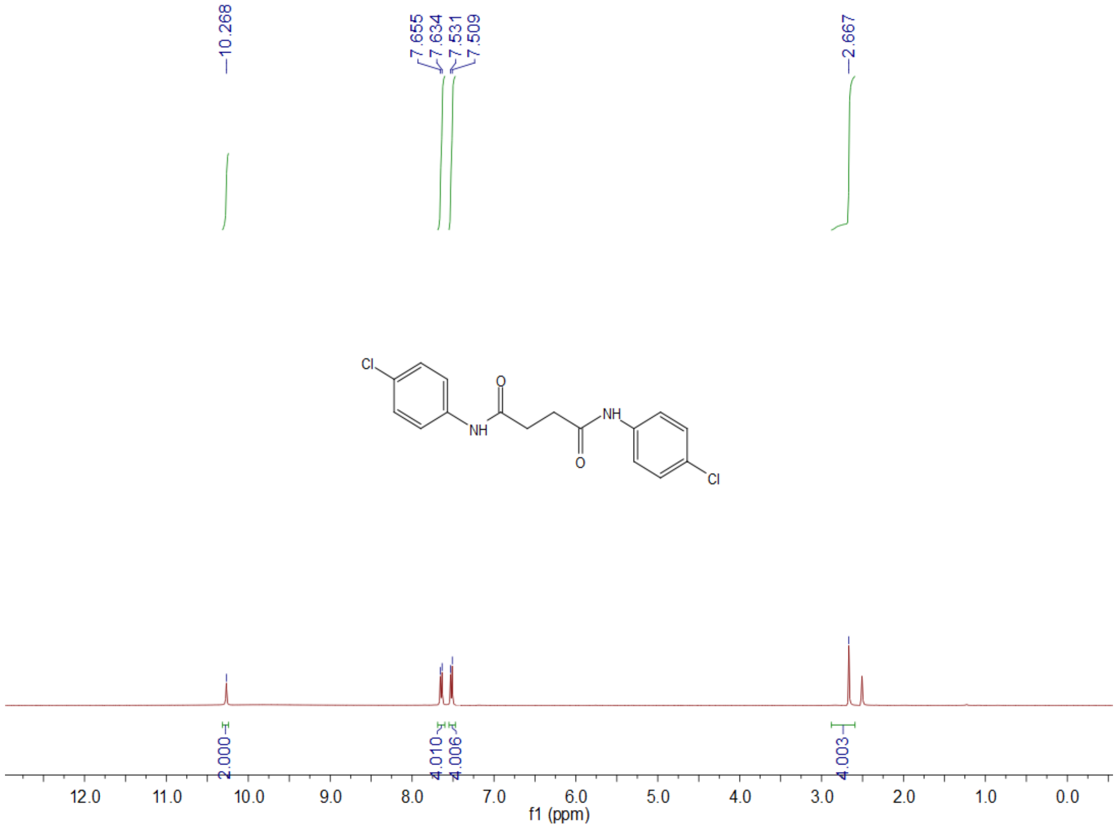

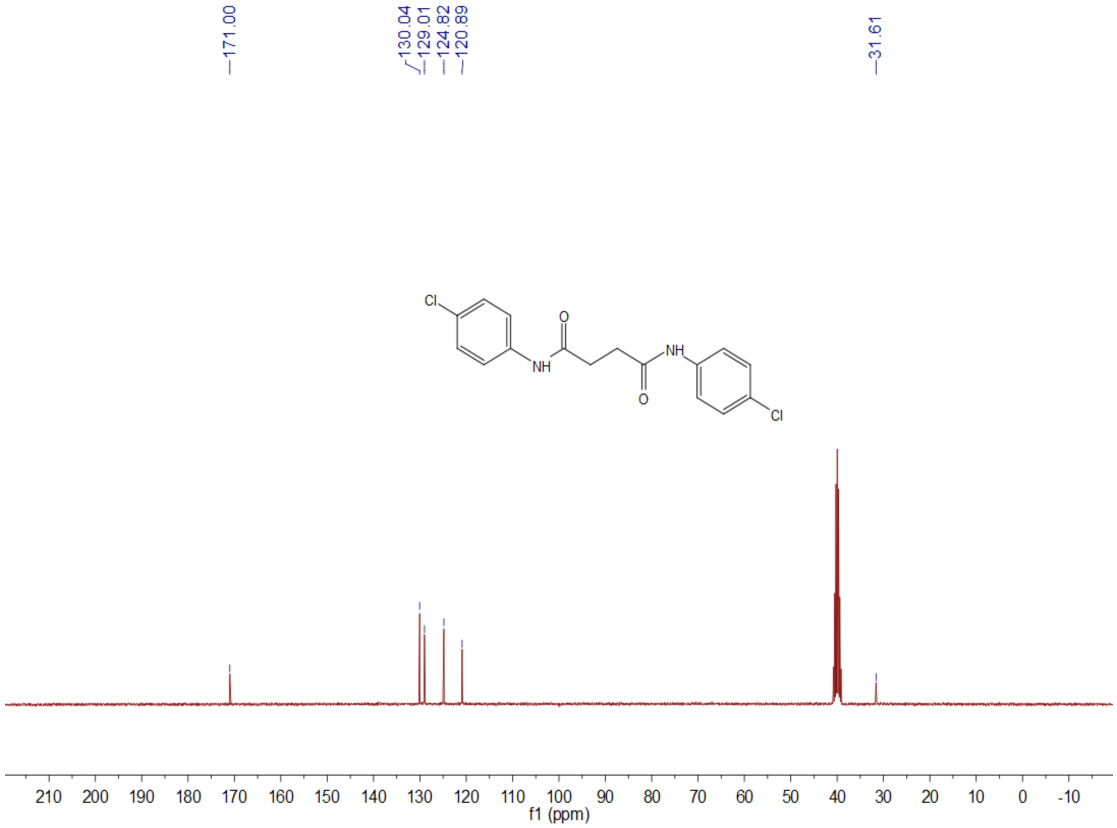

***N, N'*-Bis(4-bromophenyl)succinamide (4n)**

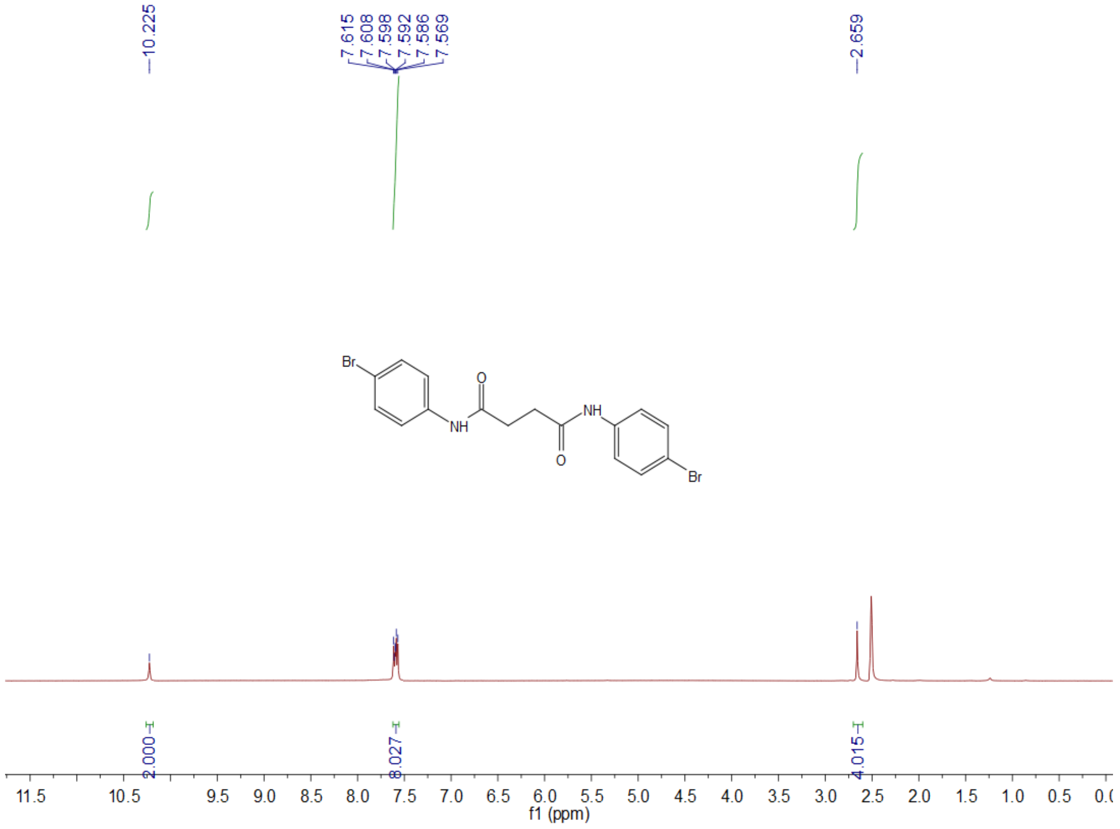

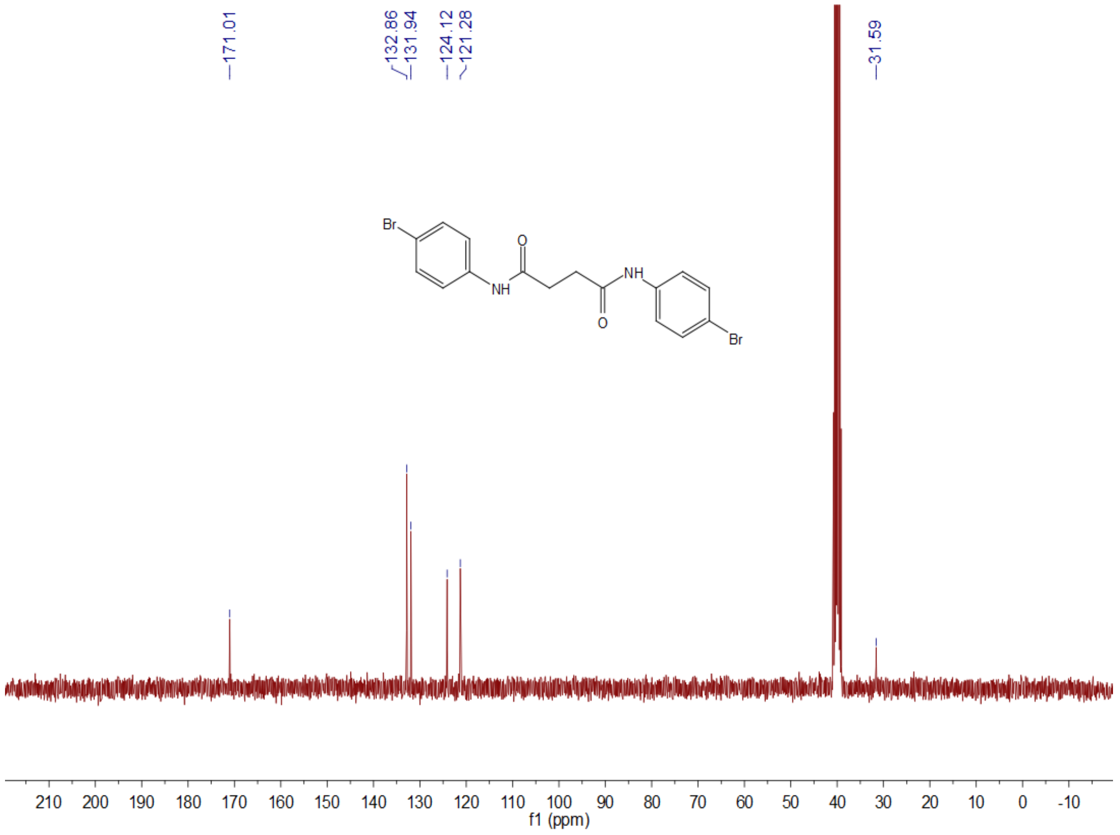

***N*, *N'*-Bis(4-iodophenyl)succinamide (4o)**

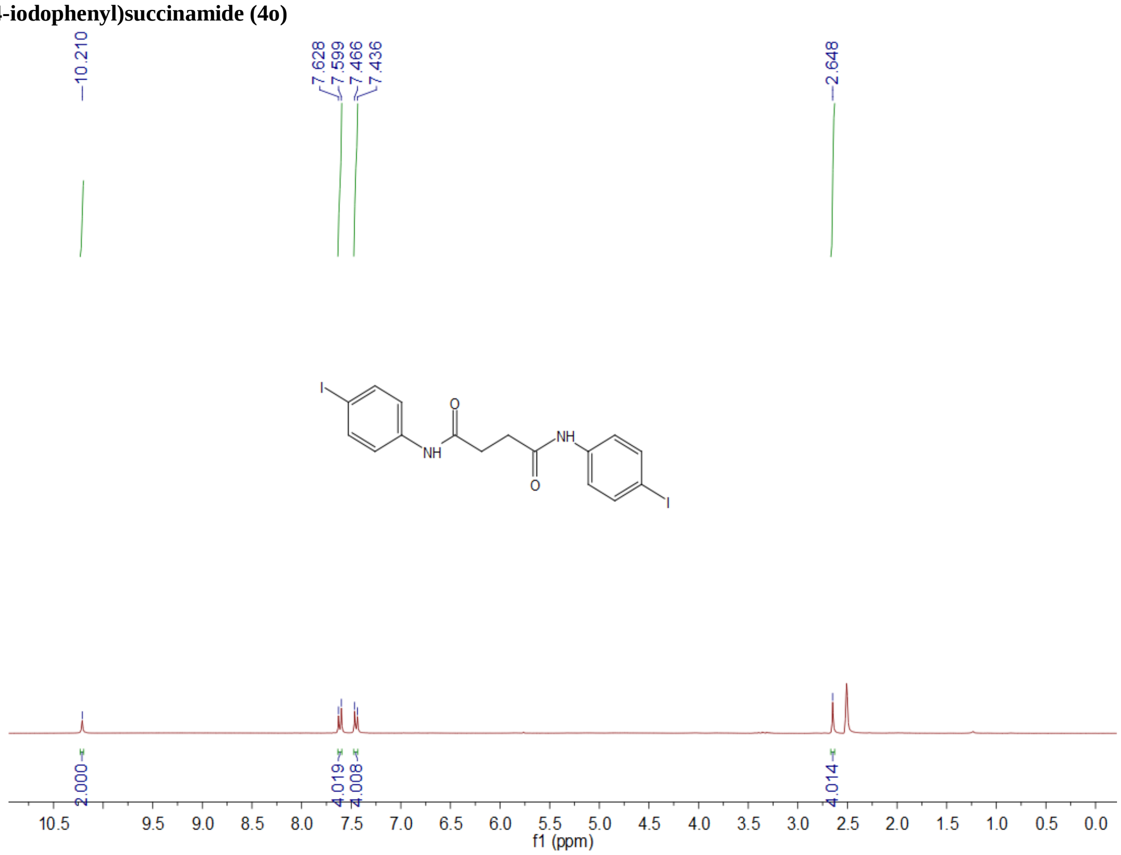

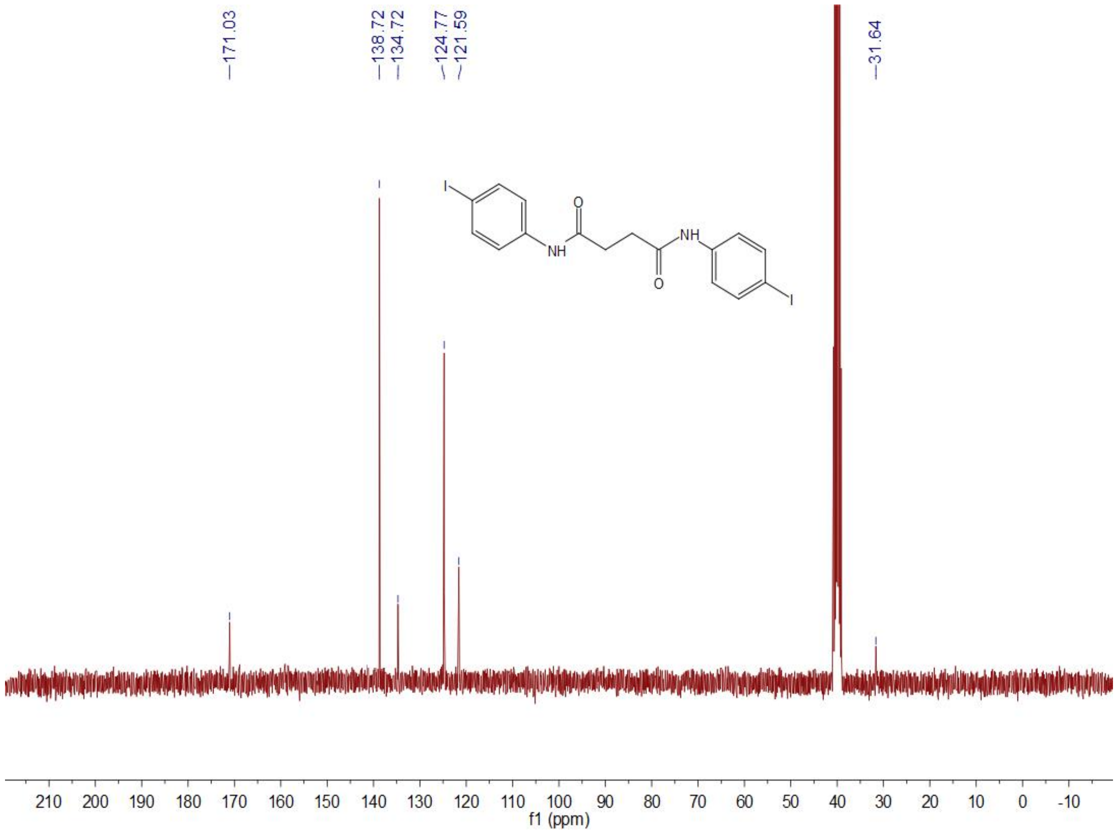

***N*, *N'*-Bis(4-(methylthio)phenyl)succinamide (4r)**

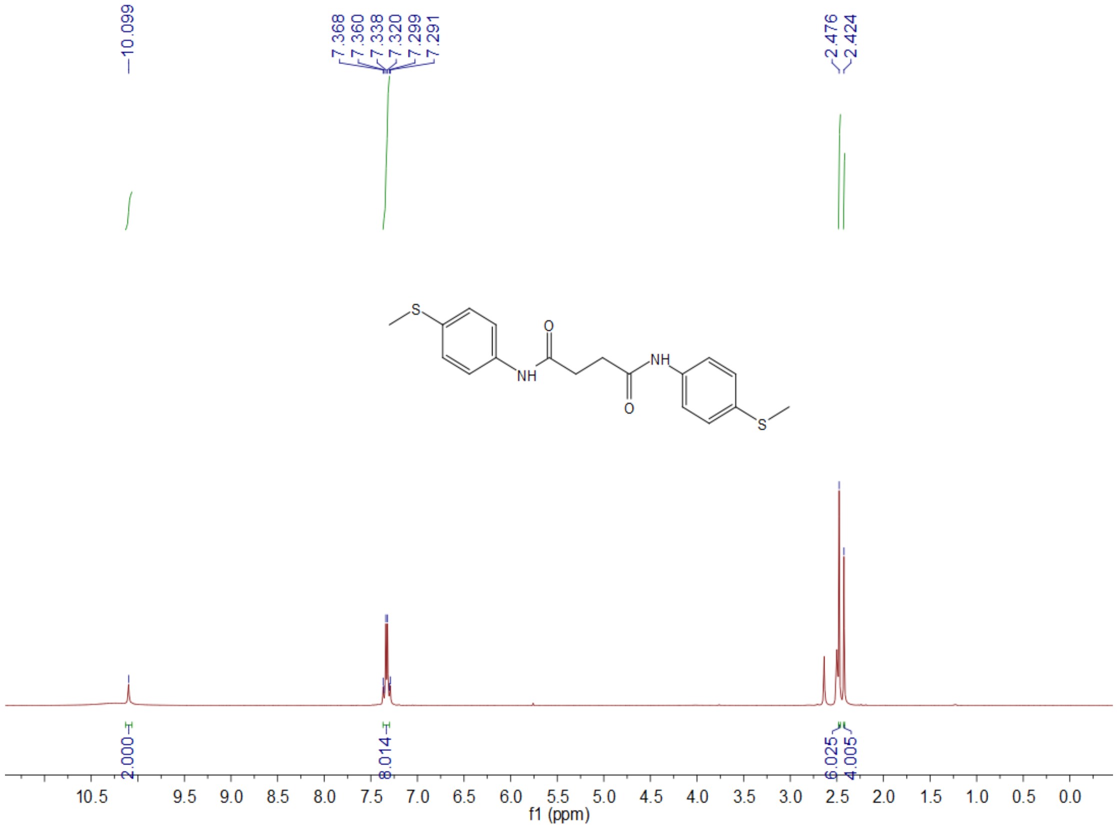

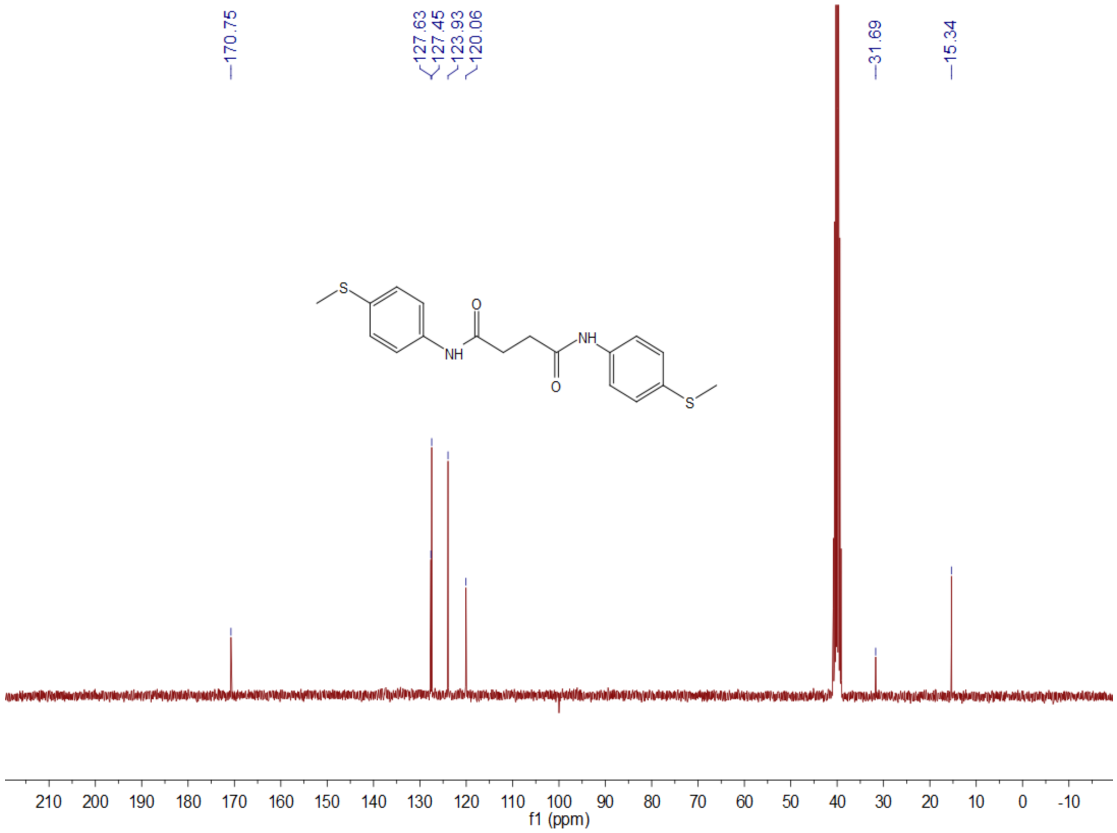

Piperidine-2,6-dione (5a)

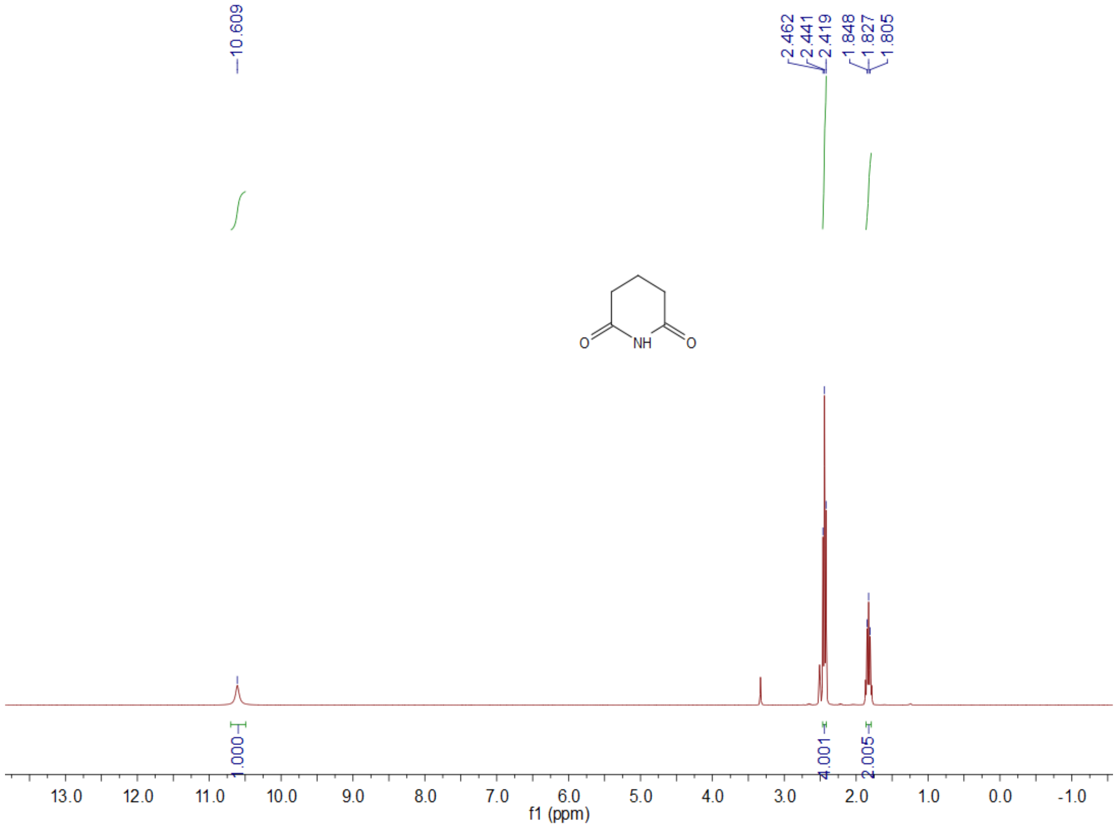

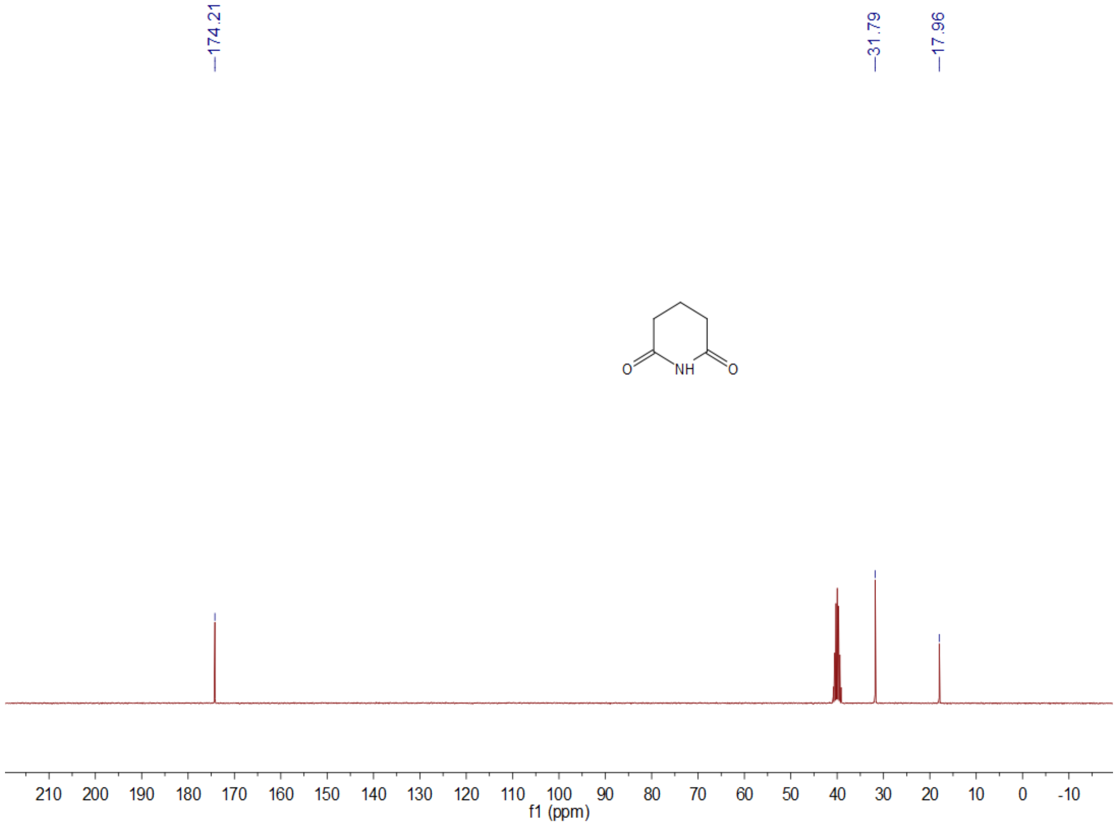

1-Methylpiperidine-2,6-dione (5b)

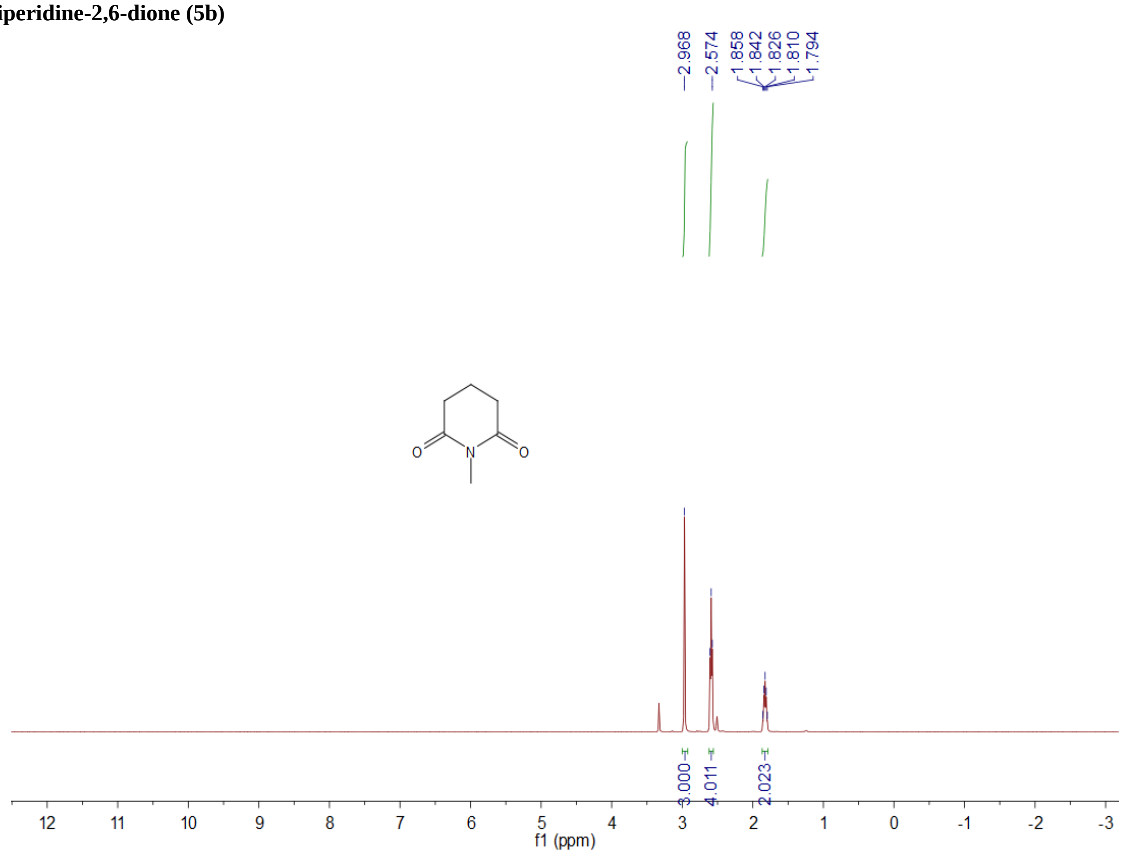

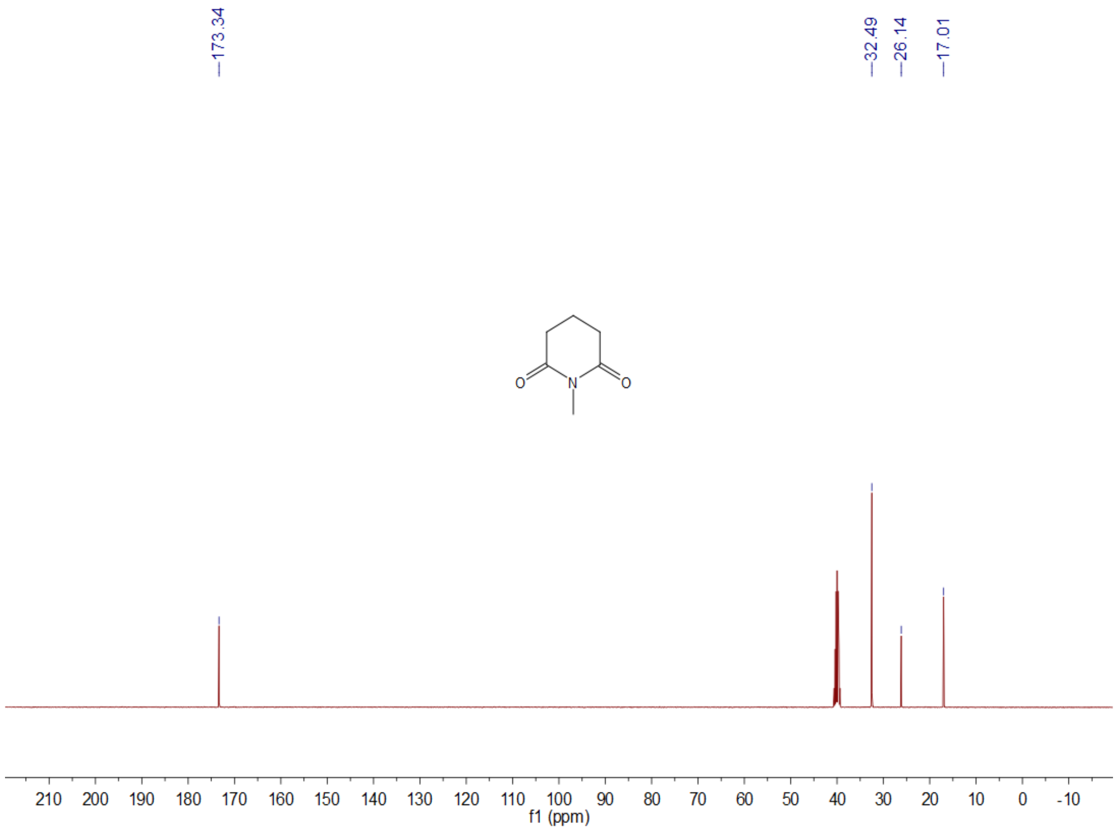

1-Butylpiperidine-2,6-dione (5c)

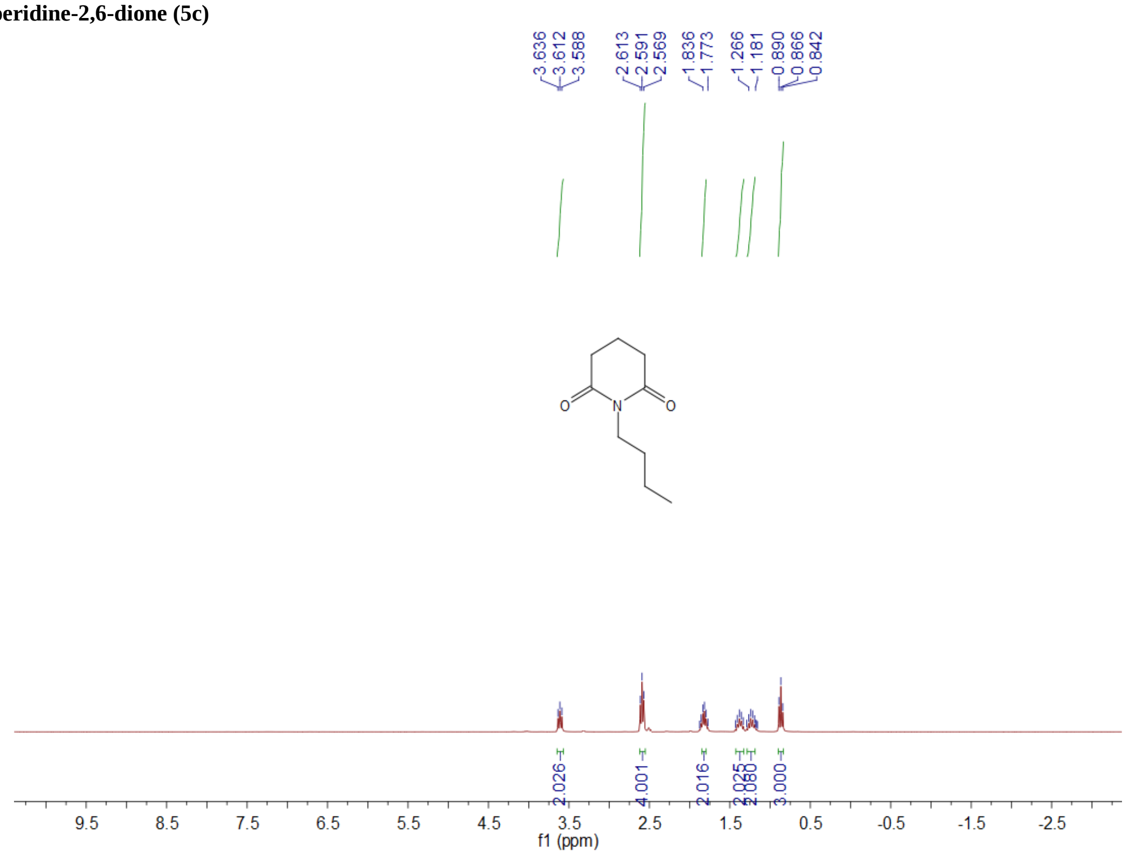

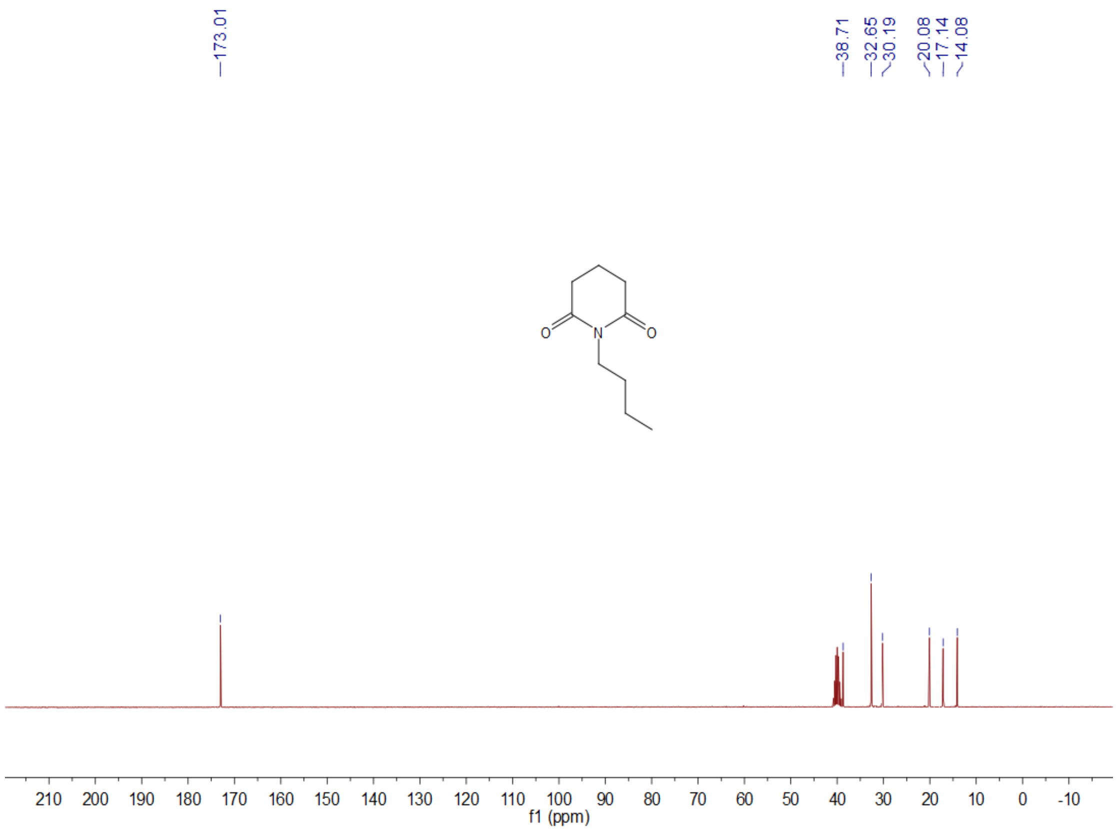

1-Benzylpiperidine-2,6-dione (5d)

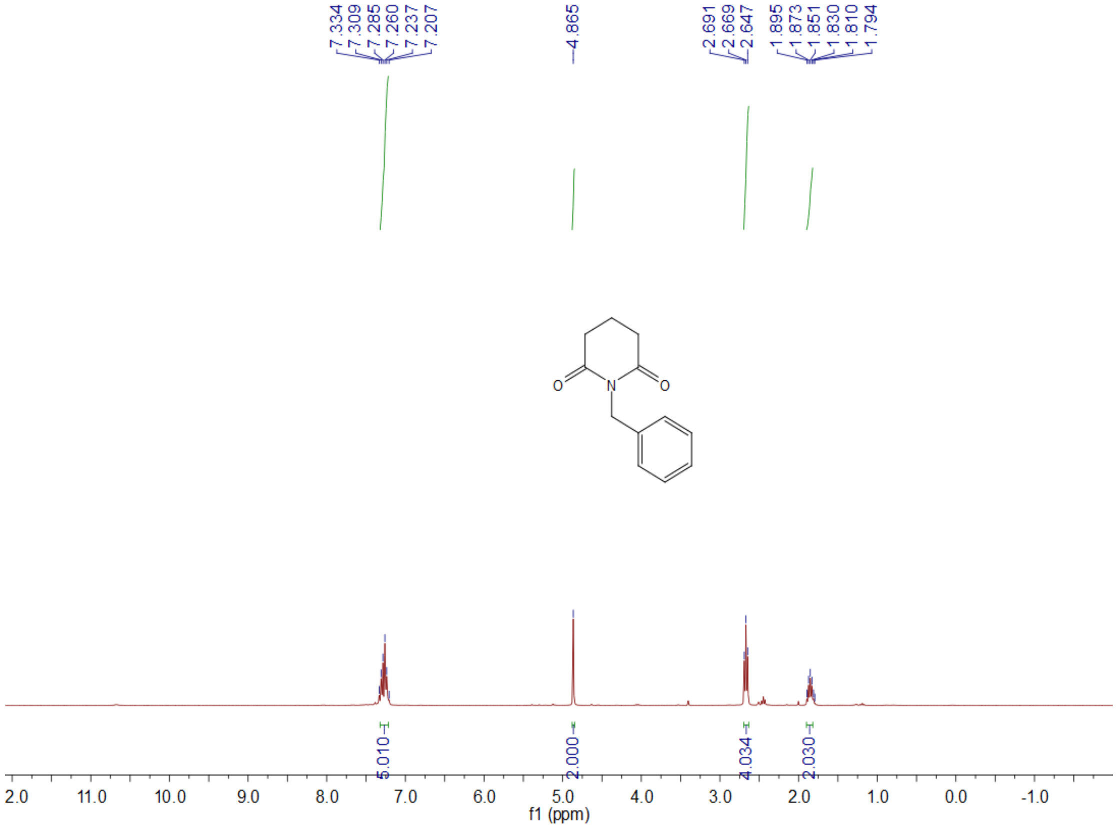

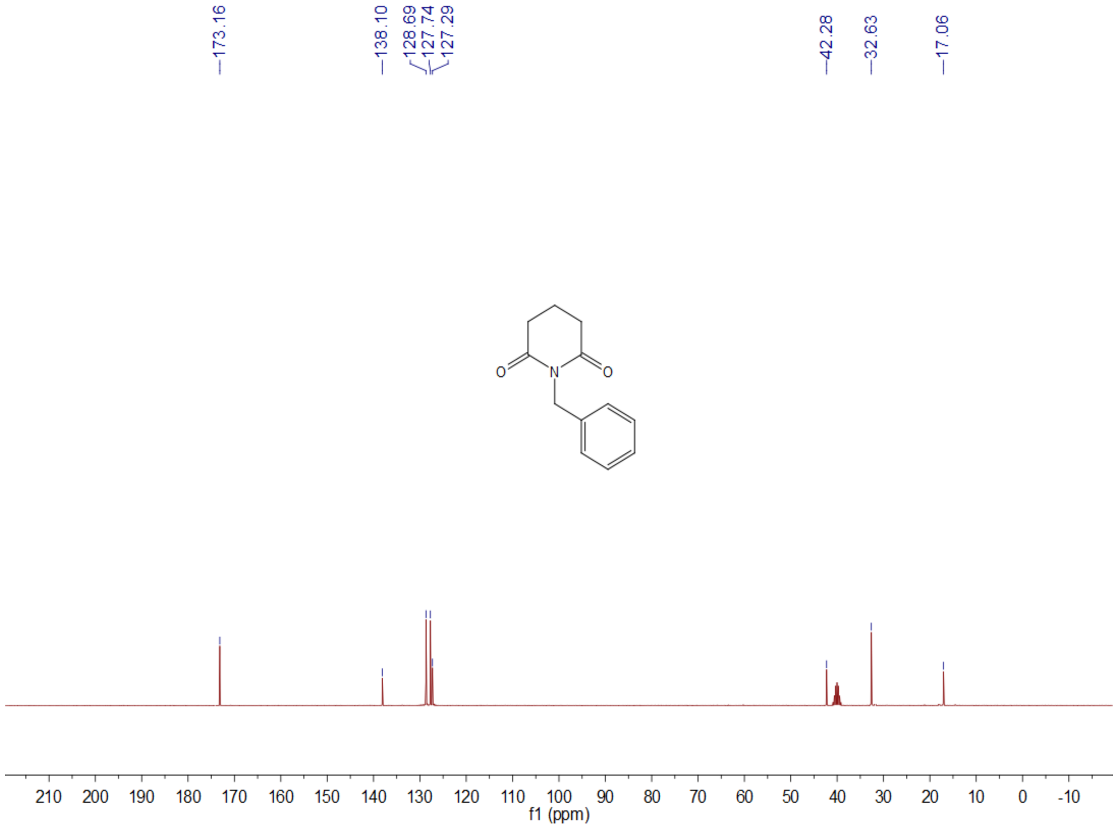

1-Phenylpiperidine-2,6-dione (5e)

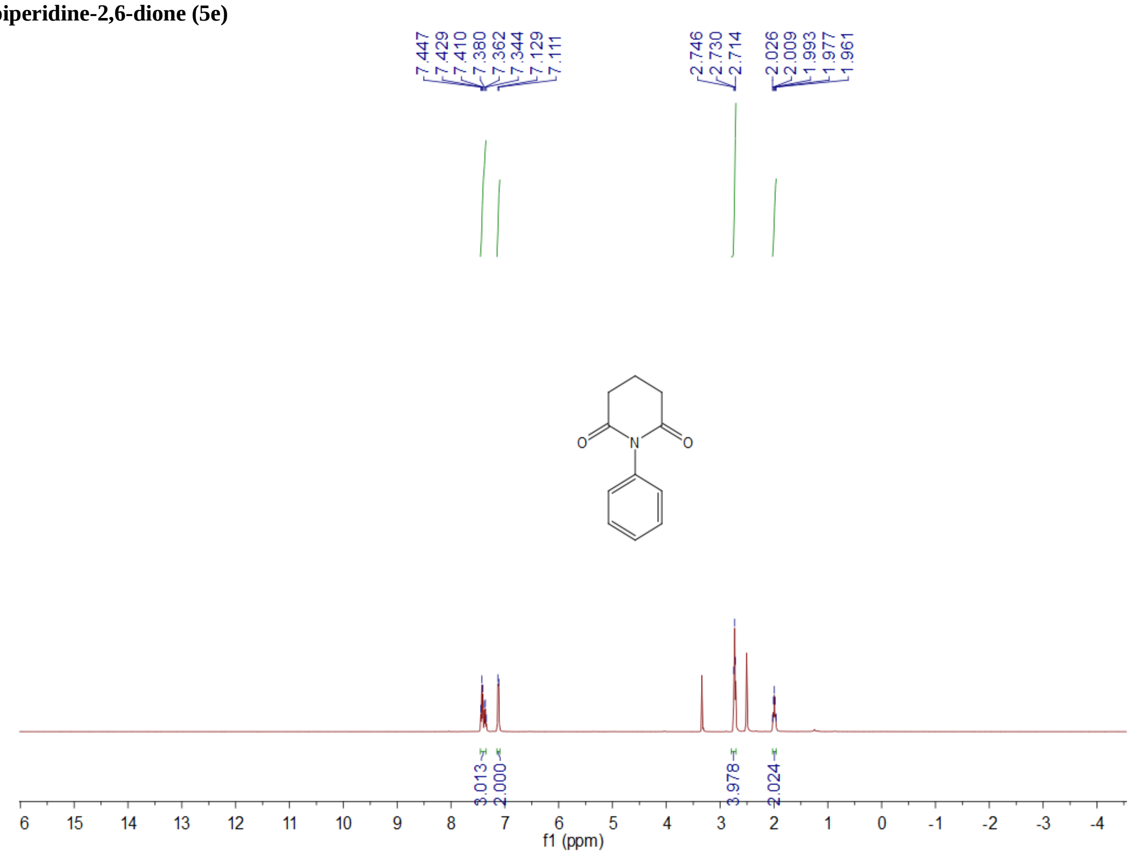

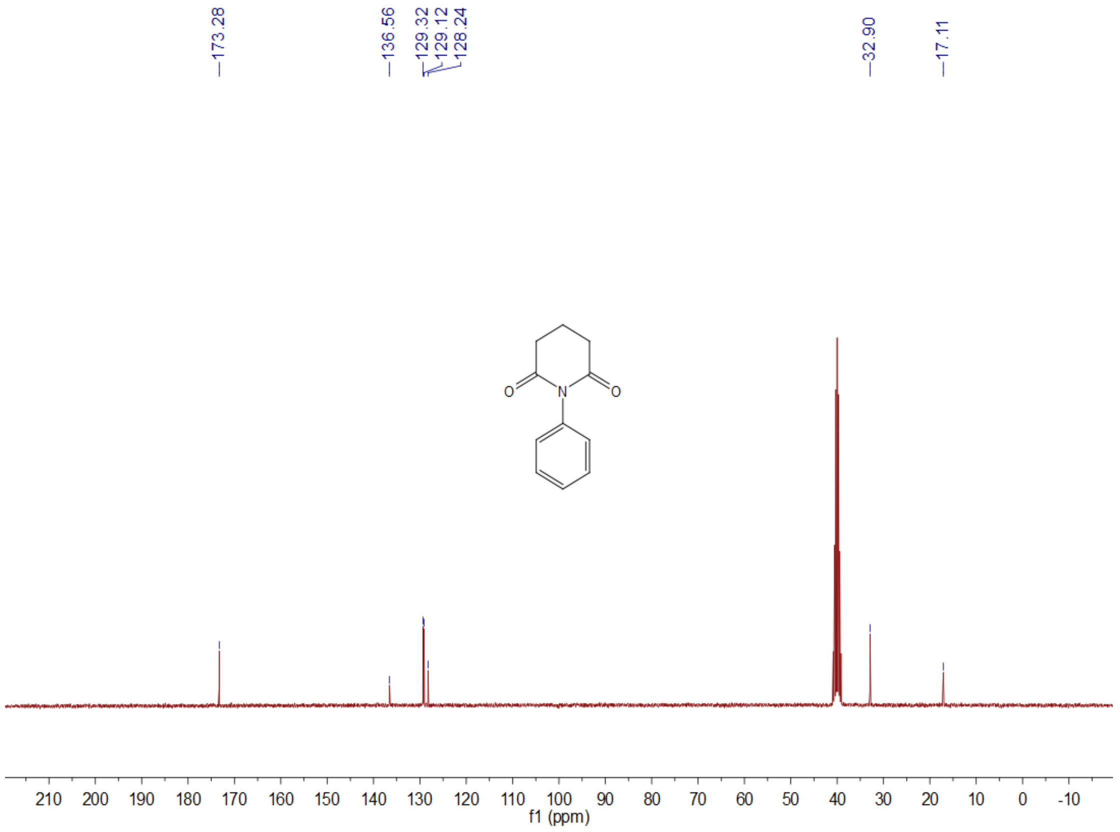

1-(*p*-Tolyl)piperidine-2,6-dione (5f)

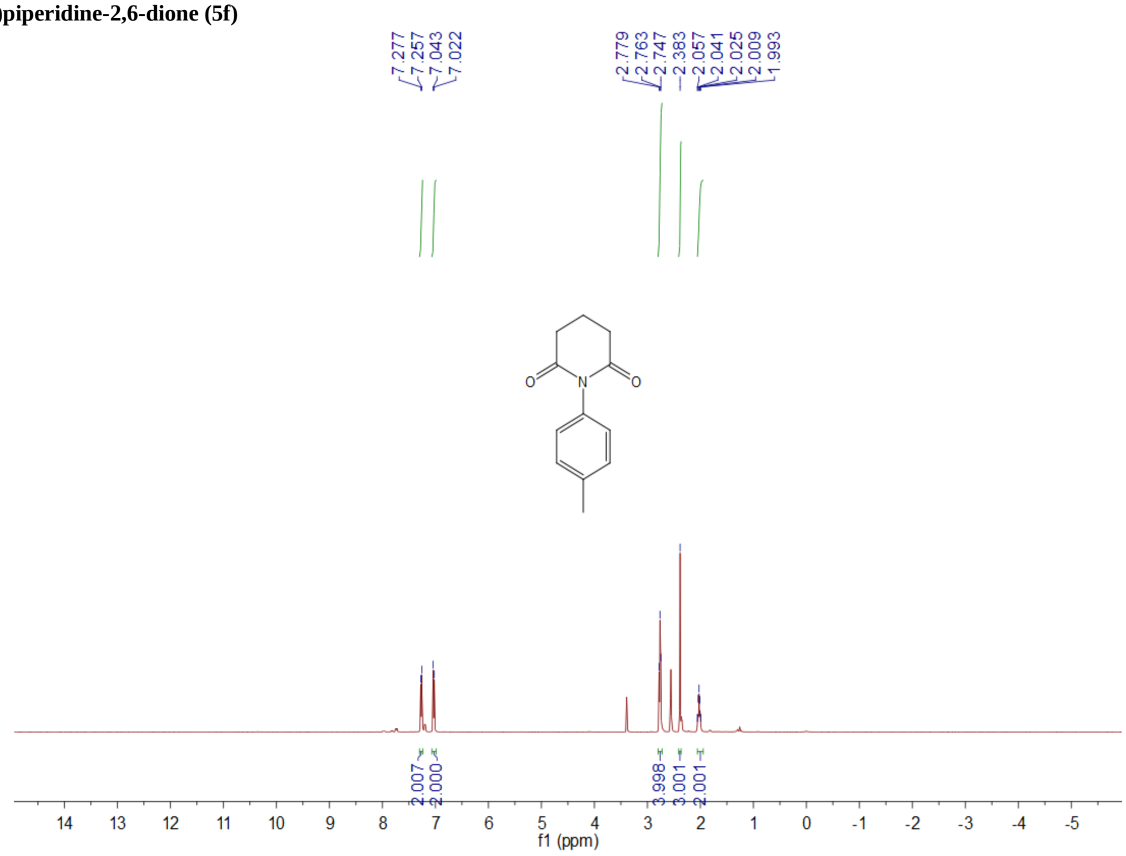

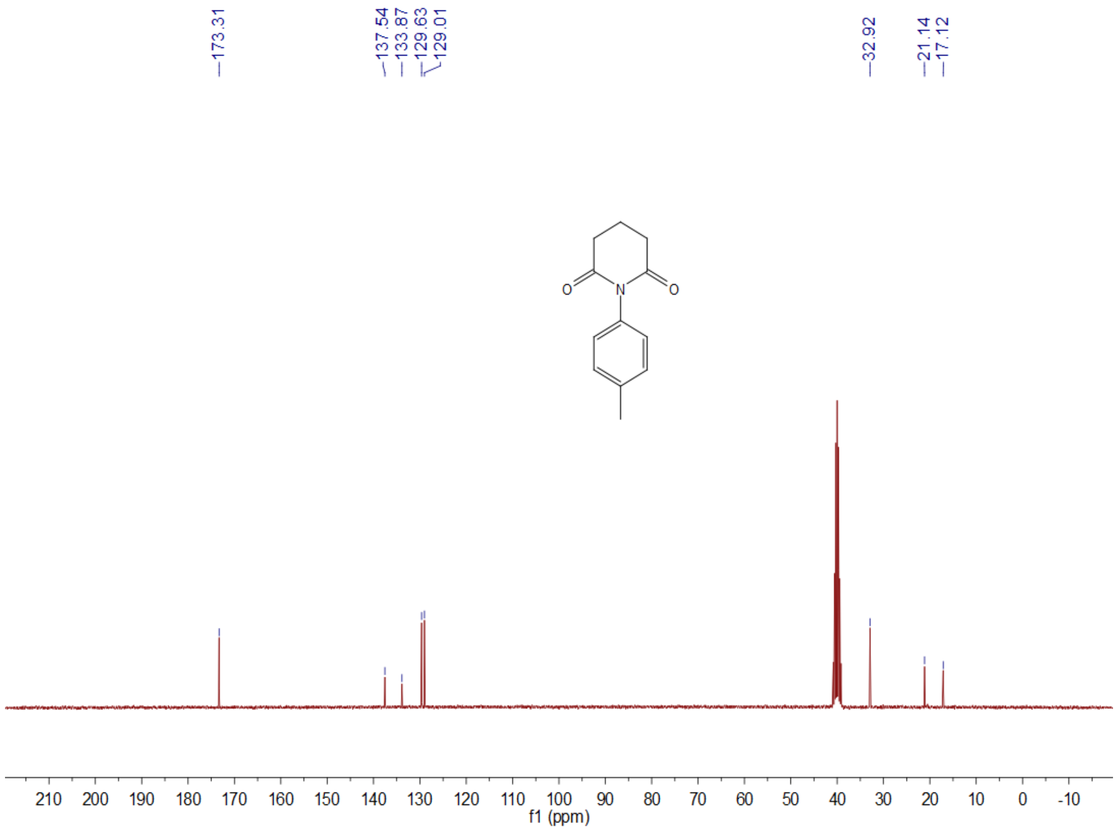

1-(*m*-Tolyl)piperidine-2,6-dione (5g)

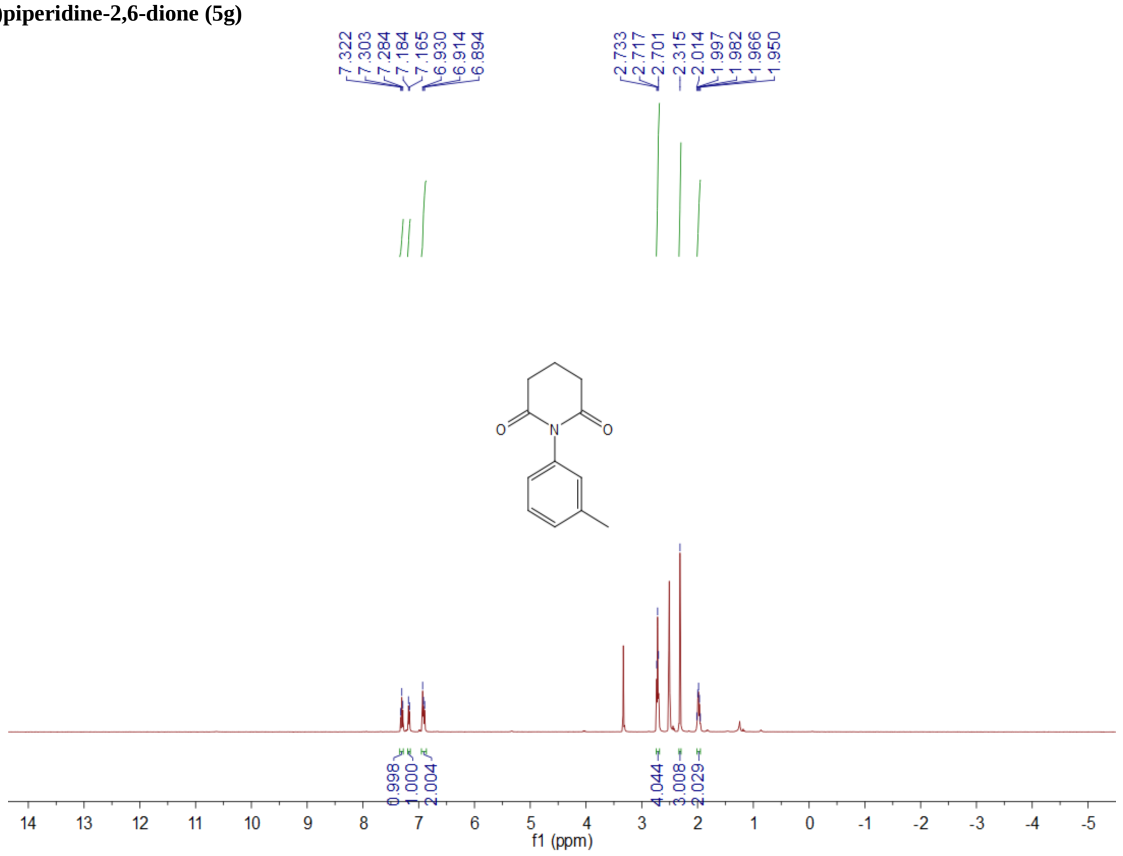

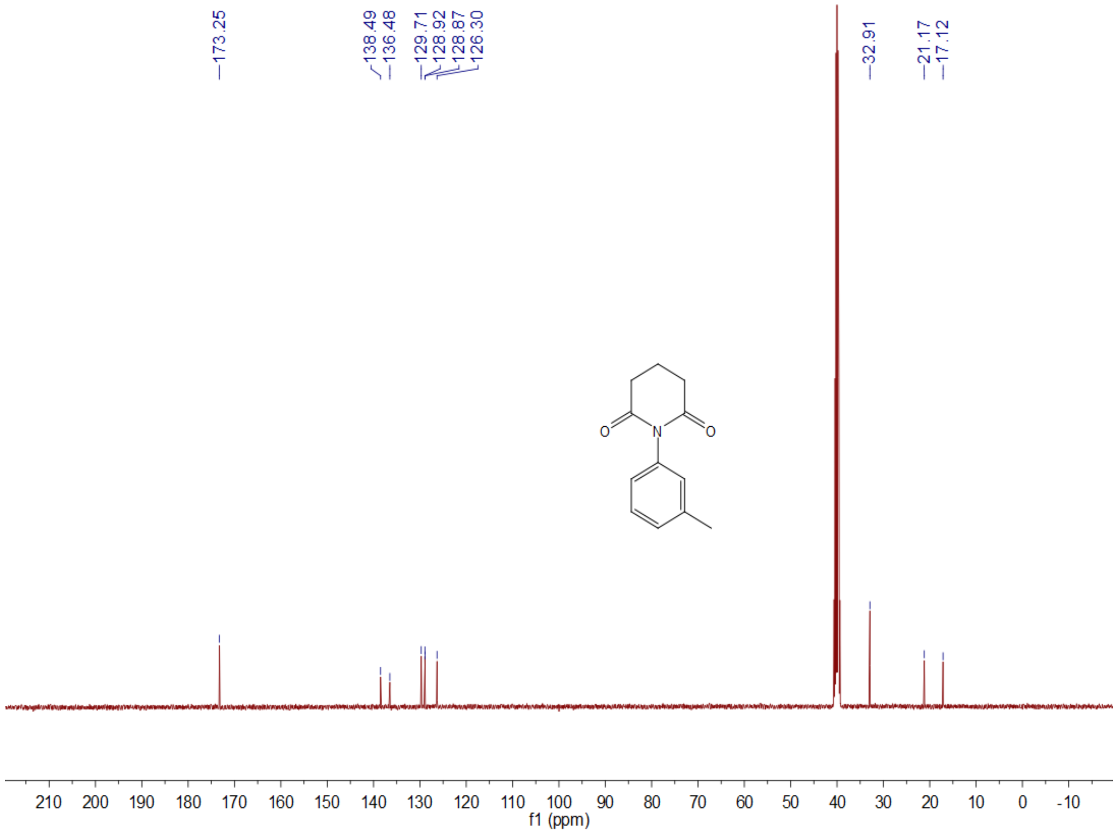

1-(4-Methoxyphenyl)piperidine-2,6-dione (5h)

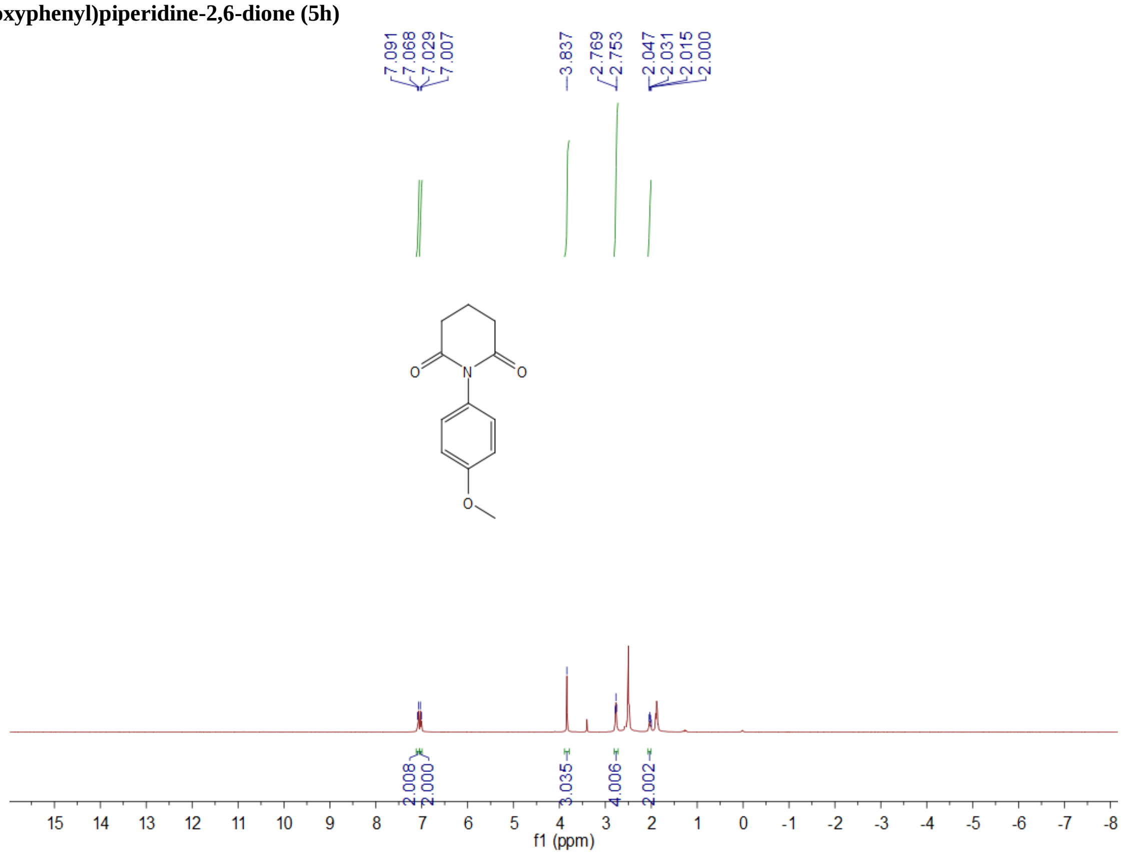

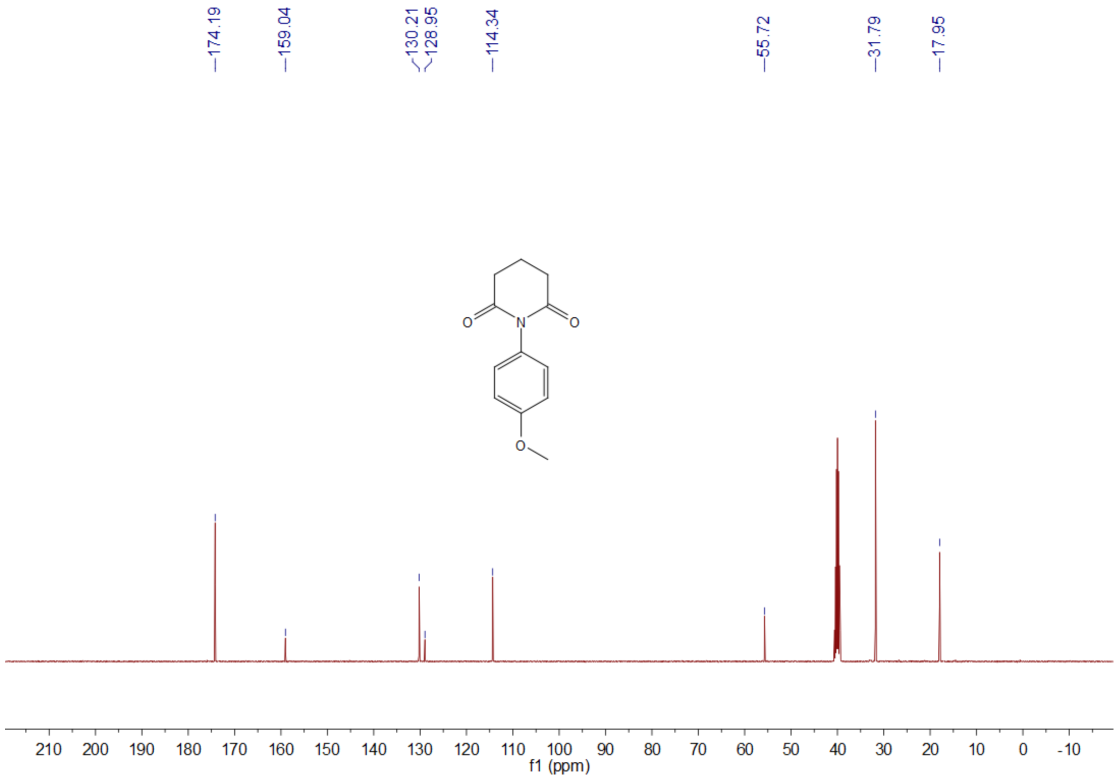

1-(4-Fluorophenyl)piperidine-2,6-dione (5i)

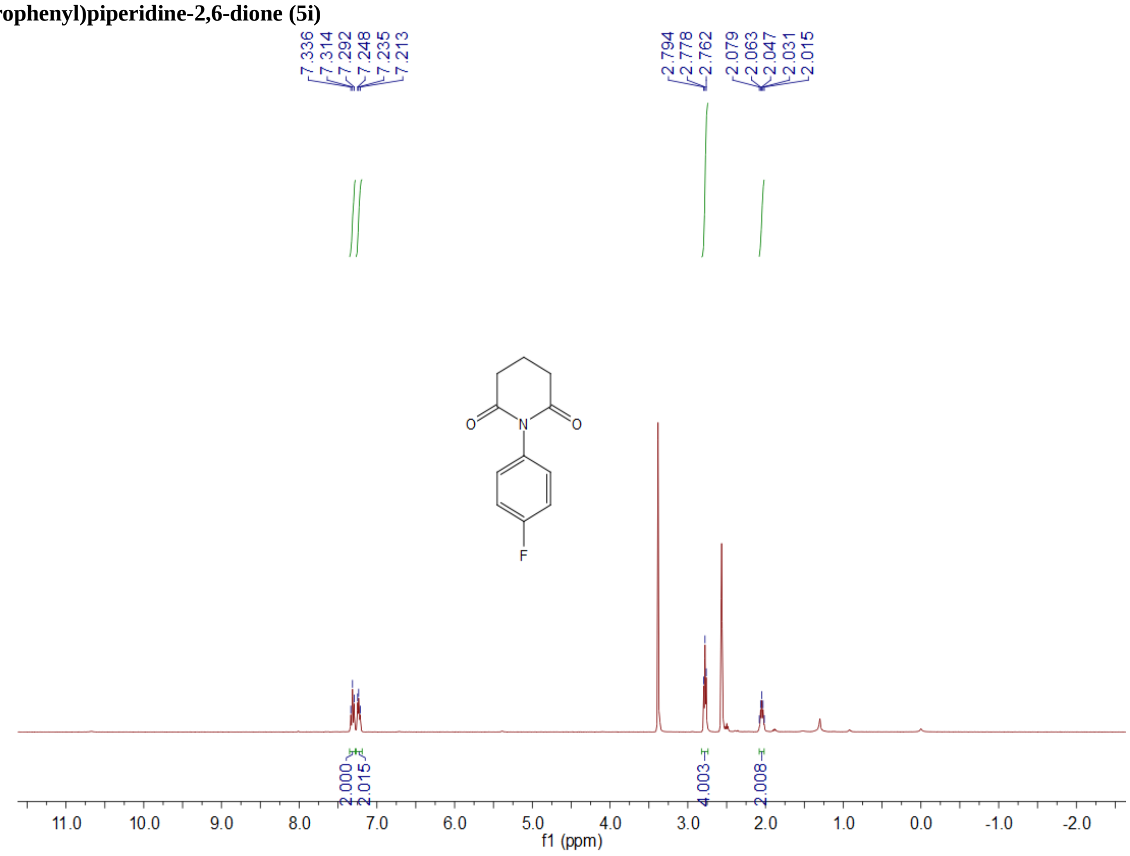

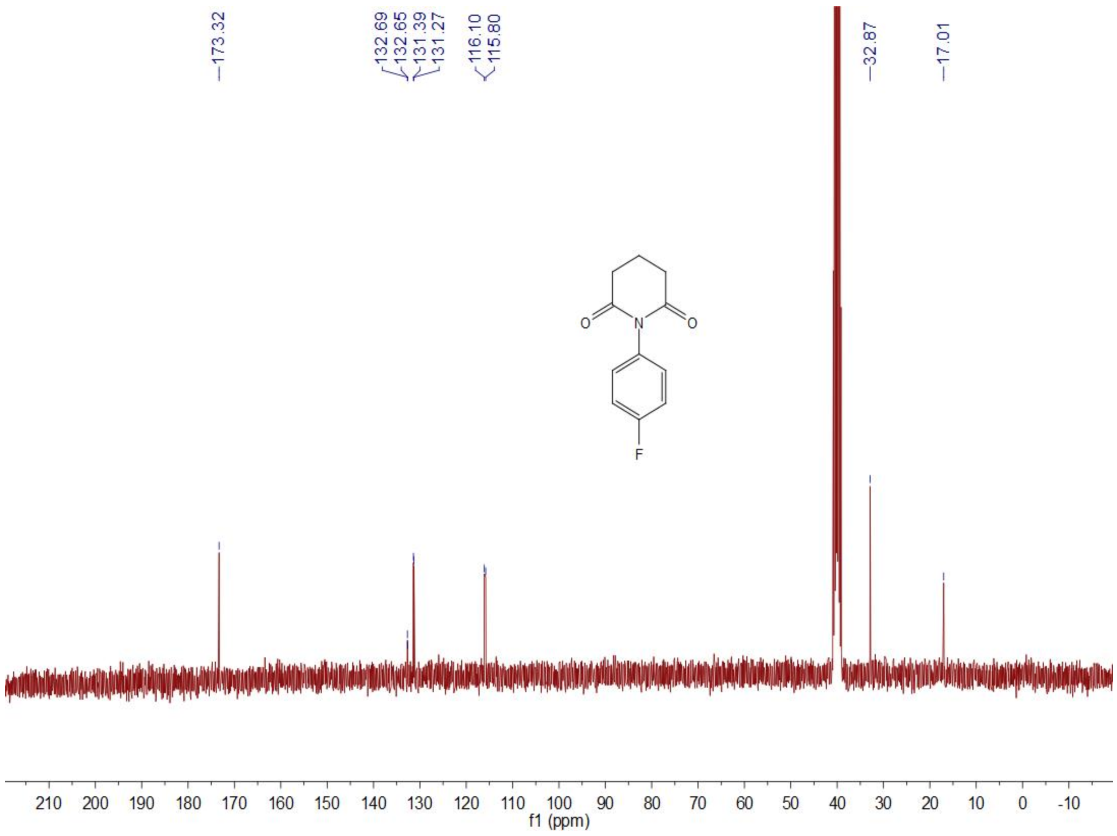

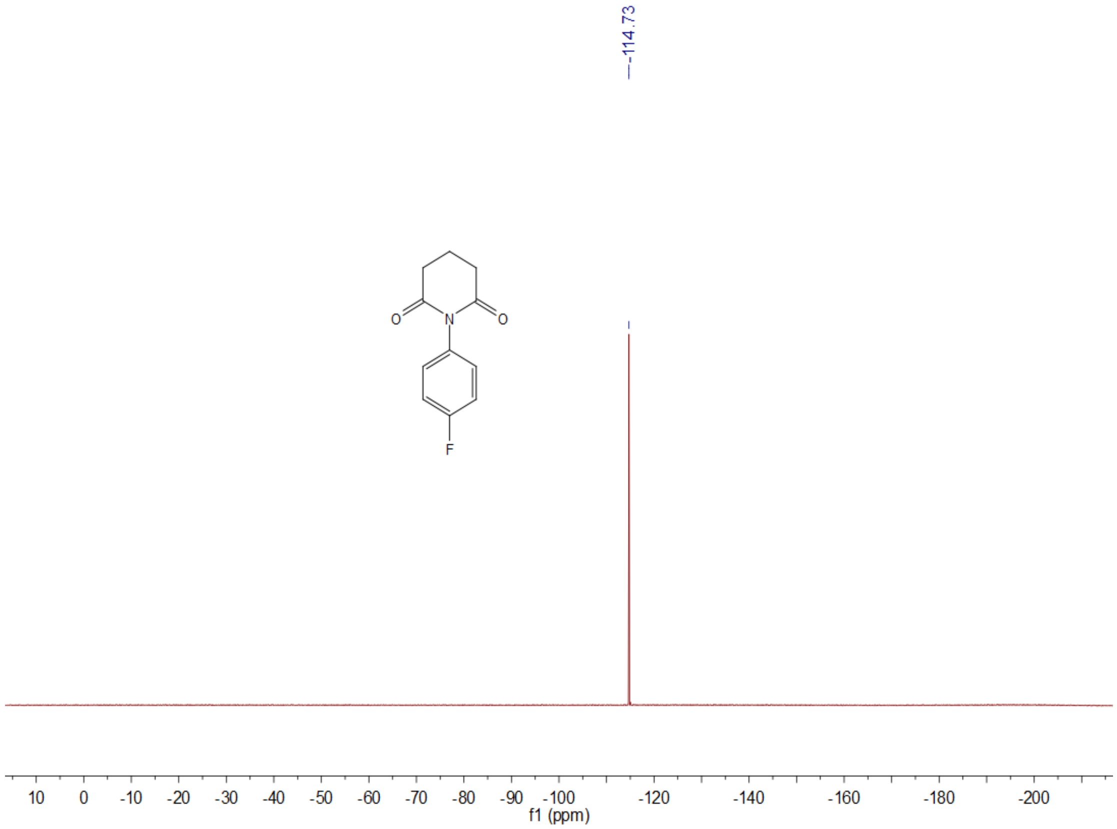
-114.73
O
N
O
F
10 0 -10 -20 -30 -40 -50 -60 -70 -80 -90 -100 -120 -140 -160 -180 -200
f1 (ppm)

1-(4-Chlorophenyl)piperidine-2,6-dione (5j)

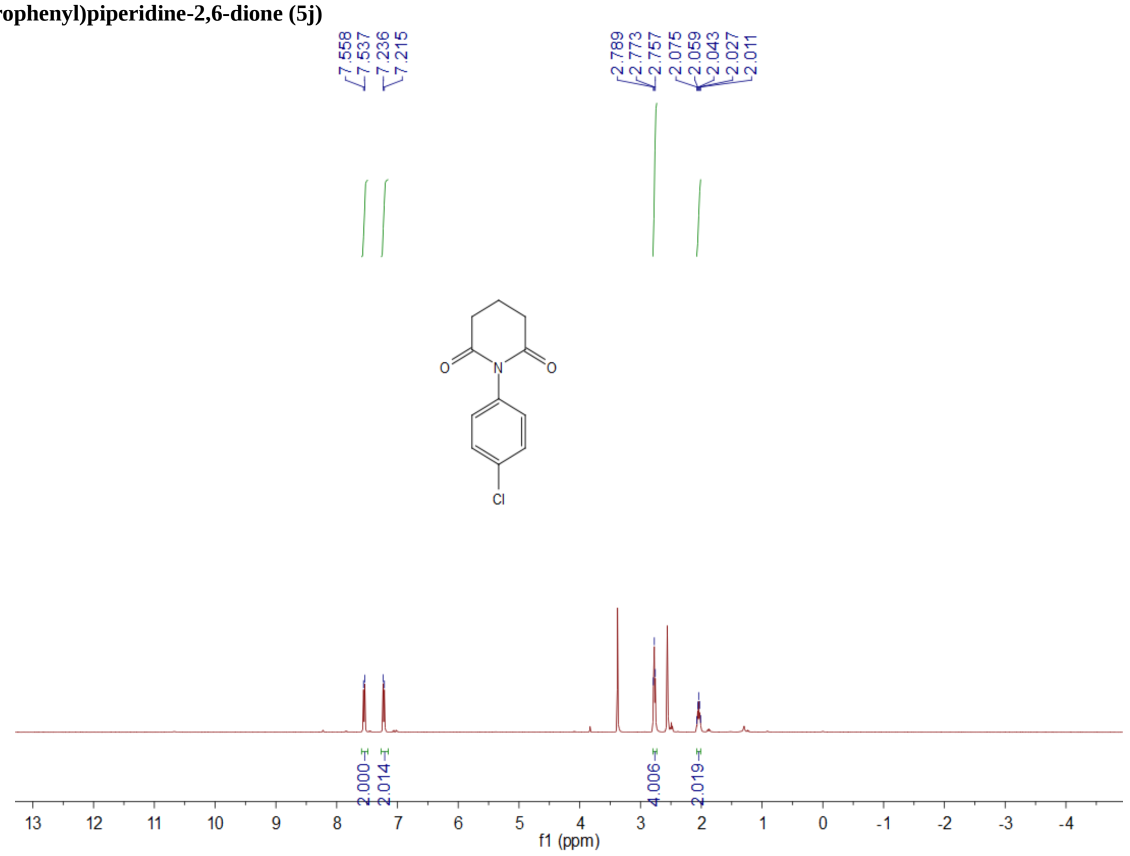

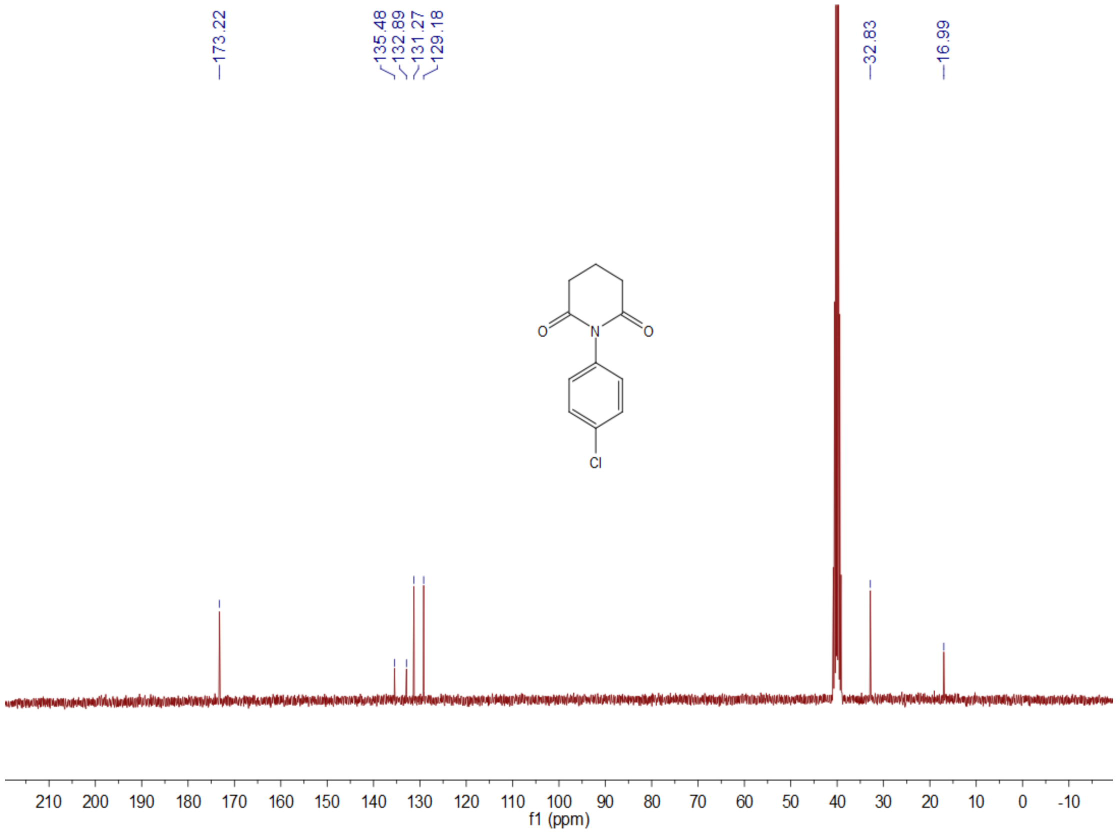

1-(4-Bromophenyl)piperidine-2,6-dione (5k)

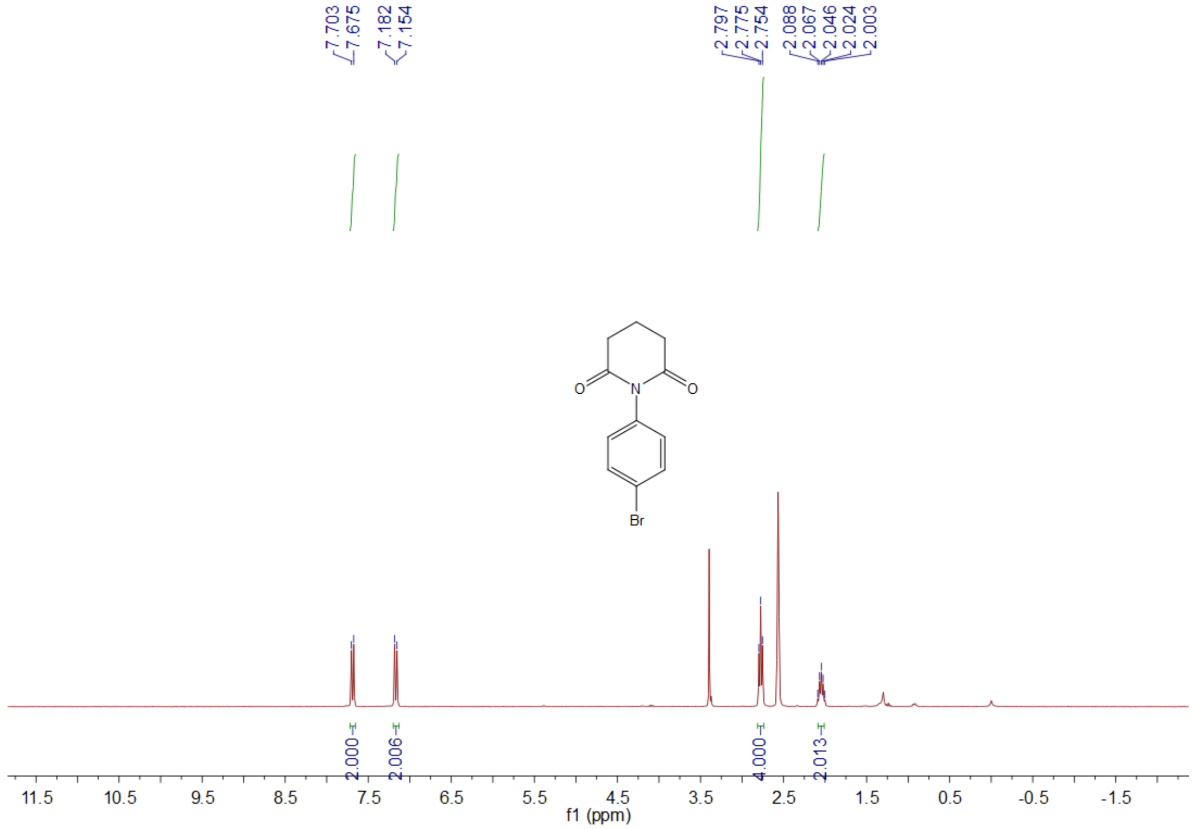

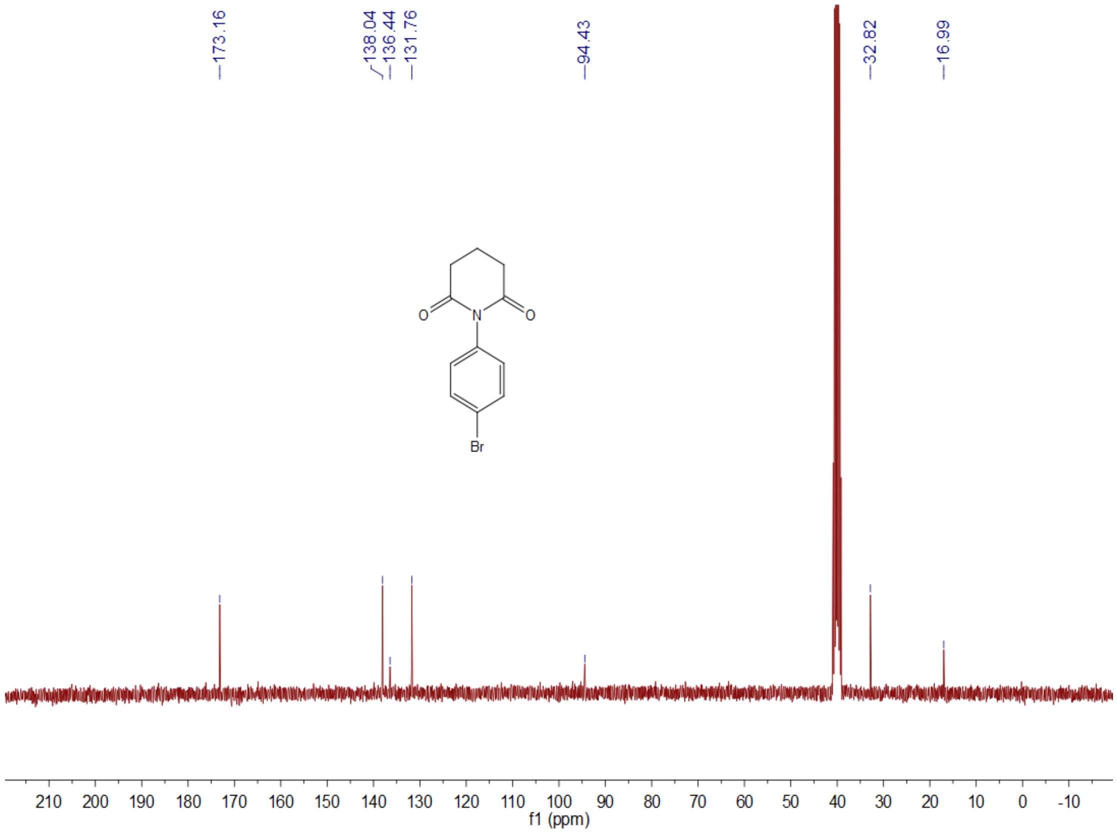

1-(4-Iodophenyl)piperidine-2,6-dione (5l)

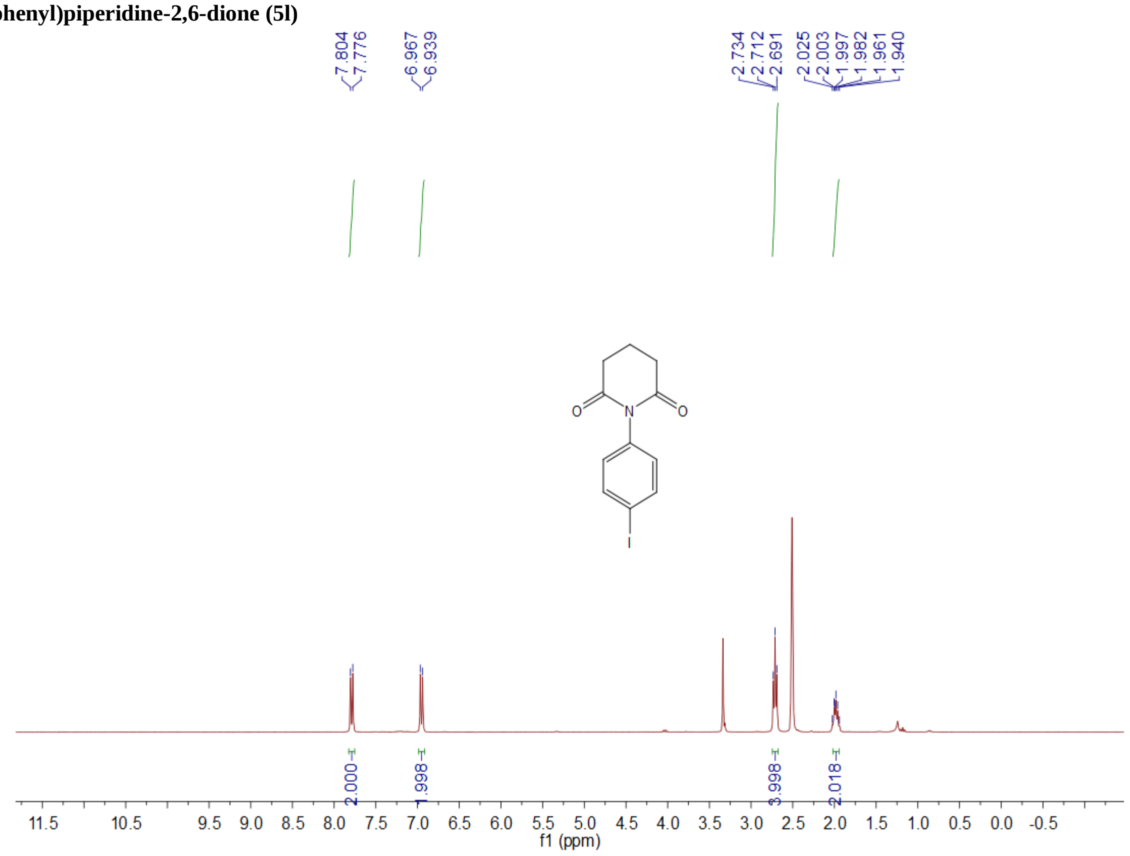

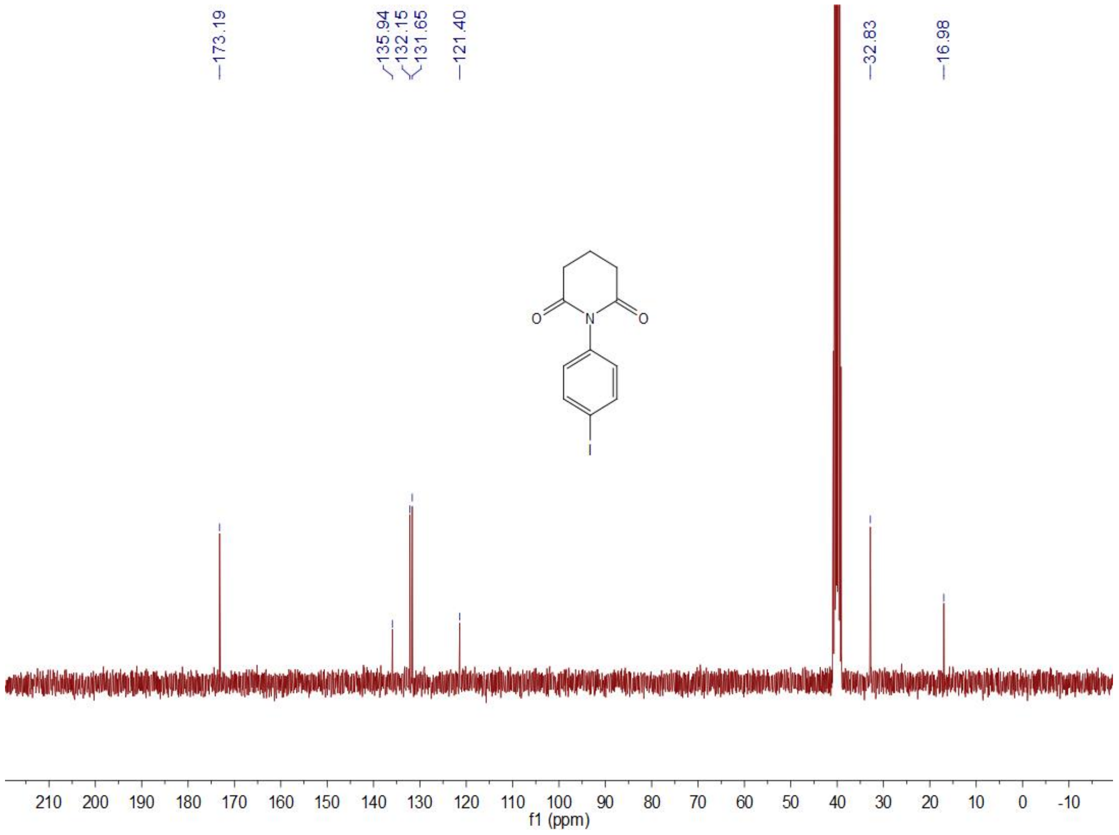

1-(4-(Methylthio)phenyl)piperidine-2,6-dione (5o)

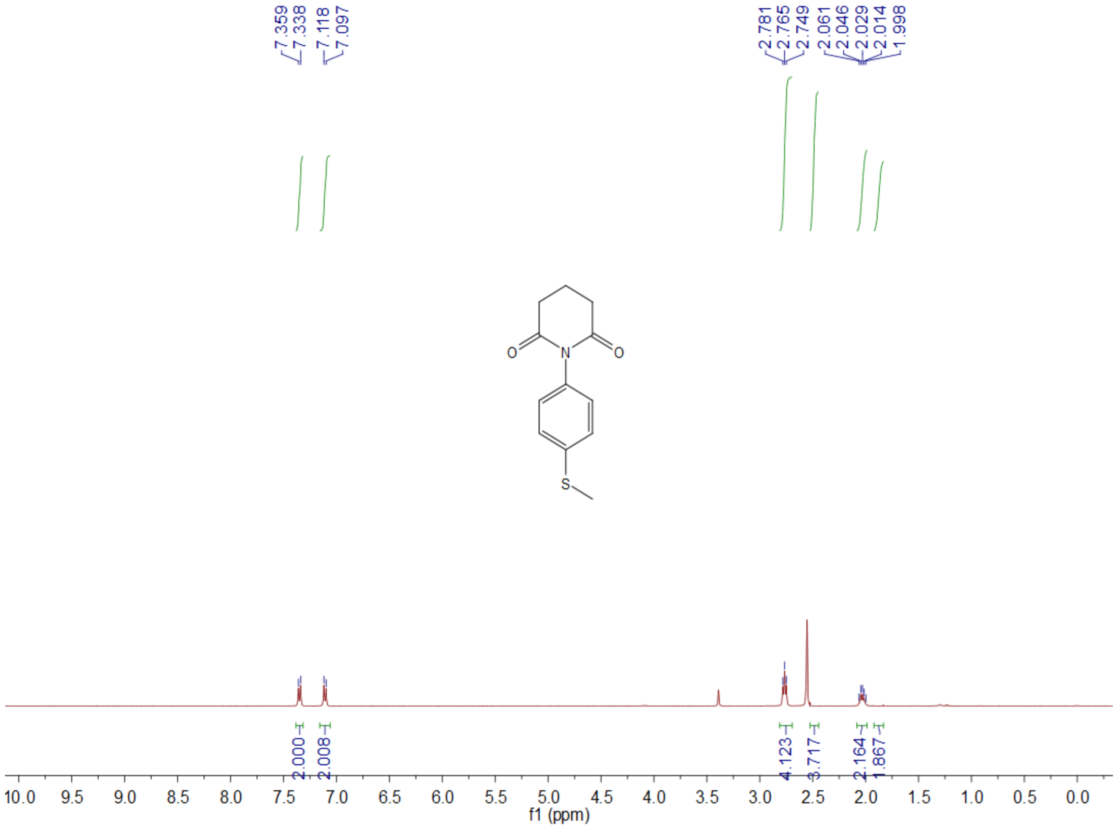

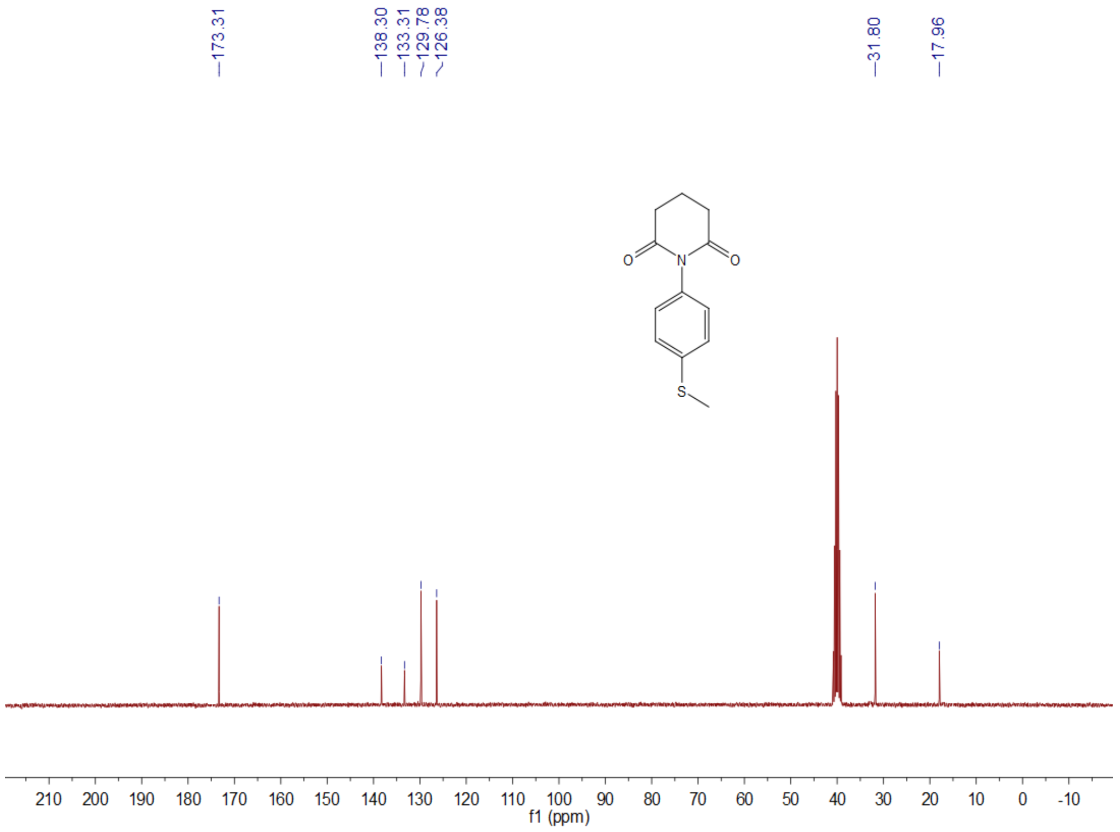

Glutaramide (6a)

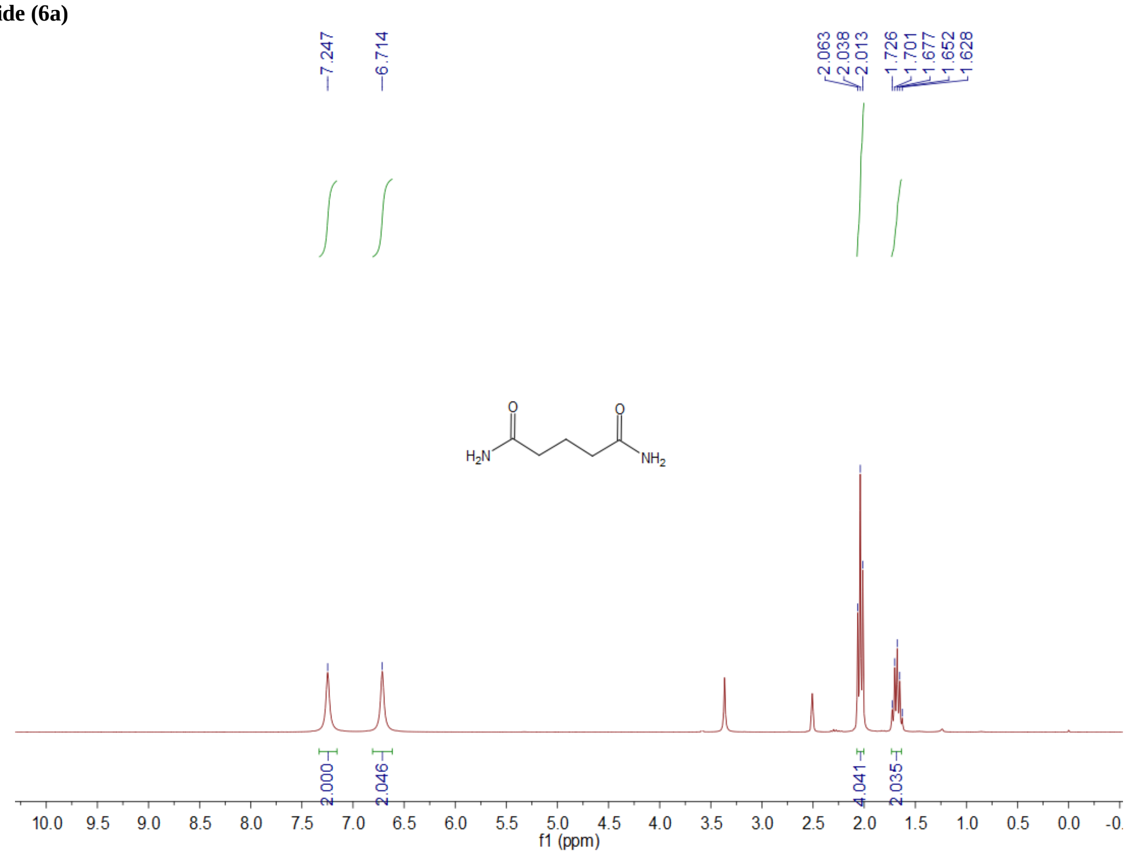

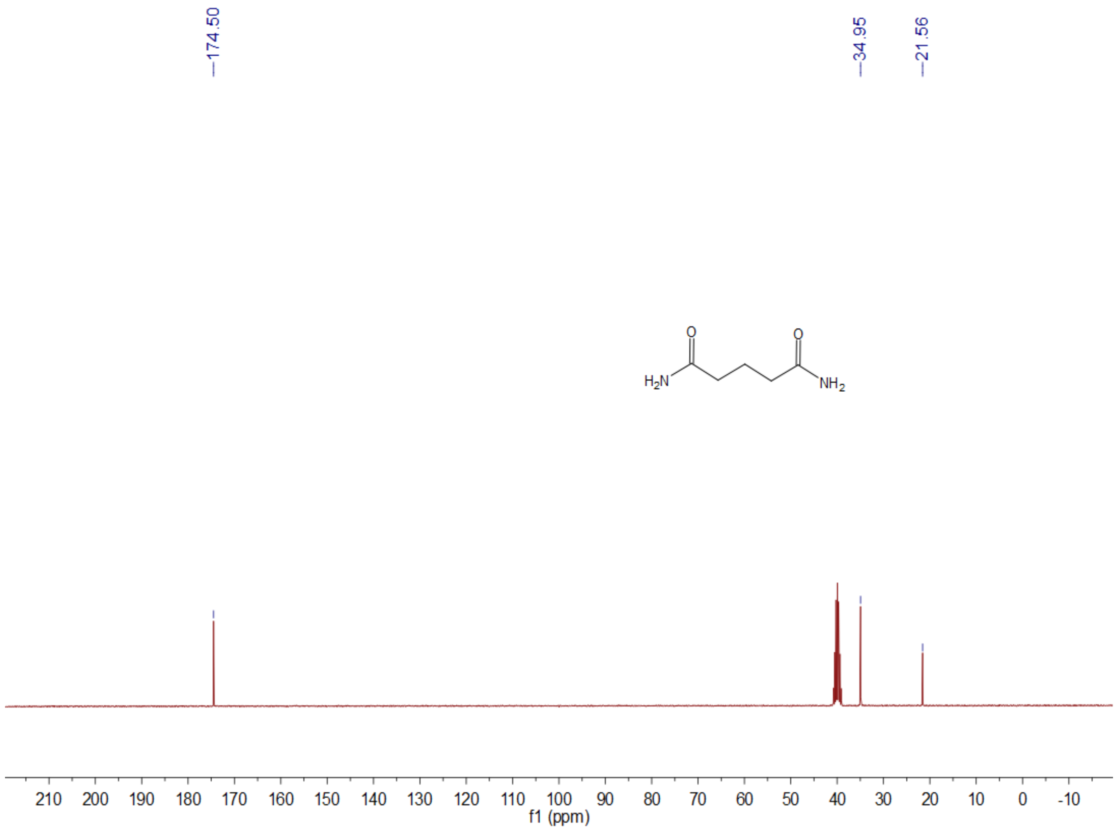

***N, N'*-Dimethylglutaramide (6b)**

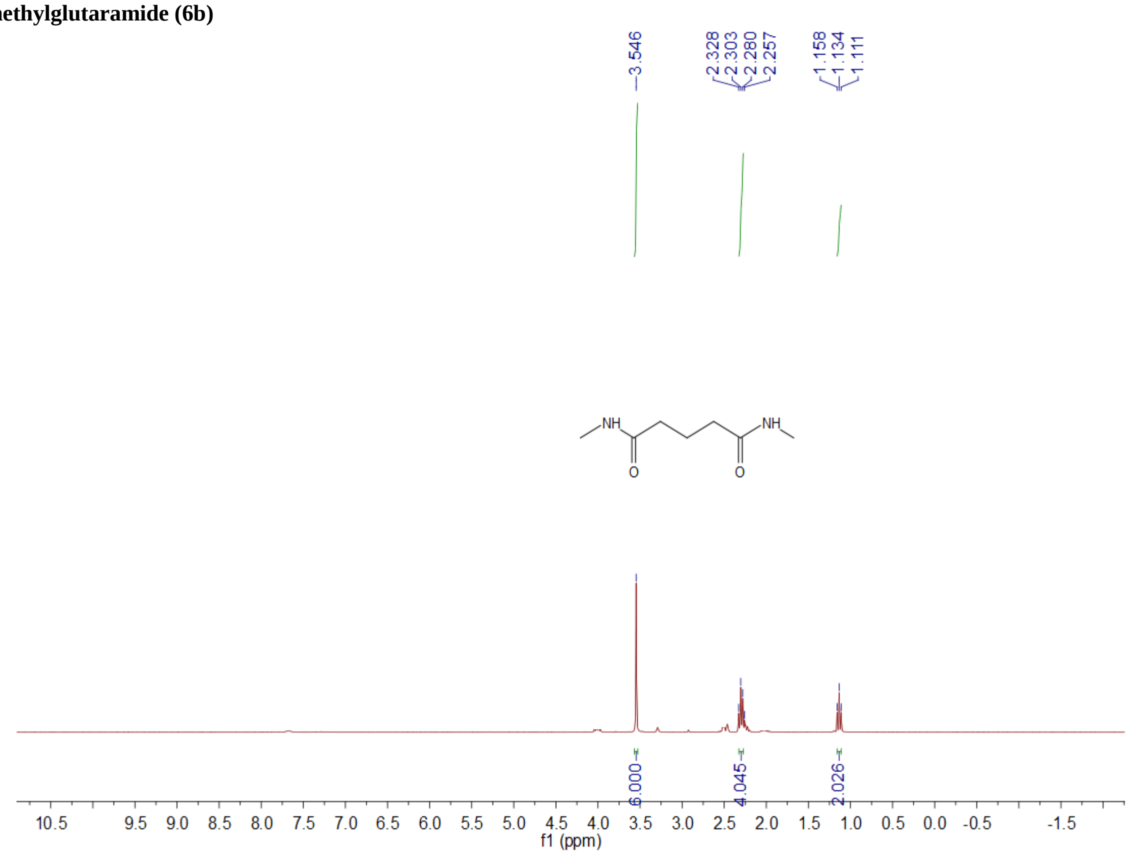

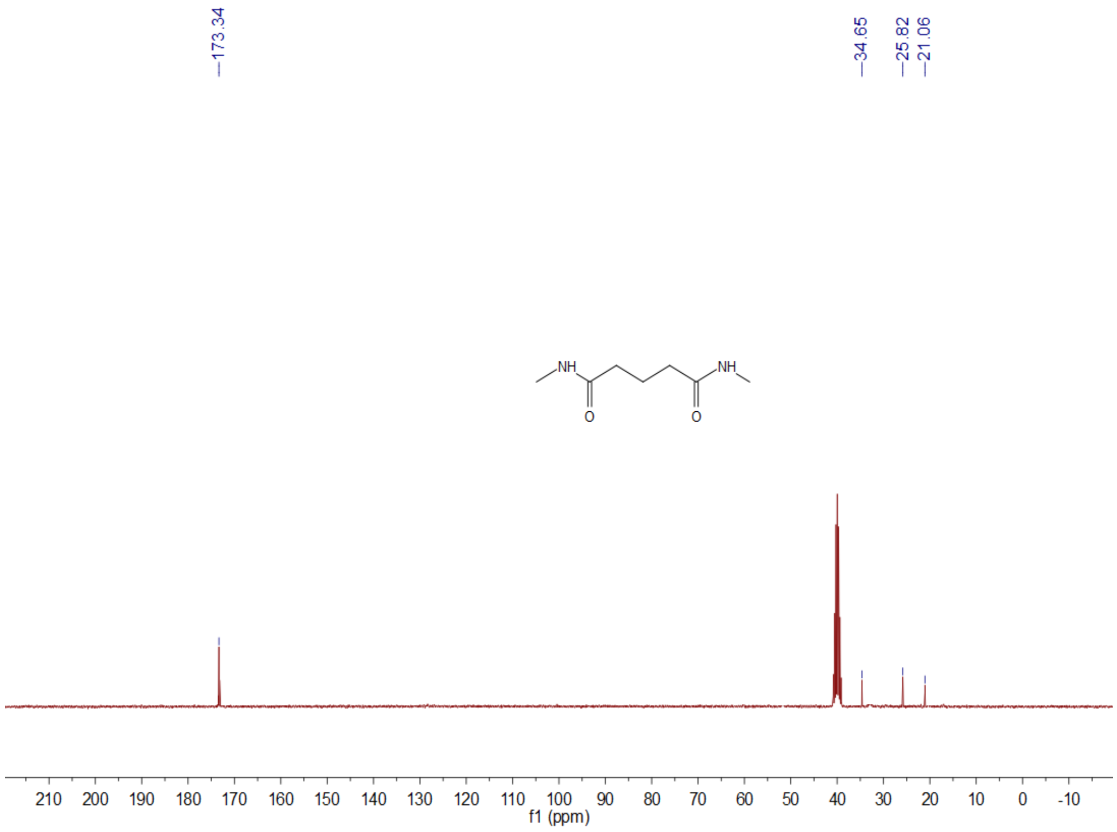

***N, N'*-Dibutylglutaramide (6c)**

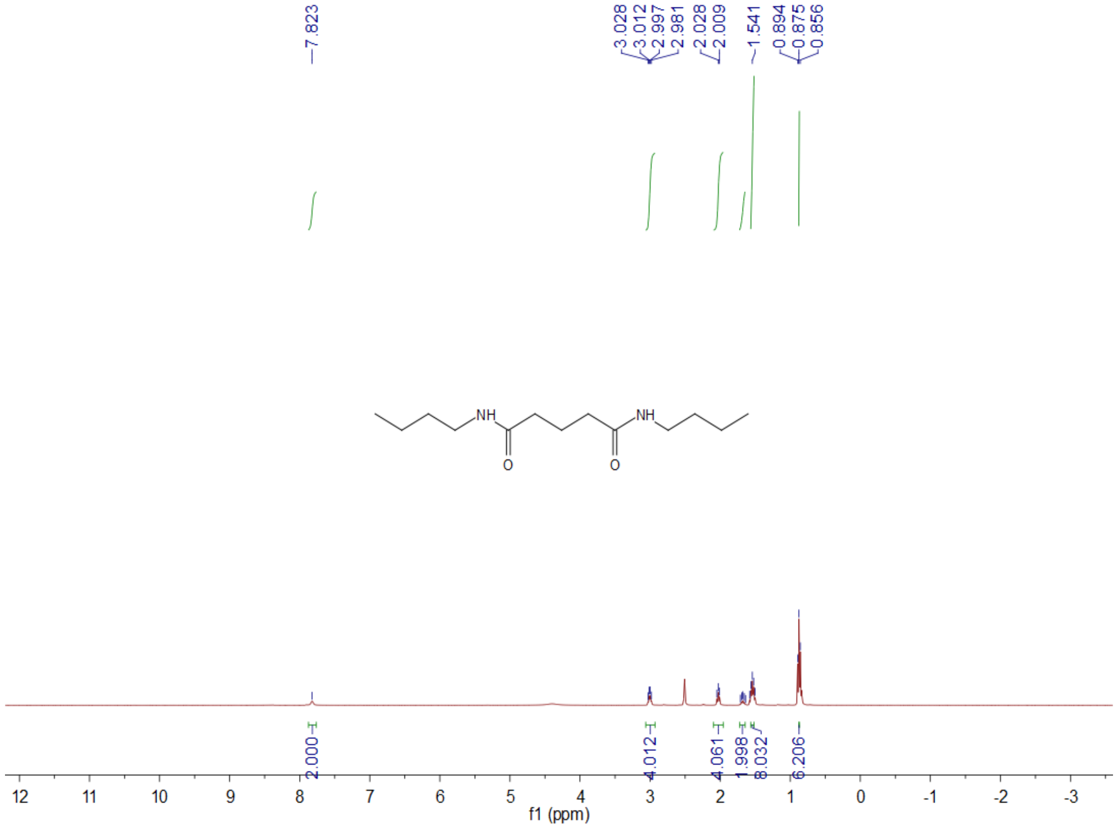

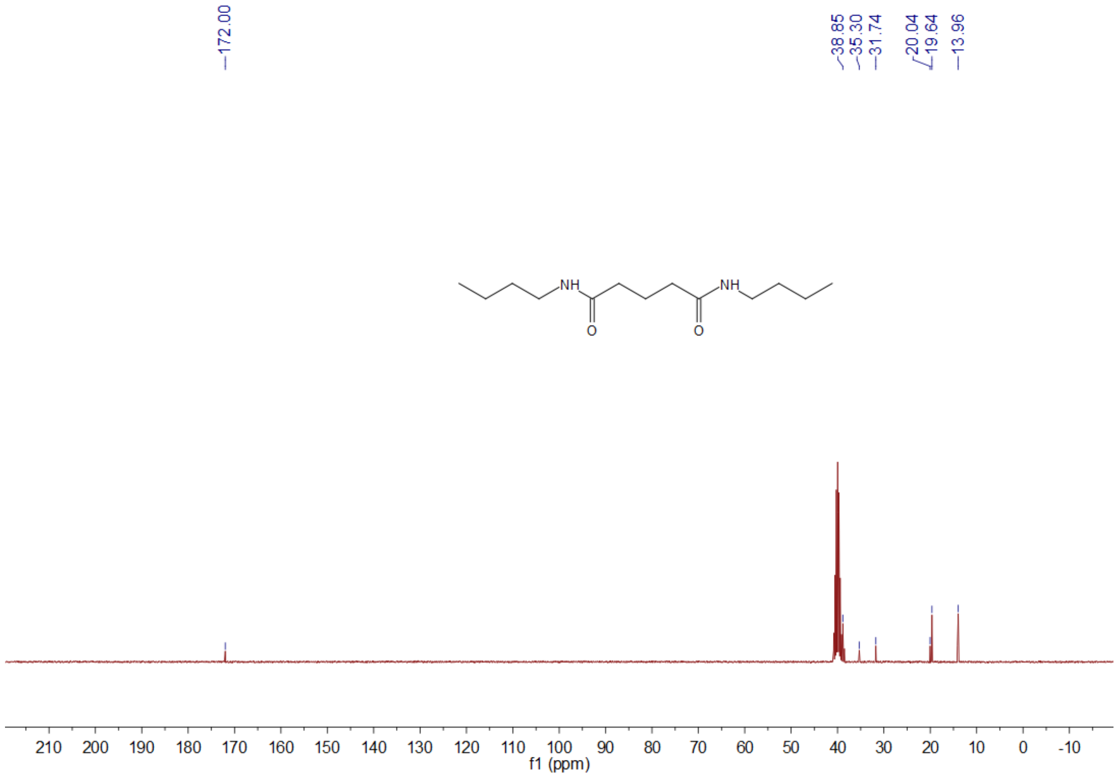

***N, N'*-Dibenzylglutaramide (6d)**

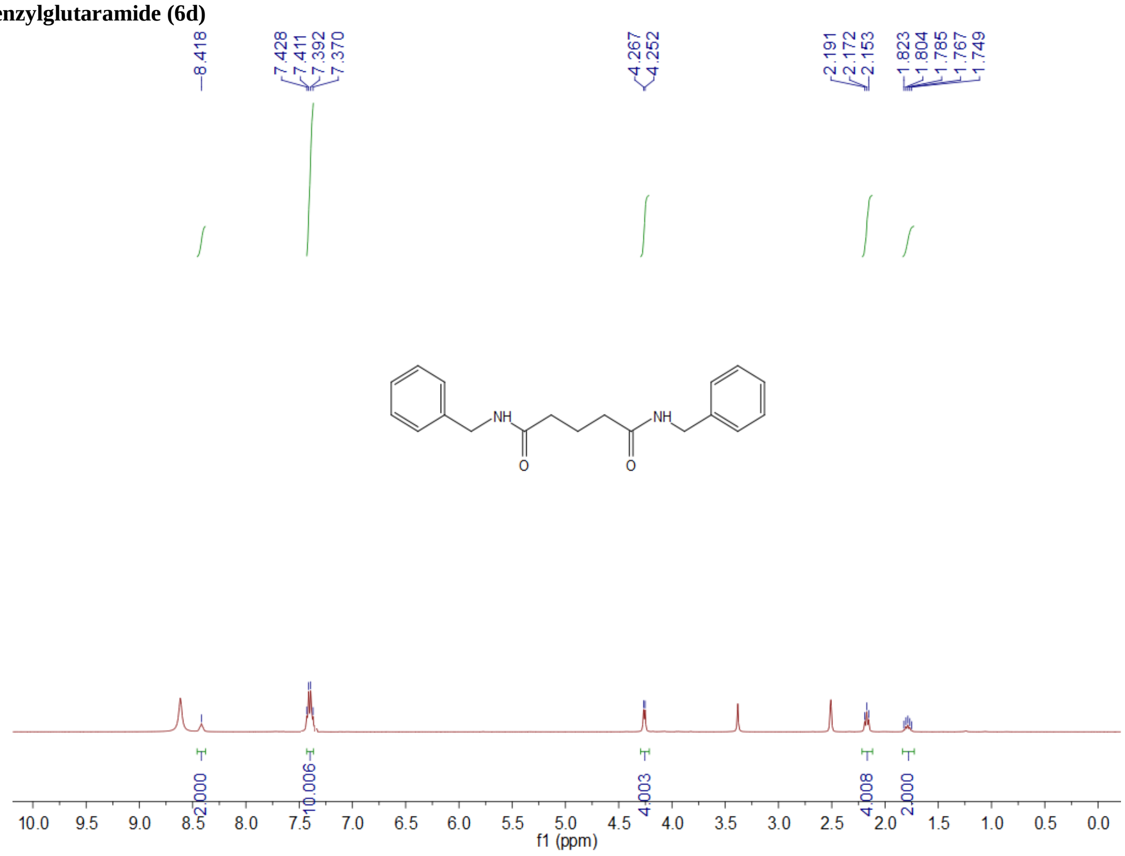

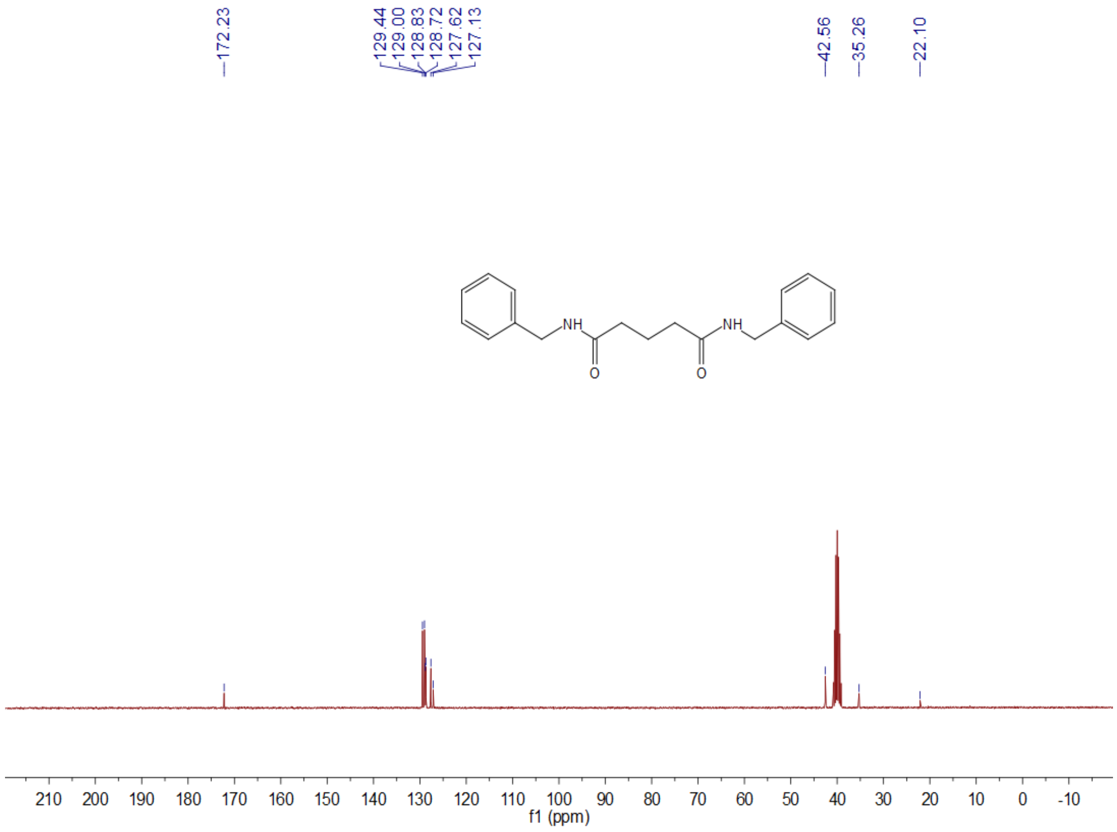

***N, N'*-Diphenylglutaramide (6e)**

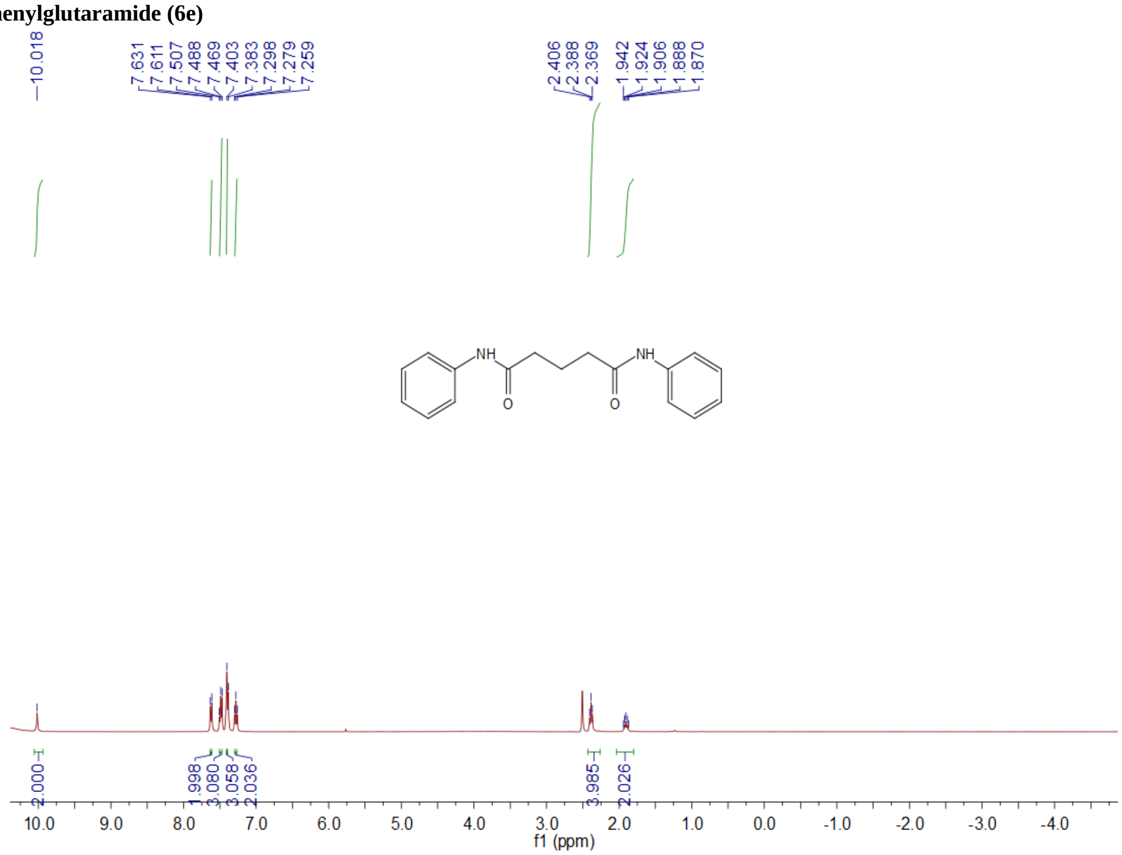

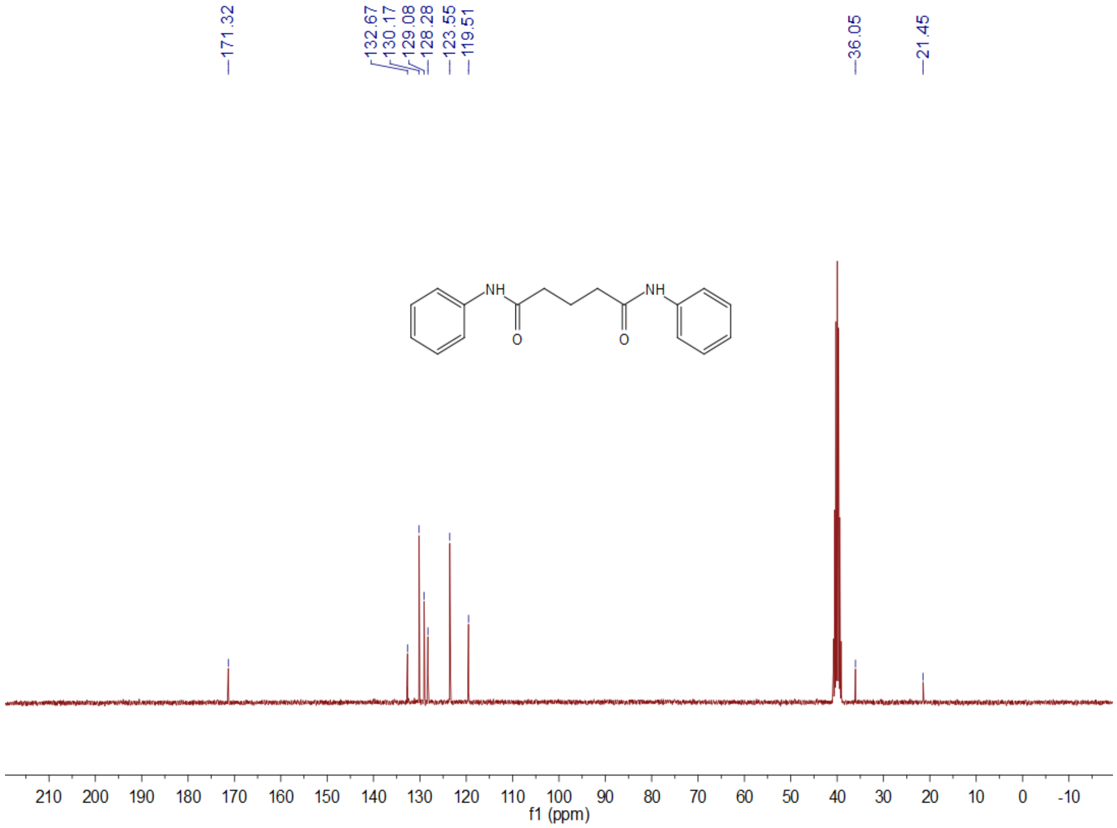

***N*, *N'*-Di-*p*-tolylglutaramide (6f)**

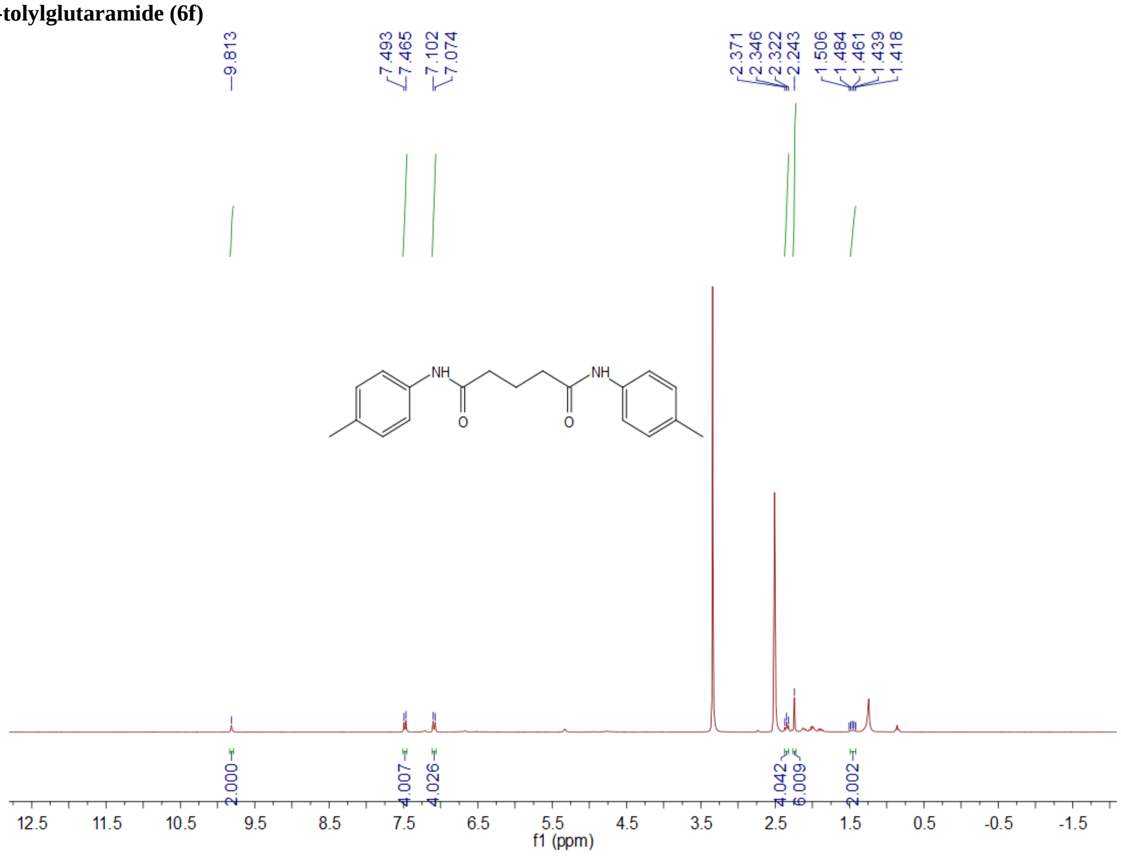

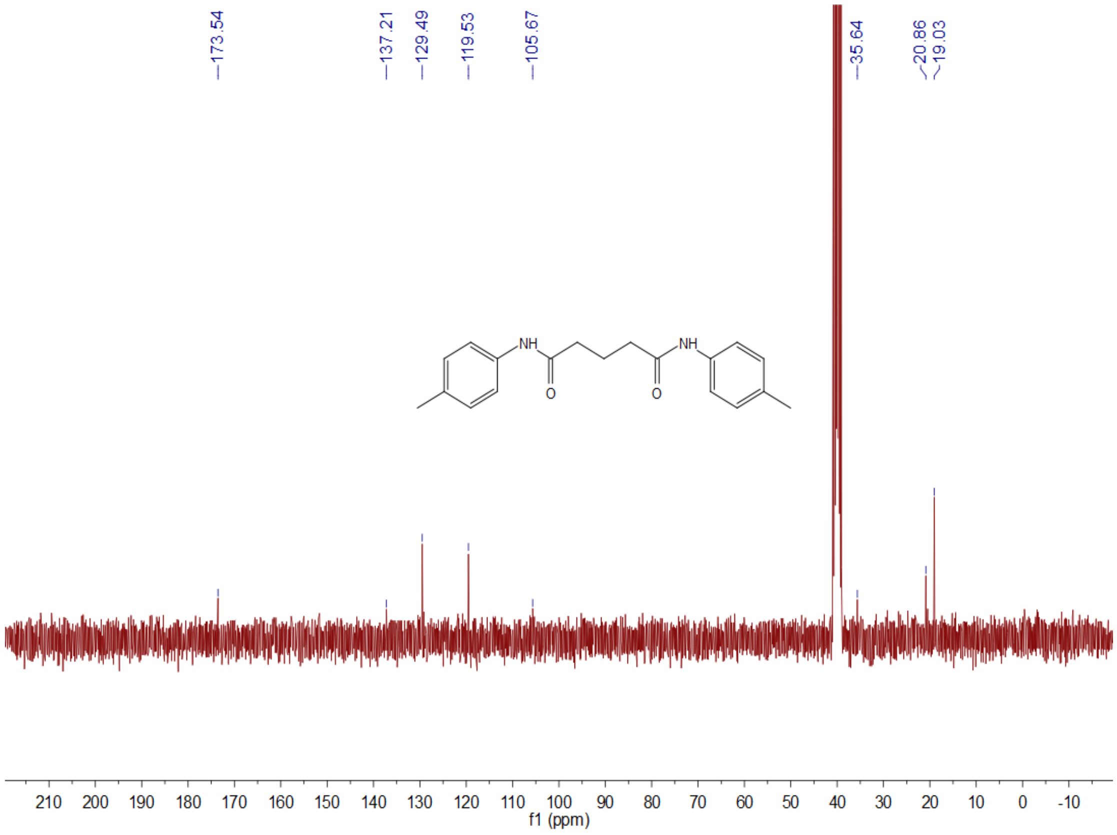

***N, N'*-Di-*m*-tolylglutaramide (6g)**

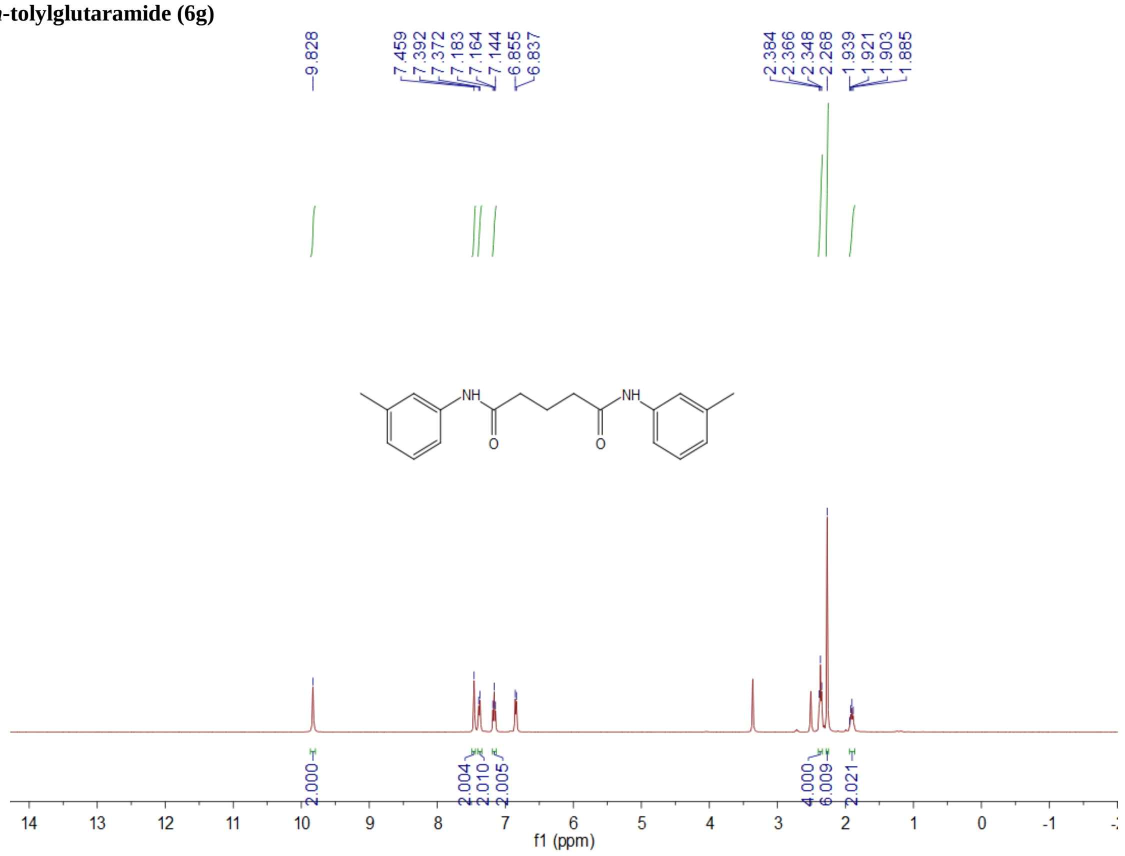

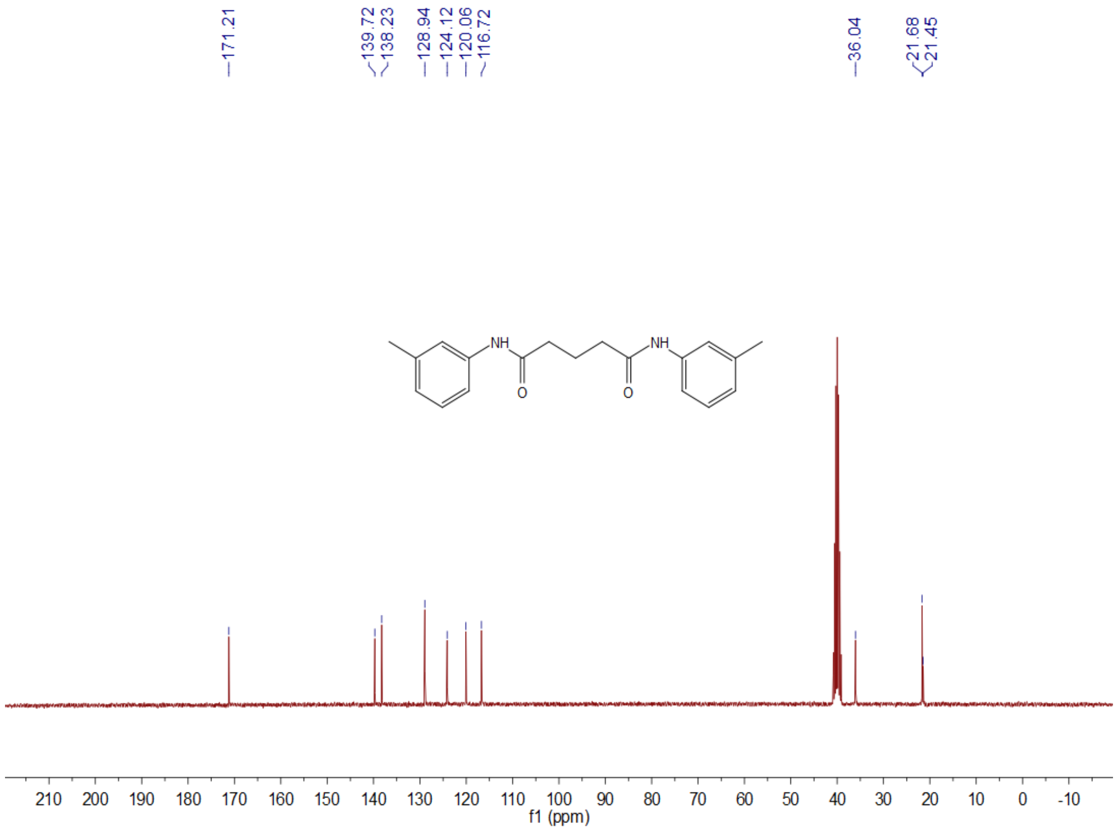

***N, N'*-Bis(4-methoxyphenyl)glutaramide (6h)**

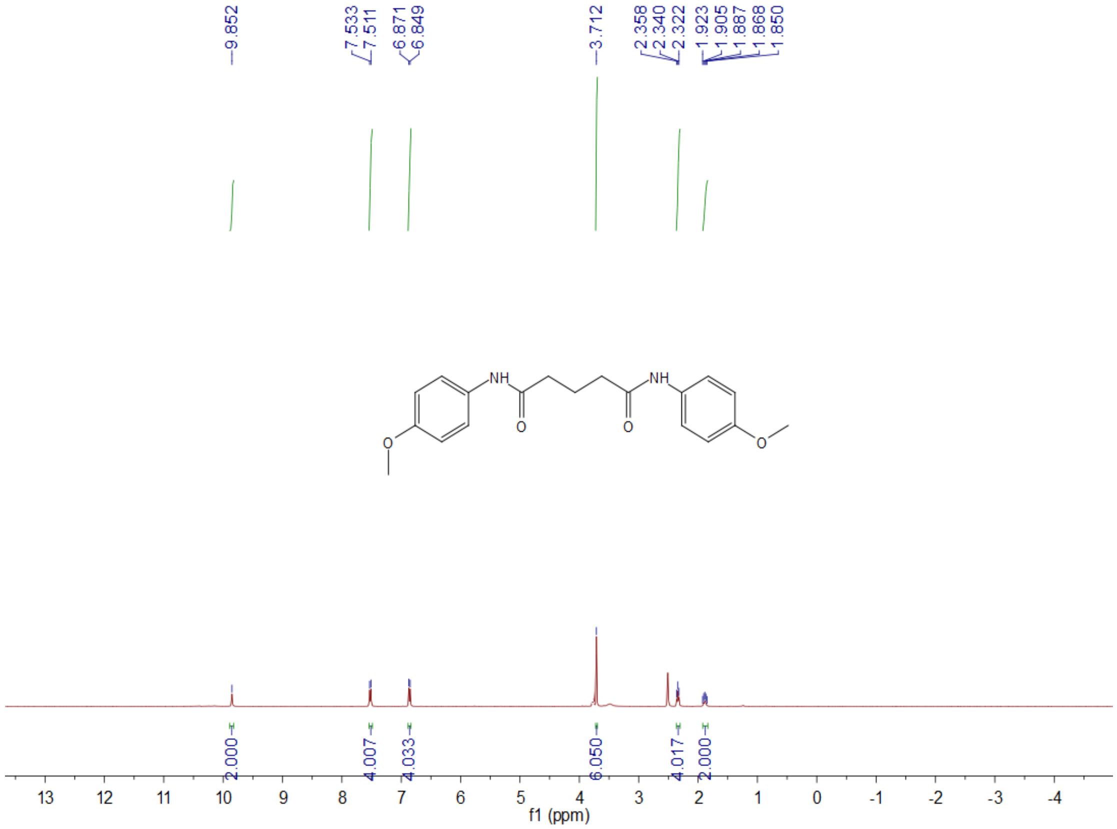

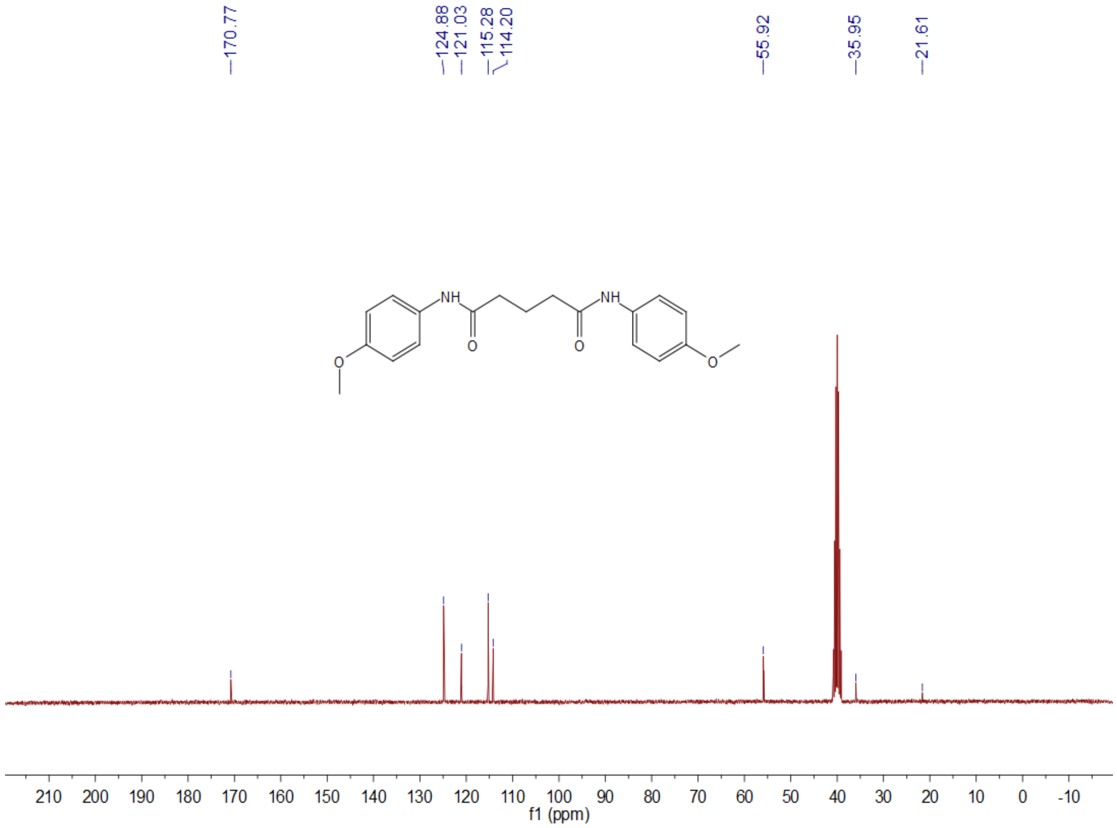

***N*, *N'*-Bis(4-fluorophenyl)glutaramide (6i)**

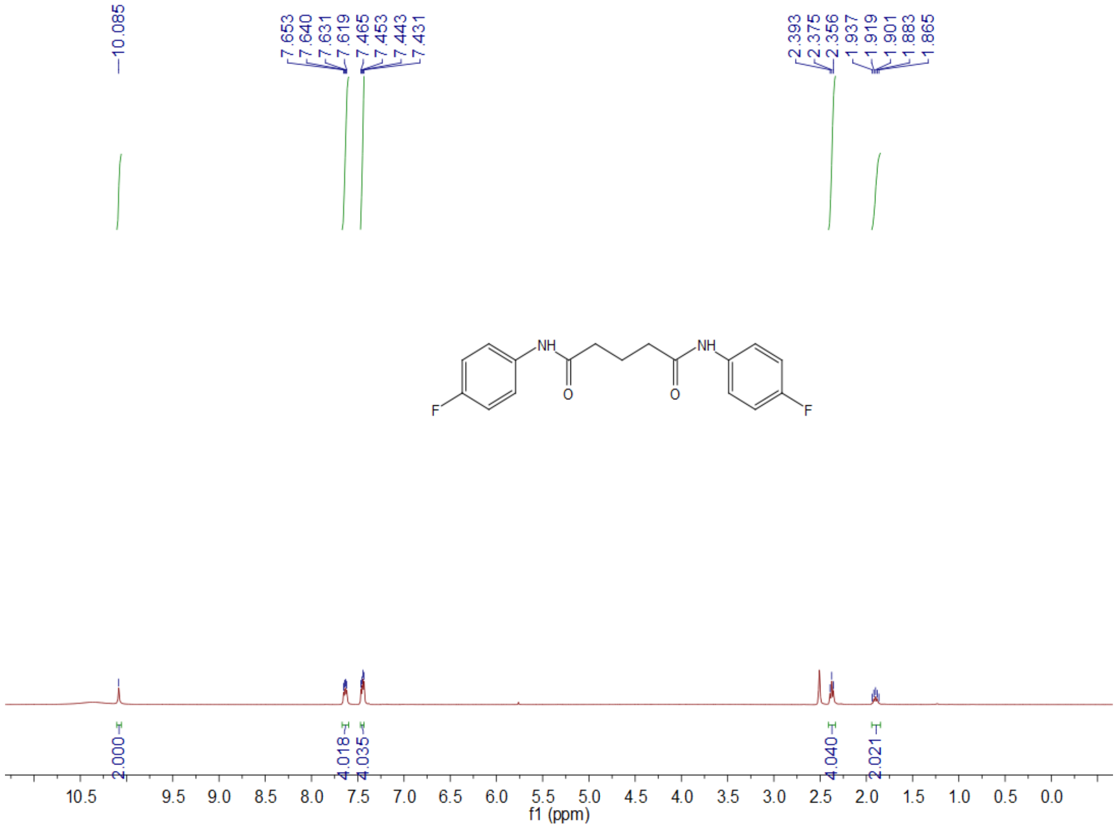

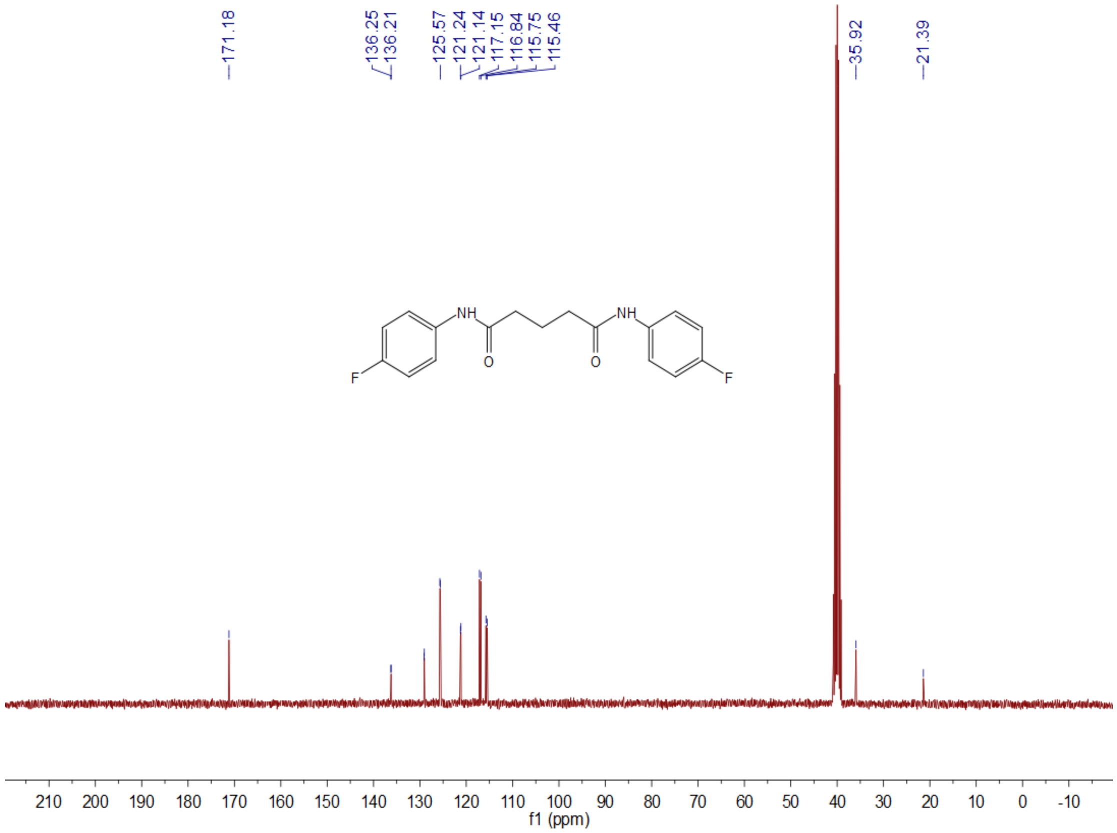

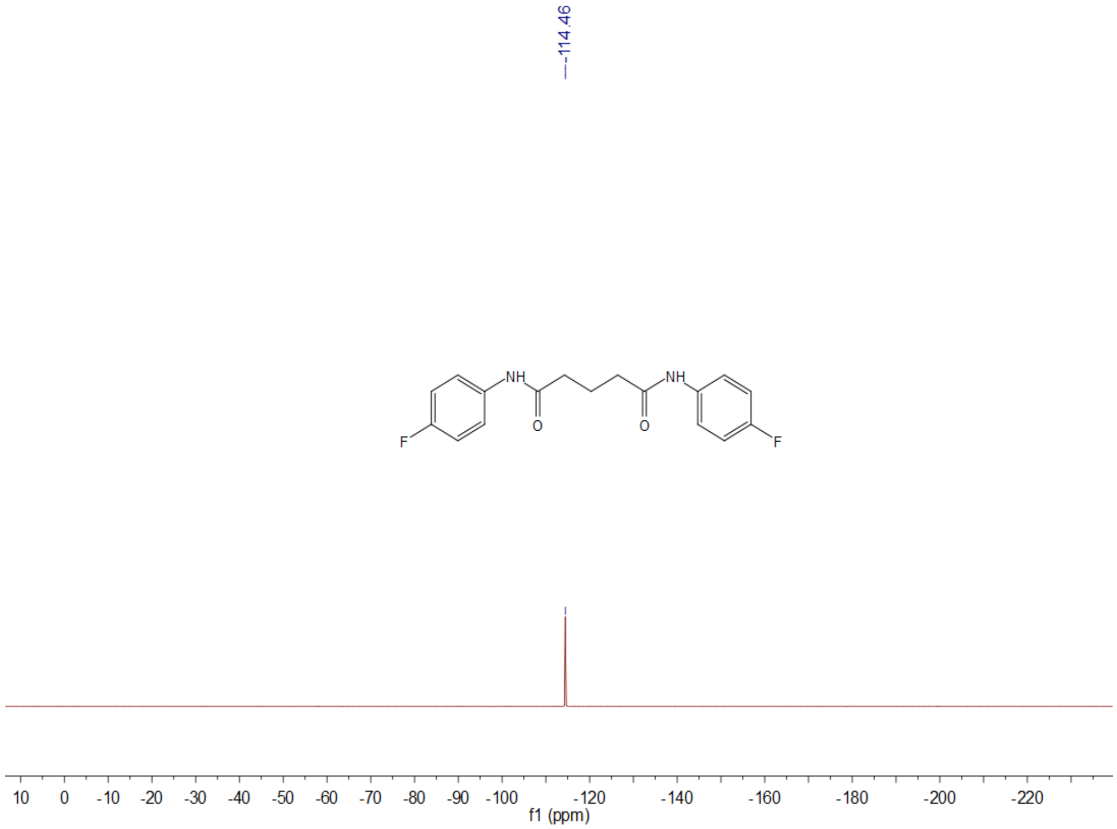
-114.46
NH
NH
F
F
O
O
10
0
-10
-20
-30
-40
-50
-60
-70
-80
-90
-100
-120
-140
-160
-180
-200
-220
f1 (ppm)

***N, N'*-Bis(4-chlorophenyl)glutaramide (6j)**

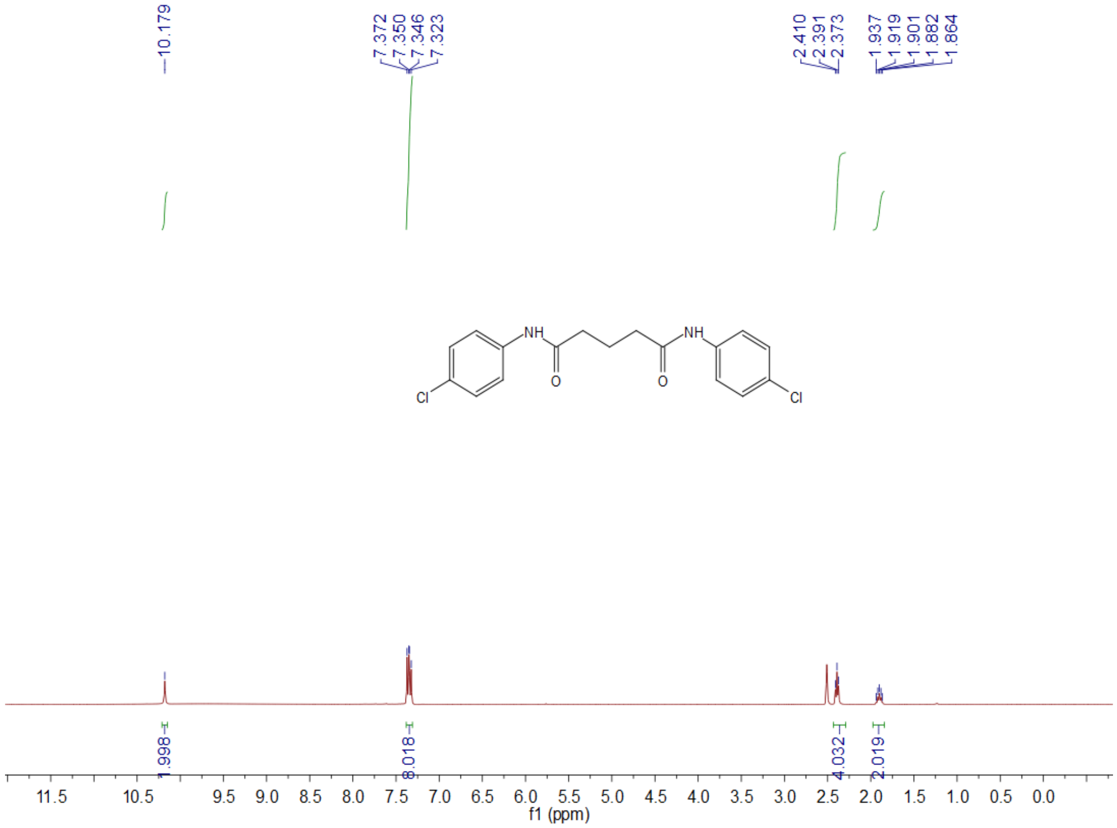

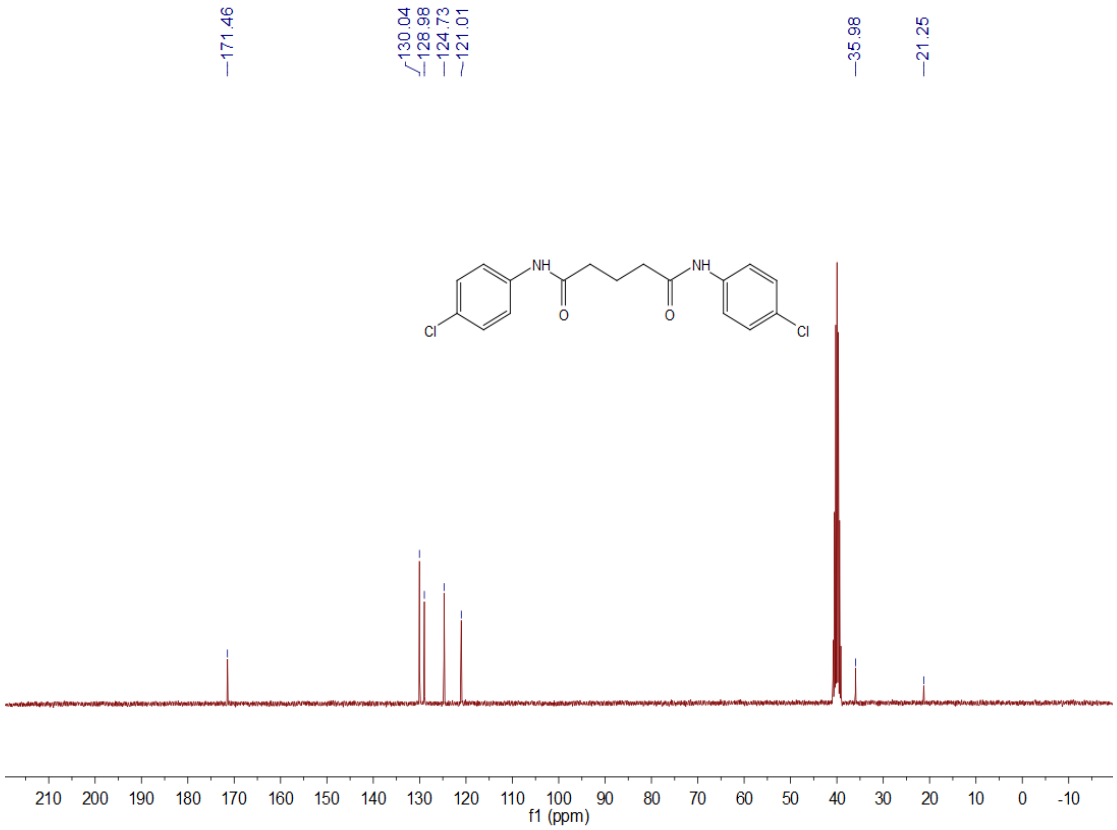

***N, N'*-Bis(4-bromophenyl)glutaramide (6k)**

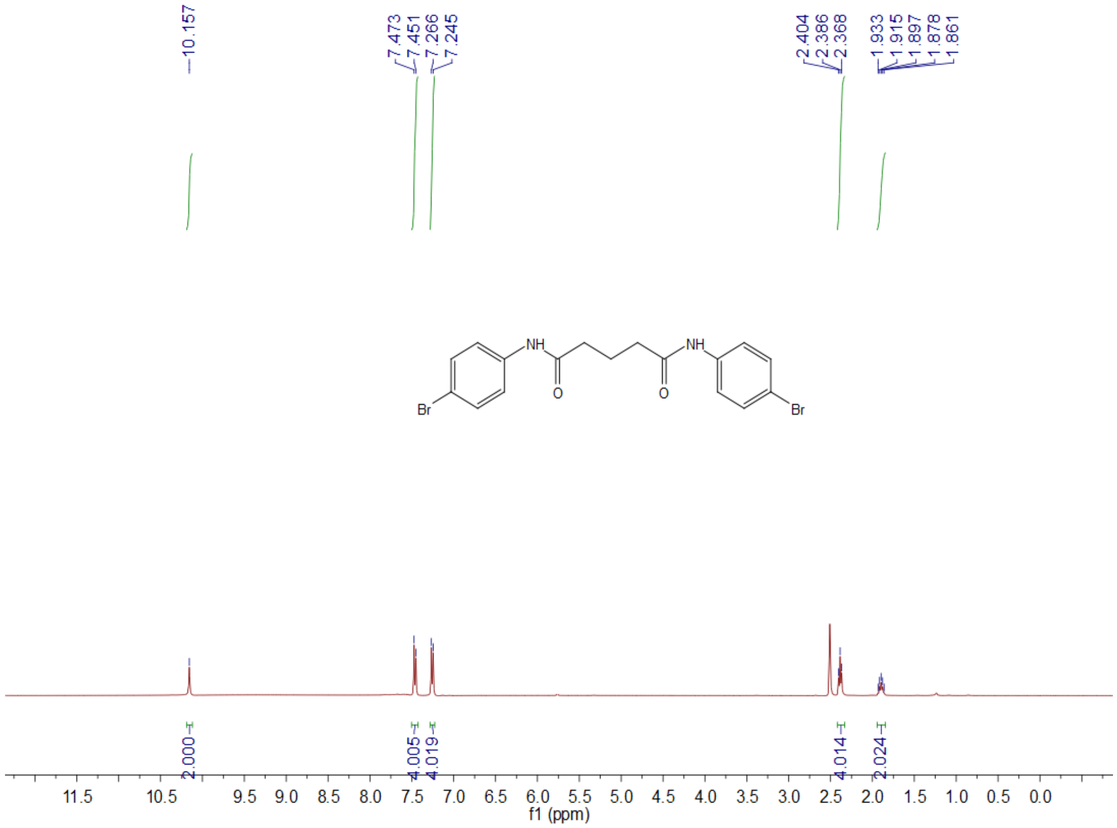

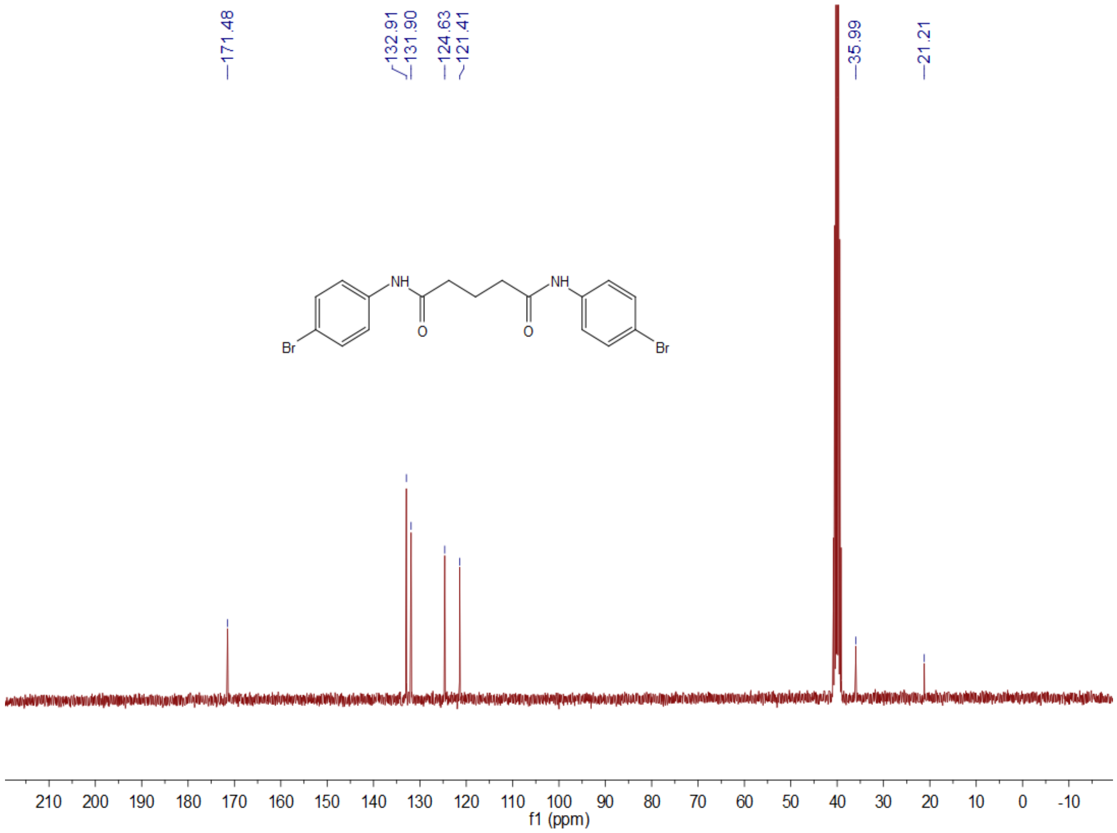

***N*, *N’*-Bis(4-iodophenyl)glutaramide (6l)**

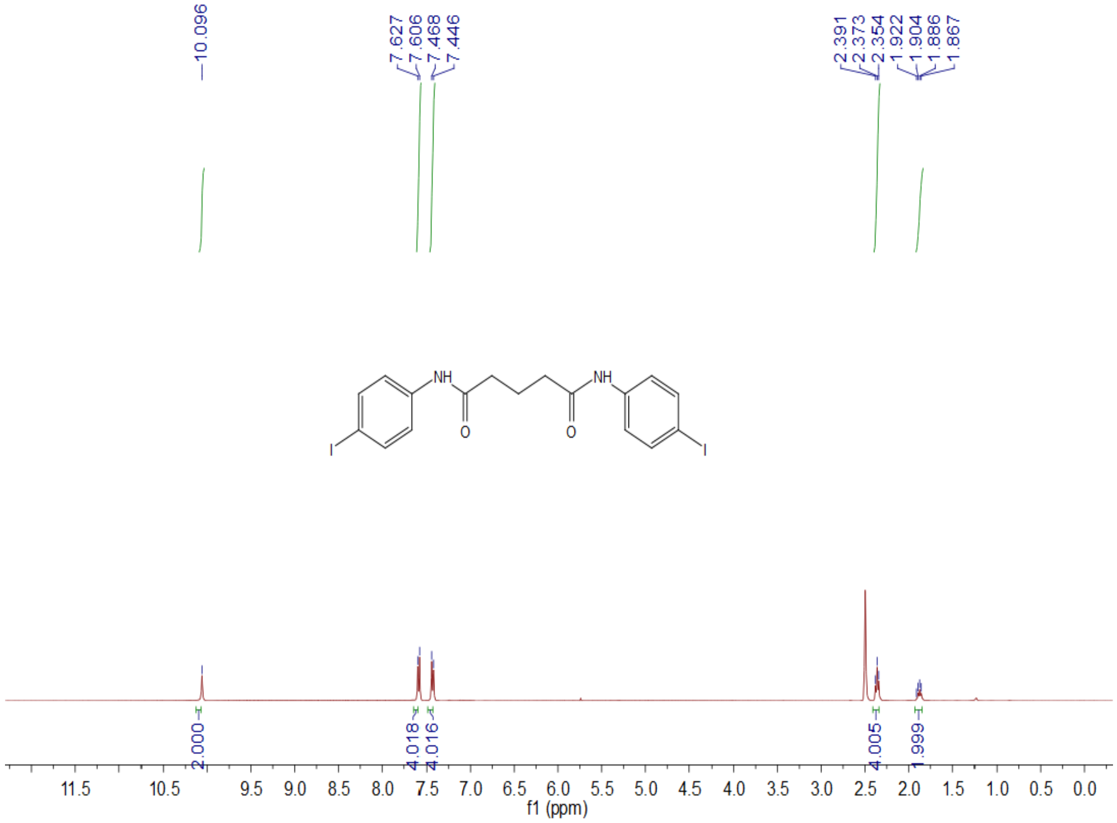

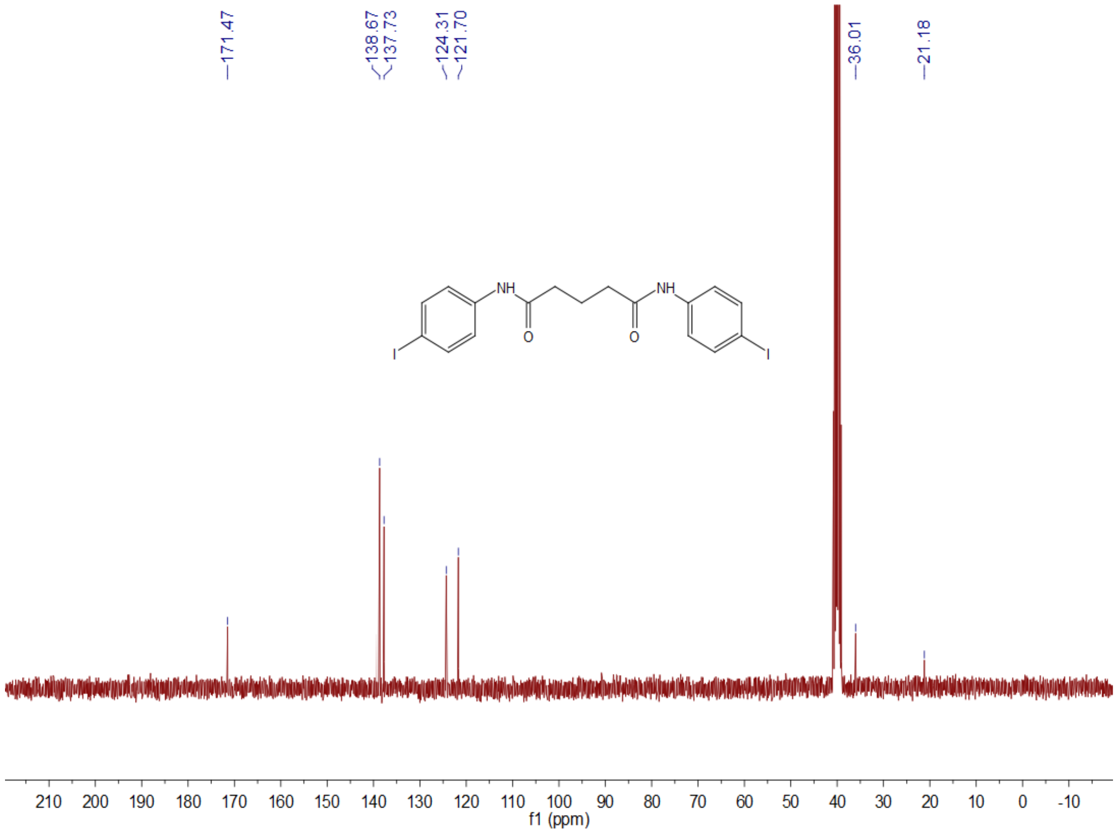

***N, N'*-Bis(4-(methylthio)phenyl)glutaramide (6o)**

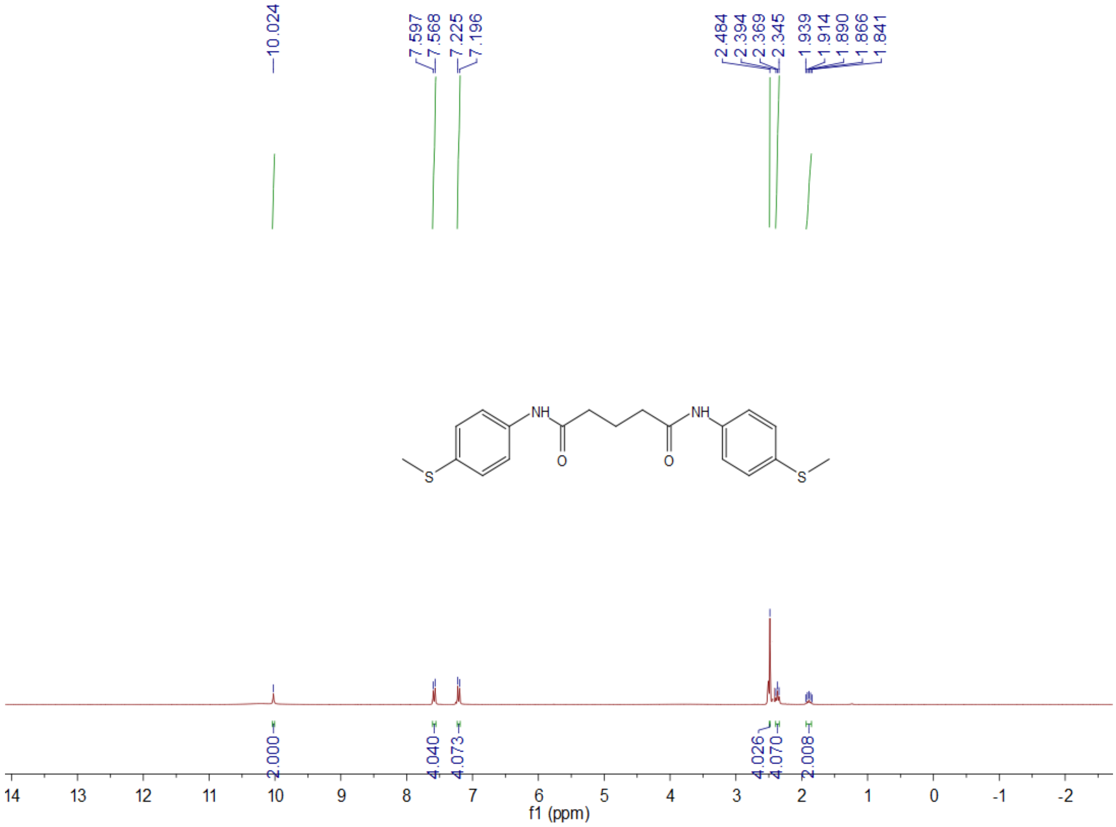

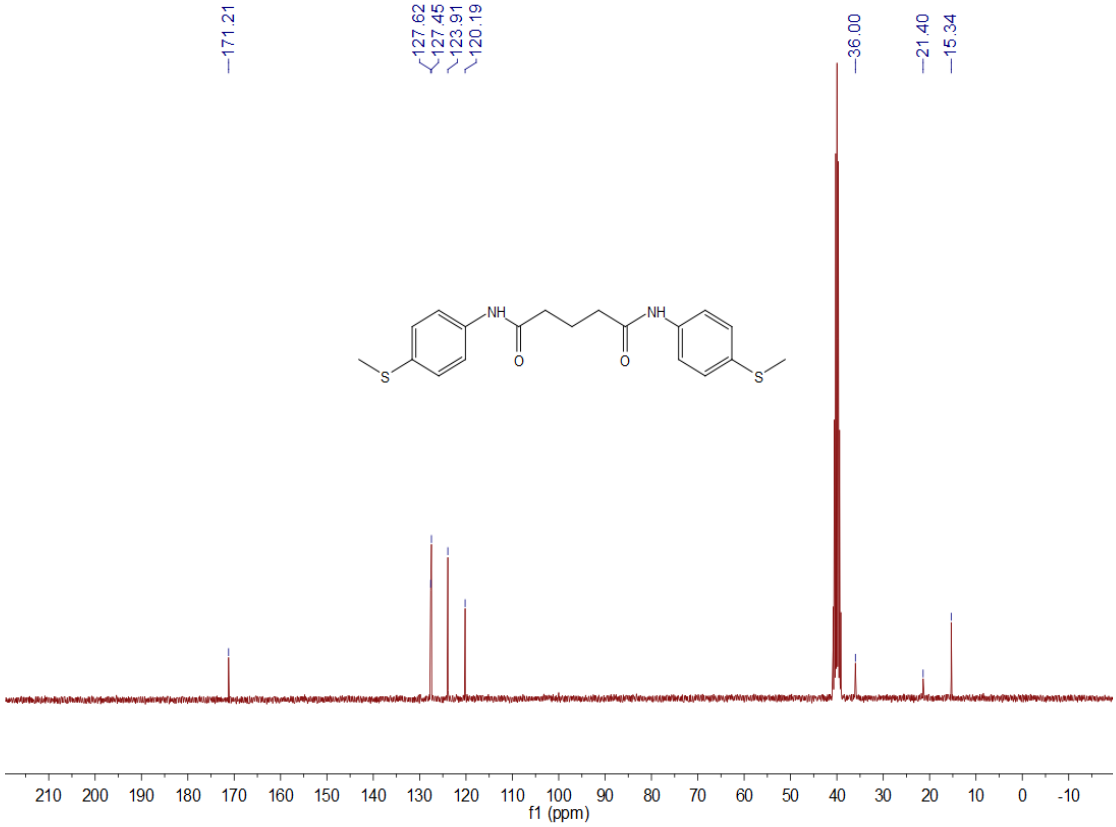

Azepane-2,7-dione (7a)

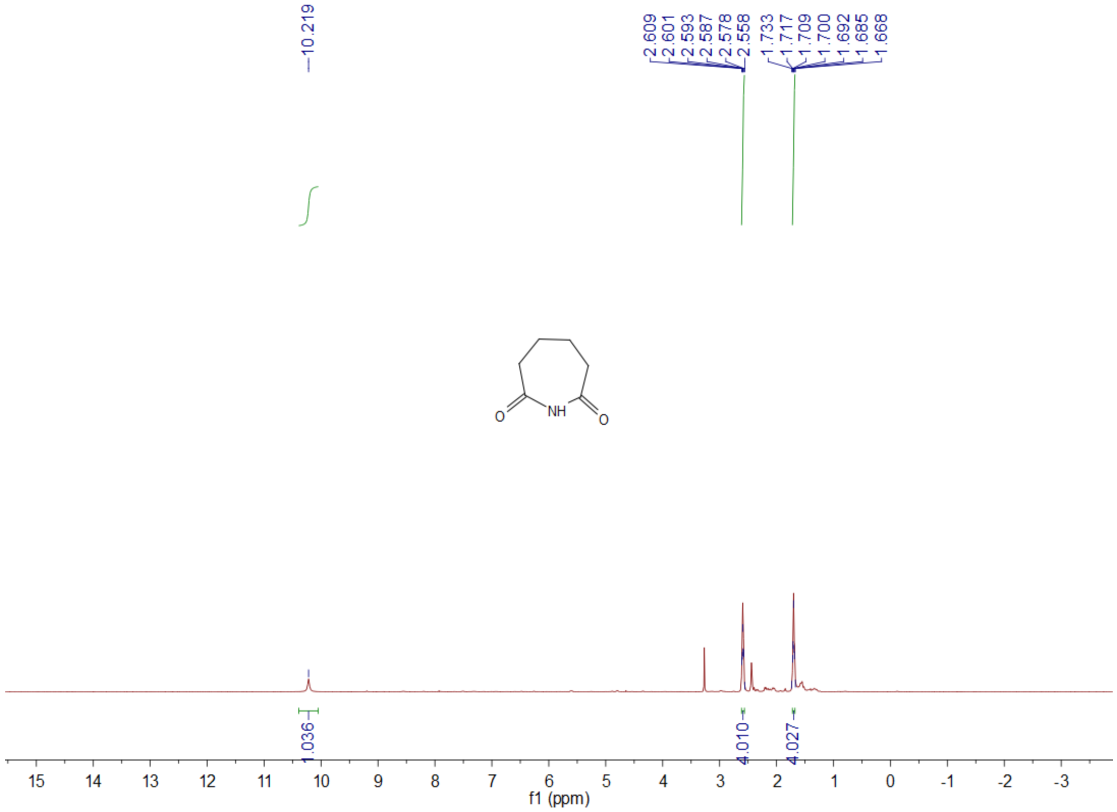

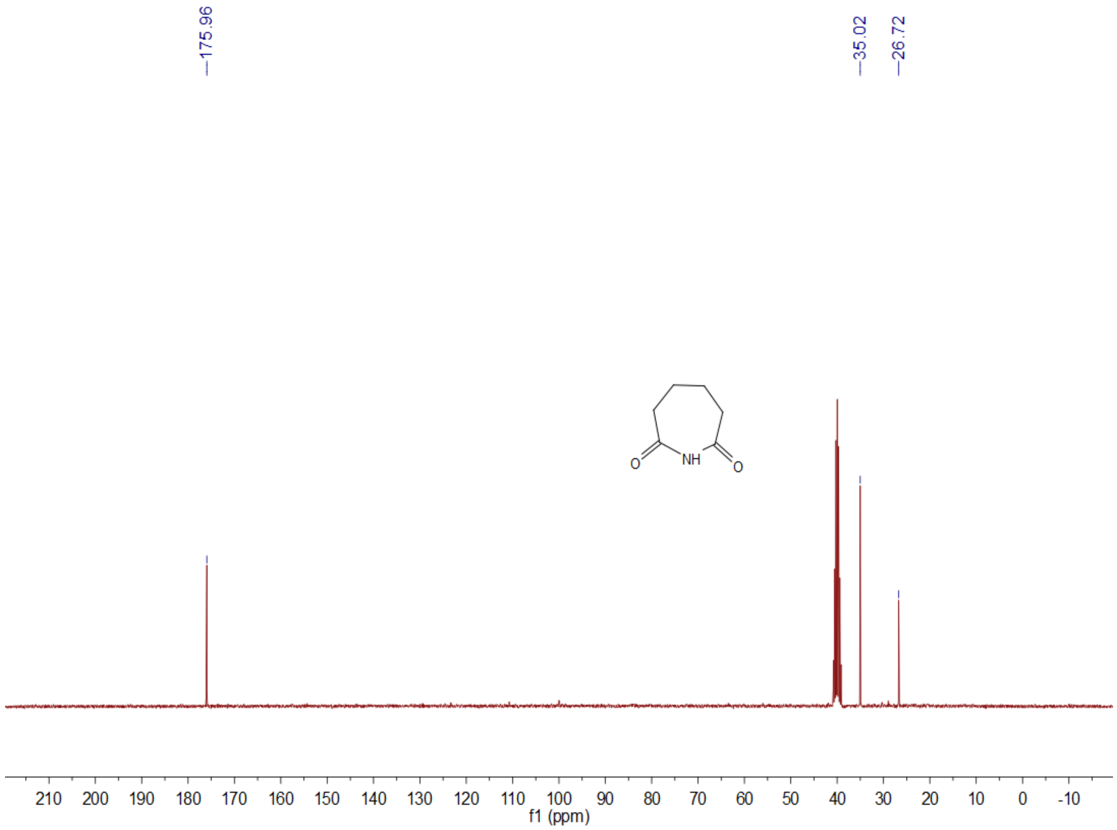

Adipamide (8a)

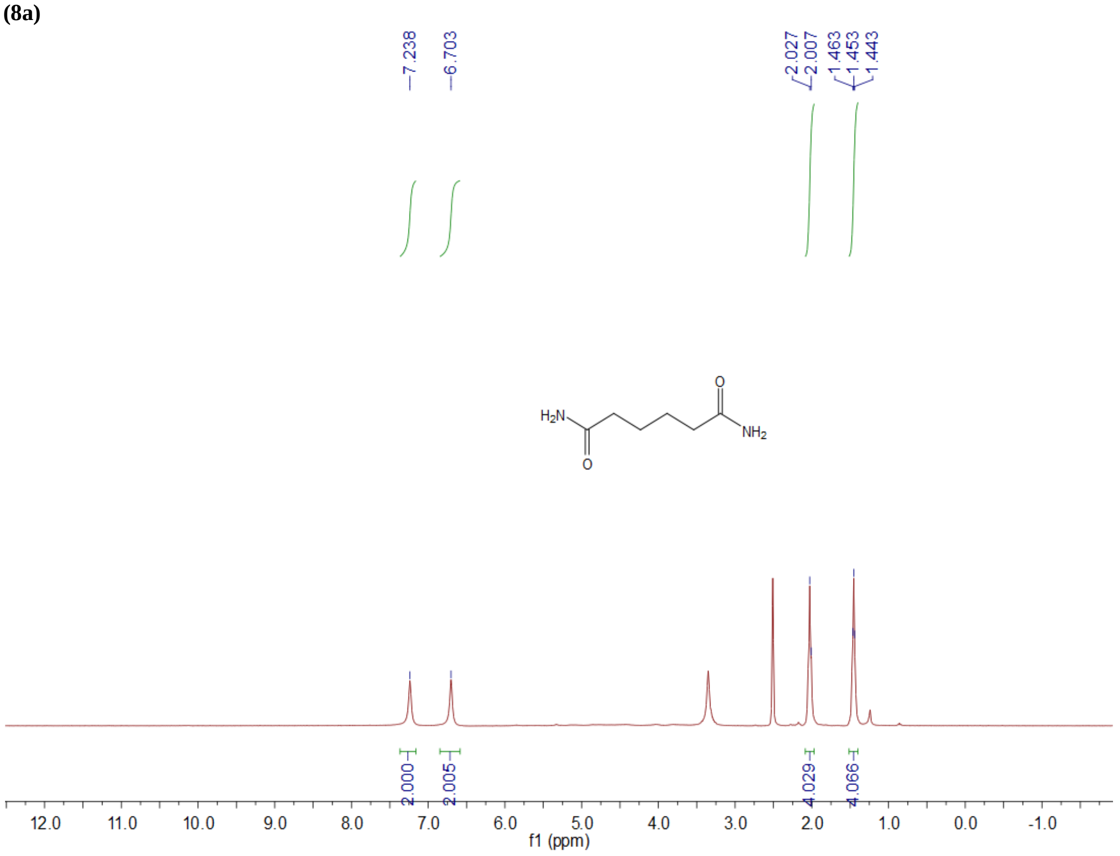

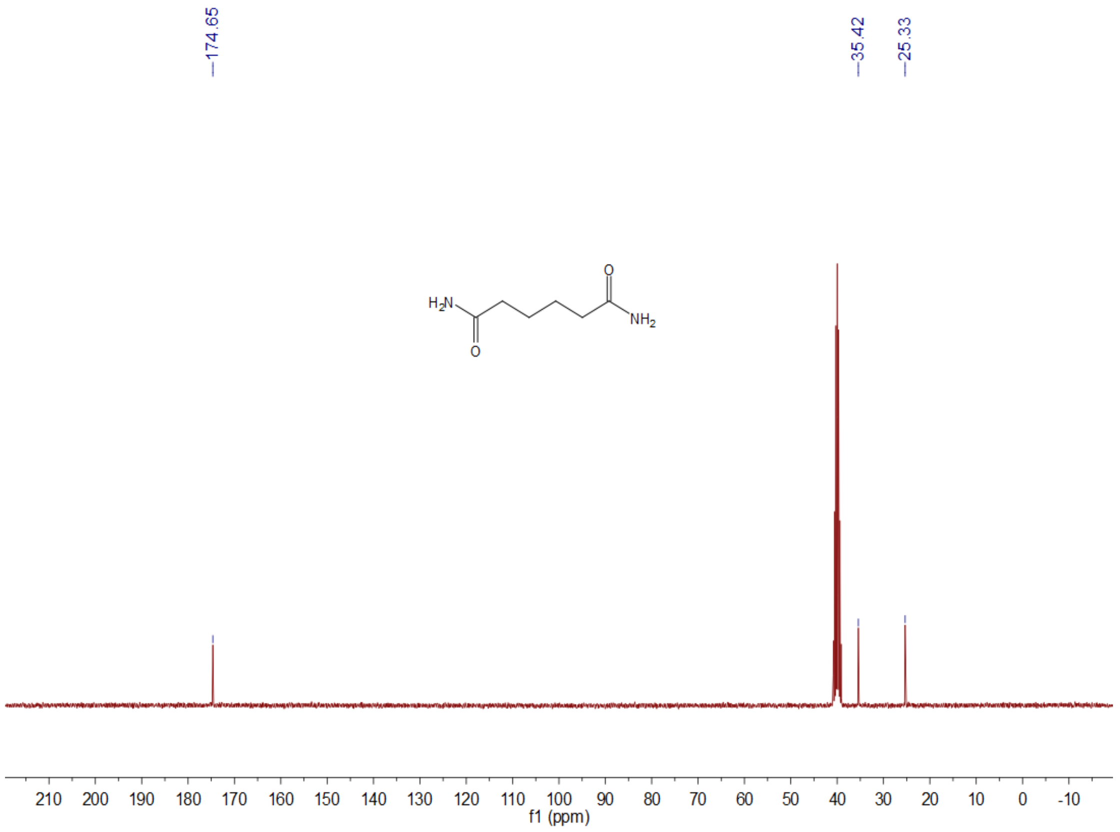

References

[1] Schönauer, E., Kany, A. M., Haupenthal, J., Hüsecken, K., Hoppe, I. J., Voos, K., Yahiaoui, S., Elsasser, B., Ducho, C., Brandstetter, H., Hartmann, R. W. *J. Am. Chem. Soc.* **139**, 12696–12703 (2017).
[2] Li, R., Zhao, Y., Wang, H., Li, D., Wu, Y., Zhang, H., Tang, M., Liu, Z. *ACS Sustainable Chem. Eng.* **9**, 2507–2514 (2021).
[3] Espinosa-Jalapa, N. A., Kumar, A., Leitus, G., Diskin-Posner, Y., Milstein, D. *J. Am. Chem. Soc.* **139**, 11722-11725 (2017).
[4] Ali, M. A., Moromi, S. K., Touchy, A. S., Shimizu, K. *ChemCatChem* **8**, 891-89 (2016).
[5] Ito, R., Umezawa, N., Higuchi, T. *J. Am. Chem. Soc.* **127**, 834-835 (2005).
[6] Houlihan, W. J., Gogerty, J. H., Ryan, E. A., Schmitt, G. *J. Med. Chem.* **28**, 28-31 (1985).
[7] Garad, D. N., Tanpure, S. D., Mhaske, S. B. *Beilstein J. Org. Chem.* **11**, 1008-1016 (2015).
[8] Liang, J., Lv, J., Fan, J., Shang, Z. *Synthetic Commun.* **39**, 2822-2828 (2009).
[9] Zhang, G. L., Yu, X. Q., You, J. S., Chen, L., Yan, M., Xie, R. G. *Synlett* **6**, 1095-1097 (2004).
[10] Hügel, H. M., Rix, C. J., Fleck, K. *Synlett* **14**, 2290-2292 (2006).
[11] Otsuka, S., Fujino, D., Murakami, K., Yorimitsu, H., Osuka, A. *Chem. Eur. J.* **20**, 13146-3149 (2014).
[12] Hurwitz, M. J., Exner, L. J., Benneville, P. L. *J. Am. Chem. Soc.* **77**, 3251-3253 (1955).
[13] Kunishima, M., Kawachi, C., Hioki, K., Terao, K., Tani, S. *Tetrahedron* **57**, 1551-1558 (2001).
[14] Fu, R., Yang, Y., Ma, Y., Yang, F., Li, J., Chai, W., Wang, Q., Yuan, R. *Tetrahedron Lett.* **56**, 4527-4531 (2015).
[15] Kubicova1, L., Waisser, K., Kunes, J., Kralova, K., Odlerova, Z., Slosarek, M., Janota, J., Svoboda, Z. *Molecules* **5**, 714-726 (2000).
[16] Kleber, C., Andrade, Z., Matos, R. A. F. *Synlett* **8**, 1189-1191 (2003).
[17] Patil, S. V., Mahale, K. A., Gosavi, K. S., Deshmukh, G. B., Patil, N. S. *Org. Prep. Proced. Int.* **45**, 314-320 (2013).
[18] Govindan, K., Chen, N. Q., Chuang, Y. W., Lin, W. Y. *Org. Lett.* **23**, 9419-9424 (2021).
[19] Shemchuk, L. A., Chernykh, V. P., Shemchuk, L. M. *Russ. J. Org. Chem.* **34**, 231-233 (1998).
[20] Naraoka, A., Naka, H. *Synlett* **30**, 1977-1980 (2019).
[21] Exner, L. J., Hurwitz, M. J., Benneville, P. L. *J. Am. Chem. Soc.* **77**, 1103-1105 (1955).
[22] Hoey, G. B., Lester, C. T. *J. Am. Chem. Soc.* **73**, 4473–4474 (1951).
[23] Minisci, F., Punta, C., Recupero, F., Fontana, F., Pedulli, G. F. *J. Org. Chem.* **67**, 2671–2676 (2002).
